# Small nucleolar RNAs signature (SNORS) identified clinical outcome and prognosis of bladder cancer (BLCA)

**DOI:** 10.1186/s12935-020-01393-7

**Published:** 2020-07-10

**Authors:** Rui Cao, Bo Ma, Lushun Yuan, Gang Wang, Ye Tian

**Affiliations:** 1 Department of Urology, Beijing Friendship Hospital, Capital Medical University, Beijing, 100050 China; 2 Department of Stomatology, Beijing Shijitan Hospital, Capital Medical University, Beijing, 100038 China; 3 Department of Internal Medicine, Division of Nephrology, Leiden University Medical Center, Leiden, 2333 ZA The Netherlands; 4 Department of Biological Repositories, Zhongnan Hospital of Wuhan University, Wuhan, 430071 China

**Keywords:** Bladder cancer, Small nucleolar RNA, Signature, Biomarker, TCGA

## Abstract

**Background:**

Small nucleolar RNAs (snoRNAs) are a new non-coding RNAs (ncRNAs), which have not been widely investigated and are identified to be involved in tumorigenesis. But the function of snoRNAs in BLCA has not been reported yet.

**Methods:**

SnoRNAs signature (SNORS) was constructed through LASSO cox regression analysis. Integrated analysis of candidate snoRNAs was performed to detect the correlation between copy number variation (CNV)/DNA methylation/protein/mRNA/alternative splicing (AS). Then we built a nomogram integrating independent prognostic factors to assist the clinical utility.

**Results:**

We have screened out 15 prognostic differentially expressed snoRNAs (DESs) and constructed SNORS consisting of 5 candidate snoRNAs which could appropriately stratify patients into low or high SNORS groups with distinct prognosis. Then we found 5 candidate snoRNAs might be regulated by their own CNV and DNA methylation. Moreover, 5 candidate snoRNAs were significantly correlated mRNA and alternative splicing (AS), which might regulate diverse biological process in tumorigenesis, such as “extracellular matrix”, “epithelial–mesenchymal transition (EMT)”, etc. signaling pathways. Furthermore, SNORS was an independent prognostic factor, which was strikingly correlated with clinical outcome. Through inporating with other variables, we have established a predictive nomogram, which was more effectively to predict prognosis than any other variables alone.

**Conclusion:**

Our findings first highlighted an important role of snoRNAs in BLCA and established a potential prognostic model which could serve as a biomarker for BLCA.

## Highlights

1. This is the first study focusing on snoRNAs in BLCA.
2. Integration analysis of 5 candidate snoRNAs indicated that they could be regulated with their own CNV/methylation, as well as influence function of mRNA, alternative splicing (AS) and protein.
3. The function annotation revealed that SNORS was enriched in “extracellular matrix”, “epithelial–mesenchymal transition (EMT)”, etc., which are so important for tumorigenesis of BLCA.
4. We have established snoRNAs signature (SNORS), which was an independent prognostic factor, could serve as a biomarker for BLCA.

## Background

Bladder cancer (BLCA) is a malignancy originated from urinary tract with high morbidity and mortality, which is reported as the 10th most common cancer with an estimated 549,000 new cases and 200,000 deaths in 2018 [1]. Epidemiological studies have identified that BLCA is a heterogeneous disease consisting of two major subtypes, non-muscle-invasive bladder cancer (NMIBC) and muscle-invasive bladder cancer (MIBC), which have distinct incidence and prognosis. NMIBC, which comprises the majority of BLCA, has not penetrated the detrusor muscle layer and is a not that life threatening with a ~ 90% five-year survival rate at most [2–4]. Transurethral resection of bladder tumour (TURBT) accompanied by intravesicular instillation of chemotherapeutics or Bacillus Calmette-Guerin (BCG) is the standard treatment for NMIBC [5], but not all the patients benefit from it. Moreover, non-responders will finally recur or even invade into the detrusor muscle layer, progressing to MIBC, which make the therapy of BLCA more complex. MIBC, which do not have a favorable prognosis with a five-year survival rate  < 50%, are more prone to metastasize and need systemic therapy combined with radical surgery and chemotherapy [6, 7]. Even with the rapid development of clinical imaging, chemotherapy and surgery, the treatment outcome of MIBC is not that satisfactory based on the current staging and grade system [8]. So it is very important to understand the potential mechanism and find some latent prognostic biomarkers for BLCA.

Recent years we have seen a rapid development of next generation sequencing and computational approaches, some protein non-coding RNA (ncRNA), such as microRNA (miRNA), long non-coding RNA (lncRNA), circular RNA (circRNA), piwi-interacting RNA (piRNA) and small nucleolar RNA (snoRNA), which was first thought to be transcriptional noise, now have been reported to play an important role in many biological process [9–12]. Among them, miRNAs and lncRNAs, which comprise the majority of ncRNAs, were largely studied. They could cooperate with each other to play a vital role in tumorigenesis of many types of cancer, such as hepatocellular carcinoma [13], oral cancer [14], pancreatic cancer [15], bladder cancer [16], and so on. But little attention has been paid to snoRNAs, which is another small ncRNAs with 60–300 nt in length mostly originated from introns of host genes in vertebrates [17, 18].

SnoRNAs interact with a set of ribonucleoproteins (RNPs) to form stable and functional snoRNPs particles to guide site-specific enzymatic modifications of other RNAs, including ribosomes (rRNAs), transfer RNAs (tRNAs), and small nuclear RNAs (snRNAs) [19, 20]. According to the differences in structure and modification approach, they are basically divided into two major subtypes: C/D box snoRNAs and H/ACA box snoRNAs [21, 22]. The C/D box snoRNAs, which consist of two major boxes, namely, C (RUGAUGA, R = purine) and D (CUGA), function as a guide for position-specific 2′-O-methylation of target molecules and are associated with four core proteins, methyltransferase fibrillarin (FBL), NOP56/NOL5A, NOP58/NOP5, and SNU13/NHP2L1, which constitute the core of C/D box snoRNPs [23–26]. Whereas, H/ACA box snoRNAs, which also have two specific hairpins and two short single-stranded regions called H and ACA boxes, are associated with pseudouridine synthase dyskerin (DKC1), GAR1, NHP2, and NOP10 to direct RNA pseudouridylation of target molecules [27, 28]. Moreover, there is another specific group of snoRNAs, called small Cajal body-specific RNAs (scaRNAs), which is gathered in Cajal bodies, could induce the post-transcriptional modification of spliceosomal RNAs [29]. Despite basic function of pseudouridylation and methylation of other RNAs, compelling study demonstrated that dysregulation of snoRNAs can also lead to the development and progression of various diseases such as Prader Willi syndrome, metabolic stress disorders and even cancers [30–32]. Chang et al. first reported h5sn2, which is an H/ACA box snoRNA, was strikingly downregulated in meningiomas compared with normal brain tissues [33]. Then more studies have suggested that snoRNAs participated in many biological processes of oncogenesis, such as proliferation, metastasis, and angiogenesis [34, 35]. All of these indicated that snoRNAs exhibited the potential to act as diagnostic biomarkers for prognosis and therapeutic targets. However, snoRNAs were reported to make some sense in variety type of cancer, but little is known whether snoRNAs influence the development of BLCA.

In this study, we have constructed a SNORS containing 5 candidate snoRNAs which could appropriately stratify patients into low or high SNORS groups with distinct prognosis using Least absolute shrinkage and selection operator (LASSO) Cox regression analysis. Furthermore, we found that candidate snoRNAs might be regulated by CNV/methylation. Moreover, candidate snoRNAs related mRNA, splicing and protein were reported to enrich in the “extracellular matrix”, “epithelial–mesenchymal transition (EMT)”, etc. signaling pathways. Finally, we evaluated potential functions and clinical utility of our prognostic model. Together, our findings highlight an important role of snoRNAs in BLCA and established a potential prognostic model which could serve as a biomarker for BLCA.

## Materials and methods

### Data collection and processing

The expression data of snoRNAs were downloaded from public database SNORic (http://bioinfo.life.hust.edu.cn/SNORic/), a website studied the function of snoRNAs in The Cancer Genome Atlas (TCGA). They mapped the reads to snoRNA from miRNA-sequencing data according to previous suggestion and normalized and quantified the expression of snoRNAs as reads per kilobase per million mapped reads (RPKM) [36, 37]. After filtering snoRNAs with average RPKM > 0.5 across all samples and removing the sample without complete survival information, we have finally obtained 392 tumour samples, 16 adjacent non-cancerous samples and 468 detectable snoRNAs in TCGA-BLCA cohort for further study. The data of corresponding clinical information, molecular subtypes, DNA methylation, CNV, mRNA, and protein data were all obtained from TCGA data portal (https://portal.gdc.cancer.gov/) or supplementary information from Robertson et al. [38]. The HTSeq-fragments Per Kilobase per Million (FPKM) data was transferred to the transcripts per million reads (TPM) which would represent expression of mRNA in TCGA-BLCA cohort. Then the mRNA with average expression value > 0.5 were retained for the subsequent analysis. Data were analysed with the R (version 3.5.2) and R Bioconductor packages.

### Identification of candidate snoRNAs

The candidate snoRNAs were screened out from two strategies. First, differentially expressed snoRNAs (DESs) between BLCA and adjacent non-cancerous samples were measured with the bioconductor package “Linear Models for Microarray Data (*limma*)” in R. The threshold for the “*limma*” test to select the significantly DESs was defined as |Log(fold change)| (logFC) ≥ 1 and a adjusted p value (adj.P.Val) < 0.05. Second, univariate Cox regression analysis was performed to select prognostic candidate snoRNAs and threshold was defined as p < 0.05. After merging DESs with prognostic snoRNAs obtained from above analysis, remained snoRNAs were identified as candidate snoRNAs for further study.

### Establishment and validation of prognostic snoRNAs signature (SNORS)

All patients were randomly divided into training and testing sets at cut-off 7:3. Then candidate snoRNAs were submitted to LASSO Cox regression analysis based on package “*glmnet*” in R to construct snoRNAs optimal prognostic signature (SNORS) in BLCA [39]. The formula for SNORS risk-score = $\sum_{i = 1}^{n}$ (coef_i_ × Expr_i_). The Expr_i_ is the relative expression of snoRNAs for patient i and coef_i_ is the LASSO Cox coefficient of the snoRNA i in each cohort. Then all patients were separated into low or high SNORS groups at the median cut-off. Kaplan–Meier (KM) survival analysis were utilized to detect the difference of prognosis, including overall survival (OS), recurrence free survival (RFS), disease free survival (DSS), progression free interval (PFI), between high/low SNORS patients and distinct stratified clinicopathological characteristics through package “*survminer*” in R. Time-dependent receiver operating characteristic (ROC) analysis was used to evaluate the prediction accuracy and ability of the signature, and the area under curve (AUC) for 1-year, 3-year and 5-year OS, RFS, DSS and DFI was measured through package “*survivalROC*” in R [40]. Furthermore, the correlation between SNORS with corresponding clinicopathological characteristics, including age, gender, grade, subtype, lymphonodes positive by hematoxylin and eosin (HE), lymphovascular invasion status, pathological T stage, pathological N stage, pathological M stage, pathological tumour stage, and clinical outcome, including neoplasm cancer status, new tumour event, primary therapy outcome and additional treatment outcome, as well as molecular subtypes [41–43] including UNC, MDA, CC, TCGAcluster and Lund were measured by t-test/one-way ANOVA test or *χ*^2^ test and shown by violin plot or cluster heat map. * p < 0.05, ** p < 0.01, *** p < 0.001.

### Integration analysis of candidate snoRNAs

As the expression of genes is significantly associated with their own CNV and DNA methylation, the correlation between candidate snoRNAs and CNV/DNA methylation was measured [44, 45]. Furthermore, snoRNAs were reported to be involved in regulating expression and activity of mRNA, proteins and alternative splicing (AS) [29, 46, 47], the correlation between them was also analysed. The PSI values for splice events on samples, which are a common intuitive ratio for quantifying splicing events, were downloaded from TCGA SpliceSeq (http://bioinformatics.mdanderson.org/TCGASpliceSeq) [48] to investigate correlation between snoRNAs and splice events. Spearman correlation analysis were utilized to measure the association between candidate snoRNAs with above molecular data, and the coefficient |Rs| ≥ 0.3 and FDR < 0.05 were defined as statistical significance.

### Functional and annotation analysis

Candidate snoRNAs related mRNA and alternative splicing (AS) were determined with spearman correlation analysis. The prognostic mRNAs screened out with univariate cox regression analysis were performed functional enrichment analysis in various Gene Ontology (GO) categories and Kyoto Encyclopedia of Genes and Genomes (KEGG) processes by using package “*ClusterProfiler*” in R [49]. Then, survival related mRNAs were performed protein–protein interactions (PPI) analysis with STRING tool (https://string-db.org/) to further investigate the potential function of these snoRNAs. Moreover, the snoRNAs associated AS mRNAs were also measured with above analysis. GO and KEGG terms/pathways with adjusted p‑values and q-value < 0.05 were considered to be significantly enriched signalling pathway/terms for these snoRNAs. Furthermore, the Hallmark gene sets (a total of 50 independent gene sets), which represent the most valuable and important signalling pathway in humans, were downloaded from the MSigDB of Broad Institute (http://software.broadinstitute.org/gsea/index.jsp) [50]. Then we conducted Gene Set Variation Analysis (GSVA) to analyse the enrichment of biological process and pathways due to SNORS through package “*GSVA*” in R [51]. The significantly enriched Hallmark gene sets were determined as p value < 0.05 and t value > 1. In addition, we performed Single Sample Gene Set Variation Analysis (ssGSEA) to calculate the ssGSEA score of each Hallmark gene set in TCGA-BLCA cohort and spearman correlation analysis were used to evaluate the correlation between SNORS and each Hallmark gene set.

### Construction of a prognostic nomogram

Independent prognostic factors were identified through univariate and multivariate Cox regression analysis by merging SNORS with other clinicopathological characteristics and visualized via package “*forestplot*” in R. Then all screened out independent prognostic factors were combined to construct nomogram, which is scoring system used to predict the clinical outcome by integrating with prognostic parameters, through package “*rms*”, “*nomogramEx*” and “*regplot*” in R [52]. According to the nomogram, patients will get total point by plus single point from multiple parameters and patients with higher total points were with worse prognosis. Furthermore, decision curve analysis (DCA) and calibration curves were detected to check the reliability of our nomogram.

### Statistical analyses

Unpaired Student t test or one-way Anova test was utilized to estimate statistical significance for parameters between two groups or more than two groups respectively. The χ^2^ test was applied to analyse the correlation between SNORS and clinical parameters. Kaplan-Meier (KM) survival curves were used to detect differences of survival rates between different groups through the package “*survminer*” in R. Time-dependent ROC were utilized to evaluate the predictive accuracy through package “*survivalROC*” in R. The correlation between snoRNAs and indicated molecular data was measured by spearman correlation analyses. Independent prognostic factors, which were identified by univariate and multivariate cox regression analysis, were integrated to construct nomogram, calibration curve and DCA according to Iasonos’ suggestion [52]. All statistical analyses were performed with R software 3.5.3. p < 0.05 was set at probability values of statistical significance.

## Results

### Identification of candidate snoRNAs

**Fig. 1:**
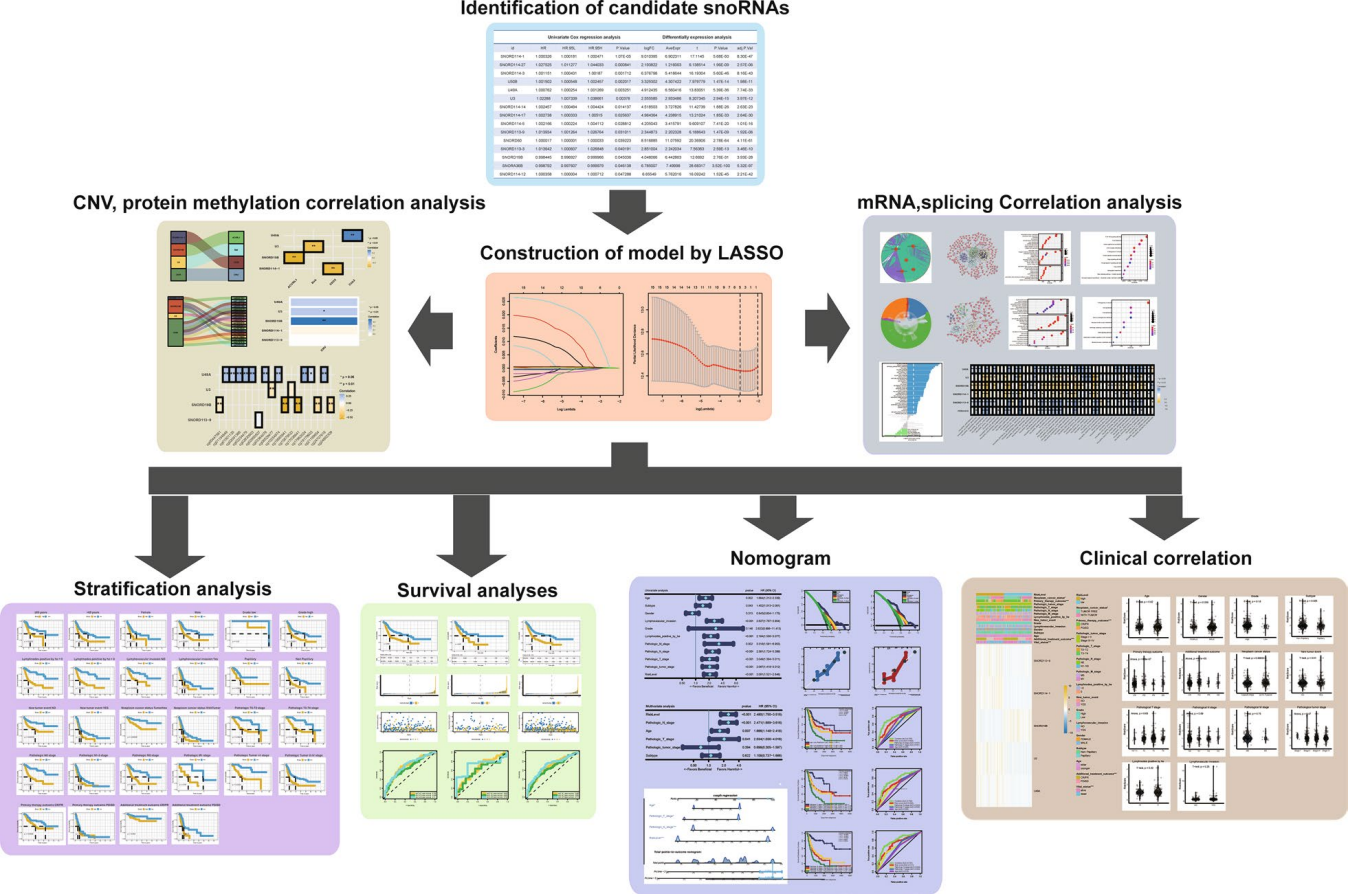
A study design and workflow

**Fig. 2:**
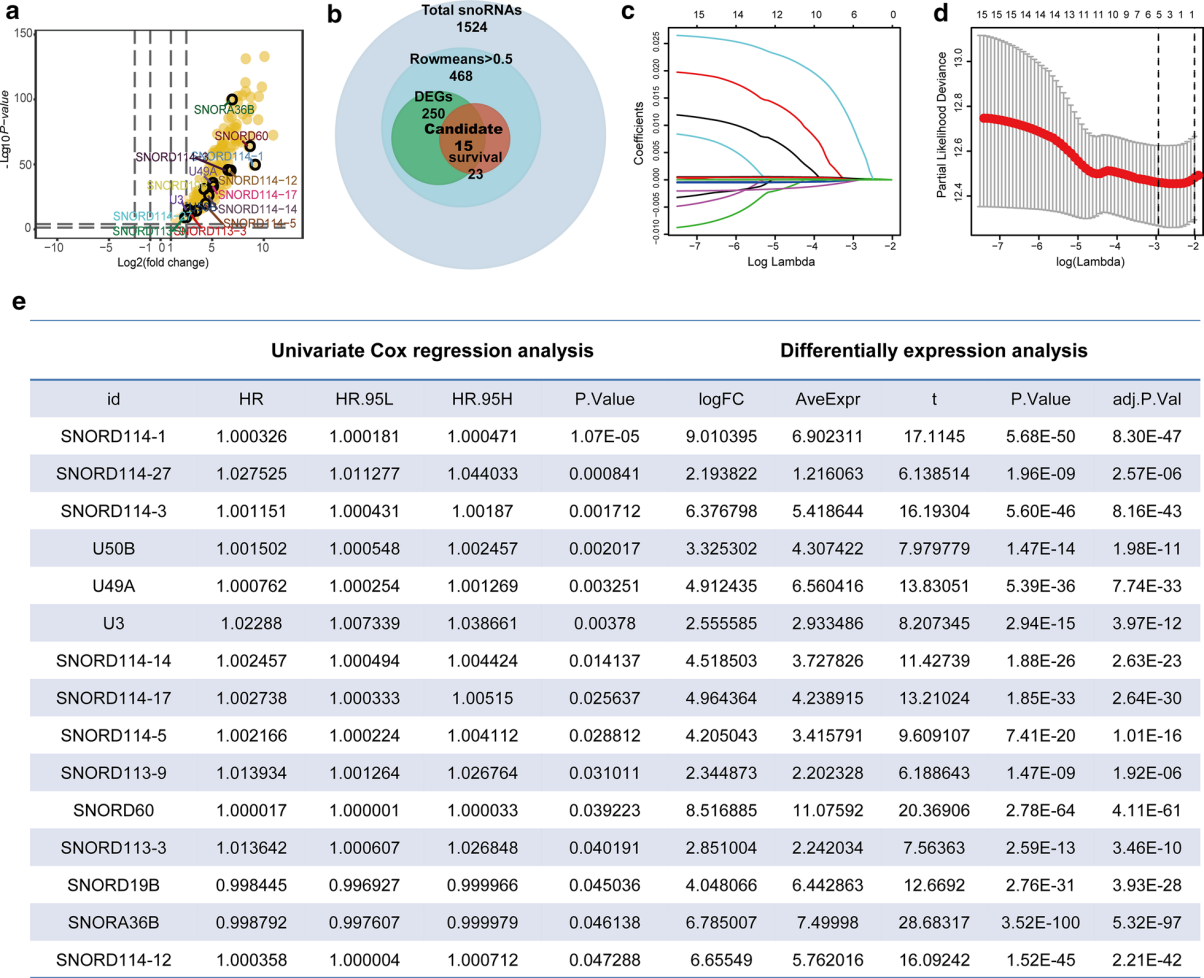
Identification of candidate snoRNAs. **a** Volcano plot demonstrated the differential expressed snoRNAs (DESs). The cut-off for DESs was logFC ≥ 1 and adj.P.Val < 0.05. Upregulated DESs (yellow); downregulated DESs (blue); 15 prognostic DESs (encircled). **b** Venn plot for screening of candidate snoRNAs. **c** LASSO coefficients profiles of 15 candidate snoRNAs. **d** Tuning parameter (λ) selection cross‐validation error curve. The vertical lines were drawn at the optimal values by the minimum criteria and the 1‐SE criteria. SNORS was chose at the left line by 1‐SE criteria. **e** Summary table for differential expression analyses and univariate Cox regression analyses for 15 prognosis DESs

A study design and workflow were shown in Fig. 1. In total, 1524 snoRNAs and 414 samples were obtained from SNORic database. Then low expression snoRNAs with average RPKM ≤ 0.5 and samples without complete survival information were removed from further study. Finally, we have enrolled 392 BLCA samples, 16 adjacent non-cancerous samples and 468 detectable snoRNAs as an entire TCGA-BLCA cohort (Fig. 2b). The detailed clinical information of entire TCGA-BLCA cohort could be found in Additional file 1: Table S1. Furthermore, we have got 250 differential expressed snoRNAs (DESs) when compared BLCA samples with adjacent non-cancerous samples tissues through package “*limma*”. Surprisingly, DESs were almost all upregulated in BLCA samples (Fig. 2a and Additional file 2: Table S2), which is consistent with previous study. Moreover, we have obtained 23 survival-related snoRNAs via univariate cox regression analyses (Additional file 3: Table S3). After merging DESs and survival-related snoRNAs, 15 prognostic candidate DESs were prepared for further research (Fig. 2b, e).

### Construction of SnoRNAs Signature (SNORS)

The entire TCGA-BLCA cohort was divided into training and testing sets at the cut-off 7:3. Then 15 prognostic DESs were submitted to LASSO and multivariate cox regression analysis, which were good at dimension reduction, to construct SNORS consisting of 5 candidate snoRNAs (Fig. 2c, d). KM survival curves demonstrated that SNORD113-9, U3, U49A, and SNORD114-1 were harmful factors, while SNORD19B was beneficial factor to patients with BLCA (Additional file 4: Figure. S1 and Additional file 5: Table S4). The formula for SNORS risk-score was calculated as follows: expression of SNORD113-9 * (0.01794) + expression of U3 * (0.02659) + expression of U49A * (0.00104) + expression of SNORD114-1 * (0.0002) + expression of SNORD19B * (− 0.0031).

### SNORS is highly associated with prognosis in BLCA

**Fig. 3:**
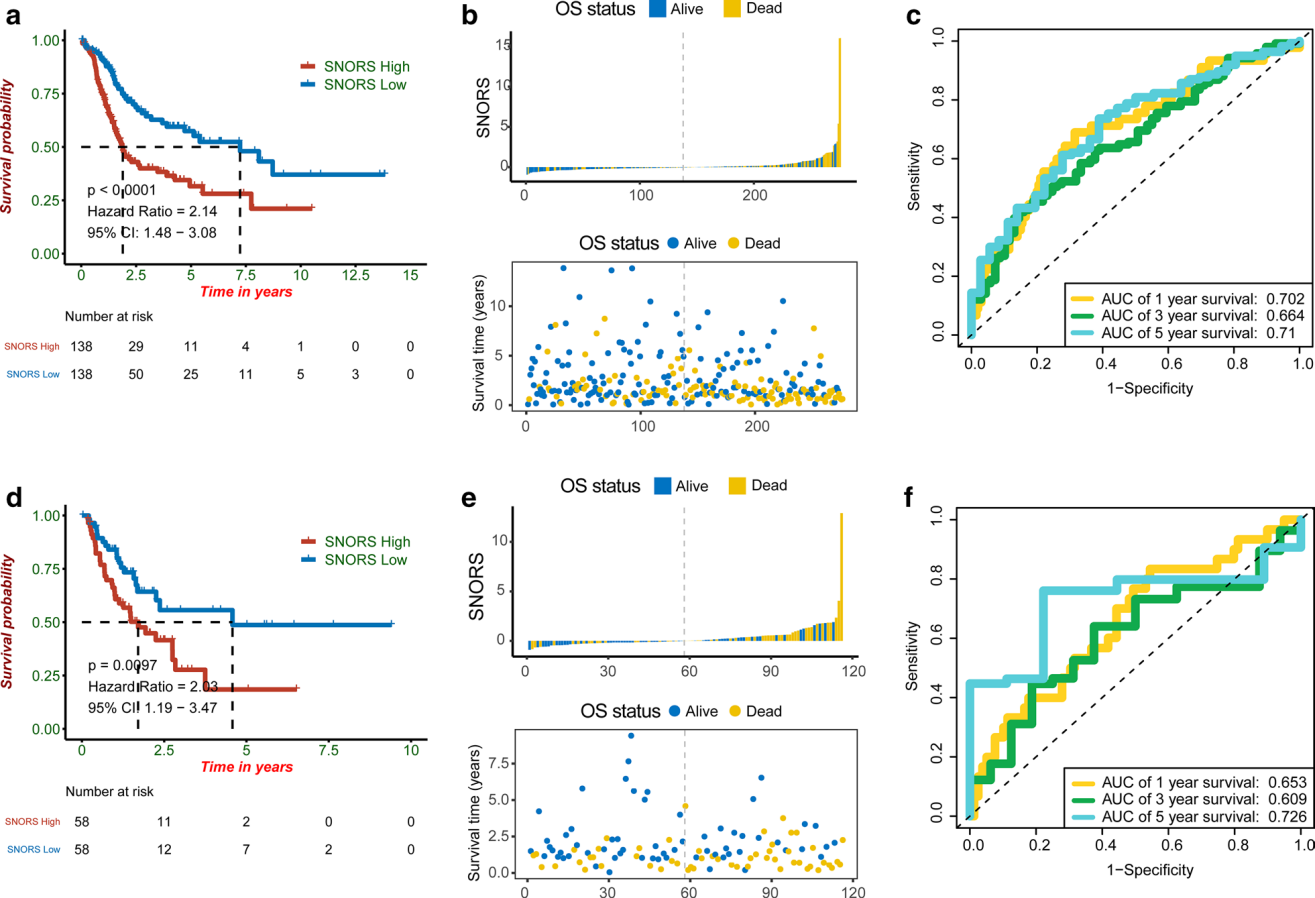
SNORS is a prognostic biomarker for overall survival (OS) in TCGA-BLCA cohort. **a**–**c** KM survival, risk score and time-dependent ROC curves of OS based on SNORS groups in training cohort. **d**–**f** KM survival, risk score and time-dependent ROC curves of OS based on SNORS groups in testing cohort. The high SNORS and low SNORS groups were stratified at median cut-off. The AUC was assessed at 1, 3 and 5 years

The patients within the training and testing sets were equally divided into high or low SNORS groups at median cut-off. KM survival analysis suggested that low SNORS group have a better OS than high SNORS group in both cohorts (p < 0.001) (Fig. 3a, d). As the SNORS risk-score increased, there were more death patients (Fig. 3b, e). And the survival time of patients with high SNORS were much shorter compared with patients with low SNORS (Fig. 3b, e). Moreover, time-dependent ROC curves demonstrated that SNORS displayed a high accuracy for predicting OS in both cohorts. AUC was 0.702 at 1 year, 0.664 at 3 years and 0.71 at 5 years in training cohort (Fig. 3c), 0.653 at 1 year, 0.609 at 3 years and 0.726 at 5 years in testing cohort (Fig. 3f). Furthermore, we also validated SNORS in prediction of DSS, PFI and RFS and found that all low SNORS groups were associated with better DSS, PFI and RFS than high SNORS patients (p < 0.01) (Additional file 6: Figure S2a, d, g). Thus, the death patients also have a higher SNORS risk-score and shorter survival time, which were all consistence with OS prediction (Additional file 6: Figure S2b, e, h). The AUC with 1-, 3- and 5-years were 0.678, 0.646, 0.695 in prediction DSS (Additional file 6: Figure S2c), 0.656, 0.632, 0.672 in in prediction PFI (Additional file 6: Figure S2f), and 0.61, 0.56, 0.531 in in prediction RFS (Additional file 6: Figure S2i).

### Integration analysis of candidate snoRNAs and SNORS

**Fig. 4:**
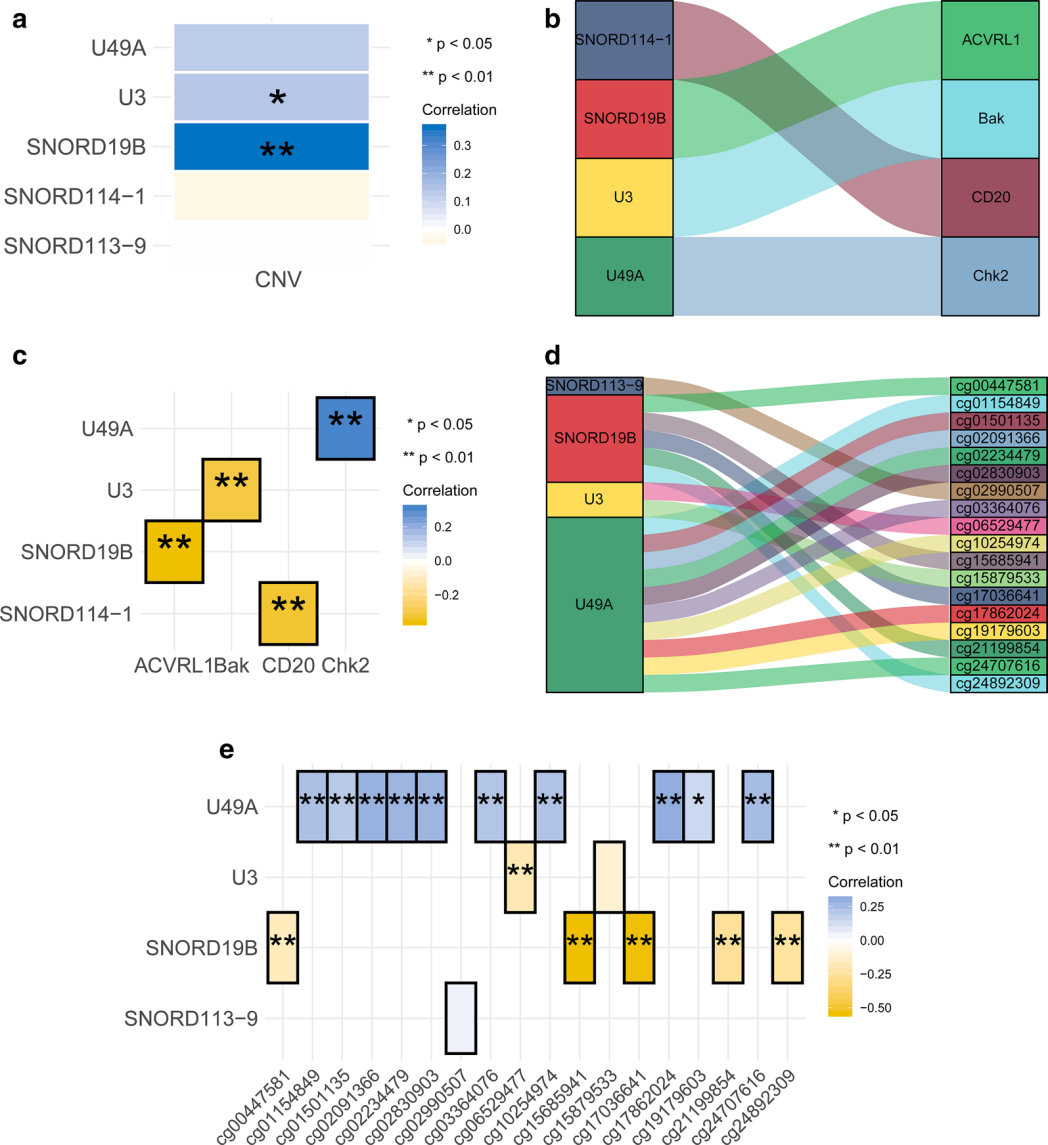
Integration analysis of candidate snoRNAs. **a** Correlation matrix showed the spearman correlation between candidate snoRNAs and their own CNV. **b** Sankey diagram showed the interaction of candidate snoRNAs and proteins. **c** Correlation matrix showed the spearman correlation between candidate snoRNAs and proteins. **d** Sankey diagram showed the interaction of candidate snoRNAs and methylation sites. **e** Correlation matrix showed the spearman correlation between candidate snoRNAs and methylation sites. The blue represented positive correlation and yellow indicated negative correlation. Shading colour and asterisks indicated the correlation coefficients. * p < 0.05, ** p < 0.01

As CNV and DNA methylation were reported to be involved in regulating snoRNAs expression in various cancers [44, 53], the correlation between them was measured. The results showed that expression of SNORD19B and U3 were positively correlated with their CNVs (Fig. 4a and Additional file 7: Table S5). In addition, we found U49A was strikingly positive correlated with their methylation probes, while U3 and SNORD19B had significantly negative correlation with their methylation probes (Fig. 4d, e and Additional file 8: Table S6). All of these indicated that expression of snoRNAs could be regulated by their CNV and methylation in BLCA.

**Fig. 5:**
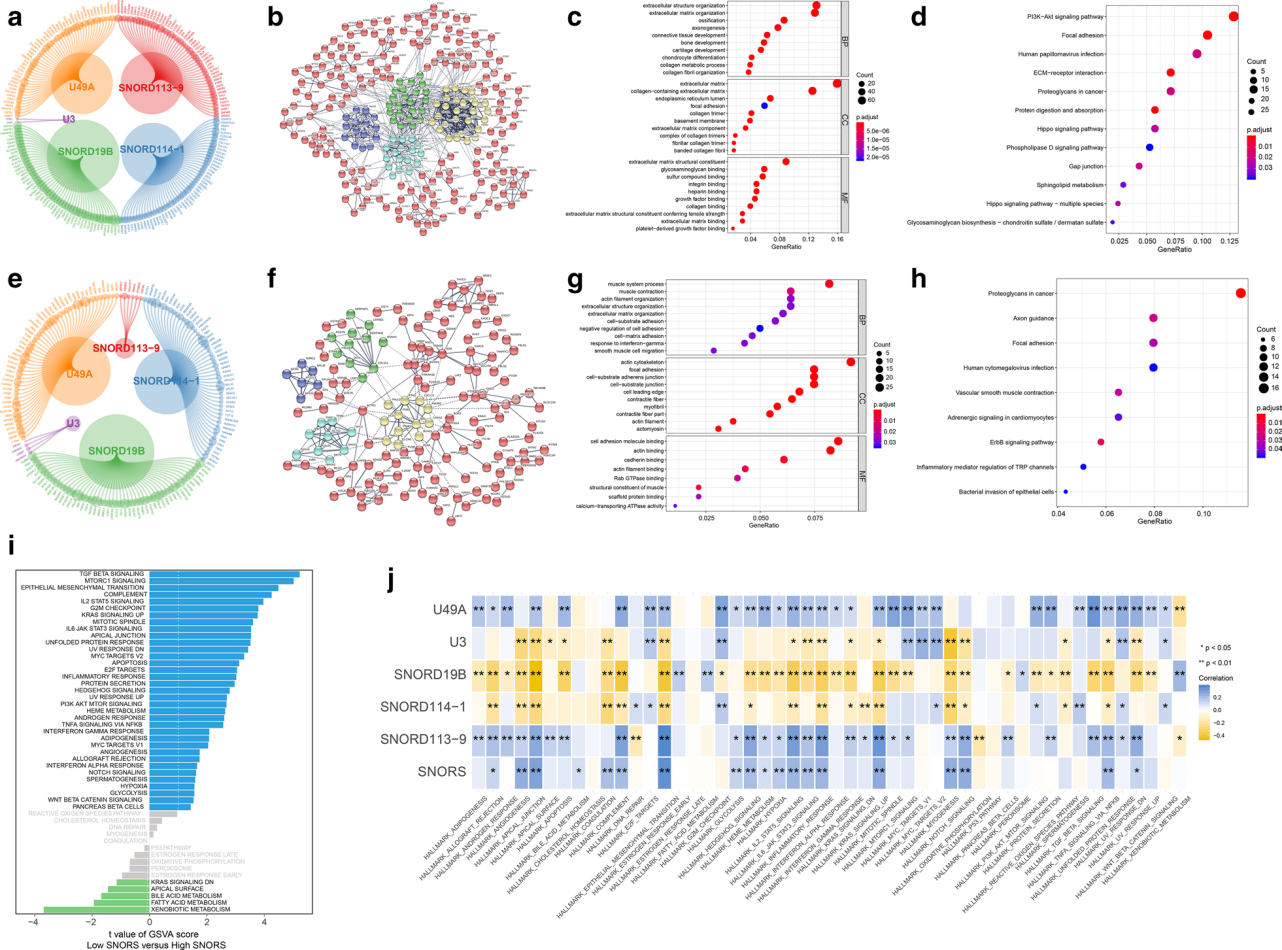
Function annotation and enrichment analysis. **a** Interaction network of 5 candidate snoRNAs and their survival associated mRNAs. The bubble size indicated the –log(pvalue) of univariate cox regression analyses for each mRNA. The top 50 associated mRNAs of each snoRNA were shown. **b** PPI network of candidate snoRNAs associated prognostic mRNAs. **c**, **d** GO and KEGG enrichment analysis of candidate snoRNAs prognostic mRNAs. The bubble size indicated the –log(pvalue) of each GO/KEGG term. **e** Interaction network of 5 candidate snoRNAs and their associated AS mRNAs. The bubble size indicated the –log(pvalue) of their correlation coefficient. The top50 associated mRNAs of each snoRNA were shown. **f** PPI network of candidate snoRNAs associated AS mRNAs. **g**, **h** GO and KEGG enrichment analysis of candidate snoRNAs associated AS mRNAs. The bubble size indicated the –log(pvalue) of each GO/KEGG term. **i** The bar plot showed the results of GSVA based on SNORS. The significantly enriched Hallmark gene sets were determined as p value < 0.05 and t value > 1. **j** Correlation matrix of SNORS, candidate snoRNAs and the relative levels of Hallmark gene sets. The blue indicated positive correlated and yellow indicated negative correlated. The asterisks represented the statistical p value (*p < 0.05; **p < 0.01)

Previous reports suggested that snoRNAs and their associated proteins, mRNAs and AS could collaborate to trigger cancer progression by influencing transcriptional and translational process. Then the correlations between them were measured. The results revealed that SNORD19B, U3, and SNORD114-1 were negatively correlated with ACVRL1, Bak, and CD20, respectively, while U49A were positively correlated with Chk2 (Fig. 4b, c and Additional file 9: Table S7). Furthermore, we found that 5 candidate snoRNAs were highly correlated with 2204 mRNAs (Additional file 10: Table S8). Among them, 361 mRNAs were correlated with two, 160 mRNAs with three, and 60 mRNAs with four candidate snoRNAs. Moreover, the results from the univariate cox regression analysis suggested that 525 associated mRNAs influence the prognosis of BLCA patients (Fig. 5; Additional file 11: Table S9). Then we found they could successfully formed clusters through PPI analysis by STRING tool (Fig. 5b and Additional file 12: Table S10). Furthermore, GO enrichment analysis showed candidate snoRNAs associated mRNAs were mainly enriched in “extracellular matrix”, “focal adhesion” etc. signalling pathways (Fig. 5c and Additional file 13: Table S11). The KEGG enrichment analysis also demonstrated that they were mainly enriched in “PI3K-Akt signalling pathway”, “focal adhesion”, “ECM-receptor interaction”, “proteoglycans in cancer”, etc. signalling pathways (Fig. 5d and Additional file 14: Table S12). Moreover, we detected the correlation between 5 candidate snoRNAs and AS. The results revealed that there were 318 snoRNAs associated AS mRNAs, and 102 AS mRNAs were included in above associated mRNAs (Fig. 5e and Additional file 15: Table S13). Then the PPI network showed that associated AS mRNAs also clustered well (Fig. 5f and Additional file 16: Table S14). Thus, GO enrichment analysis showed they were still mainly enriched in “extracellular matrix”, “focal adhesion”, etc. (Figure 5g and Additional file 17: Table S15). The KEGG enrichment analysis demonstrated they were mainly enriched in “proteoglycans in cancer”, “focal adhesion”, “ErbB signalling pathway”, etc. signalling pathways (Fig. 5h and Additional file 18: Table S16). Moreover, we next performed GSVA to figure out dynamics of biological processes and pathways for Hallmark gene sets based on SNORS. The results showed that “TGF-β signalling”, “MTORC1 signalling”, “epithelial–mesenchymal transition (EMT)”, and “KRAS signalling up”, etc. signalling pathways, were remarkably activated in high SNORS group, while low SNORS group were enriched in “KRAS signalling down” signalling pathways (Fig. 5i). Moreover, we also found that all above pathways associated with malignancy were strikingly positive correlated the SNORS risk-score (Fig. 5j and Additional file 19: Table S17).

### The correlation between SNORS with clinicopathological characteristics and molecular subtypes in BLCA

**Fig. 6:**
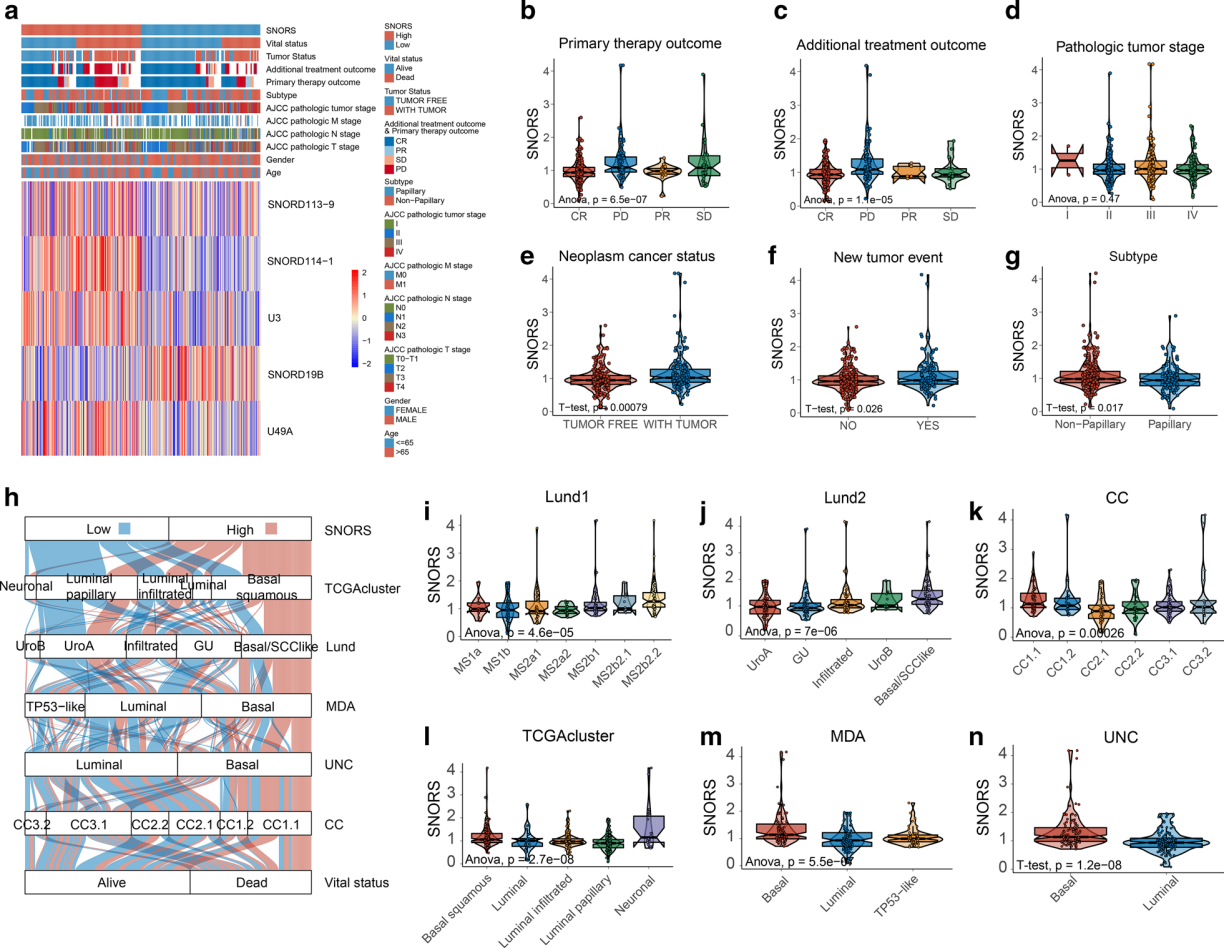
Association between the SNORS with clinicopathological characteristics and molecular subtypes. **a** Cluster heat map showed the relative levels of candidate snoRNAs which were stratified by SNORS in the TCGA-BLCA cohort. Yellow means upregulation while blue means downregulation. **b**–**g** Differences in SNORS between different clinicopathological characteristics and clinical outcome in TCGA-BLCA cohort. The upper and lower ends of the boxes represented interquartile range of values. The lines in the boxes represented median value. Student t tests or one-way Anova tests were used to compare the statistical difference between primary therapy outcome (**b**), additional treatment outcome (**c**), pathological tumour stage (**d**), neoplasm cancer statue (**e**), new tumour event (**f**), and histology subtype (**g**). **h** Alluvial diagram showing the dynamic changes of SNORS, indicated molecular subtypes and vital status in TCGA-BLCA cohort. **i**–**n** Differences in SNORS between different molecular subtypes in TCGA-BLCA cohort. The upper and lower ends of the boxes represented interquartile range of values. The lines in the boxes represented median value. Student t tests and one-way Anova tests were used to compare the statistical difference between Lund1 (**i**), Lund2 (**j**), CC (**k**), TCGAcluster (**l**), MDA (**m**), and UNC (**n**) molecular classification systems

KM survival analysis suggested that all clinical parameters were associated with prognosis of BLCA patients except for gender (Additional file 20: Figure S3). Considering positively associated with biological process in tumorigenesis, the correlation between SNORS with corresponding clinicopathological characteristics and clinical outcome were measured. Surprisingly, we found that SNORS was not correlated with clinicopathological characteristics such as pathological T/N/M stage, grade, lymphonodes metastasis, etc. (Figure 6a, d and Additional file 21: Figure S4 a–h). But the results showed that patients with high SNORS were more likely to be neoplasm cancer status with-tumor, primary/additional therapy with progressive disease/persistent disease/stable disease (PD/SD), new tumor event occurrence and non-papillary subtype (Fig. 6a–c, e–g). Although the SNORS was not correlated with many clinicopathological characteristics, we next investigated whether SNORS could apply to different clinicopathological characteristics. The stratification survival analysis demonstrated that SNORS was independent from all above parameters and could efficiently predict the prognosis in almost all the subgroups (Additional file 22: Figure S5).

Recently, a comprehensive molecular landscape has been established for BLCA by TCGA and other independent groups. They classified BLCA into many different molecular subtypes, such as luminal, basal, genomically unstable (GU), etc. Then we detected the difference of SNORS among these molecular subtypes. We were amazed to find that low SNORS group was concentrated on the molecular subtypes of luminal, luminal papillary, CC1/CC3, uroA, and genomically unstable (GU), which all represented low malignancy and better prognosis. However, molecular subtypes with basal, basal squamous, TP53-like, CC2 and basal/SCClike, which were characterized by high malignancy and worse prognosis, significantly accumulated in high SNORS group (Fig. 6h–n). Those findings suggest the clinical utility of our SNORS in BLCA.

### The SNORS was an independent prognostic factor in BLCA

**Fig. 7:**
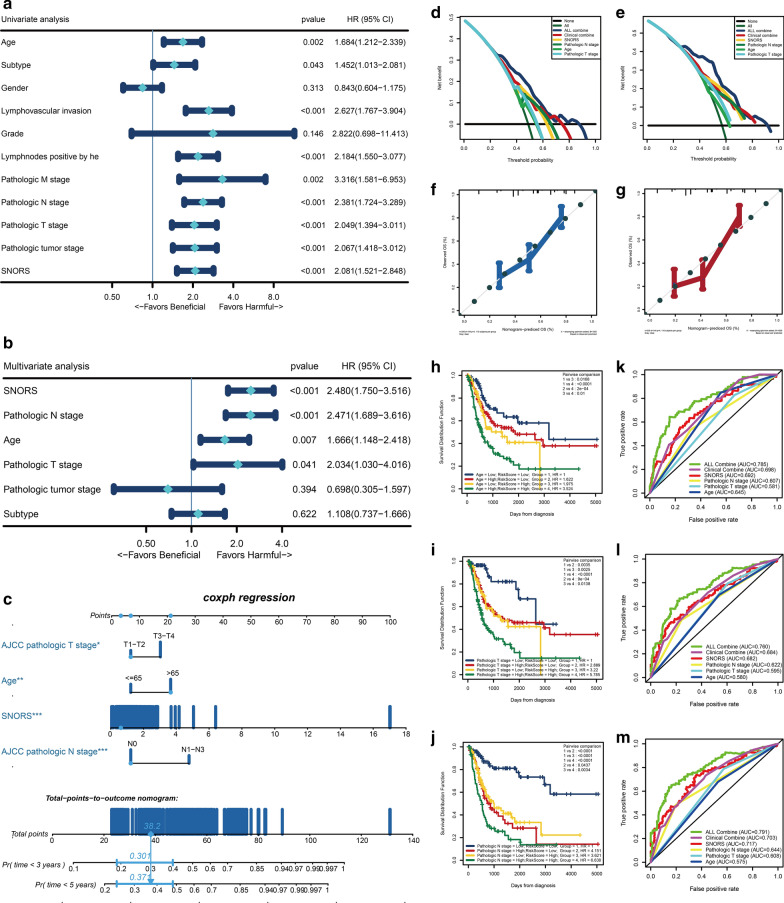
SNORS is an independent prognosis factor in BLCA. **a**, **b** Forest plot summary of the univariate and multivariable cox regression analysis measuring SNORS and clinicopathological characteristics. The p value, HR and 95% confidence interval (CI) were indicated in the figure. **c** Nomograms which integrated with independent prognosis factor for predicting the probability of patient mortality at 3- or 5-years OS. Blue circle represent the  point each parameter scored. **d**, **e** DCA curves for four independent prognostic factors or combination of them in OS prediction at 3-years (**d**) and 5-years (**e**). **f**, **g** Calibration curves of the nomogram for predicting the probability of OS at 3-years (**f**) and 5-years (**g**). **h**–**j** KM survival curves for patients stratified by both SNORS and age (**h**)/pathological T stage (**i**)/pathological N stage (**j**). The subgroup information and p value were display in detail. **k**–**m** ROC curves for four independent prognostic factors or combination of them in OS prediction at 1 years (k), 3 years (**l**) and 5 years (**m**) in TCGA-BLCA cohort. The abbreviation was as followed in Fig. 7 **d**–**e** and k-m: ALL combine: combination of SNORS, age, pathological T stage and pathological N stage; Clinical combine: combination of age, pathological T stage and pathological N stage

As SNORS was significantly correlated with clinical outcome and prognosis, next we aimed to find whether SNORS was an independent prognostic factor in BLCA. Univariate and multivariate cox regression analysis indicated that SNORS, pathological N stage, age and pathological T stage, which were four harmful independent prognostic factors for prognosis predicting in BLCA (Fig. 7a, b). Based on multivariate cox regression analysis, we then integrated four independent prognostic factors to construct a nomogram, which is a quantitative scoring method to predict survival probability for BLCA patients (Fig. 7c). The value range of each variable is determined for its contribution to the nomogram, which often referred to the regression coefficient. Then individual will get total points by plus single point of each variable within the nomogram. Finally, we can predict the clinical outcome or the probability of the individual clinical ending event through the function conversion of the total points. For example, in Fig. 7c, we found that this patient got total points of 38.2, referring to probability of 30.1% to death at 3 years and 37.1% to death at 5 years. The patients with high score would have a worse prognosis compared with patients with low score. Moreover, DCA curves suggested that SNORS was supreme beneficial when compared with all prognostic factors alone (Fig. 7d, e). Even though three clinicopathological characteristics were combined, the net benefit of SNORS was also comparable to the clinical combination model. Moreover, SNORS could strikingly promote the net benefit of clinical combination model. The calibration curves indicated that our nomogram displayed similar prediction accuracy as the ideal model (Fig. 7f, g). Then we subsequent combined SNORS with other three prognosis factors to stratify all the samples to four different groups. The results showed that patients with low SNORS/young/low pathological T stage/low pathological N stage had a longer survival, while prognosis of patients with high SNORS/elder/high pathological T stage/high pathological N stage was worse among all groups (Fig. 7h, j and l). Furthermore, ROC curves revealed that the AUC of the SNORS reached higher AUCs than the three clinicopathological characteristics alone and approximately well matched with clinical combination model. And SNORS could also remarkably improve the AUC value of clinical combination model in established nomogram (Fig. 7i, k, m and Additional file 23: Table S18). All of these suggested that our established nomogram could be of a high potential for clinical utility.

## Discussion

BLCA is a malignancy with high incidence and recurrence. Although great progress has been  achieved for recent emerging neoadjuvant chemotherapy and immunotherapy, indolent and aggressive tumours could not be distinguished just based on traditional system, such as TNM staging and tumour grade, etc., which mainly represented anatomical distribution without biological features. Even though FISH, NMP22, etc. have been approved by FDA to participate in diagnosis of BLCA, its clinical prospective utility still have a long way to validate. With the rapid development of next generation sequencing and bioinformatics, ncRNAs, which are thought to be “junk” at first, are found to play a critical roles in tumorigenesis.

Recently, snoRNAs, which are a new small ncRNAs, have attracted researchers’ attention and been identified to be involved in many important biological processes. Some study even found that dysfunction of snoRNAs may induce oncogenesis and could serve as biomarkers in cancers [32]. Thus snoRNAs was reported to exert its effect on tumorigenesis in various regulatory ways [54]. First, several snoRNAs have been found to display a specific expression pattern, directly participate in tumorigenesis in different cancer. Second, snoRNAs might regulate other genes expression or function through post-transcriptional and translational way, which indirectly induced tumorigenesis [55]. Therefore, snoRNAs were found to act as oncogenes or tumour suppressors in various biological processes, such as cell proliferation, invasion, apoptosis and metastasis. Siprashvili et al. found SNORD50A and SNORD50B were usually deleted in human cancer. Loss of SNORD50A and SNORD50B could increase the binding of K-RAS and hyper-activation of RAS-ERK1/ERK2 signalling to induce tumorigenesis [47]. A lot of snoRNAs, including SNORD78, SNORD71A and SNORD42 etc., were reported to be associated with non-small cell lung cancer (NSCLC) proliferation, migration and invasion. Yan et al. even established a six snoRNA signature to act as a non-invasive biomarker for diagnosis and prognosis prediction of renal clear cell carcinoma (ccRCC). But little was reported to elaborate the function of snoRNAs in BLCA. In present study, we aimed to study the role of snoRNAs in BLCA, which to our knowledge, is the first study focusing on snoRNAs in BLCA. We first selected DESs compared tumour samples with adjacent non-cancerous samples. What surprised us was that almost all DESs were upregulated in tumour samples, which was consistent with results from previous study. After submitting prognostic snoRNAs to LASSO cox regression analysis, we have screened out 5 candidate snoRNAs, which were all C/D box snoRNA, and constructed SNORS as the diagnosis biomarker depending on TCGA dataset.

SnoRNAs might exert similar function of other ncRNAs to regulate a lot of RNA transcripts, and at the same time be regulated by others. Therefore, aberrant expression of snoRNAs could be a disaster to the tightly controlled RNA networks, which was reported to initiate and induce oncogenesis. As 5 candidate snoRNAs were significantly differential expressed in BLCA samples, we next wanted to figure out whether CNV and DNA methylation was a key approach to regulate their expression. Then we found U3, U49A and SNORD19B, which made a larger contribution to SNORS, were significantly correlated with their own CNV and specific methylation sites. So we inferred that upregulation of snoRNAs in BLCA might be a cause of CNV and methylation, the specific mechanism should be investigated in the future. Then the correlation between snoRNAs with proteins, mRNAs and AS was measured. We found that SNORD19B, U3, SNORD114-1and U49A had a correlation with ACVRL1, Bak, CD20 and Chk2 respectively. These proteins took part in the process of apoptosis and cell cycle, which was very important in tumorigenesis. Moreover, the results showed that 5 candidate snoRNAs were highly correlated with 2204 mRNAs. Among them 581 mRNAs have a correlation with more than 2 candidate snoRNAs, and 525 associated mRNAs were associated with the prognosis of BLCA patients. In addition, we also found that 5 candidate snoRNAs were significantly correlated with AS. The PPI network demonstrated that candidate snoRNAs associated mRNAs and AS mRNAs could successfully form clusters. All of these indicated that 5 candidate snoRNAs within the signature were significantly mutually correlated and could regulate same important targets.

Extracellular matrix (ECM) is a highly dynamic structure which ubiquitously exists in all kinds of tissues and subsequently undergoes remodelling control. Remodelling of ECM is also reported to influence diverse biological process, including proliferation, migration and differentiation [56]. Dysregulation of ECM composition, structure and abundance leads to several pathological diseases, such as fibrosis and cancer. ECM is major components consisting of tumour microenvironment (TME), which could collaborate with other constituents, such as immune and stromal cells, to act as initiator and inducer by building a chronic inflammatory and pro-angiogenic intratumoural atmosphere. Moreover, ECM was also found to correlate with cancer patients’ outcomes and treatment efficacy [57]. Thus, GO and KEGG enrichment analysis from candidate snoRNAs related mRNA and AS all enriched in “extracellular matrix”, “focal adhesion”, and “proteoglycans in cancer”, etc. signalling pathways. These indicated 5 snoRNAs might be involved in regulating extracellular matrix formation and remodelling to induce BLCA occurrence. Then we investigated the function annotation of low and high risk groups according to 5 candidate snoRNAs constructed SNORS. Amazingly, the GSVA suggested that high SNORS groups were enriched in signalling pathways associated with “TGF-β signalling”, “MTORC1 signalling”, “epithelial-mesenchymal transition (EMT)”, and “KRAS signalling up”, etc., which were representative of ECM modification and malignancy of BLCA we have reported previously [58]. But low SNORS group displayed the opposite situation. Although highly related with the malignancy of BLCA, we unfortunately found that SNORS exhibited no correlation with TNM stage and grade, etc., meanwhile, we can see that SNORS was strikingly correlated with clinical outcomes, such as neoplasm cancer status, primary/additional therapy outcome and new tumour event, which were closely related to patients’ prognosis. Moreover, the patients with higher SNORS were more likely to be molecular subtypes related with EMT/TGF-β signalling pathway, worse survival, and high malignancy including basal, basal squamous, TP53-like, CC1/CC3, and basal/SCClike, while patients with molecular subtypes of luminal, luminal papillary, CC2, uroA, and genomically unstable (GU), which characterized by low malignancy and better prognosis exhibited lower SNORS. All of these demonstrated that SNORS could predict the tumour malignancy in spite of clinical parameters.

In addition, KM survival curves demonstrated SNORS was harmful risk factor for prognosis, and high SNORS patients had a worse OS, DSS, PFI and RFS. Although as a heterogeneous disease, stratification analysis showed that SNORS was independent from all of them and could predict well in all subgroups. Moreover, we found that SNORS was an independent prognostic factor in BLCA even combining with other variables. And the diagnosis accuracy of SNORS was much better than conventional clinical pathological characteristics alone and comparable to the combination of three clinical independent prognostic factors. Besides, we further integrated the SNORS with pathological N stage, pathological T stage, and age to construct a nomogram. We also found SNORS could cooperate with clinical pathological characteristics to exert higher diagnosis accuracy compared with variables alone and might have a potential value for clinical apply. All of these indicated that SNORS play an oncogenic role and was potent biomarker which could predict prognosis in BLCA.

Our study has some limitations as our analysis were retrospective and the efficiency of the SNORS should be further validated in prospective studies. Moreover, we should incorporate more independent variables with SNORS scoring system to improve the prediction accuracy, as not all patients with a high SNORS displayed a worse prognosis.

## Conclusion

We performed comprehensive analysis of snoRNAs and established a prognostic and predictive SNORS for BLCA, which could open our view in snoRNA and may provide a useful scoring system for clinical utility.

## Supplementary information

**Additional file 1: Table S1.** Summary of detailed clinical information of TCGA-BLCA cohort.

**Additional file 2: Table S2.** Summary of differentially expressed snoRNAs (DESs) in TCGA-BLCA cohort.

**Additional file 3: Table S3.** Summary of univariate Cox regression analysis for snoRNAs in TCGA-BLCA cohort.

**Additional file 4: Figure S1.** KM survival analyses of 5 candidate snoRNAs in TCGA-BLCA cohort. (a) SNORD113-9; (b) U3; (c) U49A; (d)SNORD114-1; (e) SNORD19B; (f) ROC curves of 5candidate snoRNAs and SNORS.

**Additional file 5: Table S4.** Comparison of AUC values between SNORS and 5 candidate snoRNAs in TCGA-BLCA cohort.

**Additional file 6: Figure S2.** SNORS is a correlated with DSS, DFI and RFS in TCGA-BLCA cohort. (a-c) KM survival, risk score and time-dependent ROC curves of DFS according to SNORS groups in TCGA-BLCA cohort. (d-f) KM survival, risk score and time-dependent ROC curves of PFI according to SNORS groups in TCGA-BLCA cohort. (g-i) KM survival, risk score and time-dependent ROC curves of RFS according to SNORS groups in TCGA-BLCA cohort. The high SNORS and low SNORS groups were stratified at median cut-off. The AUC was assessed at 1, 3 and 5 years.

**Additional file 7: Table S5.** Correlation between candidate snoRNAs and their copy number variations (CNV) in TCGA-BLCA cohort.

**Additional file 8: Table S6.** Correlation between candidate snoRNAs and their methylation sites in TCGA-BLCA cohort.

**Additional file 9: Table S7.** Correlation between candidate snoRNAs and relevant proteins in TCGA-BLCA cohort.

**Additional file 10: Table S8.** Correlation between candidate snoRNAs and mRNAs in TCGA-BLCA cohort.

**Additional file 11: Table S9.** The input for correlation network of candidate snoRNAs associated mRNAs in TCGA-BLCA cohort.

**Additional file 12: Table S10.** The output of PPI network for candidate snoRNAs relevant mRNAs in TCGA-BLCA cohort.

**Additional file 13: Table S11.** Summary of GO analyses for candidate snoRNAs relevant mRNAs in TCGA-BLCA cohort PPI network of candidate snoRNAs relevant AS mRNAs.

**Additional file 14: Table S12.** Summary of KEGG analyses for candidate snoRNAs relevant mRNAs in TCGA-BLCA cohort.

**Additional file 15: Table S13.** Correlation between candidate snoRNAs and alternative splicing variants (AS) mRNA in TCGA-BLCA cohort.

**Additional file 16: Table S14.** The output of PPI network for candidate snoRNAs relevant AS mRNAs in TCGA-BLCA cohortSpearman correlation analysis for candidate snoRNAs and relevant signature with Hallmark gene-set in TCGA-BLCA cohorts.

**Additional file 17: Table S15.** Summary of GO analysis for candidate snoRNAs relevant AS mRNAs in TCGA-BLCA cohort .

**Additional file 18: Table S16.** Summary of KEGG analysis for candidate snoRNAs relevant AS mRNAs in TCGA-BLCA cohort.

**Additional file 19: Table S17.** Spearman correlation analyses for candidate snoRNAs and relevant signature with Hallmark gene-sets in TCGA-BLCA cohort.

**Additional file 20: Figure S3.** KM survival analyses of different clinicopathological characteristics in TCGA-BLCA cohort. (a) Age. (b) Gender. (c) Grade. (d) lymphnodes positive by HE. (e) Lymphovascular invasion. (f) Subtype. (g) Pathology T stage. (h) Pathology N stage. (i) Pathology M stage. (j) Pathology tumor stage. (k) Neoplasm cancer status. (l) New tumor event. (m) Primary therapy outcome. (n) Additional treatment outcome.

**Additional file 21: Figure S4.** Differences in SNORS between different clinicopathological characteristics in TCGA-BLCA cohort. The upper and lower ends of the boxes represented interquartile range of values. The lines in the boxes represented median value. Student t tests and one-way Anova tests were used to compare the statistical difference between age (a), gender (b), lymphonodes positive by he (c), lymphovascular invasion (d), grade (e), pathological T stage (f), pathological N stage (h), and pathological M stage (i).

**Additional file 22: Figure S5.** KM stratification survival analyses in TCGA-BLCA cohort. (a) Age≤65 years. (b) Age >65 years. (c) Female. (d) Male. (e) Grade low. (f) Grade high. (g) lymphnodes positive HE=0. (h) lymphnodes positive by HE>0. (i) Lymphovascular invasion NO. (j) Lymphovascular invasion YES. (k) Subtype papillary and non- papillary. (l) Subtype non-papillary. (m) New tumour event NO. (n) New tumour event YES. (o) Tumour-free. (p) With-tumour. (q) Pathology T0‐T2 stage. (r) Pathology T3‐T4 stage. (s) Pathology N0 stage. (t) Pathology N1-3 stage. (u) Pathology M0 stage. (v) Pathology M1 stage. (w) Pathology tumour I-II stage. (x) Pathology tumour III-IV stage. (y) Primary therapy outcome CR/PR. (z) Primary therapy outcome PD/SD. (aa) Additional treatment outcome CR/PR. (bb) Additional treatment outcome PD/SD.

**Additional file 23: Table S18**. Comparison of AUC values in Fig. 7.

## Data Availability

All data generated or analysed during this study are included in this published article and its Additional files 1–23 information files.

## Abbreviations

AS: Alternative splicing
AUC: Area under curve
BCG: Bacillus Calmette–Guerin
BLCA: Bladder cancer
ccRCC: Renal clear cell carcinoma
CI: Confidence interval
CNV: Copy number variation
circRNA: Circular RNA
DCA: Decision curve analysis
DES: differentially expressed snoRNA
DSS: Disease free survival
ECM: Extracellular matrix
EMT: Epithelial–mesenchymal transition
FDA: Food and Drug Administration
FPKM: Fragments Per Kilobase per Million
GO: Gene Ontology
GSVA: Gene Set Variation Analysis
HE: Hematoxylin and eosin
HR: Hazard ratio
KEGG: Kyoto Encyclopedia of Genes and Genomes
KM: Kaplan–Meier
LASSO: Least absolute shrinkage and selection operator
Limma: Linear Models for Microarray Data
lncRNA: Long non-coding RNA
MIBC: Muscle-invasive bladder cancer
miRNAs: microRNA
ncRNA: Non-coding RNAs
NMIBC: Non-muscle-invasive bladder cancer
NSCLC: Non-small cell lung cancer
OS: Overall survival
PD/SD: Progressive disease/persistent disease/stable disease
PFI: Progression free interval
piRNA: Piwi-interacting RNA
PPI: Protein–protein interactions
RPKM: Reads per kilobase per million mapped reads
rRNAs: Ribosomes
RNPs: Ribonucleoproteins
ROC: Receiver operating characteristic curve
RFS: Recurrence free survival
scaRNAs: Cajal body-specific RNAs
SNORS: Small nucleolar RNAs signature
snRNAs: Small nuclear RNAs
ssGSEA: Single-sample gene set enrichment analysis
TCGA: The Cancer Genome Atlas
TME: Tumour microenvironment
TPM: Transcripts per million reads
tRNAs: Transfer RNAs
TNM: Tumor Node Metastasis
TURBT: Transurethral resection of bladder tumour
