# Supplementary material for: Small nucleolar RNAs signature (SNORS) identified clinical outcome and prognosis of bladder cancer (BLCA)
Source: Cancer Cell Int. 2020 Jul 10;20:299. doi: 10.1186/s12935-020-01393-7 (PMC7350589; doi:10.1186/s12935-020-01393-7)
Supplement: Supplementary file 1 — Additional file 1: Table S1. Summary of detailed clinical information of TCGA-BLCA cohort. [file 12935_2020_1393_MOESM1_ESM.docx]

**Additional file 1: Table S1 Summary of detailed clinical information of TCGA-BLCA cohort**

| **TCGA-BLCA** | **Low(n=196)** | **High(n=196)** | **Total(n=392)** |
| --- | --- | --- | --- |
| **Age** |  |  |  |
| <=65 | 77 (39.3%) | 79 (40.3%) | 156 (39.8%) |
| >65 | 119 (60.7%) | 117 (59.7%) | 236 (60.2%) |
| **Gender** |  |  |  |
| FEMALE | 42 (21.4%) | 60 (30.6%) | 102 (26.0%) |
| MALE | 154 (78.6%) | 136 (69.4%) | 290 (74.0%) |
| **Vital status***** |  |  |  |
| Alive | 132 (67.3%) | 90 (45.9%) | 222 (56.6%) |
| Dead | 64 (32.7%) | 106 (54.1%) | 170 (43.4%) |
| **Primary therapy outcome**** |  |  |  |
| CR | 132 (67.3%) | 90 (45.9%) | 222 (56.6%) |
| PR | 9 (4.6%) | 9 (4.6%) | 18 (4.6%) |
| SD | 13 (6.6%) | 17 (8.7%) | 30 (7.7%) |
| PD | 19 (9.7%) | 48 (24.5%) | 67 (17.1%) |
| **Additional treatment outcome**** |  |  |  |
| CR | 98 (50.0%) | 71 (36.2%) | 169 (43.1%) |
| PR | 3 (1.5%) | 2 (1.0%) | 5 (1.3%) |
| SD | 10 (5.1%) | 5 (2.6%) | 15 (3.8%) |
| PD | 30 (15.3%) | 66 (33.7%) | 96 (24.5%) |
| **Subtype** |  |  |  |
| Non-Papillary | 126 (64.3%) | 135 (68.9%) | 261 (66.6%) |
| Papillary | 69 (35.2%) | 57 (29.1%) | 126 (32.1%) |
| **Lymphnodes positive by he** |  |  |  |
| >0 | 61 (31.1%) | 54 (27.6%) | 115 (29.3%) |
| 0 | 81 (41.3%) | 87 (44.4%) | 168 (42.9%) |
| **Lymphovascular invasion** |  |  |  |
| NO | 71 (36.2%) | 53 (27.0%) | 124 (31.6%) |
| YES | 79 (40.3%) | 63 (32.1%) | 142 (36.2%) |
| **AJCC pathologic T stage** |  |  |  |
| T1 | 3 (1.5%) | 1 (0.5%) | 4 (1.0%) |
| T2 | 61 (31.1%) | 52 (26.5%) | 113 (28.8%) |
| T3 | 89 (45.4%) | 100 (51.0%) | 189 (48.2%) |
| T4 | 33 (16.8%) | 21 (10.7%) | 54 (13.8%) |
| **AJCC pathologic N stage** |  |  |  |
| N0 | 109 (55.6%) | 118 (60.2%) | 227 (57.9%) |
| N1 | 23 (11.7%) | 20 (10.2%) | 43 (11.0%) |
| N2 | 40 (20.4%) | 35 (17.9%) | 75 (19.1%) |
| N3 | 3 (1.5%) | 4 (2.0%) | 7 (1.8%) |
| **AJCC pathologic M stage** |  |  |  |
| M0 | 91 (46.4%) | 97 (49.5%) | 188 (48.0%) |
| M1 | 6 (3.1%) | 5 (2.6%) | 11 (2.8%) |
| **AJCC pathologic tumor stage** |  |  |  |
| I | 1 (0.5%) | 1 (0.5%) | 2 (0.5%) |
| II | 64 (32.7%) | 60 (30.6%) | 124 (31.6%) |
| III | 62 (31.6%) | 73 (37.2%) | 135 (34.4%) |
| IV | 68 (34.7%) | 61 (31.1%) | 129 (32.9%) |
| **Grade** |  |  |  |
| High Grade | 182 (92.9%) | 187 (95.4%) | 369 (94.1%) |
| Low Grade | 13 (6.6%) | 7 (3.6%) | 20 (5.1%) |
| **Tumor Status*** |  |  |  |
| TUMOR FREE | 113 (57.7%) | 82 (41.8%) | 195 (49.7%) |
| WITH TUMOR | 68 (34.7%) | 87 (44.4%) | 155 (39.5%) |
| **TCGA cluster**** |  |  |  |
| Basal squamous | 46 (23.5%) | 84 (42.9%) | 130 (33.2%) |
| Luminal | 12 (6.1%) | 14 (7.1%) | 26 (6.6%) |
| Luminal infiltrated | 39 (19.9%) | 37 (18.9%) | 76 (19.4%) |
| Luminal papillary | 90 (45.9%) | 48 (24.5%) | 138 (35.2%) |
| Neuronal | 6 (3.1%) | 12 (6.1%) | 18 (4.6%) |
| **Scientific Reports 2015 CC*** |  |  |  |
| CC1.1 | 10 (5.1%) | 41 (20.9%) | 51 (13.0%) |
| CC1.2 | 7 (3.6%) | 15 (7.7%) | 22 (5.6%) |
| CC2.1 | 27 (13.8%) | 14 (7.1%) | 41 (10.5%) |
| CC2.2 | 16 (8.2%) | 14 (7.1%) | 30 (7.7%) |
| CC3.1 | 28 (14.3%) | 40 (20.4%) | 68 (17.3%) |
| CC3.2 | 8 (4.1%) | 9 (4.6%) | 17 (4.3%) |
| **Scientific Reports 2015 UNC***** |  |  |  |
| Basal | 26 (13.3%) | 81 (41.3%) | 107 (27.3%) |
| Luminal | 70 (35.7%) | 52 (26.5%) | 122 (31.1%) |
| **Scientific Reports 2015 MDA***** |  |  |  |
| Basal | 20 (10.2%) | 68 (34.7%) | 88 (22.4%) |
| Luminal | 53 (27.0%) | 40 (20.4%) | 93 (23.7%) |
| TP53 like | 23 (11.7%) | 25 (12.8%) | 48 (12.2%) |
| **Lund1**** |  |  |  |
| MS1a | 6 (3.1%) | 7 (3.6%) | 13 (3.3%) |
| MS1b | 29 (14.8%) | 27 (13.8%) | 56 (14.3%) |
| MS2a1 | 21 (10.7%) | 12 (6.1%) | 33 (8.4%) |
| MS2a2 | 11 (5.6%) | 8 (4.1%) | 19 (4.8%) |
| MS2b1 | 16 (8.2%) | 24 (12.2%) | 40 (10.2%) |
| MS2b2.1 | 5 (2.6%) | 7 (3.6%) | 12 (3.1%) |
| MS2b2.2 | 8 (4.1%) | 48 (24.5%) | 56 (14.3%) |
| **Lund2***** |  |  |  |
| Basal/SCClike | 8 (4.1%) | 48 (24.5%) | 56 (14.3%) |
| Genomically unstable | 32 (16.3%) | 20 (10.2%) | 52 (13.3%) |
| Infiltrated | 16 (8.2%) | 24 (12.2%) | 40 (10.2%) |
| UroA | 35 (17.9%) | 34 (17.3%) | 69 (17.6%) |
| UroB | 5 (2.6%) | 7 (3.6%) | 12 (3.1%) |

* p < 0.05, ** p < 0.01, *** p < 0.001,
