# Supplementary material for: Small nucleolar RNAs signature (SNORS) identified clinical outcome and prognosis of bladder cancer (BLCA)
Source: Cancer Cell Int. 2020 Jul 10;20:299. doi: 10.1186/s12935-020-01393-7 (PMC7350589; doi:10.1186/s12935-020-01393-7)
Supplement: Supplementary file 2 — Additional file 2: Table S2. Summary of differentially expressed snoRNAs (DESs) in TCGA-BLCA cohort. [file 12935_2020_1393_MOESM2_ESM.docx]

**Additional file 2: Table S2 Summary of differentially expressed snoRNAs (DESs) in TCGA-BLCA cohort (n = 392)**

| id | logFC | AveExpr | t | P.Value | adj.P.Val |
| --- | --- | --- | --- | --- | --- |
| SNORD66_chr3 | 11.30299107 | 11.86629065 | 39.66845748 | 1.13E-142 | 1.73E-139 |
| SNORD104 | 11.73026579 | 12.88174612 | 38.10758995 | 4.97E-137 | 7.57E-134 |
| U31_chr11 | 10.06585925 | 11.30715439 | 37.22862678 | 8.52E-134 | 1.30E-130 |
| SNORD43 | 8.255193435 | 9.819186483 | 36.76460149 | 4.51E-132 | 6.86E-129 |
| SNORD38A | 8.223390184 | 9.853687157 | 32.0115094 | 9.77E-114 | 1.48E-110 |
| SNORD1B | 9.818434208 | 9.750415551 | 31.164223 | 2.43E-110 | 3.70E-107 |
| SNORD90 | 8.247879067 | 9.454303362 | 30.31538438 | 6.71E-107 | 1.02E-103 |
| SNORD85 | 7.909818762 | 9.433063764 | 29.56301688 | 8.08E-104 | 1.23E-100 |
| ACA45_chr15 | 7.45866315 | 9.42309879 | 29.41809797 | 3.19E-103 | 4.84E-100 |
| SNORD98 | 9.51390902 | 10.84064609 | 29.36735062 | 5.17E-103 | 7.83E-100 |
| U27 | 8.709767658 | 10.75447337 | 29.28899133 | 1.09E-102 | 1.65E-99 |
| U30 | 13.31223298 | 14.9846548 | 29.17326348 | 3.27E-102 | 4.95E-99 |
| SNORA36B | 6.785006629 | 7.499980003 | 28.6831712 | 3.52E-100 | 5.32E-97 |
| U3-4_chr17 | 7.48176665 | 9.44166392 | 28.31088269 | 1.25E-98 | 1.89E-95 |
| SNORD95 | 8.819680656 | 9.722654145 | 27.39345547 | 8.87E-95 | 1.34E-91 |
| SNORD3C | 7.031459505 | 8.890474999 | 27.2394164 | 3.96E-94 | 5.98E-91 |
| U25_chr11 | 8.591743403 | 10.28104796 | 26.60463031 | 1.95E-91 | 2.93E-88 |
| SNORD20 | 7.961423651 | 9.412562721 | 26.11918922 | 2.28E-89 | 3.44E-86 |
| SNORD119 | 6.885574691 | 8.737930838 | 25.62312562 | 3.04E-87 | 4.58E-84 |
| SNORD3B-2 | 6.57938153 | 8.420849084 | 25.39033893 | 3.05E-86 | 4.58E-83 |
| SNORD48 | 8.37892201 | 10.6462498 | 25.21702423 | 1.70E-85 | 2.55E-82 |
| SNORD99 | 9.453839594 | 10.34212415 | 25.13028967 | 4.02E-85 | 6.04E-82 |
| U57_chr20 | 7.371427888 | 9.768906564 | 24.33078226 | 1.15E-81 | 1.73E-78 |
| SNORD21 | 7.152634355 | 8.666470408 | 23.82432911 | 1.84E-79 | 2.76E-76 |
| SNORD2_chr3 | 7.324086643 | 9.392290471 | 23.72958454 | 4.75E-79 | 7.13E-76 |
| U59B_chr12 | 6.126794083 | 7.568293289 | 23.56296434 | 2.53E-78 | 3.80E-75 |
| SNORD110 | 6.837199633 | 8.497738253 | 23.512363 | 4.21E-78 | 6.31E-75 |
| SNORD59A | 6.574180939 | 7.368589271 | 22.91284813 | 1.76E-75 | 2.64E-72 |
| SNORD63 | 6.762328385 | 7.873791907 | 22.70701432 | 1.41E-74 | 2.10E-71 |
| SNORD38B | 5.481234185 | 7.514397997 | 22.57520173 | 5.32E-74 | 7.95E-71 |
| SNORD19 | 10.90769552 | 11.25546619 | 22.39869168 | 3.17E-73 | 4.73E-70 |
| SNORD114-22 | 8.767695777 | 7.861459307 | 22.23053874 | 1.74E-72 | 2.59E-69 |
| U3_chr2 | 7.238537904 | 8.793205321 | 21.92030398 | 4.01E-71 | 5.99E-68 |
| U3_chr8 | 7.187587765 | 8.824249049 | 21.78600479 | 1.57E-70 | 2.33E-67 |
| SNORD18A | 7.695326233 | 8.992626811 | 21.52542733 | 2.20E-69 | 3.28E-66 |
| U50_chr6 | 7.325441828 | 8.997791081 | 21.41995798 | 6.41E-69 | 9.55E-66 |
| SCARNA3 | 6.153323872 | 7.893138291 | 21.31013678 | 1.95E-68 | 2.91E-65 |
| SNORD114-9 | 7.814783411 | 7.380434454 | 21.19205466 | 6.48E-68 | 9.64E-65 |
| SNORD58B | 5.950864601 | 7.657481374 | 21.07554884 | 2.12E-67 | 3.14E-64 |
| SNORD51 | 6.163813178 | 7.958754782 | 20.95130037 | 7.48E-67 | 1.11E-63 |
| SNORD69 | 7.146940805 | 8.453836033 | 20.67411567 | 1.25E-65 | 1.85E-62 |
| U81_chr1 | 5.951715633 | 7.865681417 | 20.59723848 | 2.73E-65 | 4.05E-62 |
| SNORD6 | 6.00262227 | 8.061288246 | 20.56743838 | 3.70E-65 | 5.48E-62 |
| U28_chr11 | 6.812930807 | 9.344865145 | 20.38099415 | 2.46E-64 | 3.64E-61 |
| SNORD60 | 8.516885196 | 11.07591786 | 20.36906387 | 2.78E-64 | 4.11E-61 |
| SNORD4A | 5.743543653 | 7.332602815 | 20.02961837 | 8.75E-63 | 1.29E-59 |
| SNORD18B | 5.800130097 | 8.324078401 | 19.97169723 | 1.58E-62 | 2.33E-59 |
| SNORD24 | 5.723750726 | 7.483689865 | 19.89686358 | 3.37E-62 | 4.98E-59 |
| SNORD12C | 5.224677308 | 7.659075693 | 19.71423032 | 2.16E-61 | 3.19E-58 |
| SNORD61 | 6.128040484 | 7.693533564 | 19.70253002 | 2.43E-61 | 3.59E-58 |
| SNORD52 | 6.293160756 | 7.573220403 | 19.7018769 | 2.45E-61 | 3.61E-58 |
| U26_chr1 | 5.388299396 | 7.797524576 | 19.4549965 | 3.01E-60 | 4.43E-57 |
| U62A_chr9 | 5.451782553 | 6.453429753 | 19.29770691 | 1.49E-59 | 2.19E-56 |
| U62B_chr9 | 5.375372716 | 6.452388464 | 19.28096497 | 1.76E-59 | 2.59E-56 |
| HBII-276_chr8 | 5.046188954 | 6.623020289 | 19.23438701 | 2.83E-59 | 4.16E-56 |
| SNORD42A | 6.820150448 | 8.611894512 | 19.14979274 | 6.67E-59 | 9.80E-56 |
| U3_chr9 | 6.37676814 | 8.600124491 | 18.45796573 | 7.43E-56 | 1.09E-52 |
| SNORD71 | 4.541893503 | 5.774936817 | 17.90784669 | 1.93E-53 | 2.84E-50 |
| SNORA7B | 4.696040857 | 6.748604841 | 17.76506625 | 8.17E-53 | 1.20E-49 |
| SNORD93 | 6.529061902 | 7.657808537 | 17.67338775 | 2.06E-52 | 3.01E-49 |
| U74_chr1 | 5.907881239 | 7.839707581 | 17.65436341 | 2.49E-52 | 3.65E-49 |
| SNORD102 | 5.407650948 | 8.302471214 | 17.16869347 | 3.29E-50 | 4.82E-47 |
| SNORD114-1 | 9.01039451 | 6.902311273 | 17.11449908 | 5.68E-50 | 8.30E-47 |
| RNU3P3_chr14 | 5.877981902 | 8.099537334 | 17.06699606 | 9.14E-50 | 1.34E-46 |
| SNORD101 | 5.544203201 | 7.524356833 | 16.95204369 | 2.89E-49 | 4.22E-46 |
| ACA44_chr1 | 4.926323527 | 6.186354886 | 16.73586481 | 2.51E-48 | 3.67E-45 |
| SNORD3A | 4.543152157 | 6.671811447 | 16.68714355 | 4.09E-48 | 5.96E-45 |
| ACA58_chr3 | 5.08330363 | 7.173624262 | 16.67194532 | 4.76E-48 | 6.93E-45 |
| SNORD114-3 | 6.376797682 | 5.418644358 | 16.19303547 | 5.60E-46 | 8.16E-43 |
| SNORD58C | 5.324171469 | 8.576552786 | 16.15060467 | 8.54E-46 | 1.24E-42 |
| SNORD78 | 5.472799521 | 8.885082503 | 16.11407717 | 1.23E-45 | 1.78E-42 |
| U29_chr11 | 5.660253334 | 8.552193697 | 16.11048684 | 1.27E-45 | 1.85E-42 |
| SNORD114-12 | 6.655490363 | 5.762015752 | 16.09242279 | 1.52E-45 | 2.21E-42 |
| SNORD45A | 5.372839054 | 8.437305754 | 15.98413407 | 4.44E-45 | 6.45E-42 |
| SNORD3B-1 | 4.356647704 | 6.508310849 | 15.91414952 | 8.88E-45 | 1.29E-41 |
| SNORD33 | 4.998498629 | 6.86503898 | 15.64015207 | 1.33E-43 | 1.92E-40 |
| SNORD114-21 | 6.778098563 | 5.628537273 | 15.44422991 | 9.10E-43 | 1.32E-39 |
| SNORD58A | 4.988576386 | 7.365864127 | 15.44101591 | 9.40E-43 | 1.36E-39 |
| HBII-13_chr15 | 5.239230721 | 5.633526207 | 15.24402381 | 6.48E-42 | 9.37E-39 |
| SNORD100 | 4.668114793 | 6.310251802 | 15.18291779 | 1.18E-41 | 1.70E-38 |
| HBII-382_chr1 | 4.413588648 | 5.943013159 | 15.11333387 | 2.32E-41 | 3.35E-38 |
| SNORA36C | 3.657266234 | 4.460614522 | 14.69794071 | 1.32E-39 | 1.90E-36 |
| SNORA5C | 4.502443432 | 6.589808533 | 14.6368064 | 2.38E-39 | 3.43E-36 |
| SNORA7A | 4.28922543 | 6.304215682 | 14.57364043 | 4.38E-39 | 6.31E-36 |
| SNORD114-26 | 6.403515746 | 5.329446187 | 14.36822284 | 3.17E-38 | 4.57E-35 |
| SNORD114-23 | 6.293345437 | 5.26081532 | 14.35899363 | 3.46E-38 | 4.99E-35 |
| SNORD18C | 4.569119794 | 7.894312491 | 13.97295641 | 1.39E-36 | 2.00E-33 |
| SNORD82 | 5.365542076 | 8.11444908 | 13.84698871 | 4.61E-36 | 6.63E-33 |
| U49A_chr17 | 4.912435098 | 6.56041615 | 13.83051259 | 5.39E-36 | 7.74E-33 |
| SNORD84 | 6.400080521 | 8.114720423 | 13.7831886 | 8.45E-36 | 1.21E-32 |
| SNORD10 | 4.852547894 | 8.08356089 | 13.33395489 | 5.82E-34 | 8.34E-31 |
| SNORD37 | 4.290646023 | 6.908032213 | 13.32869887 | 6.11E-34 | 8.76E-31 |
| SNORD83B | 4.260092031 | 7.28461679 | 13.2811254 | 9.53E-34 | 1.37E-30 |
| U44_chr1 | 5.703341197 | 10.37871707 | 13.26164821 | 1.14E-33 | 1.64E-30 |
| SNORD92 | 4.105736299 | 6.292210288 | 13.23232833 | 1.50E-33 | 2.15E-30 |
| SNORD114-17 | 4.964363511 | 4.238915176 | 13.21023963 | 1.85E-33 | 2.64E-30 |
| SNORD45C | 4.530235776 | 7.912673185 | 13.02104861 | 1.07E-32 | 1.53E-29 |
| SNORD19B_chr3 | 4.048065565 | 6.442862716 | 12.66920266 | 2.76E-31 | 3.93E-28 |
| SNORA3_chr11 | 3.558689206 | 5.901477166 | 12.65190056 | 3.23E-31 | 4.60E-28 |
| SNORA45 | 4.202402489 | 5.796271352 | 12.53422715 | 9.47E-31 | 1.35E-27 |
| SNORA50 | 3.321727807 | 5.285676872 | 12.4855645 | 1.48E-30 | 2.10E-27 |
| SNORD116-24 | 4.868926492 | 5.176694329 | 12.43058447 | 2.44E-30 | 3.47E-27 |
| SNORD114-16 | 5.972857396 | 4.717662628 | 12.27904937 | 9.62E-30 | 1.37E-26 |
| SNORD12 | 4.005867291 | 6.720550806 | 12.18091705 | 2.33E-29 | 3.32E-26 |
| SNORD116-19 | 4.383512074 | 4.490893558 | 12.14586219 | 3.20E-29 | 4.54E-26 |
| U22_chr11 | 3.476574944 | 5.645156251 | 12.06210364 | 6.79E-29 | 9.64E-26 |
| SNORA3_chr16 | 4.328029309 | 6.540842321 | 12.00746259 | 1.11E-28 | 1.57E-25 |
| SNORD117 | 4.430959845 | 4.462797231 | 11.95904573 | 1.71E-28 | 2.42E-25 |
| SNORD42B | 4.093078001 | 4.853488579 | 11.95217238 | 1.82E-28 | 2.57E-25 |
| U42B | 4.072143341 | 7.068385371 | 11.86168208 | 4.07E-28 | 5.76E-25 |
| ACA24_chr4 | 5.54220616 | 7.38538051 | 11.85302903 | 4.40E-28 | 6.22E-25 |
| SNORD116-16 | 4.435618656 | 4.516238278 | 11.84802543 | 4.60E-28 | 6.50E-25 |
| SNORD111B | 3.354531574 | 5.522068704 | 11.78866086 | 7.79E-28 | 1.10E-24 |
| SNORD114-28 | 4.575501213 | 4.18038105 | 11.74695124 | 1.13E-27 | 1.59E-24 |
| HBII-436_chr15 | 4.793529697 | 5.946829782 | 11.69261511 | 1.82E-27 | 2.57E-24 |
| SNORA24 | 4.31859094 | 5.814920917 | 11.67385006 | 2.15E-27 | 3.03E-24 |
| SNORD5 | 3.554027987 | 6.622049694 | 11.61389149 | 3.65E-27 | 5.15E-24 |
| SNORD89 | 3.996528712 | 6.800750599 | 11.54315945 | 6.81E-27 | 9.58E-24 |
| SNORD14C | 3.816485149 | 6.992148163 | 11.53967256 | 7.02E-27 | 9.87E-24 |
| SNORD116-17 | 4.261032382 | 4.454832108 | 11.51094548 | 9.03E-27 | 1.27E-23 |
| HBII-85-20_chr15 | 4.326615869 | 4.491974094 | 11.50869465 | 9.21E-27 | 1.29E-23 |
| SNORD68 | 3.570287287 | 5.556062912 | 11.50172676 | 9.79E-27 | 1.37E-23 |
| SNORD114-14 | 4.518502711 | 3.727826236 | 11.42739267 | 1.88E-26 | 2.63E-23 |
| SCARNA13 | 3.077624997 | 4.774914517 | 11.40915235 | 2.20E-26 | 3.08E-23 |
| U79_chr1 | 4.538965133 | 6.485114454 | 11.39603892 | 2.47E-26 | 3.46E-23 |
| SNORD116-18 | 4.315441892 | 4.388080703 | 11.39142195 | 2.57E-26 | 3.60E-23 |
| SNORD114-15 | 5.109592696 | 3.78828172 | 11.3535079 | 3.58E-26 | 5.00E-23 |
| HBII-85-21_chr15 | 4.239424302 | 4.452656019 | 11.30134396 | 5.63E-26 | 7.87E-23 |
| SNORA32 | 2.396001004 | 3.984645038 | 11.08842395 | 3.55E-25 | 4.96E-22 |
| SNORD114-25 | 4.372251956 | 3.857636479 | 10.96556569 | 1.02E-24 | 1.42E-21 |
| SNORD91A | 3.170237353 | 4.919277832 | 10.94404405 | 1.22E-24 | 1.71E-21 |
| U3_chrX | 3.359220962 | 4.633546077 | 10.90007671 | 1.78E-24 | 2.48E-21 |
| SNORD12B | 3.814499188 | 7.677405044 | 10.77512441 | 5.15E-24 | 7.18E-21 |
| SNORD113-8 | 4.2093919 | 4.221720164 | 10.73821352 | 7.05E-24 | 9.80E-21 |
| SNORD96A | 3.711564639 | 6.478221633 | 10.59496908 | 2.36E-23 | 3.28E-20 |
| SNORD34 | 4.122077707 | 7.081834594 | 10.58640959 | 2.53E-23 | 3.52E-20 |
| SNORD19B_chr3 | 5.243422096 | 7.638411321 | 10.56034105 | 3.15E-23 | 4.37E-20 |
| SNORD13 | 4.606681762 | 8.674243861 | 10.55022451 | 3.43E-23 | 4.76E-20 |
| SCARNA4 | 3.914744586 | 6.395295284 | 10.49133778 | 5.61E-23 | 7.78E-20 |
| U56_chr20 | 3.417164097 | 6.373209953 | 10.28849704 | 3.02E-22 | 4.19E-19 |
| SNORD125 | 3.258136038 | 5.222147093 | 10.27445026 | 3.40E-22 | 4.70E-19 |
| SNORD116-25 | 4.67988285 | 5.726717618 | 10.22268323 | 5.20E-22 | 7.20E-19 |
| SNORA69 | 3.906657624 | 7.438400275 | 10.20528089 | 6.00E-22 | 8.30E-19 |
| snR38A_chr17 | 3.388928982 | 5.967168206 | 10.20174695 | 6.18E-22 | 8.54E-19 |
| snR38C_chr17 | 3.118119732 | 4.708524331 | 10.16991969 | 8.03E-22 | 1.11E-18 |
| SNORD41 | 3.529349156 | 6.557100541 | 10.13963797 | 1.03E-21 | 1.42E-18 |
| SNORA65 | 2.68360539 | 4.758646888 | 9.994776025 | 3.35E-21 | 4.62E-18 |
| U76_chr1 | 3.139572178 | 5.521275266 | 9.985979384 | 3.60E-21 | 4.96E-18 |
| SNORD127 | 3.571953698 | 6.056555034 | 9.962332313 | 4.36E-21 | 6.00E-18 |
| SNORD88C | 3.179217099 | 5.875283463 | 9.922742514 | 6.01E-21 | 8.26E-18 |
| ACA61_chr1 | 2.830699045 | 4.405892012 | 9.834039669 | 1.23E-20 | 1.69E-17 |
| SNORD7 | 3.198011816 | 5.318631136 | 9.651609676 | 5.29E-20 | 7.26E-17 |
| SNORD83A | 3.332015768 | 6.462085323 | 9.648526149 | 5.42E-20 | 7.43E-17 |
| SNORD123 | 4.290300774 | 6.407061933 | 9.629929605 | 6.28E-20 | 8.61E-17 |
| SNORD114-5 | 4.205043411 | 3.415791457 | 9.609107179 | 7.41E-20 | 1.01E-16 |
| U3_chr8 | 4.119240567 | 5.147611246 | 9.538906389 | 1.29E-19 | 1.77E-16 |
| U54 | 3.655632412 | 6.62354862 | 9.535661551 | 1.32E-19 | 1.81E-16 |
| SNORA7 | 3.108713909 | 5.153552919 | 9.451794773 | 2.56E-19 | 3.50E-16 |
| SNORD17 | 2.503458028 | 3.631279818 | 9.414700733 | 3.43E-19 | 4.68E-16 |
| mgU2-25/61_chr1 | 2.49841718 | 3.827264932 | 9.39348375 | 4.05E-19 | 5.53E-16 |
| SNORA77_chr1 | 2.620987438 | 3.64683441 | 9.262714503 | 1.12E-18 | 1.53E-15 |
| SNORD94 | 2.797965725 | 4.760984309 | 9.218179438 | 1.58E-18 | 2.16E-15 |
| U47_chr1 | 3.122274969 | 5.659224497 | 9.143360873 | 2.82E-18 | 3.83E-15 |
| SNORD55 | 2.888959413 | 5.029635147 | 8.997210812 | 8.61E-18 | 1.17E-14 |
| SNORD103B | 2.806301379 | 4.969855984 | 8.937386302 | 1.36E-17 | 1.84E-14 |
| SNORD32A | 3.704631653 | 7.38463429 | 8.750742814 | 5.52E-17 | 7.50E-14 |
| snoU2-30 | 2.577976193 | 4.667400835 | 8.668922008 | 1.02E-16 | 1.38E-13 |
| SNORD114-11 | 4.164426866 | 2.92406513 | 8.492249336 | 3.74E-16 | 5.07E-13 |
| SNORD103A | 2.68096609 | 4.99495154 | 8.48941738 | 3.81E-16 | 5.17E-13 |
| SNORD116-14 | 3.501521482 | 4.1845324 | 8.452848005 | 4.98E-16 | 6.75E-13 |
| ACA31_chr13 | 3.82123616 | 6.906147894 | 8.417613314 | 6.44E-16 | 8.72E-13 |
| SNORD113-7 | 3.469732826 | 4.402804247 | 8.303460994 | 1.47E-15 | 1.99E-12 |
| SCARNA11 | 2.389259229 | 3.913137569 | 8.28131496 | 1.73E-15 | 2.34E-12 |
| U3_chr17 | 2.555585454 | 2.933486255 | 8.207345334 | 2.94E-15 | 3.97E-12 |
| SNORD126 | 2.754248754 | 5.332050345 | 8.187672077 | 3.38E-15 | 4.57E-12 |
| SNORA36A | 2.185688005 | 2.650847011 | 8.143177241 | 4.64E-15 | 6.27E-12 |
| SNORD114-20 | 3.864205446 | 3.499499744 | 8.121844372 | 5.40E-15 | 7.29E-12 |
| SNORD70 | 2.572203962 | 3.633462692 | 8.034160701 | 1.01E-14 | 1.35E-11 |
| U50B_chr6 | 3.325301623 | 4.307421729 | 7.97977887 | 1.47E-14 | 1.98E-11 |
| snoU2_19 | 2.660996313 | 4.279565758 | 7.939152075 | 1.96E-14 | 2.63E-11 |
| U49B_chr17 | 2.844463348 | 5.466576974 | 7.920462166 | 2.23E-14 | 3.00E-11 |
| SCARNA5 | 2.250651888 | 4.682690476 | 7.846933766 | 3.72E-14 | 5.00E-11 |
| SNORA81 | 2.294530222 | 3.364206508 | 7.829322669 | 4.20E-14 | 5.64E-11 |
| SNORA19 | 2.177665914 | 3.555446936 | 7.821081961 | 4.45E-14 | 5.97E-11 |
| SNORA54 | 2.2523966 | 3.278162439 | 7.69263873 | 1.08E-13 | 1.44E-10 |
| SNORD113-3 | 2.851004001 | 2.242033902 | 7.563630323 | 2.59E-13 | 3.46E-10 |
| SNORD116-8 | 3.19506732 | 3.81860973 | 7.45570422 | 5.34E-13 | 7.14E-10 |
| SNORD114-29 | 3.160150315 | 2.315820747 | 7.359808555 | 1.01E-12 | 1.35E-09 |
| U80_chr1 | 2.66062244 | 5.495879915 | 7.351713632 | 1.07E-12 | 1.42E-09 |
| SNORD105 | 2.611597974 | 4.966029034 | 7.28642255 | 1.64E-12 | 2.19E-09 |
| SNORD114-10 | 2.686235196 | 1.70190314 | 7.218944069 | 2.55E-12 | 3.40E-09 |
| SNORD111 | 3.096509849 | 6.666685592 | 7.208112697 | 2.74E-12 | 3.64E-09 |
| SNORA60 | 2.055560806 | 2.81516004 | 7.121550951 | 4.80E-12 | 6.39E-09 |
| U85_chr12 | 1.942785876 | 3.909974136 | 7.117363431 | 4.93E-12 | 6.56E-09 |
| SNORD116-1 | 2.819111763 | 3.461840682 | 7.078767282 | 6.32E-12 | 8.40E-09 |
| SNORD114-6 | 3.11931695 | 2.128292326 | 6.921400513 | 1.72E-11 | 2.29E-08 |
| SNORA28 | 1.991976183 | 3.721180505 | 6.892865137 | 2.06E-11 | 2.74E-08 |
| SNORD53_SNORD92 | 2.211216186 | 4.389140468 | 6.869713052 | 2.39E-11 | 3.17E-08 |
| SNORD36C | 2.627787718 | 4.951032737 | 6.816523403 | 3.33E-11 | 4.42E-08 |
| SNORD116-3 | 2.960694001 | 3.787789803 | 6.80600277 | 3.56E-11 | 4.71E-08 |
| mgU2-19/30_chr11 | 1.622420736 | 3.194971345 | 6.75199743 | 4.98E-11 | 6.59E-08 |
| SNORD113-6 | 2.764838303 | 3.24328975 | 6.692386828 | 7.21E-11 | 9.53E-08 |
| SNORD116-6 | 2.7942111 | 3.4766575 | 6.691115704 | 7.26E-11 | 9.59E-08 |
| SNORD73A | 2.394630771 | 4.91935809 | 6.664992658 | 8.53E-11 | 1.13E-07 |
| SNORD116-9 | 2.860266008 | 3.836848016 | 6.645596469 | 9.61E-11 | 1.27E-07 |
| SNORD118 | 2.7001729 | 5.206893457 | 6.638825092 | 1.00E-10 | 1.32E-07 |
| SNORD15B | 2.28885087 | 5.040738495 | 6.614915286 | 1.16E-10 | 1.53E-07 |
| SNORA46 | 1.808754189 | 3.538377615 | 6.608047539 | 1.21E-10 | 1.59E-07 |
| SNORD46_chr1 | 2.196877843 | 5.093924343 | 6.572834443 | 1.50E-10 | 1.97E-07 |
| U8_chr9 | 1.912685053 | 2.179607849 | 6.300903954 | 7.62E-10 | 1.00E-06 |
| SNORD14D | 3.375350031 | 5.54258059 | 6.265377122 | 9.39E-10 | 1.23E-06 |
| ACA16_chr1 | 1.7574804 | 3.784532018 | 6.264903229 | 9.41E-10 | 1.24E-06 |
| SNORD114-24 | 2.294613826 | 1.380627175 | 6.207601374 | 1.32E-09 | 1.72E-06 |
| SNORD113-9 | 2.344873154 | 2.202327829 | 6.188642574 | 1.47E-09 | 1.92E-06 |
| SNORA71B | 2.344608318 | 5.599153 | 6.171881932 | 1.62E-09 | 2.12E-06 |
| SNORD114-27 | 2.193822092 | 1.21606288 | 6.138514444 | 1.96E-09 | 2.57E-06 |
| SNORD114-4 | 1.866430929 | 1.071681369 | 5.964109662 | 5.30E-09 | 6.93E-06 |
| SCARNA8 | 1.790840811 | 2.619579369 | 5.764199821 | 1.61E-08 | 2.10E-05 |
| SNORD116-2 | 2.445209502 | 3.32598257 | 5.705423386 | 2.22E-08 | 2.90E-05 |
| SNORA77_chr22 | 1.620733853 | 2.635889933 | 5.671782687 | 2.67E-08 | 3.48E-05 |
| SNORD53 | 1.865860127 | 3.633487285 | 5.656389153 | 2.90E-08 | 3.78E-05 |
| SNORD97 | 1.863635019 | 4.029005855 | 5.540219476 | 5.40E-08 | 7.03E-05 |
| SNORD116-26 | 2.334398813 | 3.383075912 | 5.509349181 | 6.36E-08 | 8.28E-05 |
| SNORD15A | 1.679978164 | 3.836597185 | 5.409702197 | 1.07E-07 | 0.000139557 |
| SCARNA18 | 1.695589201 | 2.855685907 | 5.374256589 | 1.29E-07 | 0.000167664 |
| SNORD91B | 1.657327196 | 3.395715123 | 5.36845718 | 1.33E-07 | 0.000172647 |
| SCARNA6 | 1.16105179 | 2.116282279 | 5.351463975 | 1.45E-07 | 0.000188366 |
| SNORA77_chr15 | 1.163006434 | 0.879428388 | 5.342978564 | 1.52E-07 | 0.000196651 |
| SNORD45B | 2.136150991 | 4.11050416 | 5.227681727 | 2.74E-07 | 0.000354384 |
| SNORA74A | 1.483102366 | 2.780651891 | 5.207749499 | 3.03E-07 | 0.000391701 |
| SNORD88A | 1.694544026 | 3.812732765 | 5.190179392 | 3.31E-07 | 0.00042769 |
| SNORD2_chr10 | 1.982843988 | 3.636883094 | 5.096688548 | 5.28E-07 | 0.000682216 |
| SNORD114-13 | 1.99533315 | 2.175353747 | 4.984888543 | 9.15E-07 | 0.001181762 |
| SNORD8 | 1.586026125 | 2.495880282 | 4.983815041 | 9.20E-07 | 0.001187044 |
| snoU13 | 2.074084321 | 4.376354321 | 4.971226073 | 9.78E-07 | 0.001261128 |
| SNORD116-29 | 2.093181104 | 2.681066679 | 4.956333663 | 1.05E-06 | 0.00135473 |
| U75_chr1 | 2.134208689 | 5.710296408 | 4.956070927 | 1.05E-06 | 0.001355406 |
| SNORA53 | 1.404611544 | 2.954392979 | 4.924914339 | 1.22E-06 | 0.001574836 |
| ACA62_chr17 | 1.638295335 | 3.856511946 | 4.904253498 | 1.35E-06 | 0.001738393 |
| SNORD46_chr7 | 1.65123655 | 4.630654522 | 4.807273834 | 2.15E-06 | 0.002759477 |
| SNORD72 | 1.628496842 | 4.303103522 | 4.796032352 | 2.27E-06 | 0.002907877 |
| SNORD114-30 | 1.486164735 | 0.871703198 | 4.776371258 | 2.49E-06 | 0.003187941 |
| SNORA2B | 1.754848763 | 1.922869291 | 4.671918859 | 4.05E-06 | 0.00518611 |
| SNORD105B | 1.849574195 | 4.234526377 | 4.656898573 | 4.34E-06 | 0.005554227 |
| SNORD114-19 | 1.444625158 | 0.799420573 | 4.542248632 | 7.32E-06 | 0.009366122 |
| SNORD66_chr6 | 1.363342457 | 1.316863804 | 4.317585994 | 1.98E-05 | 0.025290918 |
| SNORA33 | 1.369645405 | 2.984368668 | 4.26228032 | 2.51E-05 | 0.032046782 |
| SNORD113-5 | 1.548032052 | 1.7326654 | 4.229523929 | 2.89E-05 | 0.036831864 |
| SNORD45 | 1.814978628 | 3.303471391 | 4.195315941 | 3.34E-05 | 0.042554115 |
| SNORA14B | 1.51805595 | 2.817375798 | 4.18665845 | 3.46E-05 | 0.044105466 |
