# Supplementary material for: Small nucleolar RNAs signature (SNORS) identified clinical outcome and prognosis of bladder cancer (BLCA)
Source: Cancer Cell Int. 2020 Jul 10;20:299. doi: 10.1186/s12935-020-01393-7 (PMC7350589; doi:10.1186/s12935-020-01393-7)
Supplement: Supplementary file 3 — Additional file 3: Table S3. Summary of univariate Cox regression analysis for snoRNAs in TCGA-BLCA cohort. [file 12935_2020_1393_MOESM3_ESM.docx]

**Additional file 3: Table S3 Summary of univariate Cox regression analysis for snoRNAs in TCGA-BLCA cohort (n = 392)**

| id | HR | HR.95L | HR.95H | pvalue |
| --- | --- | --- | --- | --- |
| 14q(II-1);ENSG00000199575;SNORD114-1_chr14_101416169_101416241 | 1.000326071 | 1.000180901 | 1.000471262 | 1.07E-05 |
| ACA43_chr9_139620555_139620691 | 1.006975967 | 1.003168459 | 1.010797927 | 0.000322343 |
| ENSG00000252213;SNORA74_chr5_138611869_138612009 | 1.050212865 | 1.021447349 | 1.079788461 | 0.000545095 |
| 14q(II-27);ENSG00000200636;SNORD114-27_chr14_101454497_101454567 | 1.02752453 | 1.011277377 | 1.044032709 | 0.000840789 |
| 14q(II-3);ENSG00000201839;SNORD114-3_chr14_101419685_101419760 | 1.001150562 | 1.00043134 | 1.0018703 | 0.001712158 |
| U50B_chr6_86387306_86387377 | 1.001502051 | 1.000548337 | 1.002456674 | 0.002017189 |
| ENSG00000202335;SNORD50_chr12_110934157_110934226 | 1.001556684 | 1.000549884 | 1.002564498 | 0.002435274 |
| U49A_chr17_16343349_16343420 | 1.000761639 | 1.000254331 | 1.001269204 | 0.003251163 |
| ENSG00000212195;U3_chr17_56709003_56709197 | 1.022880186 | 1.00733873 | 1.038661418 | 0.003779512 |
| ENSG00000212611;SNORD30_chrX_113260261_113260330 | 1.089268865 | 1.027744919 | 1.154475823 | 0.003944821 |
| U99;ENSG00000206597;SNORA57_chr11_62432893_62433042 | 1.015113474 | 1.004141761 | 1.02620507 | 0.006821956 |
| 14q(II-14);ENSG00000199593;SNORD114-14_chr14_101438439_101438514 | 1.002456948 | 1.000493948 | 1.004423799 | 0.014137367 |
| ENSG00000238768;snoU13_chr11_71815120_71815216 | 0.860422127 | 0.758140512 | 0.976502674 | 0.019900759 |
| U14B;ENSG00000272034;SNORD14A_chr11_17096199_17096291 | 1.001964029 | 1.000283505 | 1.003647377 | 0.021967195 |
| ACA8;ENSG00000207304;SNORA8_chr11_93465526_93465665 | 1.01547639 | 1.002124389 | 1.029006288 | 0.022953187 |
| 14q(II-17);ENSG00000201569;SNORD114-17_chr14_101441142_101441217 | 1.002738493 | 1.000333007 | 1.005149765 | 0.025637078 |
| 14q(II-5);ENSG00000199798;SNORD114-5_chr14_101421706_101421776 | 1.002166065 | 1.000223804 | 1.004112097 | 0.028811757 |
| 14q(I-9);ENSG00000201950;SNORD113-9_chr14_101411985_101412057 | 1.013934224 | 1.001264454 | 1.026764315 | 0.031011158 |
| U60;ENSG00000206630;SNORD60_chr16_2205023_2205106 | 1.000016686 | 1.000000824 | 1.000032547 | 0.039222552 |
| 14q(I-3);ENSG00000201700;SNORD113-3_chr14_101396255_101396328 | 1.013642176 | 1.000606555 | 1.026847622 | 0.040190766 |
| HBII-108B;ENSG00000238862;SNORD19B_chr3_52724759_52724843 | 0.998445179 | 0.996927105 | 0.999965565 | 0.045035708 |
| ACA36B;ENSG00000222370;SNORA36B_chr1_220373887_220374018 | 0.998792214 | 0.997606543 | 0.999979294 | 0.046137789 |
| 14q(II-12);ENSG00000202270;SNORD114-12_chr14_101435284_101435359 | 1.000358048 | 1.000004285 | 1.000711936 | 0.047288292 |
| ENSG00000221376;SNORA77_chr15_69617632_69617759 | 1.089655453 | 0.998904192 | 1.18865154 | 0.052959404 |
| HBII-52-5;ENSG00000200503;SNORD115-5_chr15_25423884_25423966 | 0.929794508 | 0.862957883 | 1.001807672 | 0.055810492 |
| 14q(II-10);ENSG00000200279;SNORD114-10_chr14_101433388_101433460 | 1.007533661 | 0.999811205 | 1.015315765 | 0.055893194 |
| 14q(II-23);ENSG00000200406;SNORD114-23_chr14_101450212_101450284 | 1.000329712 | 0.999987185 | 1.000672356 | 0.059210786 |
| ENSG00000199934;SNORD81_chr1_86057963_86058039 | 1.088546473 | 0.995962647 | 1.18973681 | 0.061378114 |
| U59A;ENSG00000207031;SNORD59A_chr12_57038810_57038885 | 0.999240691 | 0.998433823 | 1.000048211 | 0.065329976 |
| U59B_chr12_57037463_57037538 | 0.999238023 | 0.998424411 | 1.000052298 | 0.066634715 |
| 14q(II-26);ENSG00000200413;SNORD114-26_chr14_101453382_101453454 | 1.000308664 | 0.999978368 | 1.000639069 | 0.067014298 |
| U22_chr11_62620381_62620507 | 1.002023322 | 0.999850127 | 1.004201241 | 0.068052368 |
| 14q(II-16);ENSG00000199914;SNORD114-16_chr14_101439931_101440001 | 1.000351826 | 0.999972632 | 1.000731164 | 0.068991082 |
| ENSG00000238498;snoU13_chr22_42472062_42472165 | 0.930477027 | 0.860414956 | 1.00624413 | 0.071213188 |
| 14q(II-25);ENSG00000200612;SNORD114-25_chr14_101452393_101452465 | 1.001380011 | 0.999868691 | 1.002893615 | 0.073524957 |
| HBII-52-32;ENSG00000200949;SNORD115-32_chr15_25474113_25474195 | 0.963621465 | 0.925253945 | 1.003579972 | 0.07384408 |
| ENSG00000212206;SNORA69_chr17_8232901_8233037 | 0.989269862 | 0.977628072 | 1.001050285 | 0.074072991 |
| 14q(II-21);ENSG00000272344;SNORD114-21_chr14_101448311_101448383 | 1.000281751 | 0.999969254 | 1.000594346 | 0.077211361 |
| ENSG00000252193;SCARNA15_chr20_41933195_41933319 | 0.969520715 | 0.936730002 | 1.003459284 | 0.077858735 |
| ACA6;ENSG00000206760;SNORA6_chr3_39449880_39450030 | 1.003604498 | 0.99959473 | 1.007630351 | 0.078151723 |
| SNORD121B;ENSG00000238300;SNORD121B_chr9_33934294_33934374 | 0.981874377 | 0.961899111 | 1.00226446 | 0.081111255 |
| ENSG00000199666;U3_chr1_91123307_91123520 | 1.036991648 | 0.99515459 | 1.080587568 | 0.083846346 |
| U77_chr1_173835438_173835508 | 1.007044882 | 0.999032949 | 1.015121069 | 0.084967892 |
| ENSG00000221496;U3_chr17_42381382_42381570 | 0.839701706 | 0.687822463 | 1.025117662 | 0.08611334 |
| ENSG00000202023;SNORD81_chr7_136972434_136972509 | 0.91159735 | 0.818931584 | 1.014748661 | 0.09059367 |
| U83A;ENSG00000209482;SNORD83A_chr22_39711217_39711312 | 1.000756516 | 0.99987431 | 1.001639499 | 0.092837071 |
| U43;ENSG00000263764;SNORD43_chr22_39715055_39715118 | 0.999790908 | 0.999546991 | 1.000034885 | 0.093005957 |
| 14q(II-9);ENSG00000201240;SNORD114-9_chr14_101432365_101432437 | 1.000122014 | 0.999977408 | 1.000266642 | 0.098181445 |
| U50_chr6_86387011_86387086 | 1.000092214 | 0.999982331 | 1.00020211 | 0.100013773 |
| U19-2;ENSG00000212402;SNORA74B_chr5_172447728_172447932 | 0.949928141 | 0.893494586 | 1.009926068 | 0.100200707 |
| HBII-336;ENSG00000221740;SNORD93_chr7_22896231_22896305 | 1.000319519 | 0.999936552 | 1.000702633 | 0.102008089 |
| 14q(II-4);ENSG00000200832;SNORD114-4_chr14_101420710_101420785 | 1.013947779 | 0.99722738 | 1.030948527 | 0.102533098 |
| ENSG00000238707;SNORD2_chr10_58355723_58355791 | 1.00428424 | 0.999143189 | 1.009451744 | 0.102549845 |
| ENSG00000238746;snoU13_chr9_97934777_97934880 | 0.940633086 | 0.873751558 | 1.012634077 | 0.103877604 |
| U76_chr1_173835772_173835852 | 1.00172989 | 0.999599984 | 1.003864335 | 0.111486745 |
| 14q(I-5);ENSG00000272474;SNORD113-5_chr14_101404523_101404601 | 1.006029796 | 0.998607466 | 1.013507293 | 0.111578217 |
| ENSG00000201898;SNORA72_chr1_224367343_224367469 | 0.886496363 | 0.763864295 | 1.028815992 | 0.112741657 |
| ENSG00000202269;U8_chr5_15110895_15111029 | 1.004779069 | 0.998833776 | 1.01075975 | 0.115353206 |
| HBII-95;ENSG00000238317;SNORD11_chr2_203157772_203157859 | 1.006209477 | 0.998434955 | 1.014044537 | 0.117772815 |
| ENSG00000201791;SNORA63_chr1_178722789_178722907 | 0.951885019 | 0.894709089 | 1.012714748 | 0.118711558 |
| 14q(I-8);ENSG00000200367;SNORD113-8_chr14_101409787_101409861 | 1.001763299 | 0.999527968 | 1.004003629 | 0.122173234 |
| HBII-210;ENSG00000212452;SNORD69_chr3_52726751_52726828 | 0.999794147 | 0.999529652 | 1.000058712 | 0.127244883 |
| HBII-52-14;ENSG00000199960;SNORD115-14_chr15_25440067_25440148 | 0.942789539 | 0.873550773 | 1.017516259 | 0.130083404 |
| ENSG00000206976;SNORA7_chr11_3943797_3943933 | 1.036668374 | 0.989302662 | 1.086301856 | 0.131239183 |
| mgh18S-121;ENSG00000238578;SNORD4A_chr17_27049599_27049671 | 0.999325195 | 0.998447719 | 1.000203443 | 0.132042144 |
| HBI-100;ENSG00000252906;SCARNA3_chr1_175937532_175937676 | 0.999549112 | 0.998958644 | 1.000139929 | 0.134687225 |
| ENSG00000212309;SNORD70_chr2_203142831_203142915 | 0.992506848 | 0.982764621 | 1.00234565 | 0.135059381 |
| ENSG00000212532;SNORD66_chr6_51329488_51329563 | 0.967501491 | 0.926460794 | 1.010360223 | 0.135199016 |
| ACA17_chr9_139621198_139621331 | 1.01068543 | 0.996683518 | 1.024884048 | 0.135371462 |
| U51;ENSG00000207047;SNORD51_chr2_207026602_207026681 | 0.999561856 | 0.998986381 | 1.000137663 | 0.135834942 |
| HBII-438B;ENSG00000239169;SNORD109B_chr15_25523489_25523556 | 0.980393079 | 0.955034313 | 1.00642519 | 0.13861645 |
| U95;ENSG00000264549;SNORD95_chr5_180670312_180670379 | 0.999905823 | 0.999780667 | 1.000030995 | 0.140305142 |
| HBII-295;ENSG00000212447;SNORD90_chr9_125642491_125642602 | 0.99983572 | 0.99961715 | 1.000054338 | 0.140792398 |
| ACA61_chr1_28906275_28906405 | 1.003155336 | 0.998948592 | 1.007379795 | 0.141743318 |
| ENSG00000252981;U3_chr2_170672789_170672888 | 0.964217101 | 0.918385108 | 1.01233634 | 0.142508355 |
| 14q(II-15);ENSG00000201557;SNORD114-15_chr14_101439006_101439078 | 1.000653542 | 0.999779215 | 1.001528634 | 0.142955794 |
| HBII-419;ENSG00000221182;SNORD98_chr10_70514928_70514995 | 0.999957778 | 0.999901129 | 1.000014431 | 0.144090124 |
| ENSG00000252258;SNORA70_chr18_3025432_3025564 | 0.974527955 | 0.94121279 | 1.009022344 | 0.145984101 |
| 14q(I-7);ENSG00000200632;SNORD113-7_chr14_101407462_101407539 | 1.001532186 | 0.999465915 | 1.003602729 | 0.146232404 |
| HBII-52-10;ENSG00000201943;SNORD115-10_chr15_25432682_25432763 | 0.945254198 | 0.876033032 | 1.019944986 | 0.14677974 |
| U52;ENSG00000201754;SNORD52_chr6_31804852_31804919 | 0.999612587 | 0.999086421 | 1.000139029 | 0.149174427 |
| ACA57;ENSG00000251898;SCARNA11_chr12_6690638_6690775 | 0.992986056 | 0.9834324 | 1.002632522 | 0.153589513 |
| ACA13;ENSG00000238363;SNORA13_chr5_111497181_111497314 | 1.033449132 | 0.987754833 | 1.081257286 | 0.153874601 |
| U18C;ENSG00000199574;SNORD18C_chr15_66793588_66793656 | 0.999640355 | 0.999146152 | 1.000134802 | 0.153951195 |
| U56_chr20_2637269_2637340 | 0.998902832 | 0.997379641 | 1.000428349 | 0.158560752 |
| 14q(II-6);ENSG00000201263;SNORD114-6_chr14_101423502_101423574 | 1.001670108 | 0.999346639 | 1.003998979 | 0.159024265 |
| HBII-85-3;ENSG00000207014;SNORD116-3_chr15_25302005_25302102 | 0.996855809 | 0.992485045 | 1.001245821 | 0.160131471 |
| U44_chr1_173835103_173835166 | 1.000034921 | 0.999985809 | 1.000084036 | 0.16343047 |
| ENSG00000212624;SNORA26_chr1_52190447_52190568 | 1.035789718 | 0.985496681 | 1.088649369 | 0.166151188 |
| 14q(II-11);ENSG00000200608;SNORD114-11_chr14_101434447_101434522 | 1.000703467 | 0.999698434 | 1.001709511 | 0.170169709 |
| HBII-239;ENSG00000223224;SNORD71_chr16_71792304_71792390 | 0.997933151 | 0.99498062 | 1.000894444 | 0.171131137 |
| HBII-85-29;ENSG00000207245;SNORD116-29_chr15_25351666_25351751 | 0.994534457 | 0.986728196 | 1.002402476 | 0.17284115 |
| HBII-52-4;ENSG00000200680;SNORD115-4_chr15_25421978_25422060 | 0.931848307 | 0.841813686 | 1.031512413 | 0.173353543 |
| ACA36;ENSG00000206948;SNORA36A_chrX_153996802_153996934 | 0.98083921 | 0.953651593 | 1.008801918 | 0.177356128 |
| ENSG00000238936;SNORD65_chr8_41284174_41284246 | 1.04909401 | 0.978027849 | 1.125324033 | 0.180514226 |
| U105B;ENSG00000238531;SNORD105B_chr19_10220432_10220511 | 0.99694499 | 0.992490379 | 1.001419595 | 0.18053689 |
| HBII-85-9;ENSG00000206727;SNORD116-9_chr15_25318252_25318349 | 0.996981789 | 0.992548956 | 1.00143442 | 0.183678961 |
| HBII-438A_chr15_25287120_25287187 | 0.984487604 | 0.962029723 | 1.007469748 | 0.184218947 |
| ACA60;ENSG00000199266;SNORA60_chr20_37078011_37078147 | 0.985288828 | 0.963834226 | 1.007221003 | 0.187030997 |
| 14q(II-22);ENSG00000202293;SNORD114-22_chr14_101449262_101449334 | 1.00003681 | 0.999981956 | 1.000091668 | 0.18843408 |
| U103;ENSG00000200154;SNORD103A_chr1_31408532_31408623 | 1.001925177 | 0.999055704 | 1.004802892 | 0.188727926 |
| U63;ENSG00000206989;SNORD63_chr5_137896731_137896799 | 0.999657902 | 0.999146968 | 1.000169097 | 0.189607282 |
| HBII-85-5;ENSG00000207191;SNORD116-5_chr15_25307478_25307575 | 0.986607203 | 0.966866379 | 1.006751082 | 0.191043237 |
| ENSG00000202389;SNORA70_chr17_26349356_26349490 | 0.978626873 | 0.947340498 | 1.010946495 | 0.192492601 |
| U61;ENSG00000206979;SNORD61_chrX_135961357_135961430 | 0.999616691 | 0.999035777 | 1.000197942 | 0.196136562 |
| ENSG00000201945;SNORA70_chr12_121544846_121544977 | 0.906438602 | 0.780843352 | 1.0522353 | 0.196749895 |
| HBII-296A;ENSG00000212163;SNORD91A_chr17_2233474_2233664 | 0.996946621 | 0.99232798 | 1.001586758 | 0.196789994 |
| ACA16_chr1_28907431_28907565 | 1.003032302 | 0.998405688 | 1.007680356 | 0.199301281 |
| HBII-180A;ENSG00000221241;SNORD88A_chr19_51302695_51302792 | 0.994829505 | 0.986978814 | 1.002742641 | 0.199698774 |
| ENSG00000200237;SNORA70_chr19_9930630_9930770 | 0.916927259 | 0.802895754 | 1.047154122 | 0.200561321 |
| HBII-85-23;ENSG00000207375;SNORD116-23_chr15_25336931_25337025 | 0.987445479 | 0.968429418 | 1.006834939 | 0.20287533 |
| ENSG00000206661;SNORA70_chr8_4985801_4985934 | 0.978230131 | 0.945401595 | 1.012198619 | 0.206307534 |
| E2;ENSG00000202363;SNORA62_chr3_39452544_39452698 | 1.00909326 | 0.994906333 | 1.023482486 | 0.210183101 |
| ACA23;ENSG00000201998;SNORA23_chr11_9450312_9450501 | 1.02404937 | 0.986406153 | 1.063129126 | 0.213617932 |
| U92;ENSG00000251733;SCARNA8_chr9_19063653_19063784 | 0.987398069 | 0.967839878 | 1.007351494 | 0.214087811 |
| ENSG00000238563;snoU13_chr6_114162001_114162089 | 0.980402727 | 0.950082331 | 1.01169075 | 0.216901087 |
| ACA44_chr1_28906892_28907024 | 1.000782925 | 0.999536755 | 1.002030649 | 0.218288352 |
| U105;ENSG00000209645;SNORD105_chr19_10218326_10218411 | 0.997976389 | 0.994731469 | 1.001231895 | 0.222822503 |
| ENSG00000200206;SNORD74_chr15_86716429_86716501 | 0.931911942 | 0.831474132 | 1.044482124 | 0.225524259 |
| HBII-115;ENSG00000221803;SNORD23_chr19_48259109_48259219 | 1.030234405 | 0.981544543 | 1.081339545 | 0.227876344 |
| U14A;ENSG00000201403;SNORD14B_chr11_17097324_17097415 | 1.005421524 | 0.996583223 | 1.014338209 | 0.230056566 |
| U69;ENSG00000206622;SNORA69_chrX_118921315_118921447 | 0.999744095 | 0.99932579 | 1.000162574 | 0.23066839 |
| ENSG00000212615;SNORD58_chr14_45557446_45557511 | 0.979449184 | 0.946721902 | 1.013307817 | 0.231094535 |
| HBII-55;ENSG00000221116;SNORD110_chr20_2634857_2634932 | 0.999770887 | 0.999395133 | 1.000146782 | 0.232199261 |
| ENSG00000252699;SNORA21_chr17_37007777_37007912 | 0.956043703 | 0.887724869 | 1.029620319 | 0.23471013 |
| SNORD123;ENSG00000239112;SNORD123_chr5_9548947_9549017 | 0.999531544 | 0.998757718 | 1.000305969 | 0.235709496 |
| HBII-85-6;ENSG00000207442;SNORD116-6_chr15_25310171_25310269 | 0.99685655 | 0.991659552 | 1.002080785 | 0.237782373 |
| U34;ENSG00000202503;SNORD34_chr19_49994161_49994231 | 1.00017849 | 0.999880313 | 1.000476756 | 0.240727313 |
| HBII-85-25;ENSG00000252326;SNORD116-25_chr15_25342808_25342902 | 0.999628427 | 0.999007922 | 1.000249317 | 0.240759366 |
| HBII-85-8;ENSG00000207093;SNORD116-8_chr15_25315577_25315674 | 0.997462262 | 0.993223078 | 1.00171954 | 0.242271754 |
| 14q(II-13);ENSG00000201247;SNORD114-13_chr14_101436215_101436289 | 1.002132882 | 0.998552423 | 1.005726178 | 0.243328755 |
| ENSG00000212149;SNORA40_chr17_41092592_41092719 | 0.930444519 | 0.824292083 | 1.05026728 | 0.243437567 |
| HBII-85-7;ENSG00000207133;SNORD116-7_chr15_25312933_25313030 | 0.988689177 | 0.9698683 | 1.007875284 | 0.24604317 |
| HBII-52-40;ENSG00000272460;SNORD115-40_chr15_25488760_25488842 | 0.948956905 | 0.86842312 | 1.036959044 | 0.246911106 |
| ENSG00000201847;SNORD31_chr13_107973243_107973311 | 1.008435595 | 0.993970863 | 1.023110825 | 0.25446359 |
| U106;ENSG00000209042;SNORD12C_chr20_47895477_47895565 | 0.999492805 | 0.998615262 | 1.000371119 | 0.257626554 |
| U62B_chr9_134365872_134365958 | 1.000771157 | 0.999431576 | 1.002112534 | 0.259331425 |
| ENSG00000253051;SNORA31_chr13_45910449_45910582 | 1.007392211 | 0.994532553 | 1.020418149 | 0.261190664 |
| U24;ENSG00000206611;SNORD24_chr9_136216250_136216325 | 0.999614135 | 0.998940241 | 1.000288484 | 0.262006607 |
| 14q(II-20);ENSG00000202048;SNORD114-20_chr14_101447340_101447412 | 1.000614708 | 0.999539628 | 1.001690945 | 0.262539869 |
| HBI-43;ENSG00000212232;SNORD17_chr20_17943352_17943589 | 1.004944054 | 0.99629692 | 1.013666239 | 0.263333262 |
| ENSG00000239027;snoU13_chr1_19858664_19858768 | 0.967381339 | 0.912526759 | 1.025533385 | 0.26552053 |
| ENSG00000201398;U8_chr3_153725156_153725291 | 1.046548834 | 0.965834284 | 1.134008681 | 0.266544759 |
| 14q(II-28);ENSG00000200480;SNORD114-28_chr14_101455466_101455538 | 1.000349243 | 0.999732184 | 1.000966682 | 0.267365185 |
| 14q(0)_chr14_101364256_101364333 | 1.008056574 | 0.993859301 | 1.022456655 | 0.267510684 |
| U42A;ENSG00000238649;SNORD42A_chr17_27050447_27050510 | 0.999874799 | 0.999652934 | 1.000096714 | 0.268799581 |
| HBII-85-27;ENSG00000251896;SNORD116-27_chr15_25346720_25346814 | 0.998177388 | 0.994948797 | 1.001416455 | 0.269746686 |
| SNORD127;ENSG00000239043;SNORD127_chr14_45580085_45580171 | 1.000480698 | 0.999624922 | 1.001337207 | 0.271016267 |
| U58A;ENSG00000206602;SNORD58A_chr18_47017652_47017717 | 1.00036828 | 0.999709924 | 1.00102707 | 0.272976946 |
| ACA5c;ENSG00000201772;SNORA5C_chr7_45144504_45144641 | 0.999371914 | 0.998240205 | 1.000504906 | 0.277125042 |
| HBII-52-42;ENSG00000201143;SNORD115-42_chr15_25492491_25492573 | 0.9549828 | 0.878138152 | 1.038552015 | 0.281848533 |
| HBI-115;ENSG00000238961;SNORA47_chr5_76376258_76376396 | 0.990027415 | 0.972004915 | 1.008384081 | 0.284956975 |
| HBII-85-19;ENSG00000207460;SNORD116-19_chr15_25331672_25331766 | 0.998727204 | 0.996390304 | 1.001069585 | 0.286618504 |
| HBII-52-18;ENSG00000200163;SNORD115-18_chr15_25448373_25448455 | 0.952599451 | 0.871189472 | 1.041616942 | 0.286695876 |
| U89;ENSG00000238795;SCARNA12_chr12_7076499_7076769 | 0.965942729 | 0.905891133 | 1.029975151 | 0.290012649 |
| HBII-234;ENSG00000212534;SNORD70_chr2_203141153_203141241 | 0.992558542 | 0.978861237 | 1.006447515 | 0.292112463 |
| ACA56;ENSG00000206693;SNORA56_chrX_154003272_154003401 | 0.996666126 | 0.990464912 | 1.002906165 | 0.294328041 |
| U70B;ENSG00000206937;SNORA70B_chr2_61644378_61644513 | 0.982804587 | 0.951269208 | 1.015385391 | 0.297233205 |
| ACA40;ENSG00000210825;SNORA40_chr11_93468275_93468402 | 1.039819299 | 0.965508721 | 1.119849207 | 0.302003815 |
| SNORA36C;ENSG00000207016;SNORA36C_chr2_69747174_69747306 | 0.995512686 | 0.987044557 | 1.004053465 | 0.302142529 |
| HBII-85-20_chr15_25332807_25332901 | 0.998851044 | 0.996666041 | 1.001040836 | 0.303525125 |
| U48;ENSG00000201823;SNORD48_chr6_31803039_31803103 | 0.999966995 | 0.999903908 | 1.000030087 | 0.305209735 |
| 14q(II-31);ENSG00000200089;SNORD114-31_chr14_101459572_101459647 | 1.022116863 | 0.979728891 | 1.06633875 | 0.311398533 |
| U18A;ENSG00000200623;SNORD18A_chr15_66795581_66795652 | 1.000111864 | 0.999894012 | 1.000329763 | 0.31424164 |
| HBII-85-21_chr15_25333949_25334043 | 0.99883923 | 0.996580743 | 1.001102835 | 0.314599346 |
| HBII-99;ENSG00000212304;SNORD12_chr20_47897219_47897309 | 1.000406104 | 0.999609321 | 1.001203523 | 0.317911251 |
| U18B;ENSG00000202529;SNORD18B_chr15_66794358_66794429 | 0.999792033 | 0.999381609 | 1.000202625 | 0.320791432 |
| HBII-85-15;ENSG00000207174;SNORD116-15_chr15_25326432_25326526 | 0.990285163 | 0.971370656 | 1.009567972 | 0.321114601 |
| HBII-95B;ENSG00000271852;SNORD11B_chr2_203156054_203156144 | 0.991269331 | 0.974220312 | 1.008616711 | 0.321846905 |
| HBII-52-17;ENSG00000201482;SNORD115-17_chr15_25446469_25446551 | 0.950937743 | 0.860714333 | 1.05061872 | 0.322614542 |
| ENSG00000207098;SNORA70_chr21_34214171_34214305 | 0.983585148 | 0.951785562 | 1.016447172 | 0.323608552 |
| HBII-85-17;ENSG00000206656;SNORD116-17_chr15_25328733_25328827 | 0.998889507 | 0.996686414 | 1.001097469 | 0.323984048 |
| HBII-13_chr15_25230246_25230313 | 0.998963576 | 0.996898606 | 1.001032824 | 0.326004555 |
| ACA50;ENSG00000206952;SNORA50_chr16_58593699_58593835 | 0.997856906 | 0.993553483 | 1.002178969 | 0.33059976 |
| ACA55;ENSG00000201457;SNORA55_chr1_40033045_40033182 | 1.008411904 | 0.99153213 | 1.025579039 | 0.330753489 |
| ENSG00000252792;U3_chr14_68212084_68212191 | 0.99579422 | 0.987345583 | 1.00431515 | 0.332300422 |
| U33;ENSG00000199631;SNORD33_chr19_49993872_49993956 | 1.000360321 | 0.999630121 | 1.001091053 | 0.333556088 |
| HBII-85-16;ENSG00000207263;SNORD116-16_chr15_25327913_25328007 | 0.999033008 | 0.99705348 | 1.001016467 | 0.339060763 |
| 14q(II-29);ENSG00000201689;SNORD114-29_chr14_101456427_101456497 | 1.000984609 | 0.998965508 | 1.003007791 | 0.339437802 |
| U72;ENSG00000207067;SNORA72_chr8_99054313_99054445 | 1.015597374 | 0.983812012 | 1.04840967 | 0.340090245 |
| U21;ENSG00000206680;SNORD21_chr1_93302845_93302940 | 0.999848223 | 0.999535441 | 1.000161102 | 0.341679242 |
| U108;ENSG00000212464;SNORA12_chr10_101996912_101997059 | 0.9460673 | 0.843652102 | 1.060915197 | 0.342917086 |
| U62A_chr9_134361051_134361137 | 1.000690099 | 0.999263192 | 1.002119044 | 0.343356571 |
| ACA66;ENSG00000252577;SCARNA20_chr17_58308876_58309007 | 1.034488389 | 0.964364549 | 1.109711289 | 0.34375622 |
| U84;ENSG00000265236;SNORD84_chr6_31508877_31508955 | 0.999936055 | 0.999803606 | 1.000068522 | 0.344068264 |
| ACA24_chr4_119200344_119200475 | 0.999875595 | 0.999617622 | 1.000133635 | 0.344662874 |
| U80_chr1_173833966_173834044 | 1.000954193 | 0.998972083 | 1.002940236 | 0.345656781 |
| ACA46;ENSG00000207493;SNORA46_chr16_58582402_58582537 | 0.993582938 | 0.980282053 | 1.007064296 | 0.349155622 |
| U29_chr11_62621375_62621440 | 1.000084513 | 0.999907304 | 1.000261753 | 0.34995044 |
| U57_chr20_2637584_2637656 | 0.999934887 | 0.999796798 | 1.000072995 | 0.35543866 |
| U73a;ENSG00000208797;SNORD73A_chr4_152024978_152025043 | 0.998141957 | 0.99421096 | 1.002088496 | 0.355631665 |
| HBII-108;ENSG00000212493;SNORD19_chr3_52723255_52723331 | 1.015342346 | 0.983062554 | 1.048682075 | 0.355660849 |
| ACA1;ENSG00000206834;SNORA1_chr11_93465169_93465299 | 1.006516503 | 0.992683746 | 1.020542016 | 0.357601246 |
| ACA53;ENSG00000212443;SNORA53_chr12_98993412_98993662 | 1.005094035 | 0.994257274 | 1.016048909 | 0.358265983 |
| HBII-85-24;ENSG00000207279;SNORD116-24_chr15_25339182_25339276 | 0.999396517 | 0.998107207 | 1.000687493 | 0.359391959 |
| HBII-251;ENSG00000200181;SNORD85_chr1_31441009_31441084 | 0.999900202 | 0.999686705 | 1.000113744 | 0.359648307 |
| HBII-85-1;ENSG00000207063;SNORD116-1_chr15_25296622_25296719 | 0.997493855 | 0.992150278 | 1.002866211 | 0.359870453 |
| ACA67;ENSG00000200792;SNORA80_chr21_33749495_33749631 | 1.008821498 | 0.989946003 | 1.028056896 | 0.362091412 |
| U88;ENSG00000251791;SCARNA6_chr2_234197321_234197587 | 0.980393534 | 0.939495867 | 1.023071538 | 0.362401811 |
| ACA2b;ENSG00000207313;SNORA2B_chr12_49061239_49061376 | 1.004246675 | 0.99511108 | 1.013466139 | 0.363423746 |
| ENSG00000264346;SNORA77_chr22_20113925_20114049 | 0.989741311 | 0.967930174 | 1.012043935 | 0.364425077 |
| U13;ENSG00000239039;SNORD13_chr8_33370991_33371096 | 0.999915136 | 0.999729994 | 1.000100312 | 0.3690406 |
| HBII-85-14;ENSG00000206621;SNORD116-14_chr15_25325287_25325381 | 0.998908289 | 0.996527092 | 1.001295175 | 0.369704831 |
| 14q(I-6);ENSG00000200215;SNORD113-6_chr14_101405892_101405968 | 1.001655613 | 0.998020529 | 1.005303937 | 0.372505814 |
| ENSG00000252128;SNORD27_chr13_21714025_21714096 | 0.991902058 | 0.974287406 | 1.009835175 | 0.373788459 |
| ENSG00000221750;SNORA11_chrX_54953738_54953866 | 1.071495696 | 0.918782632 | 1.249591563 | 0.37873091 |
| U31_chr11_62620796_62620867 | 0.999975898 | 0.999922104 | 1.000029696 | 0.379892632 |
| U74_chr1_173836811_173836883 | 0.999806515 | 0.999374145 | 1.000239072 | 0.38059091 |
| U90;ENSG00000238741;SCARNA7_chr3_160232694_160233024 | 0.987296038 | 0.959113747 | 1.016306428 | 0.386883369 |
| ENSG00000201042;SNORA38_chr12_119326239_119326369 | 1.039746309 | 0.950238144 | 1.137685741 | 0.396088046 |
| U36C;ENSG00000252542;SNORD36C_chr9_136217700_136217768 | 0.998838773 | 0.996139115 | 1.001545748 | 0.40010869 |
| U104;ENSG00000199753;SNORD104_chr17_62223437_62223517 | 0.999994502 | 0.999981686 | 1.000007318 | 0.400431334 |
| U19;ENSG00000200959;SNORA74A_chr5_138614468_138614668 | 1.00757262 | 0.989989508 | 1.025468025 | 0.400974419 |
| HBII-85-18;ENSG00000206688;SNORD116-18_chr15_25330530_25330624 | 0.999187318 | 0.997284564 | 1.001093703 | 0.403166563 |
| ENSG00000238536;snoU13_chr4_17530560_17530663 | 1.02235374 | 0.970466187 | 1.077015545 | 0.405471429 |
| ACA51;ENSG00000271798;SNORA51_chr20_2635712_2635844 | 1.011215256 | 0.984821232 | 1.038316662 | 0.408522771 |
| HBII-85-2;ENSG00000207001;SNORD116-2_chr15_25299355_25299452 | 0.997823433 | 0.992667818 | 1.003005824 | 0.409707905 |
| SNORD124;ENSG00000238793;SNORD124_chr17_38183794_38183898 | 0.992498395 | 0.974883238 | 1.01043184 | 0.409864498 |
| U38B;ENSG00000207421;SNORD38B_chr1_45244061_45244130 | 0.999573085 | 0.998554424 | 1.000592786 | 0.411751957 |
| ACA26;ENSG00000252808;SCARNA4_chr1_155895748_155895877 | 0.999681459 | 0.998920094 | 1.000443405 | 0.412461775 |
| U45A;ENSG00000207241;SNORD45A_chr1_76253573_76253657 | 1.000119611 | 0.99983107 | 1.000408236 | 0.416555855 |
| ACA67B;ENSG00000206633;SNORA80B_chr2_10586839_10586975 | 1.003819475 | 0.994619043 | 1.013105012 | 0.417095064 |
| 14q(I-4);ENSG00000201672;SNORD113-4_chr14_101402827_101402902 | 1.016844685 | 0.97657385 | 1.058776164 | 0.417819708 |
| ENSG00000200355;SNORA72_chr3_172489067_172489195 | 0.966892045 | 0.891109351 | 1.049119533 | 0.418807182 |
| ACA12;ENSG00000251869;SCARNA23_chrX_24762557_24762687 | 0.949574917 | 0.837584335 | 1.076539383 | 0.419034226 |
| ACA45_chr15_83424696_83424823 | 0.999893266 | 0.999633169 | 1.000153431 | 0.421313103 |
| HBII-85-22_chr15_25335068_25335162 | 0.999244775 | 0.997404484 | 1.001088462 | 0.4218059 |
| U94;ENSG00000208772;SNORD94_chr2_86362992_86363129 | 0.998411792 | 0.99453768 | 1.002300996 | 0.422959272 |
| ENSG00000201009;SNORD46_chr7_132437783_132437886 | 0.998203436 | 0.993813532 | 1.002612731 | 0.423925734 |
| ENSG00000201129;SNORA58_chr1_154232203_154232338 | 1.001971117 | 0.997063642 | 1.006902746 | 0.431823331 |
| U47_chr1_173833507_173833572 | 1.000721857 | 0.998914597 | 1.002532387 | 0.433964691 |
| SNORD121A;ENSG00000238886;SNORD121A_chr9_33952762_33952852 | 0.978890393 | 0.927914035 | 1.032667214 | 0.434267406 |
| ENSG00000212168;SNORD78_chr2_57771670_57771734 | 0.992825485 | 0.975044648 | 1.010930572 | 0.434850458 |
| HBII-52-15;ENSG00000201679;SNORD115-15_chr15_25442722_25442803 | 0.97608954 | 0.918211746 | 1.037615555 | 0.43775873 |
| ENSG00000199282;SNORA9_chr13_73160818_73160948 | 1.04647157 | 0.931882888 | 1.175150612 | 0.442677143 |
| ENSG00000200879;SNORD14E_chr11_122928785_122928869 | 1.00349719 | 0.994489559 | 1.012586409 | 0.447940008 |
| ACA62_chr17_62223698_62223831 | 1.002395566 | 0.996172604 | 1.008657403 | 0.451416598 |
| ENSG00000239128;snoU13_chr3_47292013_47292116 | 0.974847086 | 0.912302237 | 1.041679834 | 0.45146341 |
| ENSG00000201882;snoU2-30_chrX_20154184_20154253 | 0.997988825 | 0.992722005 | 1.003283588 | 0.455849015 |
| U28_chr11_62622092_62622167 | 1.000059089 | 0.999903432 | 1.000214769 | 0.456885972 |
| U97;ENSG00000238622;SNORD97_chr11_10823013_10823155 | 1.001869226 | 0.996870056 | 1.006893467 | 0.464353071 |
| U103B;ENSG00000202107;SNORD103B_chr1_31421961_31422052 | 1.001096124 | 0.998139988 | 1.004061015 | 0.467795112 |
| 14q(II-19);ENSG00000199942;SNORD114-19_chr14_101442813_101442888 | 1.001162195 | 0.998025145 | 1.004309106 | 0.468207587 |
| ENSG00000206603;SNORA22_chr7_56123058_56123195 | 1.040105503 | 0.933535309 | 1.158841501 | 0.475870545 |
| U67_chr17_7481272_7481409 | 1.00719371 | 0.987426603 | 1.02735653 | 0.478455903 |
| U45B;ENSG00000201487;SNORD45B_chr1_76255161_76255233 | 1.001724207 | 0.996951422 | 1.006519841 | 0.479583183 |
| ACA22;ENSG00000206634;SNORA22_chr7_65220512_65220646 | 0.962694654 | 0.866301817 | 1.06981306 | 0.480006602 |
| U20;ENSG00000207280;SNORD20_chr2_232321154_232321234 | 0.999944086 | 0.999788291 | 1.000099904 | 0.481833815 |
| U79_chr1_173834485_173834570 | 1.00024644 | 0.999555884 | 1.000937473 | 0.484363616 |
| ENSG00000201541;SNORA1_chr16_24344736_24344868 | 1.051611509 | 0.912715971 | 1.211643929 | 0.486245992 |
| 14q(II-24);ENSG00000201899;SNORD114-24_chr14_101451113_101451185 | 1.000544127 | 0.998995249 | 1.002095406 | 0.491328785 |
| U36A;ENSG00000199744;SNORD36A_chr9_136217310_136217383 | 1.002314396 | 0.995709805 | 1.008962796 | 0.493128548 |
| ENSG00000212378;SNORD78_chr2_72987663_72987731 | 1.010578024 | 0.980211209 | 1.041885599 | 0.499059054 |
| HBII-180B;ENSG00000221381;SNORD88B_chr19_51302285_51302382 | 1.015377658 | 0.971338142 | 1.061413883 | 0.499963493 |
| ENSG00000206761;U3_chr14_101611818_101612032 | 0.985880471 | 0.945915425 | 1.027534046 | 0.500625452 |
| U64;ENSG00000207405;SNORA64_chr16_2012973_2013107 | 1.003010829 | 0.994232629 | 1.011866533 | 0.502661878 |
| 14q(II-30);ENSG00000201318;SNORD114-30_chr14_101458255_101458327 | 1.003962177 | 0.99241855 | 1.015640078 | 0.502745197 |
| ENSG00000223213;SNORD81_chr12_54185089_54185131 | 0.985467228 | 0.944068991 | 1.028680814 | 0.50377067 |
| HBII-436_chr15_25227140_25227215 | 0.999617955 | 0.998496073 | 1.000741098 | 0.504809151 |
| U46;ENSG00000200913;SNORD46_chr1_45242162_45242265 | 0.998789324 | 0.995234963 | 1.002356379 | 0.50540794 |
| U100;ENSG00000252712;SCARNA14_chr15_66639543_66639680 | 1.003525147 | 0.993180079 | 1.013977971 | 0.505672306 |
| ACA18;ENSG00000207145;SNORA18_chr11_93466631_93466763 | 1.002489278 | 0.995164473 | 1.009867997 | 0.506391128 |
| snR38B;ENSG00000199961;SNORD1B_chr17_74557189_74557275 | 0.999967182 | 0.999870285 | 1.000064088 | 0.506829059 |
| ENSG00000212277;SNORD43_chr11_74427732_74427794 | 1.02639946 | 0.949612076 | 1.109396013 | 0.511318912 |
| ENSG00000252175;U3_chrX_54091278_54091399 | 0.992298502 | 0.969661267 | 1.015464216 | 0.511421036 |
| U27_chr11_62622483_62622555 | 0.999973936 | 0.999894776 | 1.000053103 | 0.518739624 |
| ENSG00000251944;U3_chr8_20472340_20472437 | 1.001574818 | 0.99679341 | 1.006379161 | 0.519248679 |
| ENSG00000207407;SNORA1_chr11_19612703_19612838 | 1.007687631 | 0.984095586 | 1.031845256 | 0.526353708 |
| ENSG00000222937;SNORD63_chr5_137894659_137894728 | 0.979530122 | 0.918723914 | 1.044360819 | 0.527046904 |
| mgU6-47;ENSG00000207297;SNORD7_chr17_33900675_33900772 | 1.000496924 | 0.998955827 | 1.002040398 | 0.527611287 |
| ENSG00000238484;snoU13_chr6_34660557_34660660 | 1.020465783 | 0.958060478 | 1.086935991 | 0.529192212 |
| ACA28;ENSG00000272533;SNORA28_chr14_103804185_103804311 | 0.996934094 | 0.987429029 | 1.006530654 | 0.529864952 |
| U65;ENSG00000201302;SNORA65_chr9_130210779_130210916 | 1.001460234 | 0.996885219 | 1.006056245 | 0.532234634 |
| ENSG00000207084;SNORA72_chr3_160414717_160414848 | 0.959002051 | 0.840336375 | 1.094424758 | 0.534501714 |
| ENSG00000212421;SNORA26_chr9_89875365_89875500 | 0.962221459 | 0.85191506 | 1.086810386 | 0.535313776 |
| mgU2-19/30_chr11_93454679_93455032 | 1.005368605 | 0.988485047 | 1.022540538 | 0.535498727 |
| ACA35;ENSG00000252947;SCARNA1_chr1_28160911_28161077 | 0.980375851 | 0.920083052 | 1.044619621 | 0.54053648 |
| ENSG00000221611;SNORD88_chr12_13124987_13125077 | 0.972060864 | 0.885961191 | 1.066527893 | 0.549282138 |
| ENSG00000252433;SNORA31_chr1_67568328_67568462 | 1.007226455 | 0.983561588 | 1.031460707 | 0.552792945 |
| U41;ENSG00000209702;SNORD41_chr19_12817262_12817332 | 0.999786455 | 0.999081609 | 1.000491799 | 0.552826499 |
| U75_chr1_173836016_173836076 | 1.000304046 | 0.99929963 | 1.001309473 | 0.553119714 |
| ENSG00000201229;SNORA63_chr3_183169645_183169776 | 0.964159192 | 0.852582456 | 1.09033788 | 0.560795003 |
| ENSG00000212598;U3_chr3_90079434_90079644 | 1.02676833 | 0.938807728 | 1.122970309 | 0.563197461 |
| ENSG00000238754;snoU109_chr1_193026411_193026545 | 1.036983645 | 0.915200531 | 1.174972088 | 0.568844566 |
| ENSG00000252337;SNORA31_chr5_105882366_105882504 | 0.976087407 | 0.89784186 | 1.061151935 | 0.57022814 |
| mgU6-77;ENSG00000238917;SNORD10_chr17_7480128_7480276 | 0.999903907 | 0.999571853 | 1.000236072 | 0.57066753 |
| U25_chr11_62623036_62623103 | 0.999982717 | 0.999922113 | 1.000043326 | 0.576228568 |
| U91;ENSG00000252139;SCARNA18_chr18_47340730_47340813 | 0.996092737 | 0.982506168 | 1.009867186 | 0.576363738 |
| HBII-85-26;ENSG00000251815;SNORD116-26_chr15_25344644_25344742 | 0.998948077 | 0.995234421 | 1.002675591 | 0.579680644 |
| HBII-142;ENSG00000212158;SNORD66_chr3_184043483_184043559 | 0.999992755 | 0.99996703 | 1.000018481 | 0.580980212 |
| HBII-85-13;ENSG00000207137;SNORD116-13_chr15_25324203_25324297 | 1.013537783 | 0.966273691 | 1.06311374 | 0.581023842 |
| ENSG00000238326;snoU13_chr5_55296346_55296449 | 0.994359002 | 0.97455966 | 1.014560592 | 0.581448717 |
| U45C;ENSG00000206620;SNORD45C_chr1_76252756_76252835 | 1.00010445 | 0.999732776 | 1.000476262 | 0.581820752 |
| U87;ENSG00000252010;SCARNA5_chr2_234184371_234184649 | 0.998620713 | 0.993654007 | 1.003612245 | 0.587428956 |
| U107;ENSG00000221716;SNORA11_chrX_54840802_54840933 | 0.986016141 | 0.936955565 | 1.037645611 | 0.588637845 |
| ENSG00000206947;SNORA20_chr7_39368602_39368733 | 1.001490677 | 0.996076684 | 1.006934097 | 0.590167551 |
| HBII-180C;ENSG00000220988;SNORD88C_chr19_51305581_51305678 | 0.999520613 | 0.997747843 | 1.001296533 | 0.596521134 |
| ENSG00000238295;snoU13_chr2_178210586_178210689 | 0.987992715 | 0.944664777 | 1.033307929 | 0.597530053 |
| ACA25;ENSG00000207112;SNORA25_chr11_93463678_93463812 | 1.000422267 | 0.998835697 | 1.002011357 | 0.602128594 |
| U58B;ENSG00000271982;SNORD58B_chr18_47018033_47018099 | 1.000174554 | 0.999514994 | 1.00083455 | 0.604051149 |
| ENSG00000201592;snoU2_19_chrX_20154424_20154503 | 0.998724959 | 0.993842048 | 1.003631861 | 0.609901452 |
| ENSG00000207130;SNORA24_chr3_128433414_128433548 | 0.999056998 | 0.995397086 | 1.002730367 | 0.614377062 |
| SNORD119;ENSG00000251806;SNORD119_chr20_2443604_2443686 | 0.999907457 | 0.999536519 | 1.000278532 | 0.624936188 |
| ACA11_chr4_1976362_1976487 | 0.98781662 | 0.939088389 | 1.039073304 | 0.634834965 |
| ACA10;ENSG00000206811;SNORA10_chr16_2012334_2012467 | 1.002640465 | 0.991532763 | 1.013872603 | 0.642692152 |
| U15A;ENSG00000206941;SNORD15A_chr11_75111434_75111582 | 1.001785126 | 0.994265166 | 1.009361961 | 0.642697143 |
| HBII-99B;ENSG00000222365;SNORD12B_chr20_47896855_47896946 | 0.99987151 | 0.999325466 | 1.000417853 | 0.644767432 |
| ENSG00000252921;U3_chr18_23879079_23879219 | 1.018886029 | 0.940661722 | 1.103615375 | 0.646188482 |
| ACA52;ENSG00000199785;SNORA52_chr11_811680_811814 | 1.005003144 | 0.983725072 | 1.026741462 | 0.647605021 |
| U17b;ENSG00000200087;SNORA73B_chr1_28835069_28835274 | 1.000240937 | 0.999199916 | 1.001283043 | 0.650232883 |
| mgU2-25/61_chr1_109642814_109643234 | 1.001832644 | 0.993924416 | 1.009803795 | 0.650678956 |
| U68;ENSG00000207166;SNORA68_chr19_17973396_17973529 | 0.997258638 | 0.98505024 | 1.009618344 | 0.66225283 |
| ENSG00000199769;U3_chrX_70065931_70066145 | 1.000663054 | 0.997678094 | 1.003656944 | 0.663660927 |
| ENSG00000222345;SNORD19_chr3_52725394_52725469 | 0.999997401 | 0.999985548 | 1.000009253 | 0.667288379 |
| U26_chr11_62622763_62622838 | 0.999854601 | 0.999184486 | 1.000525166 | 0.6707697 |
| ACA65;ENSG00000221303;SNORA79_chr14_81669038_81669178 | 0.990686315 | 0.948843224 | 1.034374647 | 0.670846459 |
| U32A;ENSG00000201675;SNORD32A_chr19_49993222_49993305 | 1.00007041 | 0.999744482 | 1.000396444 | 0.672034879 |
| ENSG00000199787;SNORA42_chr16_30430946_30431080 | 1.016842418 | 0.941143683 | 1.098629808 | 0.67218527 |
| ACA3;ENSG00000200983;SNORA3_chr11_8705773_8705903 | 0.999484732 | 0.997085053 | 1.001890187 | 0.674312511 |
| ENSG00000199411;SNORD62_chr9_139988797_139988882 | 1.007674346 | 0.972138963 | 1.044508681 | 0.676412245 |
| ENSG00000200652;SNORA25_chr16_28190419_28190544 | 0.981354969 | 0.898156479 | 1.072260344 | 0.677119686 |
| ENSG00000238503;SNORD18_chr2_12170429_12170498 | 1.000820229 | 0.99696214 | 1.004693248 | 0.67736987 |
| Z17B;ENSG00000238597;SNORD4B_chr17_27050698_27050772 | 0.996745414 | 0.981483914 | 1.012244222 | 0.678809923 |
| ENSG00000206903;SNORA24_chr15_65577799_65577929 | 0.999676373 | 0.998145548 | 1.001209546 | 0.678899262 |
| ENSG00000200620;SNORA7_chrX_15734331_15734469 | 1.000424135 | 0.998398881 | 1.002453498 | 0.681708022 |
| ACA27;ENSG00000207051;SNORA27_chr13_27829537_27829663 | 1.006305365 | 0.976482627 | 1.03703892 | 0.682169423 |
| SNORA38B;ENSG00000200394;SNORA38B_chr17_65736784_65736915 | 1.006332125 | 0.97535978 | 1.03828799 | 0.692288168 |
| U3-4_chr17_19015732_19015949 | 1.000038133 | 0.999848274 | 1.000228028 | 0.693855815 |
| ACA2a;ENSG00000206612;SNORA2A_chr12_49050430_49050565 | 1.00648228 | 0.97454854 | 1.039462415 | 0.694484309 |
| HBII-382_chr1_109643154_109643236 | 1.000327178 | 0.998635613 | 1.002021608 | 0.704812598 |
| E3;ENSG00000200320;SNORA63_chr3_186505087_186505222 | 1.001240707 | 0.994790998 | 1.007732232 | 0.706880916 |
| ENSG00000238327;snoU13_chrX_23525328_23525430 | 1.000527301 | 0.997765933 | 1.003296311 | 0.708516318 |
| ENSG00000253007;SNORA76_chr22_34100772_34100906 | 0.979403274 | 0.87729159 | 1.09340017 | 0.711031504 |
| hTR_chr3_169482397_169482945 | 0.990031595 | 0.938856351 | 1.043996303 | 0.711407346 |
| U53;ENSG00000265145;SNORD53_chr2_29149932_29150010 | 0.998387192 | 0.989876994 | 1.006970553 | 0.711711518 |
| HBI-6;ENSG00000212588;SNORA26_chr4_53579415_53579537 | 1.002156108 | 0.99071992 | 1.013724307 | 0.713020248 |
| U109;ENSG00000238835;SCARNA18_chr5_82360022_82360156 | 0.992380053 | 0.952565675 | 1.033858553 | 0.714267125 |
| U71a;ENSG00000225091;SNORA71A_chr20_37055948_37056086 | 1.003733538 | 0.983692998 | 1.02418236 | 0.717235162 |
| ENSG00000252543;U3_chr8_8916049_8916140 | 0.999108396 | 0.994269748 | 1.003970591 | 0.718756493 |
| ACA49;ENSG00000208892;SNORA49_chr12_132515768_132515905 | 0.991136361 | 0.943871829 | 1.040767671 | 0.720996488 |
| U101;ENSG00000206754;SNORD101_chr6_133136445_133136518 | 0.999934534 | 0.999572671 | 1.000296528 | 0.722958832 |
| snR39B;ENSG00000238942;SNORD2_chr3_186502584_186502654 | 1.000014987 | 0.999932063 | 1.000097918 | 0.723169703 |
| HBII-276_chr8_67834708_67834784 | 0.999749754 | 0.998314978 | 1.001186591 | 0.732683017 |
| ACA20;ENSG00000207392;SNORA20_chr6_160201281_160201413 | 1.000375699 | 0.998212396 | 1.002543691 | 0.733795568 |
| U102;ENSG00000207500;SNORD102_chr13_27829200_27829272 | 0.999942885 | 0.999612826 | 1.000273054 | 0.734538283 |
| U16;ENSG00000199673;SNORD16_chr15_66795148_66795249 | 1.001096856 | 0.994733686 | 1.007500731 | 0.736147089 |
| U15B;ENSG00000207445;SNORD15B_chr11_75115464_75115610 | 1.00033854 | 0.998362513 | 1.002318479 | 0.737238129 |
| ENSG00000238708;snoU13_chr2_69894491_69894593 | 0.985991844 | 0.907881094 | 1.070822957 | 0.737620677 |
| ACA64_chr16_2015184_2015311 | 1.016234772 | 0.924639446 | 1.116903584 | 0.738253335 |
| ENSG00000200418;SNORA63_chr3_186504112_186504234 | 0.983283141 | 0.890105336 | 1.086214963 | 0.739977494 |
| U83;ENSG00000201785;SNORD117_chr6_31504150_31504226 | 1.000562903 | 0.997183728 | 1.00395353 | 0.744400912 |
| HBII-240;ENSG00000212296;SNORD72_chr5_40832757_40832837 | 1.000732997 | 0.996265551 | 1.005220475 | 0.748225086 |
| U35B;ENSG00000200530;SNORD35B_chr19_50000975_50001063 | 1.001156984 | 0.99405783 | 1.008306838 | 0.750124475 |
| SNORA84;ENSG00000239183;SNORA84_chr9_95054742_95054875 | 0.998510687 | 0.989316108 | 1.00779072 | 0.752176883 |
| HBII-82B;ENSG00000221514;SNORD111B_chr16_70563411_70563498 | 0.99961331 | 0.997203809 | 1.002028633 | 0.7534408 |
| ACA37;ENSG00000207233;SNORA37_chr18_51748653_51748782 | 1.011318275 | 0.941690828 | 1.086093888 | 0.757141064 |
| ENSG00000207215;U3_chr8_98370493_98370702 | 1.000090449 | 0.999516681 | 1.000664546 | 0.757402118 |
| U71b;ENSG00000235408;SNORA71B_chr20_37053731_37054002 | 0.999823978 | 0.998688476 | 1.000960771 | 0.761411381 |
| HBII-202;ENSG00000200084;SNORD68_chr16_89627837_89627925 | 0.999649255 | 0.997354379 | 1.001949411 | 0.764816877 |
| ENSG00000265706;SNORD53_SNORD92_chr2_29150849_29150926 | 0.999251202 | 0.994325881 | 1.00420092 | 0.766369113 |
| ACA59;ENSG00000239149;SNORA59A_chr1_12567299_12567451 | 0.981378866 | 0.865859832 | 1.112309918 | 0.768626212 |
| ENSG00000252277;SNORD116-30_chr15_25353415_25353499 | 0.988903953 | 0.917119595 | 1.066306984 | 0.771661896 |
| HBII-85-11;ENSG00000206609;SNORD116-11_chr15_25321074_25321168 | 0.981487195 | 0.864602622 | 1.114173252 | 0.772704515 |
| ENSG00000252299;U3_chr9_90989184_90989274 | 0.999980541 | 0.999847897 | 1.000113202 | 0.773723793 |
| ENSG00000238852;snoU13_chr2_223562615_223562719 | 1.014149141 | 0.915966967 | 1.122855426 | 0.786822478 |
| ACA33;ENSG00000200534;SNORA33_chr6_133138357_133138490 | 0.997997168 | 0.983414801 | 1.012795767 | 0.789504414 |
| ACA19;ENSG00000207468;SNORA19_chr10_120819522_120819650 | 1.001137131 | 0.992659841 | 1.009686817 | 0.793367096 |
| ACA4;ENSG00000263776;SNORA4_chr3_186505401_186505538 | 0.995189005 | 0.958965107 | 1.032781222 | 0.798780447 |
| ACA7;ENSG00000207496;SNORA7A_chr3_12881810_12881949 | 0.999836387 | 0.998557496 | 1.001116917 | 0.802151695 |
| U78;ENSG00000208317;SNORD78_chr1_173834759_173834824 | 1.000020284 | 0.999861366 | 1.000179227 | 0.802471401 |
| U36B;ENSG00000200831;SNORD36B_chr9_136216948_136217023 | 1.001223692 | 0.991670705 | 1.010868705 | 0.802575602 |
| ENSG00000252529;RNU3P3_chr14_85738276_85738405 | 0.999976209 | 0.999789624 | 1.000162828 | 0.802673392 |
| HBII-82;ENSG00000221066;SNORD111_chr16_70571907_70572001 | 0.999950228 | 0.999553035 | 1.000347579 | 0.806034741 |
| mgh28S-2411;ENSG00000202314;SNORD6_chr11_93464668_93464740 | 0.999946743 | 0.999517132 | 1.000376539 | 0.808076499 |
| mgU12-22/U4-8;ENSG00000251992;SCARNA17_chr18_47340392_47340813 | 0.992902637 | 0.935797322 | 1.0534927 | 0.813681288 |
| mgU12-22/U4-8;ENSG00000252139;SCARNA18_chr18_47340392_47340813 | 0.992902637 | 0.935797322 | 1.0534927 | 0.813681288 |
| ACA38;ENSG00000200816;SNORA38_chr6_31590855_31590987 | 0.995436727 | 0.957239806 | 1.035157827 | 0.818787592 |
| U37;ENSG00000206775;SNORD37_chr19_3982504_3982570 | 0.999907035 | 0.99909067 | 1.000724067 | 0.823463315 |
| U38A;ENSG00000202031;SNORD38A_chr1_45243513_45243584 | 0.99998175 | 0.999820791 | 1.000142734 | 0.824149914 |
| ACA47_chr17_75085388_75085575 | 1.002835164 | 0.978080868 | 1.028215968 | 0.824305412 |
| HBII-85-4_chr15_25304683_25304781 | 1.003362202 | 0.973727519 | 1.033898794 | 0.826311033 |
| ACA9_chr7_45024976_45025109 | 1.001008682 | 0.992030151 | 1.010068474 | 0.826407617 |
| ENSG00000202482;U3_chrX_71945976_71946190 | 1.0100919 | 0.922407183 | 1.106111992 | 0.828423898 |
| ENSG00000252050;SNORA31_chrX_38167365_38167494 | 0.997188392 | 0.971803914 | 1.023235938 | 0.830538593 |
| HBII-429;ENSG00000221500;SNORD100_chr6_133137940_133138016 | 0.999867506 | 0.998636286 | 1.001100245 | 0.833066043 |
| U81_chr1_173833283_173833360 | 0.999946551 | 0.999444557 | 1.000448798 | 0.834741101 |
| ENSG00000238520;snoU13_chr2_115038661_115038752 | 1.011537411 | 0.908094177 | 1.126764118 | 0.834905309 |
| U35A;ENSG00000200259;SNORD35A_chr19_49994431_49994517 | 1.001116034 | 0.990575879 | 1.011768342 | 0.83636159 |
| U58C;ENSG00000202093;SNORD58C_chr18_47015613_47015678 | 1.000023593 | 0.999793595 | 1.000253644 | 0.840675881 |
| mgU6-53B;ENSG00000199436;SNORD9_chr14_21860309_21860412 | 0.999191577 | 0.991243358 | 1.007203528 | 0.842672522 |
| ACA5;ENSG00000206838;SNORA5A_chr7_45143947_45144081 | 1.00272051 | 0.975510499 | 1.030689491 | 0.846526153 |
| ENSG00000200677;SNORD18_chr15_91298473_91298542 | 0.995930009 | 0.953999309 | 1.039703671 | 0.852577821 |
| snR38C_chr17_74554871_74554951 | 1.000396609 | 0.996218547 | 1.004592193 | 0.852679466 |
| U3-3;ENSG00000264940;SNORD3C_chr17_19092978_19093558 | 1.000020015 | 0.99980804 | 1.000232036 | 0.853192042 |
| ENSG00000251836;U3_chr10_120545264_120545475 | 1.001478822 | 0.985822531 | 1.017383757 | 0.854159286 |
| ACA58_chr3_131197940_131198077 | 0.999939953 | 0.999293959 | 1.000586364 | 0.855488015 |
| U30_chr11_62621134_62621204 | 1.00000008 | 0.999999217 | 1.000000942 | 0.855827389 |
| U3-2;ENSG00000265185;SNORD3B-1_chr17_18965224_18965982 | 0.999912074 | 0.998962871 | 1.000862179 | 0.856006317 |
| U3-2B;ENSG00000262074;SNORD3B-2_chr17_18966659_18967449 | 1.000026411 | 0.999736459 | 1.000316448 | 0.858324733 |
| U17a_chr1_28833876_28834083 | 0.999854038 | 0.998225732 | 1.001485001 | 0.860658548 |
| mgU6-53;ENSG00000200785;SNORD8_chr14_21865451_21865560 | 0.998270226 | 0.978101558 | 1.018854777 | 0.8679608 |
| ACA14b;ENSG00000207181;SNORA14B_chr1_235291117_235291252 | 1.000697739 | 0.992455163 | 1.009008772 | 0.868719173 |
| HBII-289;ENSG00000212283;SNORD89_chr2_101889397_101889511 | 0.999938869 | 0.999211911 | 1.000666357 | 0.869140799 |
| snR38A_chr17_74557714_74557786 | 1.000116024 | 0.998731284 | 1.001502683 | 0.86963969 |
| ACA41;ENSG00000207406;SNORA41_chr2_207026951_207027083 | 1.001299523 | 0.98478832 | 1.018087558 | 0.878332013 |
| U54;ENSG00000238650;SNORD54_chr8_56986394_56986460 | 0.999943161 | 0.999191642 | 1.000695245 | 0.882203002 |
| U85_chr12_6619387_6619717 | 1.000574716 | 0.992812185 | 1.008397941 | 0.885035995 |
| HBI-61;ENSG00000221420;SNORA81_chr3_186504463_186504641 | 1.000744027 | 0.990589416 | 1.011002735 | 0.886345715 |
| ACA54;ENSG00000207008;SNORA54_chr11_2985000_2985123 | 0.999079144 | 0.986509249 | 1.011809203 | 0.886595246 |
| ENSG00000212144;U8_chr1_234729021_234729148 | 1.000499015 | 0.993504584 | 1.007542688 | 0.889151178 |
| ACA63;ENSG00000221643;SNORA77_chr1_203698708_203698833 | 0.999293483 | 0.989356257 | 1.009330519 | 0.889761031 |
| U96a;ENSG00000272296;SNORD96A_chr5_180668814_180668892 | 1.000057219 | 0.999229513 | 1.00088561 | 0.892264719 |
| ENSG00000200318;U3_chr15_59052666_59052880 | 1.002233922 | 0.969702923 | 1.035856251 | 0.894554617 |
| U3;ENSG00000263934;SNORD3A_chr17_19091328_19092027 | 0.999944049 | 0.999086205 | 1.000802629 | 0.898326199 |
| U86;ENSG00000212498;SNORD86_chr20_2636742_2636828 | 0.998403206 | 0.974093786 | 1.02331929 | 0.898887138 |
| U23;ENSG00000206885;SNORA75_chr2_232320510_232320647 | 1.000538495 | 0.992246041 | 1.008900251 | 0.899112982 |
| ACA34;ENSG00000221491;SNORA34_chr12_49048164_49048301 | 1.00183652 | 0.973343 | 1.031164156 | 0.900811225 |
| ENSG00000207344;SNORA22_chr7_64526377_64526510 | 1.006426238 | 0.909860883 | 1.113240268 | 0.900945481 |
| U42B;ENSG00000238423;SNORD42B_chr17_27047567_27047634 | 0.999947584 | 0.999092595 | 1.000803306 | 0.904402289 |
| ACA7B;ENSG00000207088;SNORA7B_chr3_129116052_129116191 | 1.000065993 | 0.998988984 | 1.001144162 | 0.904456229 |
| ENSG00000221719;SNORA3_chr16_2846409_2846533 | 1.000025167 | 0.999607371 | 1.000443138 | 0.906034669 |
| mgh28S-2409;ENSG00000239195;SNORD5_chr11_93466393_93466466 | 1.000075878 | 0.998777409 | 1.001376035 | 0.908870604 |
| ACA68;ENSG00000252835;SCARNA21_chr17_7809440_7809578 | 0.991737962 | 0.859390731 | 1.14446683 | 0.909615549 |
| SNORD126;ENSG00000238344;SNORD126_chr14_20794608_20794685 | 0.999849863 | 0.997229584 | 1.002477026 | 0.910706954 |
| ACA42;ENSG00000207475;SNORA42_chr1_155889699_155889836 | 0.998935837 | 0.980007893 | 1.018229357 | 0.91313327 |
| ACA21;ENSG00000199293;SNORA21_chr17_37009115_37009248 | 1.000341601 | 0.993986385 | 1.006737451 | 0.916349157 |
| ACA14a;ENSG00000201643;SNORA14A_chr7_75573100_75573234 | 0.997717602 | 0.953611014 | 1.043864216 | 0.921097831 |
| HBII-420;ENSG00000221539;SNORD99_chr1_28905254_28905334 | 1.000002007 | 0.999961566 | 1.000042451 | 0.922498267 |
| HBII-135_chr17_16344539_16344612 | 1.000485929 | 0.990678139 | 1.010390817 | 0.9230015 |
| ENSG00000206897;SNORA9_chr12_124101255_124101387 | 0.993073562 | 0.861817417 | 1.144320224 | 0.923443726 |
| ENSG00000200706;SNORD45_chr6_38175050_38175121 | 1.000323045 | 0.993526633 | 1.00716595 | 0.926015834 |
| ENSG00000207217;SNORA42_chr7_6056508_6056642 | 1.003090031 | 0.93802048 | 1.072673392 | 0.928158988 |
| HBII-166;ENSG00000212135;SNORD67_chr11_46783938_46784049 | 0.998655694 | 0.969549338 | 1.028635837 | 0.928972838 |
| U93;ENSG00000252481;SCARNA13_chr14_95999691_95999966 | 0.999785915 | 0.995070431 | 1.004523746 | 0.929269823 |
| ENSG00000200496;U8_chr11_123172098_123172231 | 0.999732768 | 0.99362838 | 1.005874657 | 0.931841723 |
| HBII-316;ENSG00000264994;SNORD92_chr2_29136527_29136616 | 0.999937335 | 0.998476098 | 1.001400711 | 0.93306556 |
| ENSG00000199405;SNORA1_chr8_56815282_56815414 | 0.999129591 | 0.978828339 | 1.019851898 | 0.9337403 |
| U49B_chr17_16342822_16342870 | 0.999910548 | 0.997800839 | 1.002024718 | 0.933842591 |
| U71d;ENSG00000200354;SNORA71D_chr20_37062504_37062642 | 1.000217065 | 0.994571853 | 1.005894319 | 0.940088868 |
| U96b;ENSG00000208883;SNORD96B_chrX_109468213_109468291 | 1.00189533 | 0.945273196 | 1.061909146 | 0.949133469 |
| U8;ENSG00000200463;SNORD118_chr17_8076770_8076906 | 1.000044533 | 0.9986104 | 1.001480727 | 0.95150297 |
| HBII-296B;ENSG00000275084;SNORD91B_chr17_2232310_2232531 | 0.999731213 | 0.99022224 | 1.009331499 | 0.956034611 |
| ENSG00000238854;SNORD5_chr8_142457575_142457649 | 0.998278559 | 0.934048533 | 1.066925374 | 0.959503227 |
| U66;ENSG00000207523;SNORA66_chr1_93306275_93306408 | 1.000346034 | 0.985265652 | 1.015657234 | 0.964393364 |
| ENSG00000207199;SNORD38_chr8_38876134_38876202 | 1.000930378 | 0.958643771 | 1.045082283 | 0.966319546 |
| ACA31_chr13_45911614_45911744 | 1.000003295 | 0.999844506 | 1.000162109 | 0.967561778 |
| U82;ENSG00000202400;SNORD82_chr2_232325078_232325153 | 1.000004746 | 0.999775846 | 1.000233697 | 0.967590188 |
| ACA3-2;ENSG00000212607;SNORA45_chr11_8706985_8707116 | 1.000023556 | 0.998849428 | 1.001199064 | 0.968652221 |
| ENSG00000199321;SNORD60_chr10_128468271_128468343 | 0.998499796 | 0.925254951 | 1.077542835 | 0.969190183 |
| ENSG00000252787;SNORD19B_chr3_52722898_52722977 | 0.999997981 | 0.999895327 | 1.000100645 | 0.969251286 |
| ENSG00000200072;SNORD44_chr13_112706392_112706452 | 1.000624897 | 0.966718163 | 1.035720879 | 0.971667097 |
| ENSG00000222489;SNORA79_chr14_20791338_20791485 | 0.998914094 | 0.939660075 | 1.061904613 | 0.972220288 |
| ENSG00000238790;snoU13_chr18_3811317_3811420 | 0.999672115 | 0.980375525 | 1.019348517 | 0.973694078 |
| ENSG00000207118;SNORD14D_chr11_122929617_122929703 | 1.000014768 | 0.99910857 | 1.000921787 | 0.974530709 |
| ENSG00000200969;SNORD95_chr9_84503833_84503900 | 0.998919082 | 0.930707934 | 1.072129394 | 0.976091209 |
| ENSG00000221461;U3_chr8_124192551_124192765 | 1.000001496 | 0.999894894 | 1.000108109 | 0.978060965 |
| U55;ENSG00000264294;SNORD55_chr1_45241536_45241615 | 0.999968912 | 0.997596241 | 1.002347226 | 0.979536701 |
| U70;ENSG00000207165;SNORA70_chrX_153628621_153628756 | 1.000271364 | 0.977624075 | 1.023443292 | 0.981474054 |
| ENSG00000253094;SNORD36_chr13_23377284_23377363 | 0.999860566 | 0.987916088 | 1.01194946 | 0.981856796 |
| ENSG00000200422;SNORD45_chrX_86401736_86401807 | 1.000081808 | 0.992765755 | 1.007451775 | 0.982578112 |
| U83B;ENSG00000209480;SNORD83B_chr22_39709823_39709916 | 0.999994429 | 0.999469416 | 1.000519718 | 0.983411998 |
| ENSG00000251778;SNORA3_chr21_43302314_43302409 | 0.999432742 | 0.947005572 | 1.054762332 | 0.983533146 |
| ENSG00000212182;U3_chr2_114763018_114763232 | 1.000000972 | 0.999893641 | 1.000108315 | 0.985836573 |
| ACA32;ENSG00000206799;SNORA32_chr11_93464144_93464265 | 0.999903488 | 0.986592798 | 1.013393761 | 0.988737756 |
| ENSG00000200026;U8_chr9_38147425_38147558 | 0.999838356 | 0.97684343 | 1.023374583 | 0.98913512 |
| SNORD125;ENSG00000239127;SNORD125_chr22_29729151_29729247 | 0.99999018 | 0.997901307 | 1.002083426 | 0.992656408 |
| ACA48;ENSG00000209582;SNORA48_chr17_7478030_7478165 | 0.999995181 | 0.993762537 | 1.006266914 | 0.998794568 |
| ENSG00000202252;SNORD14C_chr11_122930043_122930130 | 1.000000543 | 0.9991492 | 1.000852611 | 0.999003874 |
