# Supplementary figures and images for: Small nucleolar RNAs signature (SNORS) identified clinical outcome and prognosis of bladder cancer (BLCA)
Source: Cancer Cell Int. 2020 Jul 10;20:299. doi: 10.1186/s12935-020-01393-7 (PMC7350589; doi:10.1186/s12935-020-01393-7)

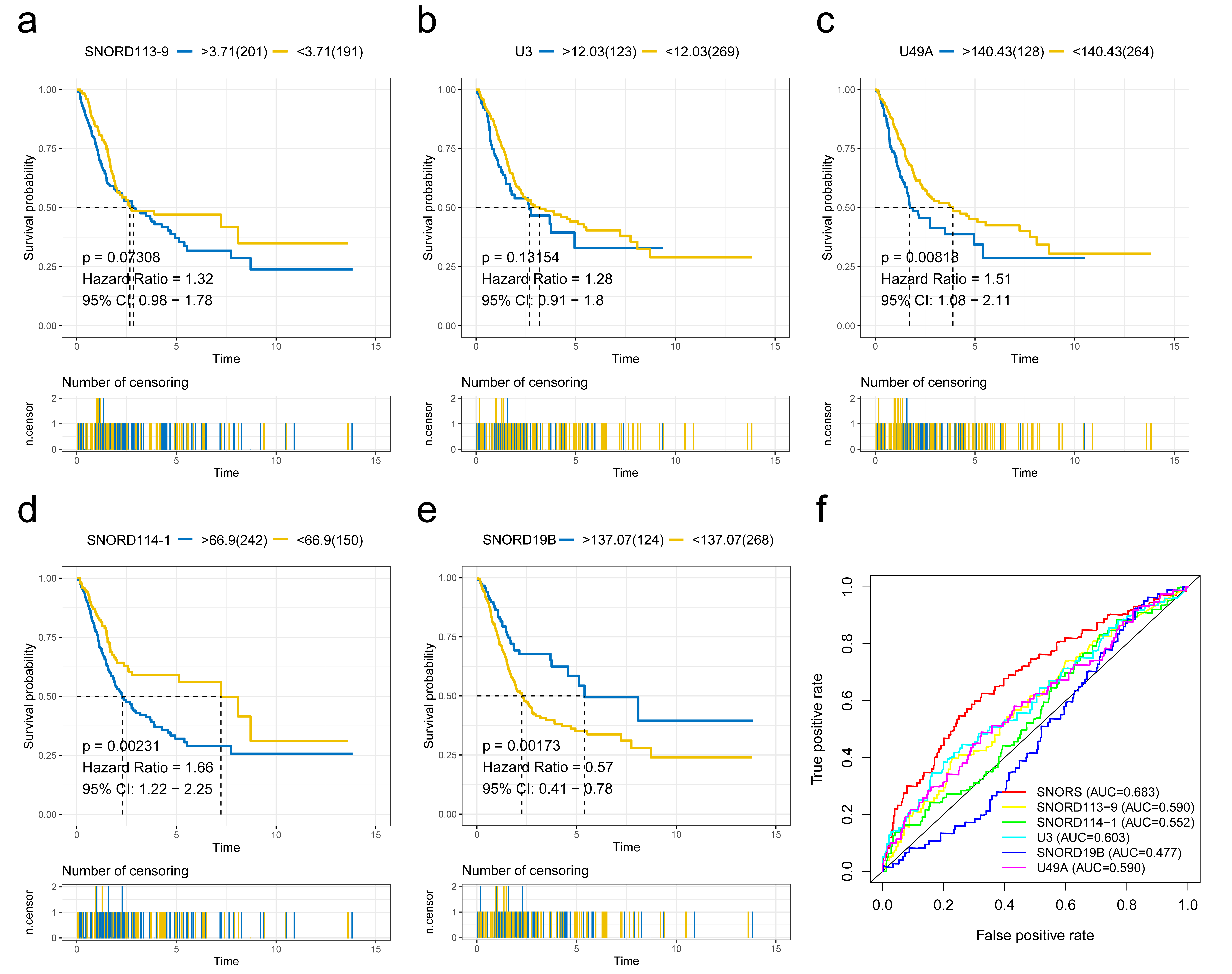

Supplement: Supplementary file 4 — Additional file 4: Figure S1. KM survival analyses of 5 candidate snoRNAs in TCGA-BLCA cohort. (a) SNORD113-9; (b) U3; (c) U49A; (d)SNORD114-1; (e) SNORD19B; (f) ROC curves of 5candidate snoRNAs and SNORS. [file 12935_2020_1393_MOESM4_ESM.tif]

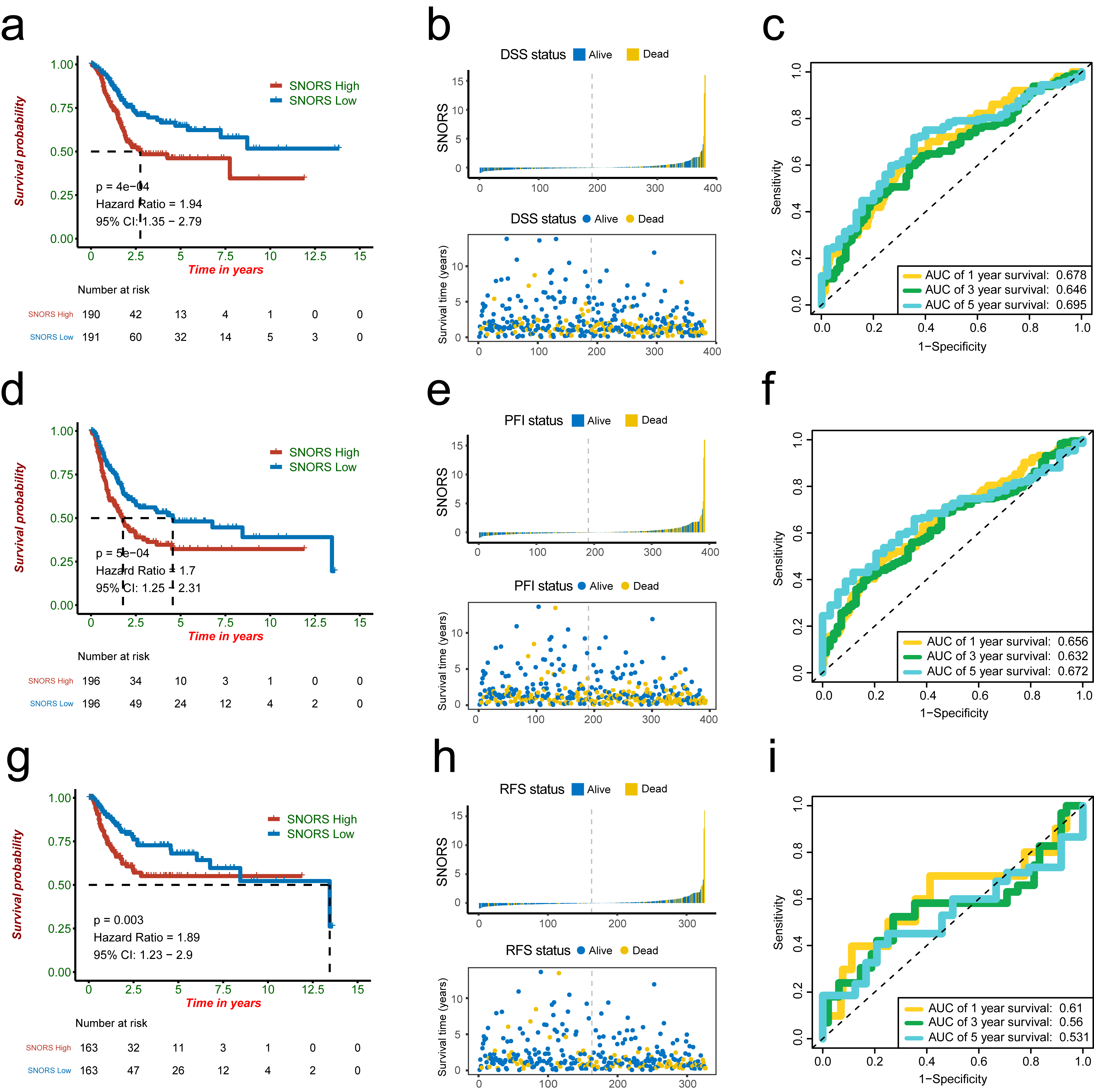

Supplement: Supplementary file 6 — Additional file 6: Figure S2. SNORS is a correlated with DSS, DFI and RFS in TCGA-BLCA cohort. (a-c) KM survival, risk score and time-dependent ROC curves of DFS according to SNORS groups in TCGA-BLCA cohort. (d-f) KM survival, risk score and time-dependent ROC curves of PFI according to SNORS groups in TCGA-BLCA cohort. (g-i) KM survival, risk score and time-dependent ROC curves of RFS according to SNORS groups in TCGA-BLCA cohort. The high SNORS and low SNORS groups were stratified at median cut-off. The AUC was assessed at 1, 3 and 5 years. [file 12935_2020_1393_MOESM6_ESM.tif]

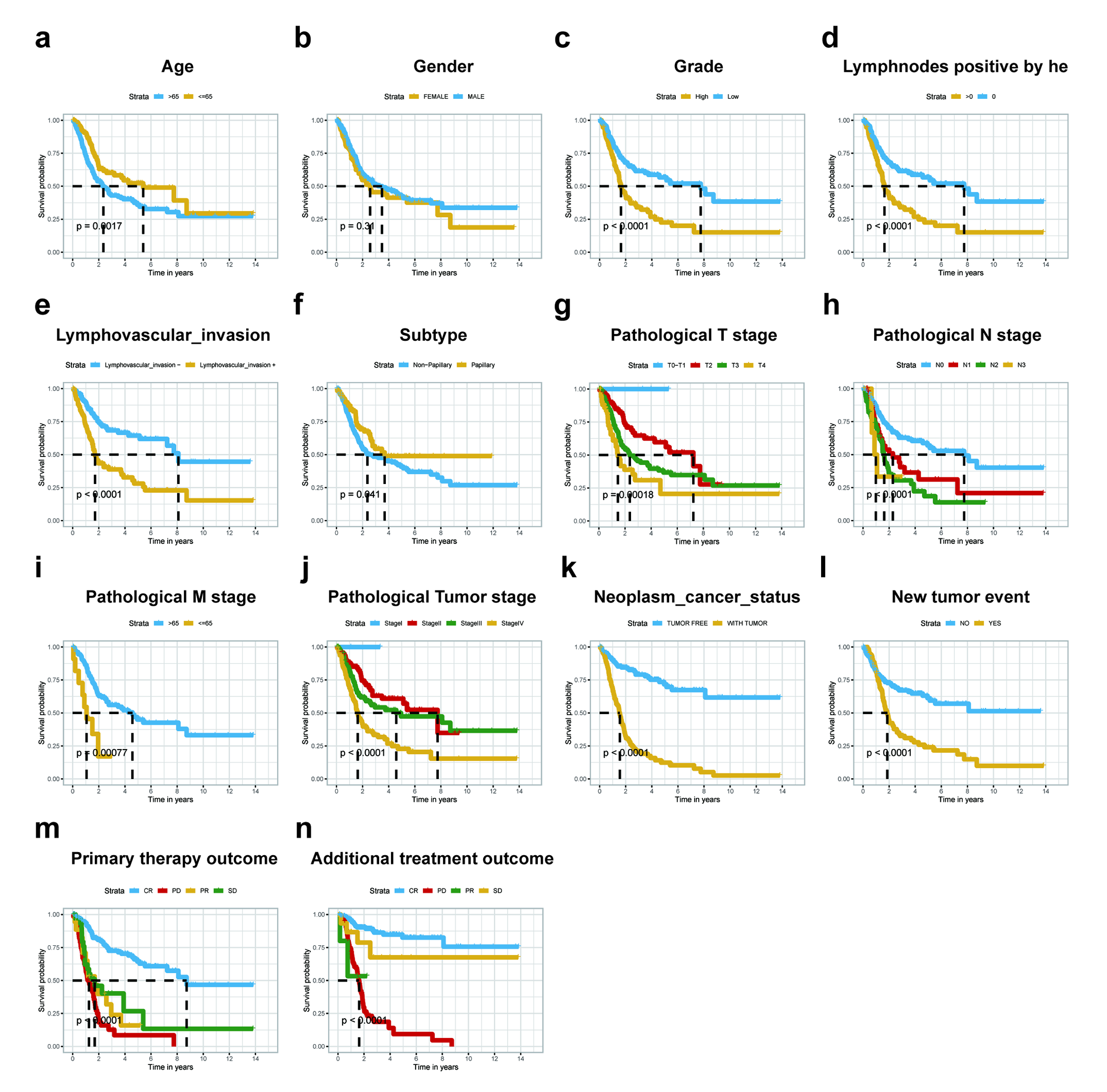

Supplement: Supplementary file 20 — Additional file 20: Figure S3. KM survival analyses of different clinicopathological characteristics in TCGA-BLCA cohort. (a) Age. (b) Gender. (c) Grade. (d) lymphnodes positive by HE. (e) Lymphovascular invasion. (f) Subtype. (g) Pathology T stage. (h) Pathology N stage. (i) Pathology M stage. (j) Pathology tumor stage. (k) Neoplasm cancer status. (l) New tumor event. (m) Primary therapy outcome. (n) Additional treatment outcome. [file 12935_2020_1393_MOESM20_ESM.tif]

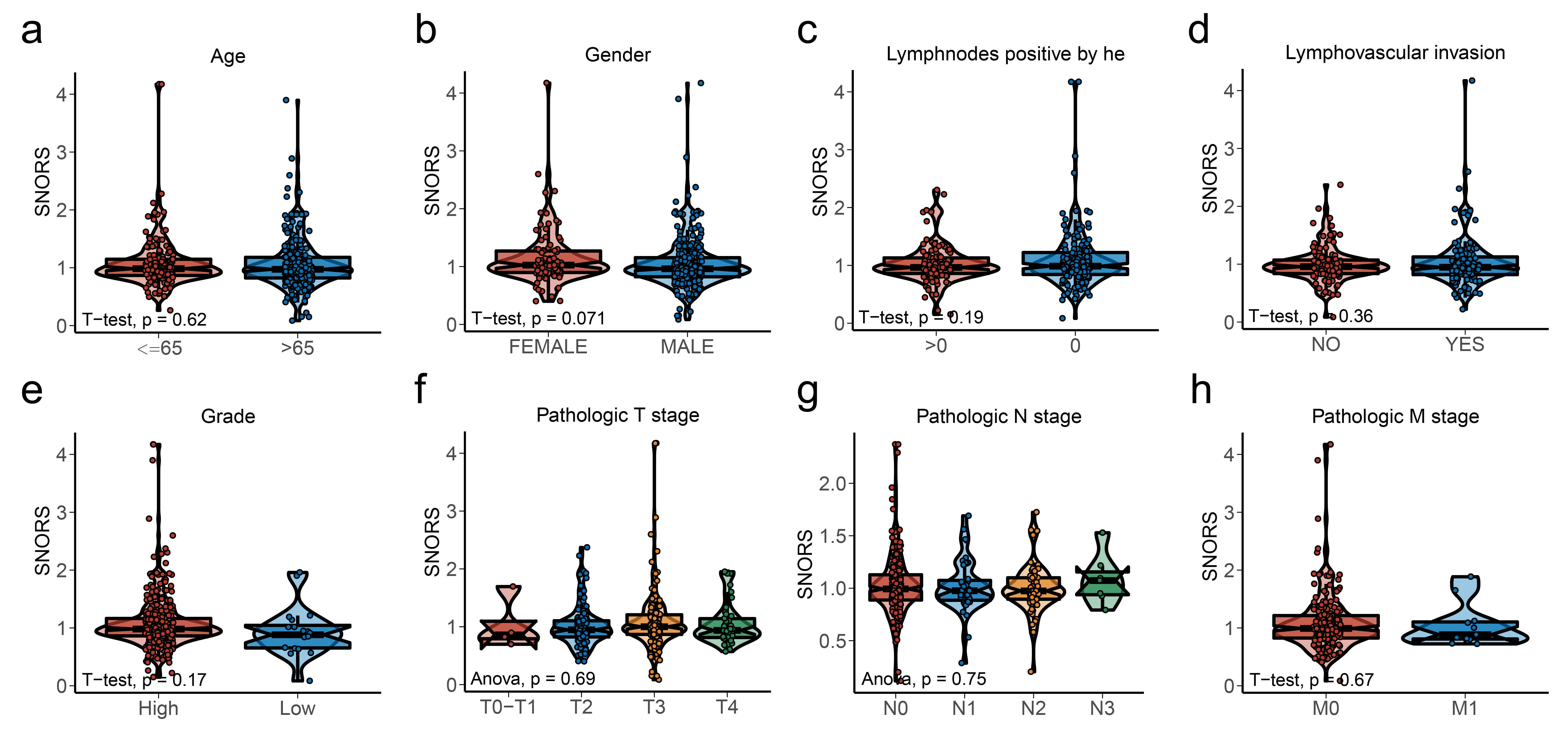

Supplement: Supplementary file 21 — Additional file 21: Figure S4. Differences in SNORS between different clinicopathological characteristics in TCGA-BLCA cohort. The upper and lower ends of the boxes represented interquartile range of values. The lines in the boxes represented median value. Student t tests and one-way Anova tests were used to compare the statistical difference between age (a), gender (b), lymphonodes positive by he (c), lymphovascular invasion (d), grade (e), pathological T stage (f), pathological N stage (h), and pathological M stage (i). [file 12935_2020_1393_MOESM21_ESM.tif]

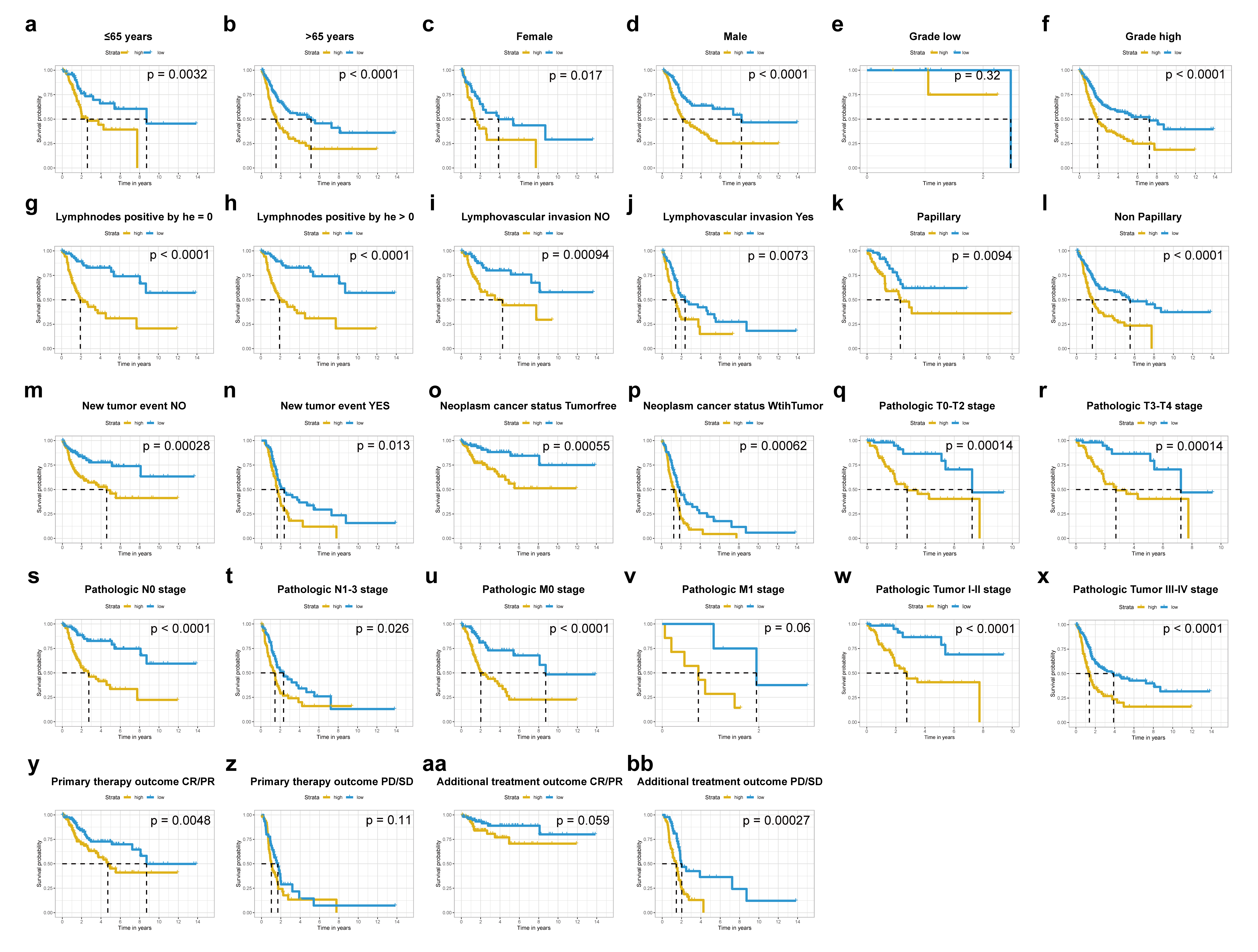

Supplement: Supplementary file 22 — Additional file 22: Figure S5. KM stratification survival analyses in TCGA-BLCA cohort. (a) Age≤65 years. (b) Age >65 years. (c) Female. (d) Male. (e) Grade low. (f) Grade high. (g) lymphnodes positive HE=0. (h) lymphnodes positive by HE>0. (i) Lymphovascular invasion NO. (j) Lymphovascular invasion YES. (k) Subtype papillary and non- papillary. (l) Subtype non-papillary. (m) New tumour event NO. (n) New tumour event YES. (o) Tumour-free. (p) With-tumour. (q) Pathology T0‐T2 stage. (r) Pathology T3‐T4 stage. (s) Pathology N0 stage. (t) Pathology N1-3 stage. (u) Pathology M0 stage. (v) Pathology M1 stage. (w) Pathology tumour I-II stage. (x) Pathology tumour III-IV stage. (y) Primary therapy outcome CR/PR. (z) Primary therapy outcome PD/SD. (aa) Additional treatment outcome CR/PR. (bb) Additional treatment outcome PD/SD. [file 12935_2020_1393_MOESM22_ESM.tif]
