# Supplementary material for: Small nucleolar RNAs signature (SNORS) identified clinical outcome and prognosis of bladder cancer (BLCA)
Source: Cancer Cell Int. 2020 Jul 10;20:299. doi: 10.1186/s12935-020-01393-7 (PMC7350589; doi:10.1186/s12935-020-01393-7)
Supplement: Supplementary file 5 — Additional file 5: Table S4. Comparison of AUC values between SNORS and 5 candidate snoRNAs in TCGA-BLCA cohort. [file 12935_2020_1393_MOESM5_ESM.docx]

**Additional file 5: Table S4 Comparison of AUC values between SNORS and 5 candidate snoRNAs (n = 392)**

| Variable 1 | SNORS |
| --- | --- |
| Variable 2 | SNORD113-9 |
| Variable 3 | SNORD114-1 |
| Variable 4 | SNORD19B |
| Variable 5 | U3 |
| Variable 6 | U49A |
| Classification variable | OS status |

| Sample size | 392 |
| --- | --- |
| Positive group ^a^ | 171 (43.62%) |
| Negative group ^b^ | 221 (56.38%) |

^a^ OS status = Dead
^b^ OS status = Alive

| Variable | AUC | SE ^a^ | 95% CI ^b^ |
| --- | --- | --- | --- |
| SNORS | 0.683 | 0.0280 | 0.634 to 0.731 |
| SNORD113-9 | 0.590 | 0.0291 | 0.539 to 0.640 |
| SNORD114-1 | 0.552 | 0.0286 | 0.502 to 0.601 |
| SNORD19B | 0.477 | 0.0287 | 0.427 to 0.527 |
| U3 | 0.603 | 0.0294 | 0.552 to 0.653 |
| U49A | 0.590 | 0.0297 | 0.540 to 0.641 |

^a^ DeLong et al., 1988

^b^ Binomial exact

## Pairwise comparison of ROC curves

| SNORS ~ SNORD113-9 | |
| --- | --- |
| Difference between areas | 0.0931 |
| Standard Error ^a^ | 0.0378 |
| 95% Confidence Interval | 0.00521to 0.134 |
| z statistic | 3.117 |
| Significance level | P = 0.00068 |
| SNORS ~ SNORD114-1 | |
| Difference between areas | 0.131 |
| Standard Error ^a^ | 0.0281 |
| 95% Confidence Interval | 0.0842 to 0.180 |
| z statistic | 4.277 |
| Significance level | P < 0.0001 |
| SNORS ~ SNORD19B | |
| Difference between areas | 0.206 |
| Standard Error ^a^ | 0.0508 |
| 95% Confidence Interval | 0.1723 to 0.241 |
| z statistic | 8.201 |
| Significance level | P < 0.0001 |
| SNORS ~ U3 | |
| Difference between areas | 0.801 |
| Standard Error ^a^ | 0.0294 |
| 95% Confidence Interval | 0.0508 to 0.110 |
| z statistic | 4.708 |
| Significance level | P = 0.0007 |
| SNORS ~ U49A | |
| Difference between areas | 0.093 |
| Standard Error ^a^ | 0.0345 |
| 95% Confidence Interval | 0.0292 to 0.156 |
| z statistic | 3.389 |
| Significance level | P = 0.0002 |

^a^ DeLong et al., 1988
