# Supplementary material for: Small nucleolar RNAs signature (SNORS) identified clinical outcome and prognosis of bladder cancer (BLCA)
Source: Cancer Cell Int. 2020 Jul 10;20:299. doi: 10.1186/s12935-020-01393-7 (PMC7350589; doi:10.1186/s12935-020-01393-7)
Supplement: Supplementary file 7 — Additional file 7: Table S5. Correlation between candidate snoRNAs and their copy number variations (CNV) in TCGA-BLCA cohort. [file 12935_2020_1393_MOESM7_ESM.docx]

**Additional file 7: Table S5 Correlation between candidate snoRNAs and their copy number variations (CNV) in TCGA-BLCA cohort (n = 392)**

| id | CNV | cor | p.value |
| --- | --- | --- | --- |
| SNORD113-9 | CNV | -0.0054 | 0.9334 |
| SNORD114-1 | CNV | -0.0433 | 0.5059 |
| U3 | CNV | 0.1374 | 0.0342 |
| SNORD19B | CNV | 0.3651 | 6.45E-09 |
| U49A | CNV | 0.1265 | 0.0513 |
