# Supplementary material for: Small nucleolar RNAs signature (SNORS) identified clinical outcome and prognosis of bladder cancer (BLCA)
Source: Cancer Cell Int. 2020 Jul 10;20:299. doi: 10.1186/s12935-020-01393-7 (PMC7350589; doi:10.1186/s12935-020-01393-7)
Supplement: Supplementary file 8 — Additional file 8: Table S6. Correlation between candidate snoRNAs and their methylation sites in TCGA-BLCA cohort. [file 12935_2020_1393_MOESM8_ESM.docx]

**Additional file 8: Table S6 Correlation between candidate snoRNAs and their methylation sites in TCGA-BLCA cohort (n = 392)**

| id | methylation | cor | p.value |
| --- | --- | --- | --- |
| SNORD113-9 | cg02990507 | 0.0491 | 0.4489 |
| U3 | cg15879533 | -0.1174 | 0.0694 |
| U3 | cg06529477 | -0.1911 | 3.00E-03 |
| SNORD19B | cg00447581 | -0.1694 | 8.60E-03 |
| SNORD19B | cg24892309 | -0.2457 | 1.00E-04 |
| SNORD19B | cg21199854 | -0.2617 | 0 |
| SNORD19B | cg17036641 | -0.5405 | 0 |
| SNORD19B | cg15685941 | -0.5441 | 0 |
| U49A | cg17862024 | 0.3035 | 1.67E-06 |
| U49A | cg02091366 | 0.2841 | 7.77E-06 |
| U49A | cg02234479 | 0.2743 | 0 |
| U49A | cg02830903 | 0.2738 | 0 |
| U49A | cg24707616 | 0.2497 | 1.00E-04 |
| U49A | cg01154849 | 0.2332 | 3.00E-04 |
| U49A | cg10254974 | 0.2206 | 6.00E-04 |
| U49A | cg03364076 | 0.2149 | 8.00E-04 |
| U49A | cg01501135 | 0.1969 | 2.20E-03 |
| U49A | cg19179603 | 0.1433 | 0.0264 |
