# Supplementary material for: Small nucleolar RNAs signature (SNORS) identified clinical outcome and prognosis of bladder cancer (BLCA)
Source: Cancer Cell Int. 2020 Jul 10;20:299. doi: 10.1186/s12935-020-01393-7 (PMC7350589; doi:10.1186/s12935-020-01393-7)
Supplement: Supplementary file 9 — Additional file 9: Table S7. Correlation between candidate snoRNAs and relevant proteins in TCGA-BLCA cohort. [file 12935_2020_1393_MOESM9_ESM.docx]

**Additional file 9: Table S7 Correlation between candidate snoRNAs and relevant proteins in TCGA-BLCA cohort (n = 392)**

| id | protein | cor | p.value |
| --- | --- | --- | --- |
| SNORD114-1 | CD20 | -0.3281 | 9.55E-10 |
| U3 | Bak | -0.3075 | 7.68E-06 |
| SNORD19B | ACVRL1 | -0.3616 | 1.16E-11 |
| U49A | Chk2 | 0.3114 | 6.48E-06 |
