# Supplementary material for: Small nucleolar RNAs signature (SNORS) identified clinical outcome and prognosis of bladder cancer (BLCA)
Source: Cancer Cell Int. 2020 Jul 10;20:299. doi: 10.1186/s12935-020-01393-7 (PMC7350589; doi:10.1186/s12935-020-01393-7)
Supplement: Supplementary file 10 — Additional file 10: Table S8. Correlation between candidate snoRNAs and mRNAs in TCGA-BLCA cohort. [file 12935_2020_1393_MOESM10_ESM.docx]

**Additional file 10: Table S8 Correlation between candidate snoRNAs and mRNAs in TCGA-BLCA cohorts (n = 392)**

| id | Gene Symbol | Coefficient | P-value |
| --- | --- | --- | --- |
| SNORD19B | GNL3 | 0.5903 | 2.89E-38 |
| SNORD19B | FLNA | -0.5421 | 2.11E-31 |
| SNORD19B | PALLD | -0.5209 | 1.01E-28 |
| SNORD19B | DNAJB5 | -0.5139 | 7.09E-28 |
| SNORD19B | AHNAK2 | -0.5132 | 8.63E-28 |
| SNORD19B | PTGS1 | -0.5025 | 1.57E-26 |
| SNORD19B | ACTN1 | -0.5021 | 1.73E-26 |
| SNORD19B | PTRF | -0.5 | 3.02E-26 |
| SNORD19B | RAB23 | -0.4937 | 1.56E-25 |
| SNORD19B | JAZF1 | -0.4927 | 2.00E-25 |
| SNORD19B | ITGA5 | -0.4924 | 2.18E-25 |
| SNORD19B | ROR2 | -0.4853 | 1.29E-24 |
| SNORD19B | CLIC4 | -0.4835 | 2.04E-24 |
| SNORD19B | ZAK | -0.4823 | 2.77E-24 |
| SNORD19B | CALD1 | -0.4821 | 2.88E-24 |
| SNORD19B | GNB4 | -0.4813 | 3.52E-24 |
| SNORD19B | KATNAL1 | -0.4782 | 7.60E-24 |
| SNORD19B | FLNC | -0.4773 | 9.35E-24 |
| SNORD19B | CAMK2A | -0.4771 | 9.77E-24 |
| SNORD19B | CFL2 | -0.4759 | 1.32E-23 |
| SNORD19B | ARSI | -0.4754 | 1.47E-23 |
| SNORD19B | ARSJ | -0.4716 | 3.75E-23 |
| SNORD19B | PRKCDBP | -0.4713 | 4.02E-23 |
| SNORD19B | CSRP1 | -0.4713 | 4.03E-23 |
| SNORD19B | CSF1 | -0.4711 | 4.19E-23 |
| SNORD19B | MSN | -0.4707 | 4.63E-23 |
| SNORD19B | ANXA5 | -0.4697 | 5.92E-23 |
| SNORD19B | DPYD | -0.4692 | 6.64E-23 |
| SNORD19B | CORO1C | -0.4683 | 8.12E-23 |
| SNORD19B | TNC | -0.4681 | 8.51E-23 |
| SNORD19B | KLHL5 | -0.468 | 8.82E-23 |
| SNORD19B | PDLIM7 | -0.4677 | 9.42E-23 |
| SNORD19B | OSMR | -0.4674 | 1.02E-22 |
| SNORD19B | BAG2 | -0.4665 | 1.24E-22 |
| SNORD19B | COPZ2 | -0.4654 | 1.61E-22 |
| SNORD19B | IFITM3 | -0.4594 | 6.53E-22 |
| SNORD19B | CD109 | -0.458 | 8.93E-22 |
| SNORD19B | TGFB3 | -0.4577 | 9.61E-22 |
| SNORD19B | C13orf18 | -0.4561 | 1.37E-21 |
| SNORD19B | NFIX | -0.4551 | 1.75E-21 |
| SNORD19B | KIAA1949 | -0.4549 | 1.80E-21 |
| SNORD19B | PARVA | -0.4542 | 2.11E-21 |
| SNORD19B | IGSF21 | -0.454 | 2.24E-21 |
| SNORD19B | SLC22A3 | -0.4539 | 2.30E-21 |
| SNORD19B | NRP2 | -0.4532 | 2.68E-21 |
| SNORD19B | TRAK1 | 0.4524 | 3.21E-21 |
| SNORD19B | AP1S2 | -0.4521 | 3.40E-21 |
| SNORD19B | CCDC80 | -0.4516 | 3.84E-21 |
| SNORD19B | MSRB3 | -0.4513 | 4.05E-21 |
| SNORD19B | COL6A2 | -0.4511 | 4.29E-21 |
| SNORD19B | ZFPM2 | -0.4505 | 4.87E-21 |
| SNORD19B | SCAP | 0.4504 | 5.00E-21 |
| SNORD19B | NEXN | -0.4503 | 5.09E-21 |
| SNORD19B | BNC2 | -0.4494 | 6.17E-21 |
| SNORD19B | GAS1 | -0.4489 | 6.96E-21 |
| SNORD19B | SYNM | -0.4481 | 8.39E-21 |
| SNORD19B | RAB27A | -0.4479 | 8.71E-21 |
| SNORD19B | C1S | -0.4478 | 8.79E-21 |
| SNORD19B | COL8A2 | -0.4474 | 9.69E-21 |
| SNORD19B | COL16A1 | -0.4471 | 1.03E-20 |
| SNORD19B | KIAA0922 | -0.4461 | 1.28E-20 |
| SNORD19B | C1R | -0.4455 | 1.49E-20 |
| SNORD19B | PLSCR4 | -0.4451 | 1.61E-20 |
| SNORD19B | AKAP6 | -0.445 | 1.63E-20 |
| SNORD19B | P4HTM | 0.4448 | 1.72E-20 |
| SNORD19B | ACOXL | 0.4447 | 1.77E-20 |
| SNORD19B | ZNF532 | -0.4445 | 1.82E-20 |
| SNORD19B | MYLK | -0.4442 | 1.94E-20 |
| SNORD19B | TGFBI | -0.4426 | 2.75E-20 |
| SNORD19B | CHST15 | -0.4415 | 3.55E-20 |
| SNORD19B | IP6K2 | 0.4408 | 4.10E-20 |
| SNORD19B | ADAM19 | -0.4407 | 4.23E-20 |
| SNORD19B | ANXA6 | -0.4403 | 4.53E-20 |
| SNORD19B | GFPT2 | -0.44 | 4.84E-20 |
| SNORD19B | CCIN | -0.4396 | 5.29E-20 |
| SNORD19B | WWTR1 | -0.4396 | 5.33E-20 |
| SNORD19B | SGTB | -0.439 | 5.99E-20 |
| SNORD19B | APCDD1L | -0.439 | 6.06E-20 |
| SNORD19B | ACAA1 | 0.4389 | 6.22E-20 |
| SNORD19B | CRYAB | -0.4382 | 7.17E-20 |
| SNORD19B | BIN1 | -0.4374 | 8.54E-20 |
| SNORD19B | FAM20C | -0.4373 | 8.75E-20 |
| SNORD19B | ST5 | -0.4365 | 1.04E-19 |
| SNORD19B | SPHK1 | -0.436 | 1.16E-19 |
| SNORD19B | OLFML3 | -0.4358 | 1.19E-19 |
| SNORD19B | MAMLD1 | -0.4354 | 1.30E-19 |
| SNORD19B | MAP7D1 | -0.4348 | 1.49E-19 |
| SNORD19B | MFGE8 | -0.4343 | 1.65E-19 |
| SNORD19B | LOX | -0.4341 | 1.71E-19 |
| SNORD19B | EMP3 | -0.434 | 1.76E-19 |
| SNORD19B | SPEG | -0.4339 | 1.80E-19 |
| SNORD19B | STOM | -0.4336 | 1.93E-19 |
| SNORD19B | KLF17 | -0.4331 | 2.14E-19 |
| SNORD19B | CAPN2 | -0.433 | 2.16E-19 |
| SNORD19B | PDLIM3 | -0.4327 | 2.33E-19 |
| SNORD19B | ALAS1 | 0.4324 | 2.45E-19 |
| SNORD19B | GPR176 | -0.432 | 2.69E-19 |
| SNORD19B | NAV3 | -0.4318 | 2.78E-19 |
| SNORD19B | ACVR1 | -0.4312 | 3.14E-19 |
| SNORD19B | ANKRD29 | -0.4312 | 3.16E-19 |
| SNORD19B | FIBIN | -0.4311 | 3.26E-19 |
| SNORD19B | NCOR2 | -0.431 | 3.28E-19 |
| SNORD19B | PRRX1 | -0.4308 | 3.41E-19 |
| SNORD19B | ASB2 | -0.43 | 4.06E-19 |
| SNORD19B | KLHL38 | -0.4299 | 4.11E-19 |
| SNORD19B | TLR1 | -0.4298 | 4.22E-19 |
| SNORD19B | C20orf151 | 0.4297 | 4.35E-19 |
| SNORD19B | TNFAIP8L3 | -0.4294 | 4.57E-19 |
| SNORD19B | PPARG | 0.4294 | 4.61E-19 |
| SNORD19B | MN1 | -0.429 | 4.96E-19 |
| SNORD19B | OSBPL6 | -0.4287 | 5.33E-19 |
| SNORD19B | RAB3IL1 | -0.4271 | 7.44E-19 |
| SNORD19B | CHRDL2 | -0.4264 | 8.55E-19 |
| SNORD19B | RAMP1 | -0.4264 | 8.61E-19 |
| SNORD19B | FAM129A | -0.4264 | 8.62E-19 |
| SNORD19B | KSR2 | 0.4262 | 8.87E-19 |
| SNORD19B | STX2 | -0.4261 | 9.05E-19 |
| SNORD19B | PRUNE2 | -0.426 | 9.29E-19 |
| SNORD19B | KIAA1644 | -0.4254 | 1.05E-18 |
| SNORD19B | TPM2 | -0.4254 | 1.06E-18 |
| SNORD19B | STAT3 | -0.425 | 1.15E-18 |
| SNORD19B | DEGS1 | -0.4249 | 1.18E-18 |
| SNORD19B | GXYLT2 | -0.4247 | 1.22E-18 |
| SNORD19B | RRAS | -0.4246 | 1.23E-18 |
| SNORD19B | KLHL4 | -0.4245 | 1.27E-18 |
| SNORD19B | FAM20A | -0.4244 | 1.30E-18 |
| SNORD19B | GPR1 | -0.4244 | 1.30E-18 |
| SNORD19B | PDGFC | -0.424 | 1.42E-18 |
| SNORD19B | C9orf21 | -0.4239 | 1.43E-18 |
| SNORD19B | NCAM1 | -0.4233 | 1.60E-18 |
| SNORD19B | CCL26 | -0.4233 | 1.61E-18 |
| SNORD19B | NR3C1 | -0.4228 | 1.80E-18 |
| SNORD19B | ARHGAP24 | -0.4227 | 1.82E-18 |
| SNORD19B | FAM46B | -0.4224 | 1.95E-18 |
| SNORD19B | TGFB1I1 | -0.4218 | 2.21E-18 |
| SNORD19B | C17orf28 | 0.4216 | 2.29E-18 |
| SNORD19B | ST3GAL6 | -0.4215 | 2.34E-18 |
| SNORD19B | MAOB | -0.4203 | 2.96E-18 |
| SNORD19B | FER1L4 | 0.4202 | 3.04E-18 |
| SNORD19B | PLN | -0.4201 | 3.12E-18 |
| SNORD19B | GPX8 | -0.4199 | 3.23E-18 |
| SNORD19B | PEBP1 | 0.4197 | 3.34E-18 |
| SNORD19B | PLXNA4 | -0.4196 | 3.40E-18 |
| SNORD19B | COL6A1 | -0.4193 | 3.62E-18 |
| SNORD19B | HTRA3 | -0.4185 | 4.26E-18 |
| SNORD19B | TLN1 | -0.4185 | 4.27E-18 |
| SNORD19B | AXL | -0.4184 | 4.38E-18 |
| SNORD19B | ICA1 | 0.4183 | 4.46E-18 |
| SNORD19B | RSPO3 | -0.4183 | 4.46E-18 |
| SNORD19B | KCTD12 | -0.4182 | 4.55E-18 |
| SNORD19B | SLC43A3 | -0.4177 | 5.06E-18 |
| SNORD19B | KIAA1614 | -0.4173 | 5.48E-18 |
| SNORD19B | WDR6 | 0.4169 | 5.83E-18 |
| SNORD19B | SCNN1G | 0.4164 | 6.50E-18 |
| SNORD19B | SERPING1 | -0.4163 | 6.67E-18 |
| SNORD19B | SFRP2 | -0.4163 | 6.70E-18 |
| SNORD19B | PIK3CD | -0.4161 | 6.93E-18 |
| SNORD19B | EHD2 | -0.4158 | 7.38E-18 |
| SNORD19B | CYBRD1 | -0.4156 | 7.59E-18 |
| SNORD19B | NCF2 | -0.4155 | 7.76E-18 |
| SNORD19B | RAB31 | -0.4151 | 8.49E-18 |
| SNORD19B | GNAO1 | -0.4147 | 9.11E-18 |
| SNORD19B | MFAP5 | -0.4147 | 9.19E-18 |
| SNORD19B | LRIG1 | -0.4144 | 9.63E-18 |
| SNORD19B | RUSC2 | -0.4141 | 1.02E-17 |
| SNORD19B | CREB5 | -0.414 | 1.06E-17 |
| SNORD19B | PLXNB1 | 0.4136 | 1.14E-17 |
| SNORD19B | RBMS3 | -0.4129 | 1.29E-17 |
| SNORD19B | DPYSL3 | -0.4127 | 1.35E-17 |
| SNORD19B | CNTN1 | -0.4122 | 1.48E-17 |
| SNORD19B | DNAJB4 | -0.4113 | 1.78E-17 |
| SNORD19B | OMD | -0.4113 | 1.79E-17 |
| SNORD19B | SGCB | -0.4101 | 2.25E-17 |
| SNORD19B | CYB5A | 0.4101 | 2.26E-17 |
| SNORD19B | GPC6 | -0.41 | 2.29E-17 |
| SNORD19B | BACH2 | -0.4098 | 2.37E-17 |
| SNORD19B | KIAA0114 | 0.4096 | 2.47E-17 |
| SNORD19B | CCPG1 | -0.4093 | 2.62E-17 |
| SNORD19B | BCAT2 | 0.4092 | 2.69E-17 |
| SNORD19B | DACT1 | -0.4091 | 2.76E-17 |
| SNORD19B | TRIM47 | -0.4085 | 3.08E-17 |
| SNORD19B | GLIPR1 | -0.4084 | 3.16E-17 |
| SNORD19B | KCNMB1 | -0.408 | 3.41E-17 |
| SNORD19B | FERMT2 | -0.4078 | 3.53E-17 |
| SNORD19B | C21orf34 | -0.4078 | 3.54E-17 |
| SNORD19B | AEBP1 | -0.4075 | 3.76E-17 |
| SNORD19B | FAM69A | -0.4073 | 3.91E-17 |
| SNORD19B | TAGLN | -0.4069 | 4.18E-17 |
| SNORD19B | UPK2 | 0.4068 | 4.24E-17 |
| SNORD19B | DPT | -0.4068 | 4.30E-17 |
| SNORD19B | P4HA3 | -0.4066 | 4.41E-17 |
| SNORD19B | LRP1 | -0.4066 | 4.43E-17 |
| SNORD19B | CTHRC1 | -0.4064 | 4.61E-17 |
| SNORD19B | GLI2 | -0.4057 | 5.26E-17 |
| SNORD19B | KAL1 | -0.4052 | 5.85E-17 |
| SNORD19B | ALOX5AP | -0.4046 | 6.57E-17 |
| SNORD19B | HSPB7 | -0.4044 | 6.77E-17 |
| SNORD19B | HS3ST3A1 | -0.4042 | 7.09E-17 |
| SNORD19B | ZCCHC5 | -0.4037 | 7.69E-17 |
| SNORD19B | KIAA1755 | -0.4037 | 7.77E-17 |
| SNORD19B | ZEB2 | -0.4037 | 7.79E-17 |
| SNORD19B | ADAMTS14 | -0.4036 | 7.85E-17 |
| SNORD19B | LOC399959 | -0.4036 | 7.90E-17 |
| SNORD19B | ANKH | -0.4035 | 8.09E-17 |
| SNORD19B | TYMP | -0.4034 | 8.16E-17 |
| SNORD19B | MXRA7 | -0.4034 | 8.21E-17 |
| SNORD19B | KCNQ5 | -0.4034 | 8.22E-17 |
| SNORD19B | SGCD | -0.4034 | 8.26E-17 |
| SNORD19B | EID3 | -0.4034 | 8.29E-17 |
| SNORD19B | MEOX2 | -0.4031 | 8.64E-17 |
| SNORD19B | SYNPO | -0.403 | 8.85E-17 |
| SNORD19B | TEKT5 | 0.4028 | 9.23E-17 |
| SNORD19B | MAP1B | -0.4028 | 9.25E-17 |
| SNORD19B | ELOVL6 | 0.4025 | 9.75E-17 |
| SNORD19B | KIAA0247 | -0.4024 | 9.96E-17 |
| SNORD19B | PNMA1 | -0.4023 | 1.02E-16 |
| SNORD19B | TUBB6 | -0.4022 | 1.04E-16 |
| SNORD19B | JPH2 | -0.4021 | 1.06E-16 |
| SNORD19B | MACF1 | -0.4021 | 1.06E-16 |
| SNORD19B | TOX3 | 0.4019 | 1.09E-16 |
| SNORD19B | HMGCS2 | 0.4015 | 1.19E-16 |
| SNORD19B | C10orf129 | 0.4014 | 1.20E-16 |
| SNORD19B | SPCS1 | 0.4013 | 1.21E-16 |
| SNORD19B | EFEMP1 | -0.4012 | 1.24E-16 |
| SNORD19B | MAP3K3 | -0.4009 | 1.31E-16 |
| SNORD19B | ZFHX4 | -0.4009 | 1.31E-16 |
| SNORD19B | ZNF469 | -0.4009 | 1.33E-16 |
| SNORD19B | SPON2 | -0.4008 | 1.34E-16 |
| SNORD19B | PABPC1 | 0.4008 | 1.35E-16 |
| SNORD19B | ITPRIP | -0.4006 | 1.41E-16 |
| SNORD19B | GATA3 | 0.4004 | 1.46E-16 |
| SNORD19B | PMEPA1 | -0.3998 | 1.63E-16 |
| SNORD19B | GLIPR2 | -0.3997 | 1.65E-16 |
| SNORD19B | TNS1 | -0.3996 | 1.67E-16 |
| SNORD19B | C6orf142 | -0.3995 | 1.73E-16 |
| SNORD19B | MPP1 | -0.3993 | 1.77E-16 |
| SNORD19B | PPFIBP2 | 0.399 | 1.88E-16 |
| SNORD19B | SLC16A2 | -0.3987 | 2.01E-16 |
| SNORD19B | CNN1 | -0.3986 | 2.04E-16 |
| SNORD19B | PDCD1LG2 | -0.3986 | 2.05E-16 |
| SNORD19B | CYLD | -0.3984 | 2.13E-16 |
| SNORD19B | SCN1B | -0.3982 | 2.18E-16 |
| SNORD19B | ANXA2P1 | -0.3982 | 2.21E-16 |
| SNORD19B | WIPF1 | -0.398 | 2.27E-16 |
| SNORD19B | CHSY3 | -0.398 | 2.29E-16 |
| SNORD19B | FMO2 | -0.398 | 2.30E-16 |
| SNORD19B | PIP4K2A | -0.3978 | 2.38E-16 |
| SNORD19B | ORMDL3 | 0.3974 | 2.53E-16 |
| SNORD19B | FAM55C | -0.3973 | 2.60E-16 |
| SNORD19B | DIXDC1 | -0.3971 | 2.70E-16 |
| SNORD19B | GPR68 | -0.3969 | 2.78E-16 |
| SNORD19B | FAM26E | -0.3966 | 2.94E-16 |
| SNORD19B | RGL3 | 0.3966 | 2.97E-16 |
| SNORD19B | CSDC2 | -0.3964 | 3.09E-16 |
| SNORD19B | HAPLN3 | -0.3962 | 3.21E-16 |
| SNORD19B | TMEM173 | -0.3959 | 3.40E-16 |
| SNORD19B | PCBD1 | 0.3958 | 3.45E-16 |
| SNORD19B | PLEKHA4 | -0.3958 | 3.45E-16 |
| SNORD19B | MRC2 | -0.3957 | 3.48E-16 |
| SNORD19B | PLIN5 | 0.3957 | 3.49E-16 |
| SNORD19B | SAMD10 | 0.3956 | 3.59E-16 |
| SNORD19B | TIMP2 | -0.3954 | 3.72E-16 |
| SNORD19B | C19orf45 | 0.3947 | 4.20E-16 |
| SNORD19B | HSD17B6 | -0.3945 | 4.40E-16 |
| SNORD19B | DDR2 | -0.3942 | 4.61E-16 |
| SNORD19B | RGS4 | -0.3942 | 4.66E-16 |
| SNORD19B | MT1L | -0.3941 | 4.70E-16 |
| SNORD19B | FAS | -0.394 | 4.76E-16 |
| SNORD19B | CCDC72 | 0.394 | 4.82E-16 |
| SNORD19B | ADCY9 | -0.3938 | 4.99E-16 |
| SNORD19B | NNMT | -0.3937 | 5.06E-16 |
| SNORD19B | MAF | -0.3936 | 5.20E-16 |
| SNORD19B | GATA6 | -0.393 | 5.78E-16 |
| SNORD19B | STK32B | -0.3928 | 5.93E-16 |
| SNORD19B | RASSF9 | -0.3924 | 6.45E-16 |
| SNORD19B | AP1M2 | 0.3921 | 6.83E-16 |
| SNORD19B | ATP8B4 | -0.392 | 6.92E-16 |
| SNORD19B | TLR6 | -0.392 | 6.97E-16 |
| SNORD19B | FKBP5 | -0.3915 | 7.66E-16 |
| SNORD19B | HCK | -0.3914 | 7.77E-16 |
| SNORD19B | CFI | -0.3914 | 7.80E-16 |
| SNORD19B | HIC1 | -0.3913 | 7.82E-16 |
| SNORD19B | BEST1 | -0.3913 | 7.93E-16 |
| SNORD19B | CLEC2B | -0.3912 | 8.08E-16 |
| SNORD19B | LMCD1 | -0.3909 | 8.42E-16 |
| SNORD19B | PCDHGC3 | -0.3908 | 8.57E-16 |
| SNORD19B | FBP1 | 0.3908 | 8.66E-16 |
| SNORD19B | SPIRE2 | 0.3907 | 8.73E-16 |
| SNORD19B | C1orf38 | -0.3907 | 8.81E-16 |
| SNORD19B | FBN1 | -0.3907 | 8.86E-16 |
| SNORD19B | SEMA3A | -0.3906 | 8.90E-16 |
| SNORD19B | BCKDHB | 0.3905 | 9.07E-16 |
| SNORD19B | PAM | -0.3904 | 9.27E-16 |
| SNORD19B | HTRA1 | -0.3902 | 9.70E-16 |
| SNORD19B | ARHGAP20 | -0.3899 | 1.01E-15 |
| SNORD19B | AOX1 | -0.3898 | 1.04E-15 |
| SNORD19B | MST1 | 0.3894 | 1.12E-15 |
| SNORD19B | FLJ36031 | -0.3892 | 1.16E-15 |
| SNORD19B | MARVELD1 | -0.3891 | 1.18E-15 |
| SNORD19B | NFIL3 | -0.389 | 1.19E-15 |
| SNORD19B | PKDCC | -0.3891 | 1.19E-15 |
| SNORD19B | SULF2 | -0.3889 | 1.23E-15 |
| SNORD19B | FAM180A | -0.3885 | 1.31E-15 |
| SNORD19B | NCKAP1L | -0.3884 | 1.33E-15 |
| SNORD19B | TNFAIP6 | -0.3884 | 1.34E-15 |
| SNORD19B | NUAK1 | -0.3882 | 1.39E-15 |
| SNORD19B | GLIS2 | -0.3881 | 1.41E-15 |
| SNORD19B | ACTA2 | -0.3881 | 1.42E-15 |
| SNORD19B | DTX4 | 0.3881 | 1.42E-15 |
| SNORD19B | TBX3 | 0.3881 | 1.42E-15 |
| SNORD19B | PCDH7 | -0.388 | 1.43E-15 |
| SNORD19B | TNFRSF9 | -0.388 | 1.43E-15 |
| SNORD19B | TMPRSS2 | 0.3879 | 1.45E-15 |
| SNORD19B | SSC5D | -0.3879 | 1.46E-15 |
| SNORD19B | EGR2 | -0.3877 | 1.52E-15 |
| SNORD19B | SLC16A1 | -0.3874 | 1.60E-15 |
| SNORD19B | CELF2 | -0.3873 | 1.63E-15 |
| SNORD19B | SRPX | -0.3869 | 1.77E-15 |
| SNORD19B | HIVEP3 | -0.3868 | 1.78E-15 |
| SNORD19B | FHL3 | -0.3867 | 1.81E-15 |
| SNORD19B | GFOD1 | -0.3866 | 1.85E-15 |
| SNORD19B | TIAM2 | -0.3866 | 1.85E-15 |
| SNORD19B | SPINK1 | 0.3865 | 1.87E-15 |
| SNORD19B | LEFTY2 | -0.3864 | 1.91E-15 |
| SNORD19B | FAP | -0.3863 | 1.95E-15 |
| SNORD19B | SIRPA | -0.3863 | 1.96E-15 |
| SNORD19B | CSF1R | -0.3862 | 1.98E-15 |
| SNORD19B | ZNF823 | 0.3861 | 2.01E-15 |
| SNORD19B | DOCK2 | -0.3859 | 2.10E-15 |
| SNORD19B | MBNL1 | -0.3859 | 2.11E-15 |
| SNORD19B | PLEKHO2 | -0.3858 | 2.15E-15 |
| SNORD19B | DACT3 | -0.3857 | 2.17E-15 |
| SNORD19B | DES | -0.3857 | 2.18E-15 |
| SNORD19B | ECM1 | -0.3856 | 2.24E-15 |
| SNORD19B | ICAM1 | -0.3855 | 2.26E-15 |
| SNORD19B | FAM190A | 0.3851 | 2.42E-15 |
| SNORD19B | SULF1 | -0.3851 | 2.43E-15 |
| SNORD19B | IFITM2 | -0.3851 | 2.44E-15 |
| SNORD19B | STX1B | -0.3849 | 2.52E-15 |
| SNORD19B | ABCC9 | -0.3849 | 2.53E-15 |
| SNORD19B | TMEM45A | -0.3849 | 2.53E-15 |
| SNORD19B | TWIST2 | -0.3848 | 2.54E-15 |
| SNORD19B | IL32 | -0.3848 | 2.58E-15 |
| SNORD19B | SACS | -0.3847 | 2.63E-15 |
| SNORD19B | CYTH4 | -0.3845 | 2.72E-15 |
| SNORD19B | KCNC4 | -0.3844 | 2.74E-15 |
| SNORD19B | POSTN | -0.3844 | 2.74E-15 |
| SNORD19B | CHST11 | -0.3843 | 2.80E-15 |
| SNORD19B | ASPN | -0.3842 | 2.84E-15 |
| SNORD19B | ATP8B2 | -0.3842 | 2.84E-15 |
| SNORD19B | EPN3 | 0.3841 | 2.92E-15 |
| SNORD19B | MYL9 | -0.3839 | 3.00E-15 |
| SNORD19B | CLEC5A | -0.3838 | 3.08E-15 |
| SNORD19B | ODZ3 | -0.3836 | 3.16E-15 |
| SNORD19B | VSIG2 | 0.3836 | 3.18E-15 |
| SNORD19B | FXYD6 | -0.3835 | 3.21E-15 |
| SNORD19B | NMT2 | -0.3835 | 3.24E-15 |
| SNORD19B | MT2A | -0.3834 | 3.31E-15 |
| SNORD19B | C3orf23 | 0.3832 | 3.40E-15 |
| SNORD19B | COLEC12 | -0.3832 | 3.42E-15 |
| SNORD19B | PIK3C2B | 0.3832 | 3.43E-15 |
| SNORD19B | KIAA1543 | 0.3829 | 3.60E-15 |
| SNORD19B | SCN2B | -0.3828 | 3.63E-15 |
| SNORD19B | CYS1 | -0.3827 | 3.74E-15 |
| SNORD19B | LAYN | -0.3825 | 3.83E-15 |
| SNORD19B | FMO9P | 0.3824 | 3.90E-15 |
| SNORD19B | AK3L1 | -0.3824 | 3.94E-15 |
| SNORD19B | DECR1 | 0.3823 | 3.97E-15 |
| SNORD19B | RASA3 | -0.3823 | 3.97E-15 |
| SNORD19B | PODNL1 | -0.3822 | 4.08E-15 |
| SNORD19B | C21orf63 | -0.3821 | 4.15E-15 |
| SNORD19B | CACNA2D1 | -0.3821 | 4.17E-15 |
| SNORD19B | NTN1 | -0.3819 | 4.27E-15 |
| SNORD19B | VIPR1 | 0.3819 | 4.31E-15 |
| SNORD19B | ACTG2 | -0.3818 | 4.36E-15 |
| SNORD19B | CSGALNACT2 | -0.3818 | 4.36E-15 |
| SNORD19B | C22orf26 | -0.3816 | 4.53E-15 |
| SNORD19B | RNF128 | 0.3816 | 4.54E-15 |
| SNORD19B | WBSCR17 | -0.3816 | 4.54E-15 |
| SNORD19B | ISLR | -0.3815 | 4.64E-15 |
| SNORD19B | GEM | -0.3814 | 4.68E-15 |
| SNORD19B | LOC100188947 | 0.3814 | 4.70E-15 |
| SNORD19B | KDELC1 | -0.3813 | 4.74E-15 |
| SNORD19B | ACTB | -0.3811 | 4.92E-15 |
| SNORD19B | RPSAP52 | -0.3811 | 4.94E-15 |
| SNORD19B | SLAMF8 | -0.3811 | 4.95E-15 |
| SNORD19B | QARS | 0.3811 | 4.96E-15 |
| SNORD19B | TCP11L1 | -0.3811 | 4.98E-15 |
| SNORD19B | FILIP1L | -0.381 | 5.05E-15 |
| SNORD19B | CD300C | -0.381 | 5.06E-15 |
| SNORD19B | BACE1 | -0.3809 | 5.08E-15 |
| SNORD19B | SLC9A2 | 0.3808 | 5.19E-15 |
| SNORD19B | FBXO32 | -0.3808 | 5.21E-15 |
| SNORD19B | KCNIP3 | -0.3806 | 5.40E-15 |
| SNORD19B | ADAMTS6 | -0.3805 | 5.52E-15 |
| SNORD19B | DYRK3 | -0.3804 | 5.62E-15 |
| SNORD19B | SAMD4A | -0.3804 | 5.62E-15 |
| SNORD19B | BHMT | 0.3802 | 5.78E-15 |
| SNORD19B | ADAMTS16 | -0.3802 | 5.83E-15 |
| SNORD19B | LYPD6 | 0.3801 | 5.90E-15 |
| SNORD19B | CD14 | -0.3801 | 5.91E-15 |
| SNORD19B | ADAM12 | -0.3799 | 6.08E-15 |
| SNORD19B | C4A | -0.3798 | 6.20E-15 |
| SNORD19B | REEP6 | 0.3798 | 6.20E-15 |
| SNORD19B | GLT8D2 | -0.3798 | 6.26E-15 |
| SNORD19B | GUCA1A | -0.3797 | 6.33E-15 |
| SNORD19B | ADCY7 | -0.3797 | 6.35E-15 |
| SNORD19B | WARS | -0.3794 | 6.72E-15 |
| SNORD19B | CXorf21 | -0.3793 | 6.81E-15 |
| SNORD19B | VENTX | -0.3792 | 6.86E-15 |
| SNORD19B | ZYX | -0.3792 | 6.88E-15 |
| SNORD19B | CCDC64B | 0.3792 | 6.97E-15 |
| SNORD19B | DHRS11 | 0.3791 | 7.02E-15 |
| SNORD19B | FLJ45983 | 0.379 | 7.10E-15 |
| SNORD19B | MDGA1 | -0.379 | 7.20E-15 |
| SNORD19B | GOLT1A | 0.3789 | 7.31E-15 |
| SNORD19B | SIGLEC9 | -0.3788 | 7.40E-15 |
| SNORD19B | SERPINE2 | -0.3787 | 7.59E-15 |
| SNORD19B | PLEKHH1 | 0.3786 | 7.63E-15 |
| SNORD19B | GALNT10 | -0.3785 | 7.78E-15 |
| SNORD19B | DGKB | -0.3785 | 7.85E-15 |
| SNORD19B | DIRC1 | -0.3784 | 7.96E-15 |
| SNORD19B | DDX24 | -0.3784 | 7.98E-15 |
| SNORD19B | FMO1 | -0.3781 | 8.32E-15 |
| SNORD19B | SETBP1 | -0.3781 | 8.33E-15 |
| SNORD19B | FCGR2A | -0.378 | 8.59E-15 |
| SNORD19B | TSPAN4 | -0.3778 | 8.91E-15 |
| SNORD19B | RGS9 | -0.3777 | 8.93E-15 |
| SNORD19B | TMEM158 | -0.3777 | 8.98E-15 |
| SNORD19B | ITGAM | -0.3775 | 9.25E-15 |
| SNORD19B | HEMK1 | 0.3775 | 9.34E-15 |
| SNORD19B | NEGR1 | -0.3772 | 9.74E-15 |
| SNORD19B | LOC100129034 | -0.3772 | 9.79E-15 |
| SNORD19B | RNASE2 | -0.3772 | 9.85E-15 |
| SNORD19B | NEK6 | -0.3771 | 9.96E-15 |
| SNORD19B | TRAM2 | -0.377 | 1.01E-14 |
| SNORD19B | COL5A2 | -0.3769 | 1.03E-14 |
| SNORD19B | CES1 | -0.3769 | 1.04E-14 |
| SNORD19B | MKX | -0.3769 | 1.04E-14 |
| SNORD19B | NADSYN1 | 0.3769 | 1.04E-14 |
| SNORD19B | CCDC46 | -0.3768 | 1.06E-14 |
| SNORD19B | FGL2 | -0.3767 | 1.07E-14 |
| SNORD19B | FNDC1 | -0.3767 | 1.07E-14 |
| SNORD19B | STXBP2 | 0.3765 | 1.10E-14 |
| SNORD19B | TGFBR1 | -0.3765 | 1.11E-14 |
| SNORD19B | OSTM1 | -0.3764 | 1.13E-14 |
| SNORD19B | ASB5 | -0.3763 | 1.14E-14 |
| SNORD19B | PPAPDC3 | -0.3763 | 1.14E-14 |
| SNORD19B | PTCD3 | 0.3761 | 1.18E-14 |
| SNORD19B | SYT11 | -0.3758 | 1.25E-14 |
| SNORD19B | RGMA | -0.3757 | 1.27E-14 |
| SNORD19B | TRPS1 | -0.3756 | 1.31E-14 |
| SNORD19B | SH3GLB2 | 0.3753 | 1.36E-14 |
| SNORD19B | GGT6 | 0.3753 | 1.37E-14 |
| SNORD19B | GREM1 | -0.3752 | 1.39E-14 |
| SNORD19B | MYCL1 | 0.3751 | 1.41E-14 |
| SNORD19B | GPR34 | -0.375 | 1.44E-14 |
| SNORD19B | LIX1L | -0.375 | 1.44E-14 |
| SNORD19B | ERC1 | -0.3749 | 1.47E-14 |
| SNORD19B | CYBB | -0.3748 | 1.49E-14 |
| SNORD19B | PLEKHG6 | 0.3747 | 1.52E-14 |
| SNORD19B | SPI1 | -0.3747 | 1.52E-14 |
| SNORD19B | IFFO1 | -0.3747 | 1.53E-14 |
| SNORD19B | TPM1 | -0.3746 | 1.53E-14 |
| SNORD19B | FLRT2 | -0.3746 | 1.55E-14 |
| SNORD19B | RASGRP4 | -0.3744 | 1.59E-14 |
| SNORD19B | RRAD | -0.3744 | 1.59E-14 |
| SNORD19B | AIF1 | -0.3744 | 1.60E-14 |
| SNORD19B | WISP1 | -0.3743 | 1.61E-14 |
| SNORD19B | SCN3B | -0.3743 | 1.63E-14 |
| SNORD19B | PDZRN3 | -0.3742 | 1.64E-14 |
| SNORD19B | ALDH1L2 | -0.3742 | 1.65E-14 |
| SNORD19B | CDC25B | -0.3742 | 1.65E-14 |
| SNORD19B | PGM2L1 | -0.374 | 1.70E-14 |
| SNORD19B | CERK | -0.374 | 1.71E-14 |
| SNORD19B | TEAD4 | -0.3739 | 1.73E-14 |
| SNORD19B | MFI2 | -0.3736 | 1.82E-14 |
| SNORD19B | KITLG | -0.3735 | 1.87E-14 |
| SNORD19B | CRB3 | 0.3732 | 1.95E-14 |
| SNORD19B | CAV1 | -0.3731 | 1.99E-14 |
| SNORD19B | DUSP3 | -0.3729 | 2.08E-14 |
| SNORD19B | KCNJ12 | -0.3726 | 2.18E-14 |
| SNORD19B | FGFR1 | -0.3724 | 2.24E-14 |
| SNORD19B | PTGER3 | -0.3724 | 2.25E-14 |
| SNORD19B | SERPINA3 | -0.3721 | 2.36E-14 |
| SNORD19B | LY96 | -0.3721 | 2.37E-14 |
| SNORD19B | GAB3 | -0.372 | 2.40E-14 |
| SNORD19B | HPS5 | -0.372 | 2.41E-14 |
| SNORD19B | DCN | -0.3719 | 2.44E-14 |
| SNORD19B | PXDN | -0.3719 | 2.45E-14 |
| SNORD19B | SFRP4 | -0.3719 | 2.46E-14 |
| SNORD19B | NPRL2 | 0.3717 | 2.52E-14 |
| SNORD19B | LRFN2 | 0.3717 | 2.53E-14 |
| SNORD19B | FCGR1A | -0.3715 | 2.63E-14 |
| SNORD19B | RNASE6 | -0.3715 | 2.63E-14 |
| SNORD19B | SLC44A3 | 0.3714 | 2.65E-14 |
| SNORD19B | HEG1 | -0.3714 | 2.68E-14 |
| SNORD19B | MAP1A | -0.3714 | 2.69E-14 |
| SNORD19B | STXBP1 | -0.3712 | 2.74E-14 |
| SNORD19B | OSCAR | -0.3712 | 2.75E-14 |
| SNORD19B | KCNMA1 | -0.3712 | 2.76E-14 |
| SNORD19B | C3AR1 | -0.3712 | 2.79E-14 |
| SNORD19B | COL1A1 | -0.3712 | 2.79E-14 |
| SNORD19B | FMNL3 | -0.3708 | 2.96E-14 |
| SNORD19B | DOCK11 | -0.3708 | 2.97E-14 |
| SNORD19B | ARHGAP27 | 0.3707 | 2.99E-14 |
| SNORD19B | TNFSF4 | -0.3706 | 3.09E-14 |
| SNORD19B | COL6A3 | -0.3705 | 3.14E-14 |
| SNORD19B | COL5A1 | -0.3704 | 3.16E-14 |
| SNORD19B | PLA2G4C | -0.37 | 3.39E-14 |
| SNORD19B | NOD2 | -0.37 | 3.40E-14 |
| SNORD19B | SCARF2 | -0.3699 | 3.44E-14 |
| SNORD19B | LOC100192378 | -0.3699 | 3.46E-14 |
| SNORD19B | GAS6 | -0.3698 | 3.49E-14 |
| SNORD19B | ANXA2 | -0.3697 | 3.55E-14 |
| SNORD19B | MSC | -0.3697 | 3.57E-14 |
| SNORD19B | VIM | -0.3697 | 3.57E-14 |
| SNORD19B | CISD1 | -0.3697 | 3.59E-14 |
| SNORD19B | P2RX1 | -0.3695 | 3.67E-14 |
| SNORD19B | MGC45800 | -0.3695 | 3.68E-14 |
| SNORD19B | ANXA2P2 | -0.3695 | 3.72E-14 |
| SNORD19B | PLA2G5 | -0.3693 | 3.83E-14 |
| SNORD19B | PDLIM4 | -0.3692 | 3.88E-14 |
| SNORD19B | GLT8D1 | 0.3691 | 3.94E-14 |
| SNORD19B | HEPH | -0.3691 | 3.97E-14 |
| SNORD19B | PCNX | -0.369 | 4.03E-14 |
| SNORD19B | FCGR1C | -0.369 | 4.04E-14 |
| SNORD19B | LAMA4 | -0.369 | 4.05E-14 |
| SNORD19B | LRRC15 | -0.3689 | 4.07E-14 |
| SNORD19B | NTNG2 | -0.3689 | 4.10E-14 |
| SNORD19B | GLIS1 | -0.3688 | 4.13E-14 |
| SNORD19B | LRRK2 | -0.3688 | 4.16E-14 |
| SNORD19B | TSEN2 | 0.3688 | 4.18E-14 |
| SNORD19B | PTGDR | -0.3687 | 4.20E-14 |
| SNORD19B | ALPK2 | -0.3687 | 4.22E-14 |
| SNORD19B | FCGR3A | -0.3687 | 4.24E-14 |
| SNORD19B | HAND2 | -0.3685 | 4.37E-14 |
| SNORD19B | LOC100130872 | -0.3685 | 4.40E-14 |
| SNORD19B | FOXH1 | 0.3683 | 4.50E-14 |
| SNORD19B | ZEB1 | -0.3683 | 4.51E-14 |
| SNORD19B | ACOT9 | -0.3683 | 4.52E-14 |
| SNORD19B | B4GALNT1 | -0.368 | 4.74E-14 |
| SNORD19B | CLEC4A | -0.3679 | 4.83E-14 |
| SNORD19B | PIK3R6 | -0.3677 | 5.00E-14 |
| SNORD19B | LRRC25 | -0.3676 | 5.08E-14 |
| SNORD19B | FGF5 | -0.3675 | 5.15E-14 |
| SNORD19B | FHL1 | -0.3675 | 5.19E-14 |
| SNORD19B | VSIG4 | -0.3675 | 5.21E-14 |
| SNORD19B | LILRB2 | -0.3674 | 5.27E-14 |
| SNORD19B | DHRS2 | 0.3673 | 5.32E-14 |
| SNORD19B | NBLA00301 | -0.3673 | 5.37E-14 |
| SNORD19B | OR7E91P | 0.3672 | 5.41E-14 |
| SNORD19B | GRHL3 | 0.3672 | 5.43E-14 |
| SNORD19B | ATXN1 | -0.3671 | 5.51E-14 |
| SNORD19B | DPYSL2 | -0.3671 | 5.52E-14 |
| SNORD19B | CLIP4 | -0.367 | 5.66E-14 |
| SNORD19B | DOCK10 | -0.367 | 5.67E-14 |
| SNORD19B | ILK | -0.367 | 5.68E-14 |
| SNORD19B | BATF3 | -0.3668 | 5.86E-14 |
| SNORD19B | SAV1 | -0.3668 | 5.87E-14 |
| SNORD19B | CCL21 | -0.3666 | 5.99E-14 |
| SNORD19B | TFE3 | -0.3665 | 6.16E-14 |
| SNORD19B | C7orf10 | -0.3664 | 6.26E-14 |
| SNORD19B | SLC22A2 | -0.3663 | 6.31E-14 |
| SNORD19B | EMILIN1 | -0.3663 | 6.32E-14 |
| SNORD19B | FAM70A | -0.3661 | 6.54E-14 |
| SNORD19B | CDH2 | -0.366 | 6.67E-14 |
| SNORD19B | FAM69C | -0.366 | 6.67E-14 |
| SNORD19B | FYB | -0.366 | 6.70E-14 |
| SNORD19B | TGM2 | -0.3659 | 6.76E-14 |
| SNORD19B | CTSB | -0.3659 | 6.82E-14 |
| SNORD19B | L1CAM | -0.3658 | 6.95E-14 |
| SNORD19B | ITGB2 | -0.3656 | 7.09E-14 |
| SNORD19B | LOC653653 | -0.3653 | 7.50E-14 |
| SNORD19B | ACTC1 | -0.3652 | 7.67E-14 |
| SNORD19B | RPLP0P2 | -0.3651 | 7.77E-14 |
| SNORD19B | BTK | -0.3651 | 7.82E-14 |
| SNORD19B | NUAK2 | -0.3649 | 7.97E-14 |
| SNORD19B | NUDT10 | -0.3649 | 7.99E-14 |
| SNORD19B | ANTXR1 | -0.3649 | 8.04E-14 |
| SNORD19B | TMEM106A | -0.3649 | 8.06E-14 |
| SNORD19B | COL3A1 | -0.3648 | 8.19E-14 |
| SNORD19B | BMP1 | -0.3647 | 8.33E-14 |
| SNORD19B | FN1 | -0.3646 | 8.48E-14 |
| SNORD19B | KIFC2 | 0.3645 | 8.58E-14 |
| SNORD19B | C6orf114 | -0.3644 | 8.73E-14 |
| SNORD19B | CD53 | -0.3643 | 8.88E-14 |
| SNORD19B | TSPAN6 | 0.3642 | 9.01E-14 |
| SNORD19B | ZCCHC24 | -0.3642 | 9.05E-14 |
| SNORD19B | ITGAV | -0.3641 | 9.15E-14 |
| SNORD19B | ANGPTL2 | -0.3638 | 9.62E-14 |
| SNORD19B | KLF13 | -0.3638 | 9.65E-14 |
| SNORD19B | LOC100126784 | -0.3637 | 9.86E-14 |
| SNORD19B | RECK | -0.3636 | 9.94E-14 |
| SNORD19B | TGFB2 | -0.3636 | 1.00E-13 |
| SNORD19B | ABHD12 | 0.3633 | 1.04E-13 |
| SNORD19B | NSUN6 | 0.3632 | 1.06E-13 |
| SNORD19B | HCFC2 | -0.3632 | 1.07E-13 |
| SNORD19B | KCNJ8 | -0.3631 | 1.08E-13 |
| SNORD19B | MITF | -0.3631 | 1.08E-13 |
| SNORD19B | CYP4F12 | 0.3629 | 1.11E-13 |
| SNORD19B | MNDA | -0.3628 | 1.13E-13 |
| SNORD19B | TSHZ3 | -0.3627 | 1.15E-13 |
| SNORD19B | AMOTL1 | -0.3625 | 1.19E-13 |
| SNORD19B | LILRA6 | -0.3625 | 1.19E-13 |
| SNORD19B | DOK2 | -0.3623 | 1.24E-13 |
| SNORD19B | NCRNA00152 | -0.362 | 1.30E-13 |
| SNORD19B | TNFSF13B | -0.362 | 1.30E-13 |
| SNORD19B | FGF7 | -0.3619 | 1.31E-13 |
| SNORD19B | ADAMTS2 | -0.3619 | 1.32E-13 |
| SNORD19B | CD300A | -0.3618 | 1.34E-13 |
| SNORD19B | CYP2J2 | 0.3617 | 1.36E-13 |
| SNORD19B | BVES | -0.3617 | 1.37E-13 |
| SNORD19B | AGAP11 | 0.3616 | 1.39E-13 |
| SNORD19B | OVGP1 | 0.3615 | 1.41E-13 |
| SNORD19B | NEDD4L | 0.3615 | 1.42E-13 |
| SNORD19B | LMO1 | -0.3613 | 1.45E-13 |
| SNORD19B | MSR1 | -0.361 | 1.52E-13 |
| SNORD19B | CILP | -0.3609 | 1.56E-13 |
| SNORD19B | ADAMTS12 | -0.3608 | 1.59E-13 |
| SNORD19B | AGPAT4 | -0.3608 | 1.59E-13 |
| SNORD19B | ARSB | -0.3608 | 1.59E-13 |
| SNORD19B | C20orf103 | -0.3607 | 1.61E-13 |
| SNORD19B | SH3YL1 | 0.3607 | 1.62E-13 |
| SNORD19B | FAM126A | -0.3606 | 1.64E-13 |
| SNORD19B | MYADM | -0.3606 | 1.64E-13 |
| SNORD19B | OXSM | 0.3605 | 1.66E-13 |
| SNORD19B | ARHGEF17 | -0.3604 | 1.69E-13 |
| SNORD19B | FCER1G | -0.3604 | 1.70E-13 |
| SNORD19B | ANTXR2 | -0.3603 | 1.73E-13 |
| SNORD19B | TNFSF14 | -0.3602 | 1.75E-13 |
| SNORD19B | CADM3 | -0.3601 | 1.79E-13 |
| SNORD19B | TCF4 | -0.3593 | 2.02E-13 |
| SNORD19B | CACNA1D | 0.3592 | 2.05E-13 |
| SNORD19B | SLC25A38 | 0.3592 | 2.07E-13 |
| SNORD19B | COL1A2 | -0.3591 | 2.09E-13 |
| SNORD19B | RGS2 | -0.3591 | 2.09E-13 |
| SNORD19B | MFAP3L | 0.359 | 2.12E-13 |
| SNORD19B | LILRA2 | -0.3588 | 2.19E-13 |
| SNORD19B | SLC29A3 | 0.3588 | 2.19E-13 |
| SNORD19B | CEBPA | 0.3588 | 2.20E-13 |
| SNORD19B | SGMS2 | -0.3588 | 2.20E-13 |
| SNORD19B | LIMD1 | 0.3587 | 2.23E-13 |
| SNORD19B | PDPN | -0.3587 | 2.25E-13 |
| SNORD19B | BOC | -0.3586 | 2.29E-13 |
| SNORD19B | CCDC88A | -0.3584 | 2.34E-13 |
| SNORD19B | ACVR2B | 0.3584 | 2.35E-13 |
| SNORD19B | TRAF4 | 0.3584 | 2.37E-13 |
| SNORD19B | MBTD1 | 0.3583 | 2.38E-13 |
| SNORD19B | LAT2 | -0.3582 | 2.42E-13 |
| SNORD19B | DCLK1 | -0.3582 | 2.45E-13 |
| SNORD19B | RHBG | 0.3581 | 2.45E-13 |
| SNORD19B | SLIT2 | -0.358 | 2.49E-13 |
| SNORD19B | RAB11FIP4 | 0.358 | 2.50E-13 |
| SNORD19B | FCGR1B | -0.3578 | 2.60E-13 |
| SNORD19B | SLFN11 | -0.3577 | 2.62E-13 |
| SNORD19B | HSD17B2 | 0.3577 | 2.63E-13 |
| SNORD19B | IDH1 | 0.3577 | 2.65E-13 |
| SNORD19B | ENGASE | 0.3577 | 2.66E-13 |
| SNORD19B | HIP1R | 0.3576 | 2.69E-13 |
| SNORD19B | PLAUR | -0.3573 | 2.79E-13 |
| SNORD19B | C1orf126 | 0.3572 | 2.84E-13 |
| SNORD19B | SNX31 | 0.3572 | 2.85E-13 |
| SNORD19B | C2orf15 | 0.3572 | 2.87E-13 |
| SNORD19B | ST6GALNAC5 | -0.3571 | 2.90E-13 |
| SNORD19B | MYO1G | -0.3568 | 3.04E-13 |
| SNORD19B | CASQ2 | -0.3566 | 3.16E-13 |
| SNORD19B | HS3ST3B1 | -0.3566 | 3.16E-13 |
| SNORD19B | CD33 | -0.3565 | 3.21E-13 |
| SNORD19B | VMO1 | -0.3565 | 3.22E-13 |
| SNORD19B | ARNTL | -0.3564 | 3.24E-13 |
| SNORD19B | LY86 | -0.3564 | 3.24E-13 |
| SNORD19B | TYROBP | -0.3564 | 3.26E-13 |
| SNORD19B | CD82 | -0.3563 | 3.32E-13 |
| SNORD19B | LOC284837 | 0.3563 | 3.34E-13 |
| SNORD19B | LPAR2 | 0.3562 | 3.36E-13 |
| SNORD19B | KANK4 | -0.3562 | 3.38E-13 |
| SNORD19B | CDK6 | -0.3562 | 3.39E-13 |
| SNORD19B | ACOX1 | 0.3561 | 3.40E-13 |
| SNORD19B | KIRREL | -0.356 | 3.48E-13 |
| SNORD19B | PANX1 | -0.356 | 3.50E-13 |
| SNORD19B | NTRK1 | -0.3558 | 3.59E-13 |
| SNORD19B | PHF19 | -0.3558 | 3.62E-13 |
| SNORD19B | LGALS1 | -0.3556 | 3.69E-13 |
| SNORD19B | EDNRA | -0.3556 | 3.71E-13 |
| SNORD19B | MYO5A | -0.3555 | 3.75E-13 |
| SNORD19B | LST1 | -0.3554 | 3.80E-13 |
| SNORD19B | SCNN1B | 0.3554 | 3.83E-13 |
| SNORD19B | PRICKLE1 | -0.3552 | 3.94E-13 |
| SNORD19B | WISP2 | -0.3551 | 4.03E-13 |
| SNORD19B | NUDT11 | -0.355 | 4.06E-13 |
| SNORD19B | PRKG1 | -0.355 | 4.09E-13 |
| SNORD19B | UPK1A | 0.355 | 4.09E-13 |
| SNORD19B | CPXM1 | -0.3549 | 4.15E-13 |
| SNORD19B | DTX1 | -0.3549 | 4.16E-13 |
| SNORD19B | ANXA13 | -0.3549 | 4.18E-13 |
| SNORD19B | RAB15 | 0.3548 | 4.19E-13 |
| SNORD19B | PAPSS2 | -0.3547 | 4.31E-13 |
| SNORD19B | CPXM2 | -0.3546 | 4.33E-13 |
| SNORD19B | NR2F6 | 0.3546 | 4.33E-13 |
| SNORD19B | HSPA12A | -0.3545 | 4.40E-13 |
| SNORD19B | ENTPD1 | -0.3545 | 4.43E-13 |
| SNORD19B | LMOD1 | -0.3544 | 4.49E-13 |
| SNORD19B | PDGFRB | -0.3543 | 4.54E-13 |
| SNORD19B | SYDE1 | -0.3543 | 4.57E-13 |
| SNORD19B | NEFH | -0.3542 | 4.62E-13 |
| SNORD19B | ENPP3 | -0.3542 | 4.63E-13 |
| SNORD19B | WT1 | -0.3542 | 4.65E-13 |
| SNORD19B | C1orf162 | -0.3542 | 4.66E-13 |
| SNORD19B | GSN | -0.3542 | 4.67E-13 |
| SNORD19B | LOC283267 | -0.354 | 4.81E-13 |
| SNORD19B | THRB | 0.3536 | 5.09E-13 |
| SNORD19B | NIPSNAP1 | 0.3536 | 5.11E-13 |
| SNORD19B | SLC22A5 | 0.3535 | 5.18E-13 |
| SNORD19B | CUEDC1 | -0.3533 | 5.34E-13 |
| SNORD19B | ECM2 | -0.3533 | 5.38E-13 |
| SNORD19B | ZNF521 | -0.3533 | 5.38E-13 |
| SNORD19B | HGF | -0.3532 | 5.47E-13 |
| SNORD19B | MXRA8 | -0.3532 | 5.48E-13 |
| SNORD19B | CHST3 | -0.3531 | 5.54E-13 |
| SNORD19B | ERBB3 | 0.353 | 5.67E-13 |
| SNORD19B | ADRB3 | -0.3529 | 5.70E-13 |
| SNORD19B | NXN | -0.3528 | 5.83E-13 |
| SNORD19B | TPP1 | -0.3527 | 5.95E-13 |
| SNORD19B | C3orf34 | -0.3526 | 5.97E-13 |
| SNORD19B | IFI16 | -0.3524 | 6.18E-13 |
| SNORD19B | PTGFR | -0.3524 | 6.23E-13 |
| SNORD19B | LAIR1 | -0.3524 | 6.25E-13 |
| SNORD19B | TNFRSF8 | -0.3523 | 6.33E-13 |
| SNORD19B | EVI2A | -0.3523 | 6.34E-13 |
| SNORD19B | HAND1 | -0.3522 | 6.41E-13 |
| SNORD19B | JAK2 | -0.3522 | 6.41E-13 |
| SNORD19B | CHADL | 0.352 | 6.65E-13 |
| SNORD19B | CD44 | -0.3518 | 6.79E-13 |
| SNORD19B | NLRC5 | -0.3518 | 6.82E-13 |
| SNORD19B | PHLPP1 | 0.3518 | 6.83E-13 |
| SNORD19B | ATP8B1 | 0.3517 | 6.89E-13 |
| SNORD19B | FPR1 | -0.3517 | 6.92E-13 |
| SNORD19B | C10orf116 | 0.3517 | 6.94E-13 |
| SNORD19B | CD99L2 | -0.3517 | 6.97E-13 |
| SNORD19B | C1QC | -0.3516 | 7.03E-13 |
| SNORD19B | PNMAL2 | -0.3516 | 7.04E-13 |
| SNORD19B | RASSF8 | -0.3516 | 7.08E-13 |
| SNORD19B | ABCD3 | 0.3515 | 7.11E-13 |
| SNORD19B | PPAPDC1A | -0.3513 | 7.39E-13 |
| SNORD19B | PCYT2 | 0.3512 | 7.52E-13 |
| SNORD19B | CLIP3 | -0.3511 | 7.61E-13 |
| SNORD19B | LOC728264 | -0.3511 | 7.67E-13 |
| SNORD19B | ACTBL2 | -0.3511 | 7.69E-13 |
| SNORD19B | GPR133 | -0.351 | 7.77E-13 |
| SNORD19B | TRIT1 | 0.351 | 7.81E-13 |
| SNORD19B | ITGB3 | -0.3508 | 7.97E-13 |
| SNORD19B | GYPC | -0.3506 | 8.23E-13 |
| SNORD19B | TMCC3 | -0.3506 | 8.25E-13 |
| SNORD19B | P2RX7 | -0.3505 | 8.37E-13 |
| SNORD19B | TMEM97 | 0.3505 | 8.39E-13 |
| SNORD19B | EIF5A2 | -0.3505 | 8.46E-13 |
| SNORD19B | PYGB | -0.3503 | 8.60E-13 |
| SNORD19B | POU5F1B | 0.3503 | 8.65E-13 |
| SNORD19B | ECE1 | -0.3503 | 8.69E-13 |
| SNORD19B | LOC221442 | 0.3503 | 8.73E-13 |
| SNORD19B | C20orf141 | -0.3502 | 8.75E-13 |
| SNORD19B | CD86 | -0.3502 | 8.80E-13 |
| SNORD19B | MAPRE2 | -0.3502 | 8.81E-13 |
| SNORD19B | FPR3 | -0.3501 | 8.95E-13 |
| SNORD19B | MAFB | -0.3501 | 8.96E-13 |
| SNORD19B | TMCC2 | -0.3501 | 9.02E-13 |
| SNORD19B | GCNT1 | -0.35 | 9.15E-13 |
| SNORD19B | IL13RA1 | -0.3499 | 9.26E-13 |
| SNORD19B | GPR135 | -0.3498 | 9.34E-13 |
| SNORD19B | HDAC9 | -0.3498 | 9.42E-13 |
| SNORD19B | CPEB1 | -0.3497 | 9.60E-13 |
| SNORD19B | FGF1 | -0.3496 | 9.73E-13 |
| SNORD19B | SSH3 | 0.3495 | 9.84E-13 |
| SNORD19B | GAS7 | -0.3494 | 9.95E-13 |
| SNORD19B | NINJ2 | -0.3494 | 9.96E-13 |
| SNORD19B | FILIP1 | -0.3493 | 1.02E-12 |
| SNORD19B | MT1X | -0.3493 | 1.02E-12 |
| SNORD19B | ASAM | -0.3491 | 1.05E-12 |
| SNORD19B | CNGA1 | 0.3489 | 1.08E-12 |
| SNORD19B | HPGD | 0.3489 | 1.08E-12 |
| SNORD19B | SH2D5 | -0.3489 | 1.08E-12 |
| SNORD19B | ASCC2 | 0.3487 | 1.11E-12 |
| SNORD19B | CYP4F8 | 0.3488 | 1.11E-12 |
| SNORD19B | SHOX2 | -0.3487 | 1.11E-12 |
| SNORD19B | KCNE4 | -0.3487 | 1.12E-12 |
| SNORD19B | ZHX3 | -0.3487 | 1.12E-12 |
| SNORD19B | OGN | -0.3485 | 1.15E-12 |
| SNORD19B | S100A10 | -0.3484 | 1.18E-12 |
| SNORD19B | WHSC2 | 0.3484 | 1.18E-12 |
| SNORD19B | C6orf122 | -0.3482 | 1.21E-12 |
| SNORD19B | C1QTNF1 | -0.3482 | 1.22E-12 |
| SNORD19B | IRAK3 | -0.348 | 1.24E-12 |
| SNORD19B | TEP1 | -0.348 | 1.24E-12 |
| SNORD19B | SLC30A4 | -0.3478 | 1.28E-12 |
| SNORD19B | VSNL1 | -0.3478 | 1.28E-12 |
| SNORD19B | PRR16 | -0.3478 | 1.29E-12 |
| SNORD19B | AKT3 | -0.3477 | 1.30E-12 |
| SNORD19B | C15orf17 | 0.3478 | 1.30E-12 |
| SNORD19B | INHBA | -0.3477 | 1.31E-12 |
| SNORD19B | CAPN5 | 0.3474 | 1.38E-12 |
| SNORD19B | TNFRSF6B | -0.3473 | 1.39E-12 |
| SNORD19B | ERBB2 | 0.3473 | 1.40E-12 |
| SNORD19B | ACY1 | 0.3472 | 1.41E-12 |
| SNORD19B | FMOD | -0.3472 | 1.41E-12 |
| SNORD19B | DUSP22 | -0.3471 | 1.44E-12 |
| SNORD19B | LOC202781 | 0.3471 | 1.44E-12 |
| SNORD19B | ANKS1B | -0.3471 | 1.45E-12 |
| SNORD19B | MLXIP | -0.347 | 1.46E-12 |
| SNORD19B | SEC23A | -0.3468 | 1.50E-12 |
| SNORD19B | CTDSP2 | -0.3467 | 1.52E-12 |
| SNORD19B | GPR37 | -0.3467 | 1.53E-12 |
| SNORD19B | GNG2 | -0.3467 | 1.54E-12 |
| SNORD19B | OGFRL1 | -0.3466 | 1.54E-12 |
| SNORD19B | HTR2A | -0.3466 | 1.56E-12 |
| SNORD19B | PDZRN4 | -0.3466 | 1.56E-12 |
| SNORD19B | SCRG1 | -0.3465 | 1.57E-12 |
| SNORD19B | CMKLR1 | -0.3464 | 1.60E-12 |
| SNORD19B | HAVCR2 | -0.3463 | 1.64E-12 |
| SNORD19B | PCSK1 | -0.3463 | 1.64E-12 |
| SNORD19B | MPEG1 | -0.3462 | 1.65E-12 |
| SNORD19B | GPR84 | -0.3462 | 1.66E-12 |
| SNORD19B | ADAMTS10 | -0.3462 | 1.67E-12 |
| SNORD19B | LILRA5 | -0.3461 | 1.67E-12 |
| SNORD19B | RIN2 | -0.3461 | 1.67E-12 |
| SNORD19B | BGN | -0.3461 | 1.68E-12 |
| SNORD19B | VCAN | -0.3461 | 1.68E-12 |
| SNORD19B | CTSK | -0.346 | 1.71E-12 |
| SNORD19B | LOC100130933 | 0.3459 | 1.74E-12 |
| SNORD19B | NACC2 | -0.3459 | 1.74E-12 |
| SNORD19B | CTGF | -0.3456 | 1.81E-12 |
| SNORD19B | EXT2 | -0.3455 | 1.85E-12 |
| SNORD19B | GLG1 | -0.3453 | 1.91E-12 |
| SNORD19B | SPARC | -0.3451 | 1.98E-12 |
| SNORD19B | C5orf4 | -0.345 | 1.99E-12 |
| SNORD19B | MRO | -0.3448 | 2.06E-12 |
| SNORD19B | NPAS2 | 0.3448 | 2.06E-12 |
| SNORD19B | LIMS2 | -0.3447 | 2.09E-12 |
| SNORD19B | GPD1L | 0.3446 | 2.13E-12 |
| SNORD19B | DGAT2 | 0.3445 | 2.16E-12 |
| SNORD19B | LOC285830 | -0.3443 | 2.22E-12 |
| SNORD19B | SLC14A1 | 0.3443 | 2.22E-12 |
| SNORD19B | TNFAIP8L2 | -0.3443 | 2.23E-12 |
| SNORD19B | LOC541471 | -0.3443 | 2.24E-12 |
| SNORD19B | UBLCP1 | -0.3442 | 2.24E-12 |
| SNORD19B | SRCIN1 | 0.3442 | 2.25E-12 |
| SNORD19B | ABHD14A | 0.3441 | 2.28E-12 |
| SNORD19B | BCL2A1 | -0.3441 | 2.30E-12 |
| SNORD19B | NCAM2 | -0.344 | 2.32E-12 |
| SNORD19B | ATL3 | -0.344 | 2.34E-12 |
| SNORD19B | PTPN22 | -0.3439 | 2.35E-12 |
| SNORD19B | THRA | -0.3439 | 2.38E-12 |
| SNORD19B | NCK1 | -0.3438 | 2.39E-12 |
| SNORD19B | PLXNC1 | -0.3438 | 2.41E-12 |
| SNORD19B | ANGPTL1 | -0.3437 | 2.44E-12 |
| SNORD19B | CD209 | -0.3437 | 2.44E-12 |
| SNORD19B | OTUD1 | -0.3437 | 2.45E-12 |
| SNORD19B | NCF1 | -0.3435 | 2.52E-12 |
| SNORD19B | SLC25A10 | 0.3435 | 2.53E-12 |
| SNORD19B | PRR5L | -0.3434 | 2.57E-12 |
| SNORD19B | FZD8 | -0.3433 | 2.60E-12 |
| SNORD19B | CXCL12 | -0.3432 | 2.65E-12 |
| SNORD19B | RUNX3 | -0.3432 | 2.65E-12 |
| SNORD19B | HDAC4 | -0.343 | 2.70E-12 |
| SNORD19B | MEF2A | -0.343 | 2.71E-12 |
| SNORD19B | ADAMTS15 | -0.343 | 2.72E-12 |
| SNORD19B | IFITM1 | -0.343 | 2.73E-12 |
| SNORD19B | CYP4F22 | 0.3429 | 2.76E-12 |
| SNORD19B | NIN | -0.3428 | 2.80E-12 |
| SNORD19B | TUBA1A | -0.3428 | 2.80E-12 |
| SNORD19B | TINF2 | -0.3428 | 2.82E-12 |
| SNORD19B | SAMSN1 | -0.3428 | 2.83E-12 |
| SNORD19B | AMOTL2 | -0.3427 | 2.87E-12 |
| SNORD19B | PCSK5 | -0.3426 | 2.91E-12 |
| SNORD19B | TLL1 | -0.3425 | 2.93E-12 |
| SNORD19B | FUT9 | 0.3425 | 2.96E-12 |
| SNORD19B | FAM78A | -0.3424 | 2.98E-12 |
| SNORD19B | HLF | -0.3424 | 2.99E-12 |
| SNORD19B | SRC | 0.3424 | 2.99E-12 |
| SNORD19B | UCHL1 | -0.3421 | 3.13E-12 |
| SNORD19B | NLRP3 | -0.3421 | 3.14E-12 |
| SNORD19B | RBM24 | -0.3421 | 3.14E-12 |
| SNORD19B | COL18A1 | -0.342 | 3.20E-12 |
| SNORD19B | ZNF799 | 0.342 | 3.20E-12 |
| SNORD19B | LDHA | -0.3419 | 3.23E-12 |
| SNORD19B | IL15 | -0.3418 | 3.29E-12 |
| SNORD19B | C1QA | -0.3418 | 3.30E-12 |
| SNORD19B | GRB7 | 0.3417 | 3.35E-12 |
| SNORD19B | SLC39A14 | -0.3416 | 3.37E-12 |
| SNORD19B | COL5A3 | -0.3416 | 3.38E-12 |
| SNORD19B | HPSE | -0.3416 | 3.41E-12 |
| SNORD19B | SELPLG | -0.3414 | 3.46E-12 |
| SNORD19B | GBP5 | -0.3414 | 3.47E-12 |
| SNORD19B | KLF9 | -0.3414 | 3.49E-12 |
| SNORD19B | MS4A6A | -0.3414 | 3.50E-12 |
| SNORD19B | NT5DC3 | -0.3414 | 3.50E-12 |
| SNORD19B | RNF19B | -0.3414 | 3.51E-12 |
| SNORD19B | HSPB2 | -0.3413 | 3.55E-12 |
| SNORD19B | EVPL | 0.3413 | 3.56E-12 |
| SNORD19B | MST1P2 | 0.3413 | 3.56E-12 |
| SNORD19B | ATP10A | -0.3412 | 3.57E-12 |
| SNORD19B | KLHDC7A | 0.3412 | 3.60E-12 |
| SNORD19B | PID1 | -0.3411 | 3.65E-12 |
| SNORD19B | FBXL7 | -0.3411 | 3.66E-12 |
| SNORD19B | SETMAR | 0.3411 | 3.67E-12 |
| SNORD19B | ENPP1 | -0.341 | 3.70E-12 |
| SNORD19B | C8orf55 | 0.3409 | 3.77E-12 |
| SNORD19B | GPR77 | -0.3408 | 3.80E-12 |
| SNORD19B | IFI30 | -0.3407 | 3.90E-12 |
| SNORD19B | RNF217 | -0.3407 | 3.90E-12 |
| SNORD19B | WFDC13 | 0.3407 | 3.91E-12 |
| SNORD19B | LILRB3 | -0.3403 | 4.16E-12 |
| SNORD19B | LATS2 | -0.3402 | 4.19E-12 |
| SNORD19B | SECTM1 | -0.3402 | 4.20E-12 |
| SNORD19B | PIK3R5 | -0.3401 | 4.23E-12 |
| SNORD19B | PSCA | 0.3401 | 4.27E-12 |
| SNORD19B | CD84 | -0.3401 | 4.28E-12 |
| SNORD19B | DNAL4 | 0.34 | 4.31E-12 |
| SNORD19B | C3orf57 | 0.34 | 4.33E-12 |
| SNORD19B | TLR8 | -0.34 | 4.33E-12 |
| SNORD19B | LTBP2 | -0.3398 | 4.48E-12 |
| SNORD19B | C20orf200 | -0.3397 | 4.50E-12 |
| SNORD19B | F8 | -0.3396 | 4.56E-12 |
| SNORD19B | ESRP1 | 0.3396 | 4.60E-12 |
| SNORD19B | ZNF385B | -0.3396 | 4.61E-12 |
| SNORD19B | KRTAP5 | 0.3394 | 4.71E-12 |
| SNORD19B | PTGFRN | -0.3393 | 4.81E-12 |
| SNORD19B | DSE | -0.3393 | 4.84E-12 |
| SNORD19B | TNIP3 | -0.3393 | 4.84E-12 |
| SNORD19B | SIGLEC7 | -0.339 | 5.03E-12 |
| SNORD19B | PLEK | -0.3389 | 5.09E-12 |
| SNORD19B | COL10A1 | -0.3388 | 5.16E-12 |
| SNORD19B | KLF10 | -0.3387 | 5.24E-12 |
| SNORD19B | CDH11 | -0.3387 | 5.25E-12 |
| SNORD19B | MAMDC2 | -0.3387 | 5.29E-12 |
| SNORD19B | SOD2 | -0.3386 | 5.32E-12 |
| SNORD19B | ZNF440 | 0.3386 | 5.32E-12 |
| SNORD19B | C1orf70 | -0.3385 | 5.41E-12 |
| SNORD19B | ZNF69 | 0.3385 | 5.41E-12 |
| SNORD19B | CAV2 | -0.3384 | 5.54E-12 |
| SNORD19B | GNG12 | -0.3384 | 5.55E-12 |
| SNORD19B | CD163 | -0.3383 | 5.60E-12 |
| SNORD19B | GPR155 | -0.3383 | 5.62E-12 |
| SNORD19B | IGFBP6 | -0.3383 | 5.64E-12 |
| SNORD19B | RPL29 | 0.3382 | 5.67E-12 |
| SNORD19B | FBXO16 | 0.338 | 5.86E-12 |
| SNORD19B | ESRP2 | 0.3379 | 6.00E-12 |
| SNORD19B | ZNF443 | 0.3378 | 6.01E-12 |
| SNORD19B | TM7SF2 | 0.3378 | 6.02E-12 |
| SNORD19B | NFAM1 | -0.3377 | 6.12E-12 |
| SNORD19B | ARHGAP9 | -0.3377 | 6.17E-12 |
| SNORD19B | C5AR1 | -0.3376 | 6.24E-12 |
| SNORD19B | NTRK2 | -0.3376 | 6.24E-12 |
| SNORD19B | TNFRSF18 | -0.3376 | 6.26E-12 |
| SNORD19B | FOXD3 | -0.3376 | 6.27E-12 |
| SNORD19B | KIAA0664 | 0.3374 | 6.43E-12 |
| SNORD19B | SS18L2 | 0.3373 | 6.48E-12 |
| SNORD19B | HPSE2 | -0.3371 | 6.69E-12 |
| SNORD19B | CCDC147 | -0.3371 | 6.74E-12 |
| SNORD19B | BTBD16 | 0.3369 | 6.93E-12 |
| SNORD19B | TNFRSF21 | 0.3369 | 6.94E-12 |
| SNORD19B | ZMYND15 | -0.3368 | 7.00E-12 |
| SNORD19B | FOXQ1 | 0.3368 | 7.06E-12 |
| SNORD19B | SH3RF3 | -0.3368 | 7.06E-12 |
| SNORD19B | IL7R | -0.3367 | 7.11E-12 |
| SNORD19B | LEPRE1 | -0.3367 | 7.16E-12 |
| SNORD19B | PICK1 | 0.3366 | 7.20E-12 |
| SNORD19B | PTPRD | -0.3366 | 7.20E-12 |
| SNORD19B | PCOLCE2 | -0.3366 | 7.27E-12 |
| SNORD19B | GATA5 | -0.3365 | 7.32E-12 |
| SNORD19B | CYP1A1 | 0.3364 | 7.50E-12 |
| SNORD19B | TRAF3IP3 | -0.3363 | 7.56E-12 |
| SNORD19B | FLJ42709 | -0.3363 | 7.57E-12 |
| SNORD19B | IL1RAP | -0.3362 | 7.74E-12 |
| SNORD19B | AKAP13 | -0.3362 | 7.75E-12 |
| SNORD19B | DENND5A | -0.3361 | 7.88E-12 |
| SNORD19B | PHGR1 | 0.336 | 7.88E-12 |
| SNORD19B | ARHGEF10L | 0.336 | 7.89E-12 |
| SNORD19B | CDV3 | -0.336 | 7.93E-12 |
| SNORD19B | ACSF2 | 0.3358 | 8.15E-12 |
| SNORD19B | ARHGEF10 | -0.3358 | 8.19E-12 |
| SNORD19B | IGF1 | -0.3357 | 8.30E-12 |
| SNORD19B | ITGAX | -0.3356 | 8.39E-12 |
| SNORD19B | ACOX2 | -0.3356 | 8.43E-12 |
| SNORD19B | C1QB | -0.3356 | 8.48E-12 |
| SNORD19B | GLT25D1 | -0.3355 | 8.56E-12 |
| SNORD19B | NDUFA7 | 0.3355 | 8.60E-12 |
| SNORD19B | LOC100134713 | 0.3354 | 8.66E-12 |
| SNORD19B | PCDH9 | -0.3354 | 8.70E-12 |
| SNORD19B | BTBD19 | -0.3352 | 8.93E-12 |
| SNORD19B | SIGLEC1 | -0.3352 | 8.95E-12 |
| SNORD19B | AP1G2 | 0.3351 | 9.10E-12 |
| SNORD19B | TEX264 | 0.3351 | 9.10E-12 |
| SNORD19B | RALB | -0.3351 | 9.11E-12 |
| SNORD19B | F13A1 | -0.3351 | 9.13E-12 |
| SNORD19B | MGST2 | 0.3349 | 9.42E-12 |
| SNORD19B | SPSB1 | -0.3349 | 9.43E-12 |
| SNORD19B | SLC6A17 | -0.3348 | 9.46E-12 |
| SNORD19B | PIK3AP1 | -0.3348 | 9.48E-12 |
| SNORD19B | ITGA11 | -0.3348 | 9.52E-12 |
| SNORD19B | NGFR | -0.3348 | 9.52E-12 |
| SNORD19B | WDFY4 | -0.3347 | 9.62E-12 |
| SNORD19B | CYTH3 | -0.3347 | 9.67E-12 |
| SNORD19B | IKZF2 | 0.3347 | 9.73E-12 |
| SNORD19B | NOTCH2 | -0.3346 | 9.84E-12 |
| SNORD19B | EFCAB4A | 0.3346 | 9.86E-12 |
| SNORD19B | MMP9 | -0.3346 | 9.87E-12 |
| SNORD19B | FGD6 | -0.3345 | 9.94E-12 |
| SNORD19B | CCL4 | -0.3345 | 1.00E-11 |
| SNORD19B | FSTL1 | -0.3342 | 1.03E-11 |
| SNORD19B | THBS1 | -0.3343 | 1.03E-11 |
| SNORD19B | MMP2 | -0.3342 | 1.04E-11 |
| SNORD19B | C5orf62 | -0.3341 | 1.06E-11 |
| SNORD19B | ARHGAP31 | -0.334 | 1.07E-11 |
| SNORD19B | NPC1 | -0.3339 | 1.08E-11 |
| SNORD19B | PTPRJ | -0.334 | 1.08E-11 |
| SNORD19B | DUS1L | 0.3339 | 1.09E-11 |
| SNORD19B | EVI2B | -0.3338 | 1.10E-11 |
| SNORD19B | TRIM3 | -0.3339 | 1.10E-11 |
| SNORD19B | ADAMTSL3 | -0.3338 | 1.11E-11 |
| SNORD19B | STAT4 | -0.3338 | 1.11E-11 |
| SNORD19B | EHBP1L1 | -0.3337 | 1.13E-11 |
| SNORD19B | HECTD2 | -0.3333 | 1.19E-11 |
| SNORD19B | 6-Sep | -0.3333 | 1.19E-11 |
| SNORD19B | MAP7D3 | -0.3332 | 1.22E-11 |
| SNORD19B | ACER2 | 0.333 | 1.24E-11 |
| SNORD19B | CHMP4C | 0.333 | 1.24E-11 |
| SNORD19B | AADAT | 0.3329 | 1.27E-11 |
| SNORD19B | MAP4K4 | -0.3328 | 1.28E-11 |
| SNORD19B | LOC100216001 | -0.3328 | 1.29E-11 |
| SNORD19B | PPFIBP1 | -0.3327 | 1.30E-11 |
| SNORD19B | TUSC2 | 0.3327 | 1.30E-11 |
| SNORD19B | C1orf213 | 0.3326 | 1.31E-11 |
| SNORD19B | RHOH | -0.3327 | 1.31E-11 |
| SNORD19B | AOAH | -0.3326 | 1.32E-11 |
| SNORD19B | RIN3 | -0.3326 | 1.33E-11 |
| SNORD19B | SPSB4 | -0.3326 | 1.33E-11 |
| SNORD19B | VNN1 | -0.3325 | 1.34E-11 |
| SNORD19B | RAB25 | 0.3324 | 1.36E-11 |
| SNORD19B | ADORA1 | -0.3324 | 1.37E-11 |
| SNORD19B | CFB | -0.3324 | 1.37E-11 |
| SNORD19B | CSDAP1 | -0.3324 | 1.37E-11 |
| SNORD19B | HSPB6 | -0.3323 | 1.38E-11 |
| SNORD19B | APBB2 | -0.3322 | 1.40E-11 |
| SNORD19B | GTF3C2 | 0.3321 | 1.42E-11 |
| SNORD19B | WWC3 | -0.332 | 1.45E-11 |
| SNORD19B | IGF2BP2 | -0.3319 | 1.46E-11 |
| SNORD19B | KLF12 | -0.3318 | 1.48E-11 |
| SNORD19B | TMEM184A | 0.3317 | 1.51E-11 |
| SNORD19B | NDE1 | -0.3316 | 1.53E-11 |
| SNORD19B | NEIL1 | 0.3316 | 1.53E-11 |
| SNORD19B | POF1B | 0.3316 | 1.53E-11 |
| SNORD19B | PDZD3 | 0.3314 | 1.57E-11 |
| SNORD19B | TNNC1 | 0.3314 | 1.58E-11 |
| SNORD19B | FSTL3 | -0.3313 | 1.61E-11 |
| SNORD19B | GJC2 | -0.3311 | 1.65E-11 |
| SNORD19B | PLOD2 | -0.3311 | 1.66E-11 |
| SNORD19B | COPS8 | -0.3309 | 1.70E-11 |
| SNORD19B | LOC100144604 | 0.3309 | 1.71E-11 |
| SNORD19B | DAPK1 | 0.3308 | 1.73E-11 |
| SNORD19B | PRELP | -0.3307 | 1.75E-11 |
| SNORD19B | ITGA1 | -0.3307 | 1.76E-11 |
| SNORD19B | TMEM90B | -0.3306 | 1.78E-11 |
| SNORD19B | SFRS13B | -0.3304 | 1.83E-11 |
| SNORD19B | AGPAT9 | -0.3304 | 1.84E-11 |
| SNORD19B | FAM3B | 0.3303 | 1.86E-11 |
| SNORD19B | RUNX1T1 | -0.3302 | 1.89E-11 |
| SNORD19B | FERMT3 | -0.3301 | 1.92E-11 |
| SNORD19B | OVOL1 | 0.33 | 1.93E-11 |
| SNORD19B | MCCC2 | 0.3298 | 2.00E-11 |
| SNORD19B | SIGLEC15 | 0.3298 | 2.00E-11 |
| SNORD19B | RBP1 | -0.3298 | 2.01E-11 |
| SNORD19B | SNCG | 0.3297 | 2.02E-11 |
| SNORD19B | OR13A1 | 0.3297 | 2.05E-11 |
| SNORD19B | SASH3 | -0.3296 | 2.07E-11 |
| SNORD19B | ACTR8 | 0.3295 | 2.09E-11 |
| SNORD19B | GNAL | -0.3294 | 2.12E-11 |
| SNORD19B | NPAS4 | -0.3294 | 2.13E-11 |
| SNORD19B | NT5E | -0.3293 | 2.14E-11 |
| SNORD19B | PRKCD | 0.3294 | 2.14E-11 |
| SNORD19B | RAP1GAP | 0.3294 | 2.14E-11 |
| SNORD19B | SLC15A3 | -0.3294 | 2.14E-11 |
| SNORD19B | CAB39L | 0.3293 | 2.16E-11 |
| SNORD19B | MEFV | -0.3293 | 2.16E-11 |
| SNORD19B | ACTR3B | 0.3291 | 2.21E-11 |
| SNORD19B | MCTP1 | -0.3291 | 2.22E-11 |
| SNORD19B | HSPG2 | -0.3291 | 2.23E-11 |
| SNORD19B | KLRD1 | -0.329 | 2.25E-11 |
| SNORD19B | COL11A1 | -0.329 | 2.26E-11 |
| SNORD19B | KRT12 | 0.3289 | 2.28E-11 |
| SNORD19B | TJP3 | 0.3289 | 2.28E-11 |
| SNORD19B | RAB32 | -0.3288 | 2.31E-11 |
| SNORD19B | ACP6 | 0.3287 | 2.34E-11 |
| SNORD19B | SNTG1 | 0.3287 | 2.36E-11 |
| SNORD19B | CCDC8 | -0.3285 | 2.42E-11 |
| SNORD19B | CREB3L1 | -0.3285 | 2.43E-11 |
| SNORD19B | MAGI1 | 0.3285 | 2.44E-11 |
| SNORD19B | ST3GAL4 | 0.3283 | 2.50E-11 |
| SNORD19B | NCRNA00173 | -0.3282 | 2.52E-11 |
| SNORD19B | SLC7A7 | -0.3282 | 2.54E-11 |
| SNORD19B | PCSK9 | -0.3282 | 2.55E-11 |
| SNORD19B | FAM118A | 0.3281 | 2.57E-11 |
| SNORD19B | ARL2BP | -0.3281 | 2.58E-11 |
| SNORD19B | HMGA2 | -0.3281 | 2.58E-11 |
| SNORD19B | SLC36A2 | 0.328 | 2.60E-11 |
| SNORD19B | KRT20 | 0.328 | 2.62E-11 |
| SNORD19B | FBXO31 | -0.3279 | 2.64E-11 |
| SNORD19B | PDLIM2 | -0.3278 | 2.70E-11 |
| SNORD19B | ANK2 | -0.3277 | 2.72E-11 |
| SNORD19B | ELF3 | 0.3277 | 2.72E-11 |
| SNORD19B | ZNF253 | 0.3277 | 2.73E-11 |
| SNORD19B | CAPS | 0.3276 | 2.75E-11 |
| SNORD19B | LCP2 | -0.3275 | 2.79E-11 |
| SNORD19B | PLEKHJ1 | 0.3275 | 2.80E-11 |
| SNORD19B | ZSCAN16 | 0.3275 | 2.81E-11 |
| SNORD19B | FOXA1 | 0.3273 | 2.88E-11 |
| SNORD19B | SLC24A3 | -0.3273 | 2.91E-11 |
| SNORD19B | STON1 | -0.3272 | 2.93E-11 |
| SNORD19B | PHYHIP | -0.3272 | 2.94E-11 |
| SNORD19B | CFLAR | -0.3272 | 2.95E-11 |
| SNORD19B | RGS18 | -0.3271 | 2.97E-11 |
| SNORD19B | SIGLEC10 | -0.3271 | 2.97E-11 |
| SNORD19B | SLC26A10 | -0.327 | 3.01E-11 |
| SNORD19B | VSIG10 | 0.327 | 3.01E-11 |
| SNORD19B | HK3 | -0.327 | 3.02E-11 |
| SNORD19B | IL21R | -0.327 | 3.03E-11 |
| SNORD19B | GULP1 | -0.3269 | 3.07E-11 |
| SNORD19B | ROR1 | -0.3269 | 3.07E-11 |
| SNORD19B | LEAP2 | 0.3268 | 3.10E-11 |
| SNORD19B | F3 | -0.3268 | 3.11E-11 |
| SNORD19B | GPR160 | 0.3268 | 3.11E-11 |
| SNORD19B | SOD3 | -0.3266 | 3.19E-11 |
| SNORD19B | ZBTB7C | 0.3266 | 3.19E-11 |
| SNORD19B | FAT4 | -0.3266 | 3.20E-11 |
| SNORD19B | FCGR2C | -0.3265 | 3.25E-11 |
| SNORD19B | RIMBP2 | -0.3263 | 3.33E-11 |
| SNORD19B | DHX30 | 0.3263 | 3.36E-11 |
| SNORD19B | OPTN | -0.3263 | 3.36E-11 |
| SNORD19B | WIT1 | -0.3262 | 3.38E-11 |
| SNORD19B | MRC1 | -0.3261 | 3.44E-11 |
| SNORD19B | P4HA2 | -0.326 | 3.50E-11 |
| SNORD19B | MMGT1 | -0.3259 | 3.53E-11 |
| SNORD19B | PCDHGA12 | -0.3259 | 3.53E-11 |
| SNORD19B | TTYH2 | -0.3259 | 3.53E-11 |
| SNORD19B | JAK1 | -0.3258 | 3.62E-11 |
| SNORD19B | GPM6A | -0.3257 | 3.64E-11 |
| SNORD19B | ZNF321 | 0.3257 | 3.64E-11 |
| SNORD19B | TPST1 | -0.3257 | 3.65E-11 |
| SNORD19B | TSTD1 | 0.3256 | 3.69E-11 |
| SNORD19B | MS4A4A | -0.3256 | 3.70E-11 |
| SNORD19B | BMPER | -0.3255 | 3.76E-11 |
| SNORD19B | IL12RB2 | -0.3255 | 3.76E-11 |
| SNORD19B | HAS1 | -0.3255 | 3.77E-11 |
| SNORD19B | C17orf87 | -0.3254 | 3.81E-11 |
| SNORD19B | CALB2 | -0.3254 | 3.81E-11 |
| SNORD19B | CERKL | -0.3254 | 3.81E-11 |
| SNORD19B | SLA | -0.3253 | 3.85E-11 |
| SNORD19B | TPBG | -0.3252 | 3.91E-11 |
| SNORD19B | SHROOM1 | 0.3252 | 3.96E-11 |
| SNORD19B | TMEM163 | 0.3251 | 3.96E-11 |
| SNORD19B | TREM1 | -0.3252 | 3.96E-11 |
| SNORD19B | XIRP1 | -0.325 | 4.03E-11 |
| SNORD19B | DEGS2 | 0.325 | 4.04E-11 |
| SNORD19B | C7orf46 | 0.3249 | 4.08E-11 |
| SNORD19B | RCN3 | -0.3249 | 4.12E-11 |
| SNORD19B | PDE4B | -0.3247 | 4.20E-11 |
| SNORD19B | LILRB1 | -0.3247 | 4.22E-11 |
| SNORD19B | CDON | -0.3247 | 4.24E-11 |
| SNORD19B | IL31RA | -0.3247 | 4.25E-11 |
| SNORD19B | GNGT2 | -0.3246 | 4.26E-11 |
| SNORD19B | GPR124 | -0.3246 | 4.31E-11 |
| SNORD19B | CNRIP1 | -0.3245 | 4.32E-11 |
| SNORD19B | S100B | -0.3246 | 4.32E-11 |
| SNORD19B | FCGR2B | -0.3245 | 4.35E-11 |
| SNORD19B | ZNF737 | 0.3245 | 4.35E-11 |
| SNORD19B | SLCO2B1 | -0.3243 | 4.45E-11 |
| SNORD19B | WNT2B | -0.3243 | 4.46E-11 |
| SNORD19B | SMARCC1 | 0.3243 | 4.51E-11 |
| SNORD19B | ST6GALNAC6 | -0.3242 | 4.57E-11 |
| SNORD19B | IL10RA | -0.324 | 4.70E-11 |
| SNORD19B | TBC1D3B | 0.3239 | 4.72E-11 |
| SNORD19B | C3orf62 | 0.3238 | 4.81E-11 |
| SNORD19B | SORBS1 | -0.3238 | 4.82E-11 |
| SNORD19B | C15orf59 | -0.3238 | 4.83E-11 |
| SNORD19B | APEH | 0.3236 | 4.92E-11 |
| SNORD19B | MAP3K6 | -0.3236 | 4.92E-11 |
| SNORD19B | CD70 | -0.3235 | 4.99E-11 |
| SNORD19B | DPP4 | -0.3235 | 5.02E-11 |
| SNORD19B | ACSL5 | 0.3234 | 5.07E-11 |
| SNORD19B | NEURL1B | -0.3234 | 5.08E-11 |
| SNORD19B | NID1 | -0.3231 | 5.33E-11 |
| SNORD19B | UQCR10 | 0.3231 | 5.36E-11 |
| SNORD19B | MT1M | -0.323 | 5.44E-11 |
| SNORD19B | SOCS3 | -0.3229 | 5.44E-11 |
| SNORD19B | HCST | -0.3229 | 5.51E-11 |
| SNORD19B | MYO5B | 0.3228 | 5.57E-11 |
| SNORD19B | RYR2 | -0.3228 | 5.59E-11 |
| SNORD19B | CCL2 | -0.3227 | 5.60E-11 |
| SNORD19B | HRH2 | -0.3227 | 5.62E-11 |
| SNORD19B | GPR141 | -0.3227 | 5.66E-11 |
| SNORD19B | POGK | 0.3227 | 5.67E-11 |
| SNORD19B | GAP43 | -0.3226 | 5.75E-11 |
| SNORD19B | TMC4 | 0.3225 | 5.84E-11 |
| SNORD19B | TWIST1 | -0.3224 | 5.87E-11 |
| SNORD19B | GUCY1A3 | -0.3223 | 5.94E-11 |
| SNORD19B | LILRB4 | -0.3223 | 5.96E-11 |
| SNORD19B | C10orf72 | -0.3223 | 5.97E-11 |
| SNORD19B | CD300LB | -0.3222 | 6.04E-11 |
| SNORD19B | RBM7 | -0.322 | 6.19E-11 |
| SNORD19B | ARHGAP22 | -0.322 | 6.20E-11 |
| SNORD19B | ZFP62 | 0.322 | 6.24E-11 |
| SNORD19B | WDR52 | 0.3219 | 6.29E-11 |
| SNORD19B | LYPLA1 | 0.3219 | 6.34E-11 |
| SNORD19B | ITPR2 | -0.3219 | 6.36E-11 |
| SNORD19B | WBP1 | 0.3219 | 6.37E-11 |
| SNORD19B | NFATC1 | -0.3218 | 6.45E-11 |
| SNORD19B | LUM | -0.3216 | 6.64E-11 |
| SNORD19B | OLFML2B | -0.3215 | 6.72E-11 |
| SNORD19B | TRIM24 | 0.3214 | 6.79E-11 |
| SNORD19B | SPTA1 | -0.3214 | 6.84E-11 |
| SNORD19B | TMEM200A | -0.3213 | 6.93E-11 |
| SNORD19B | P2RY13 | -0.3212 | 7.01E-11 |
| SNORD19B | BCAS1 | 0.3212 | 7.03E-11 |
| SNORD19B | CPVL | -0.3211 | 7.08E-11 |
| SNORD19B | TDH | 0.321 | 7.15E-11 |
| SNORD19B | CALU | -0.321 | 7.19E-11 |
| SNORD19B | FAM124A | -0.3209 | 7.25E-11 |
| SNORD19B | CD80 | -0.3209 | 7.26E-11 |
| SNORD19B | LOC283731 | -0.3209 | 7.34E-11 |
| SNORD19B | SMARCE1 | 0.3207 | 7.46E-11 |
| SNORD19B | ARHGEF37 | -0.3207 | 7.54E-11 |
| SNORD19B | AFAP1 | -0.3206 | 7.62E-11 |
| SNORD19B | IGSF6 | -0.3206 | 7.62E-11 |
| SNORD19B | SLC9A4 | 0.3206 | 7.67E-11 |
| SNORD19B | IRF8 | -0.3204 | 7.82E-11 |
| SNORD19B | OXCT1 | -0.3204 | 7.86E-11 |
| SNORD19B | TMEM150B | -0.3203 | 7.91E-11 |
| SNORD19B | CD47 | -0.3202 | 8.11E-11 |
| SNORD19B | KRT6B | -0.3202 | 8.12E-11 |
| SNORD19B | BLVRA | -0.3201 | 8.13E-11 |
| SNORD19B | IDO1 | -0.3201 | 8.19E-11 |
| SNORD19B | RNF175 | -0.32 | 8.25E-11 |
| SNORD19B | FGF2 | -0.32 | 8.26E-11 |
| SNORD19B | SAMD12 | 0.32 | 8.26E-11 |
| SNORD19B | S100P | 0.3199 | 8.42E-11 |
| SNORD19B | CCL3 | -0.3197 | 8.61E-11 |
| SNORD19B | EYA1 | -0.3197 | 8.67E-11 |
| SNORD19B | TCHH | -0.3197 | 8.70E-11 |
| SNORD19B | C17orf37 | 0.3196 | 8.85E-11 |
| SNORD19B | TPD52L1 | -0.3195 | 8.87E-11 |
| SNORD19B | CCL7 | -0.3194 | 9.08E-11 |
| SNORD19B | TMEM176B | -0.3194 | 9.09E-11 |
| SNORD19B | C1orf54 | -0.3193 | 9.18E-11 |
| SNORD19B | NKAPL | -0.3192 | 9.25E-11 |
| SNORD19B | CSMD2 | -0.3192 | 9.30E-11 |
| SNORD19B | PKD2 | -0.3191 | 9.47E-11 |
| SNORD19B | TRHDE | 0.3191 | 9.49E-11 |
| SNORD19B | RNF145 | -0.319 | 9.54E-11 |
| SNORD19B | LAMP2 | -0.319 | 9.56E-11 |
| SNORD19B | BCHE | -0.3189 | 9.76E-11 |
| SNORD19B | PARVG | -0.3188 | 9.84E-11 |
| SNORD19B | ME1 | -0.3188 | 9.90E-11 |
| SNORD19B | LOC149134 | 0.3187 | 9.98E-11 |
| SNORD19B | SLC19A1 | 0.3187 | 1.00E-10 |
| SNORD19B | PLAU | -0.3186 | 1.01E-10 |
| SNORD19B | PGPEP1 | 0.3185 | 1.02E-10 |
| SNORD19B | MRAS | -0.3185 | 1.03E-10 |
| SNORD19B | PMS2L3 | 0.3185 | 1.03E-10 |
| SNORD19B | BICD1 | -0.3184 | 1.04E-10 |
| SNORD19B | C8orf47 | 0.3184 | 1.04E-10 |
| SNORD19B | CD180 | -0.3184 | 1.04E-10 |
| SNORD19B | FAIM2 | -0.3184 | 1.04E-10 |
| SNORD19B | MRVI1 | -0.3184 | 1.04E-10 |
| SNORD19B | PAPLN | -0.3184 | 1.04E-10 |
| SNORD19B | SERPINB9 | -0.3184 | 1.04E-10 |
| SNORD19B | SLC18A2 | -0.3184 | 1.04E-10 |
| SNORD19B | GDF5 | -0.3183 | 1.05E-10 |
| SNORD19B | PABPC4L | -0.3183 | 1.05E-10 |
| SNORD19B | TSGA10 | 0.3182 | 1.07E-10 |
| SNORD19B | TTLL11 | -0.3182 | 1.07E-10 |
| SNORD19B | ZNF44 | 0.318 | 1.10E-10 |
| SNORD19B | GDF7 | 0.318 | 1.11E-10 |
| SNORD19B | SPRED2 | 0.3179 | 1.11E-10 |
| SNORD19B | KIAA1045 | -0.3178 | 1.13E-10 |
| SNORD19B | PTPLA | -0.3178 | 1.13E-10 |
| SNORD19B | KIF5C | 0.3177 | 1.15E-10 |
| SNORD19B | HPGDS | -0.3176 | 1.16E-10 |
| SNORD19B | NSUN5P2 | 0.3176 | 1.16E-10 |
| SNORD19B | CCNG2 | 0.3176 | 1.17E-10 |
| SNORD19B | STAC | -0.3176 | 1.17E-10 |
| SNORD19B | POLR1C | 0.3175 | 1.18E-10 |
| SNORD19B | MRPS25 | 0.3174 | 1.19E-10 |
| SNORD19B | PATZ1 | 0.3174 | 1.19E-10 |
| SNORD19B | ABL2 | -0.3174 | 1.20E-10 |
| SNORD19B | WAS | -0.3174 | 1.20E-10 |
| SNORD19B | EVC | -0.3174 | 1.21E-10 |
| SNORD19B | PLEKHO1 | -0.3173 | 1.22E-10 |
| SNORD19B | TTC7A | -0.3173 | 1.22E-10 |
| SNORD19B | PTPRC | -0.3172 | 1.23E-10 |
| SNORD19B | CD38 | -0.3172 | 1.24E-10 |
| SNORD19B | RFPL1S | -0.3172 | 1.24E-10 |
| SNORD19B | CD37 | -0.3171 | 1.25E-10 |
| SNORD19B | RAET1L | -0.317 | 1.27E-10 |
| SNORD19B | SELM | -0.317 | 1.27E-10 |
| SNORD19B | CYR61 | -0.3169 | 1.29E-10 |
| SNORD19B | C9orf116 | 0.3168 | 1.30E-10 |
| SNORD19B | CD2AP | 0.3168 | 1.30E-10 |
| SNORD19B | CES4 | -0.3168 | 1.30E-10 |
| SNORD19B | MIAT | -0.3167 | 1.32E-10 |
| SNORD19B | SLC2A3 | -0.3166 | 1.34E-10 |
| SNORD19B | STAB1 | -0.3166 | 1.34E-10 |
| SNORD19B | GPR183 | -0.3165 | 1.35E-10 |
| SNORD19B | PLCL1 | -0.3166 | 1.35E-10 |
| SNORD19B | C13orf33 | -0.3164 | 1.38E-10 |
| SNORD19B | CD300LF | -0.3163 | 1.40E-10 |
| SNORD19B | LRRC8A | -0.3163 | 1.40E-10 |
| SNORD19B | PRSS35 | -0.3163 | 1.40E-10 |
| SNORD19B | AMPH | -0.3162 | 1.41E-10 |
| SNORD19B | LOC283392 | 0.3162 | 1.42E-10 |
| SNORD19B | SEMA3F | 0.316 | 1.45E-10 |
| SNORD19B | CCDC102A | -0.3159 | 1.48E-10 |
| SNORD19B | LOC80054 | 0.3159 | 1.48E-10 |
| SNORD19B | KIF9 | 0.3158 | 1.51E-10 |
| SNORD19B | CRISPLD2 | -0.3156 | 1.54E-10 |
| SNORD19B | IL15RA | -0.3156 | 1.54E-10 |
| SNORD19B | DMWD | -0.3156 | 1.55E-10 |
| SNORD19B | THY1 | -0.3155 | 1.57E-10 |
| SNORD19B | FBLN2 | -0.3154 | 1.58E-10 |
| SNORD19B | AOC2 | 0.3154 | 1.59E-10 |
| SNORD19B | EPB41L2 | -0.3154 | 1.59E-10 |
| SNORD19B | PRR5 | 0.3153 | 1.60E-10 |
| SNORD19B | SCD5 | -0.3153 | 1.61E-10 |
| SNORD19B | GRM3 | 0.3152 | 1.62E-10 |
| SNORD19B | MYO1F | -0.3152 | 1.63E-10 |
| SNORD19B | MKRN1 | 0.3151 | 1.66E-10 |
| SNORD19B | TPRN | 0.315 | 1.67E-10 |
| SNORD19B | GPR65 | -0.3148 | 1.73E-10 |
| SNORD19B | LOC646851 | 0.3147 | 1.74E-10 |
| SNORD19B | SCG2 | -0.3148 | 1.74E-10 |
| SNORD19B | PALM2 | -0.3147 | 1.76E-10 |
| SNORD19B | PTPN7 | -0.3146 | 1.77E-10 |
| SNORD19B | TOP2B | 0.3146 | 1.77E-10 |
| SNORD19B | BIN2 | -0.3146 | 1.78E-10 |
| SNORD19B | LCP1 | -0.3145 | 1.79E-10 |
| SNORD19B | PGA3 | -0.3145 | 1.80E-10 |
| SNORD19B | RHOC | -0.3145 | 1.81E-10 |
| SNORD19B | PBX3 | -0.3143 | 1.84E-10 |
| SNORD19B | ANKRD46 | 0.3143 | 1.85E-10 |
| SNORD19B | KPNA3 | -0.3143 | 1.85E-10 |
| SNORD19B | SLC6A9 | -0.3143 | 1.86E-10 |
| SNORD19B | DENND2D | 0.3142 | 1.89E-10 |
| SNORD19B | LRRC32 | -0.3142 | 1.89E-10 |
| SNORD19B | ANKRD44 | -0.314 | 1.93E-10 |
| SNORD19B | RSPO2 | -0.314 | 1.93E-10 |
| SNORD19B | LAMA3 | -0.3139 | 1.95E-10 |
| SNORD19B | RNF130 | -0.3139 | 1.97E-10 |
| SNORD19B | UBE2E2 | -0.3139 | 1.97E-10 |
| SNORD19B | AMIGO3 | 0.3138 | 1.98E-10 |
| SNORD19B | CDX1 | -0.3138 | 1.98E-10 |
| SNORD19B | IL16 | -0.3138 | 1.98E-10 |
| SNORD19B | MPP3 | -0.3138 | 1.99E-10 |
| SNORD19B | ADORA3 | -0.3137 | 2.02E-10 |
| SNORD19B | ATP6V1B2 | -0.3136 | 2.04E-10 |
| SNORD19B | HLA | -0.3136 | 2.04E-10 |
| SNORD19B | MAPK11 | -0.3136 | 2.04E-10 |
| SNORD19B | HNF1B | 0.3135 | 2.06E-10 |
| SNORD19B | SIRPB2 | -0.3135 | 2.08E-10 |
| SNORD19B | SLC22A16 | -0.3134 | 2.09E-10 |
| SNORD19B | MCFD2 | -0.3134 | 2.10E-10 |
| SNORD19B | SV2B | -0.3133 | 2.12E-10 |
| SNORD19B | ZNF513 | 0.3133 | 2.13E-10 |
| SNORD19B | PLA2G6 | 0.3133 | 2.14E-10 |
| SNORD19B | CHI3L1 | -0.3132 | 2.17E-10 |
| SNORD19B | C6orf150 | -0.313 | 2.23E-10 |
| SNORD19B | MMP23B | -0.3129 | 2.24E-10 |
| SNORD19B | PPP1R9A | 0.3129 | 2.24E-10 |
| SNORD19B | C1orf159 | 0.3129 | 2.26E-10 |
| SNORD19B | GPM6B | -0.3129 | 2.26E-10 |
| SNORD19B | MYO1B | -0.3129 | 2.26E-10 |
| SNORD19B | DCAKD | 0.3128 | 2.27E-10 |
| SNORD19B | LLGL2 | 0.3128 | 2.29E-10 |
| SNORD19B | SGCA | -0.3127 | 2.31E-10 |
| SNORD19B | EMR1 | -0.3126 | 2.36E-10 |
| SNORD19B | C6orf59 | -0.3125 | 2.37E-10 |
| SNORD19B | MPST | 0.3125 | 2.37E-10 |
| SNORD19B | KLHDC5 | -0.3125 | 2.38E-10 |
| SNORD19B | GLT1D1 | -0.3125 | 2.39E-10 |
| SNORD19B | RHPN1 | 0.3125 | 2.39E-10 |
| SNORD19B | LOXL3 | -0.3124 | 2.43E-10 |
| SNORD19B | PRRX2 | -0.3122 | 2.47E-10 |
| SNORD19B | RBMS2 | -0.3122 | 2.47E-10 |
| SNORD19B | TBX4 | -0.3122 | 2.48E-10 |
| SNORD19B | RTN1 | -0.3121 | 2.52E-10 |
| SNORD19B | CDA | -0.3121 | 2.53E-10 |
| SNORD19B | RNF208 | 0.312 | 2.54E-10 |
| SNORD19B | CXCL9 | -0.3119 | 2.58E-10 |
| SNORD19B | SLFN12L | -0.3118 | 2.62E-10 |
| SNORD19B | MPND | 0.3117 | 2.66E-10 |
| SNORD19B | NCS1 | -0.3116 | 2.69E-10 |
| SNORD19B | RHOU | 0.3116 | 2.70E-10 |
| SNORD19B | ZNF763 | 0.3114 | 2.78E-10 |
| SNORD19B | SNAI2 | -0.3114 | 2.79E-10 |
| SNORD19B | PENK | -0.3113 | 2.81E-10 |
| SNORD19B | PLK2 | -0.3112 | 2.83E-10 |
| SNORD19B | CHST7 | -0.3112 | 2.85E-10 |
| SNORD19B | HRH3 | 0.3111 | 2.89E-10 |
| SNORD19B | C1orf172 | 0.311 | 2.94E-10 |
| SNORD19B | SSFA2 | -0.311 | 2.94E-10 |
| SNORD19B | SIRPB1 | -0.3109 | 2.95E-10 |
| SNORD19B | CST7 | -0.3109 | 2.96E-10 |
| SNORD19B | RAB11A | 0.3109 | 2.96E-10 |
| SNORD19B | C2orf40 | -0.3109 | 2.97E-10 |
| SNORD19B | CARD11 | 0.3109 | 2.98E-10 |
| SNORD19B | PNMAL1 | -0.3108 | 3.00E-10 |
| SNORD19B | SAMHD1 | -0.3108 | 3.01E-10 |
| SNORD19B | AGTR1 | -0.3107 | 3.03E-10 |
| SNORD19B | IL2RA | -0.3107 | 3.06E-10 |
| SNORD19B | KANK2 | -0.3107 | 3.06E-10 |
| SNORD19B | TBX2 | 0.3107 | 3.06E-10 |
| SNORD19B | C17orf60 | -0.3106 | 3.09E-10 |
| SNORD19B | CD24 | 0.3106 | 3.09E-10 |
| SNORD19B | RNF115 | -0.3105 | 3.13E-10 |
| SNORD19B | ACAP1 | -0.3104 | 3.18E-10 |
| SNORD19B | NSUN5P1 | 0.3103 | 3.23E-10 |
| SNORD19B | PLEKHM1P | 0.3103 | 3.23E-10 |
| SNORD19B | RECQL | -0.3101 | 3.29E-10 |
| SNORD19B | TREM2 | -0.3102 | 3.29E-10 |
| SNORD19B | PCOLCE | -0.3101 | 3.30E-10 |
| SNORD19B | BMP2K | -0.31 | 3.34E-10 |
| SNORD19B | TM4SF1 | -0.31 | 3.34E-10 |
| SNORD19B | TAGAP | -0.31 | 3.36E-10 |
| SNORD19B | GDPD3 | 0.3098 | 3.44E-10 |
| SNORD19B | LOC646999 | -0.3097 | 3.47E-10 |
| SNORD19B | DNAJC24 | -0.3097 | 3.50E-10 |
| SNORD19B | RARRES2 | -0.3097 | 3.51E-10 |
| SNORD19B | IKZF1 | -0.3096 | 3.53E-10 |
| SNORD19B | SORCS2 | -0.3096 | 3.53E-10 |
| SNORD19B | PLEKHA6 | 0.3096 | 3.54E-10 |
| SNORD19B | GCAT | 0.3095 | 3.59E-10 |
| SNORD19B | ATP2B4 | -0.3095 | 3.61E-10 |
| SNORD19B | TRAF3 | -0.3093 | 3.68E-10 |
| SNORD19B | COMP | -0.3093 | 3.69E-10 |
| SNORD19B | OAT | -0.3091 | 3.79E-10 |
| SNORD19B | ZDHHC3 | 0.3091 | 3.79E-10 |
| SNORD19B | NT5C2 | 0.3091 | 3.80E-10 |
| SNORD19B | TBXAS1 | -0.3091 | 3.81E-10 |
| SNORD19B | LOC152225 | -0.3091 | 3.82E-10 |
| SNORD19B | CPNE8 | -0.3089 | 3.91E-10 |
| SNORD19B | RBPMS2 | -0.3087 | 4.04E-10 |
| SNORD19B | S100A5 | 0.3086 | 4.08E-10 |
| SNORD19B | MMP14 | -0.3085 | 4.10E-10 |
| SNORD19B | IKBIP | -0.3085 | 4.12E-10 |
| SNORD19B | STK17A | -0.3085 | 4.13E-10 |
| SNORD19B | PLA2G12A | 0.3085 | 4.14E-10 |
| SNORD19B | ZPLD1 | -0.3085 | 4.14E-10 |
| SNORD19B | SEMA6A | 0.3084 | 4.15E-10 |
| SNORD19B | BNC1 | -0.3083 | 4.22E-10 |
| SNORD19B | UPK3A | 0.3082 | 4.28E-10 |
| SNORD19B | LOXL2 | -0.3082 | 4.30E-10 |
| SNORD19B | NID2 | -0.3082 | 4.30E-10 |
| SNORD19B | THBS4 | -0.3082 | 4.30E-10 |
| SNORD19B | SLC23A3 | 0.3081 | 4.33E-10 |
| SNORD19B | SLC2A14 | -0.3081 | 4.33E-10 |
| SNORD19B | UNC5C | -0.3081 | 4.36E-10 |
| SNORD19B | POPDC2 | -0.308 | 4.39E-10 |
| SNORD19B | LHFP | -0.3078 | 4.50E-10 |
| SNORD19B | NT5DC2 | 0.3078 | 4.56E-10 |
| SNORD19B | IL17B | -0.3077 | 4.57E-10 |
| SNORD19B | PTX3 | -0.3077 | 4.57E-10 |
| SNORD19B | LGI2 | -0.3076 | 4.63E-10 |
| SNORD19B | C7orf55 | 0.3076 | 4.64E-10 |
| SNORD19B | TMEM176A | -0.3076 | 4.65E-10 |
| SNORD19B | DYSF | -0.3076 | 4.66E-10 |
| SNORD19B | ARHGAP8 | 0.3076 | 4.68E-10 |
| SNORD19B | TOB1 | 0.3075 | 4.69E-10 |
| SNORD19B | GNG4 | -0.3075 | 4.72E-10 |
| SNORD19B | PNMT | 0.3075 | 4.75E-10 |
| SNORD19B | PHLDB2 | -0.3074 | 4.81E-10 |
| SNORD19B | CCR5 | -0.3072 | 4.89E-10 |
| SNORD19B | CD226 | -0.3072 | 4.92E-10 |
| SNORD19B | FAM176A | -0.3072 | 4.93E-10 |
| SNORD19B | NFE2L1 | -0.3071 | 4.98E-10 |
| SNORD19B | FBLN5 | -0.307 | 5.05E-10 |
| SNORD19B | CCDC125 | 0.307 | 5.08E-10 |
| SNORD19B | PTGIS | -0.3069 | 5.12E-10 |
| SNORD19B | VMAC | 0.3069 | 5.15E-10 |
| SNORD19B | ABL1 | -0.3068 | 5.17E-10 |
| SNORD19B | CDO1 | -0.3068 | 5.17E-10 |
| SNORD19B | EHBP1 | -0.3068 | 5.20E-10 |
| SNORD19B | TSPAN2 | -0.3068 | 5.20E-10 |
| SNORD19B | STAP2 | 0.3068 | 5.21E-10 |
| SNORD19B | TXK | 0.3068 | 5.22E-10 |
| SNORD19B | JAM3 | -0.3067 | 5.24E-10 |
| SNORD19B | FKBP9L | -0.3066 | 5.30E-10 |
| SNORD19B | CD274 | -0.3066 | 5.32E-10 |
| SNORD19B | SMTN | -0.3066 | 5.34E-10 |
| SNORD19B | MOGS | 0.3065 | 5.38E-10 |
| SNORD19B | SLC5A7 | 0.3065 | 5.40E-10 |
| SNORD19B | PHLDB3 | 0.3064 | 5.46E-10 |
| SNORD19B | ZNF433 | 0.3064 | 5.46E-10 |
| SNORD19B | EPHA6 | -0.3064 | 5.47E-10 |
| SNORD19B | FAAH | 0.3064 | 5.47E-10 |
| SNORD19B | NARF | 0.3064 | 5.48E-10 |
| SNORD19B | FAM43A | -0.3064 | 5.49E-10 |
| SNORD19B | LOC440944 | 0.3064 | 5.49E-10 |
| SNORD19B | PTPRZ1 | -0.3064 | 5.50E-10 |
| SNORD19B | TMEM55A | -0.3064 | 5.51E-10 |
| SNORD19B | RALGPS1 | 0.3063 | 5.53E-10 |
| SNORD19B | PLXDC2 | -0.3063 | 5.58E-10 |
| SNORD19B | CCL15 | 0.3062 | 5.60E-10 |
| SNORD19B | EGR3 | -0.3062 | 5.60E-10 |
| SNORD19B | ATP10D | -0.3062 | 5.62E-10 |
| SNORD19B | NCF1B | -0.3061 | 5.69E-10 |
| SNORD19B | CXCL10 | -0.3061 | 5.75E-10 |
| SNORD19B | RNF186 | 0.306 | 5.78E-10 |
| SNORD19B | FBXO41 | 0.306 | 5.80E-10 |
| SNORD19B | SMPD2 | 0.306 | 5.80E-10 |
| SNORD19B | LITAF | -0.306 | 5.81E-10 |
| SNORD19B | IL10 | -0.3059 | 5.85E-10 |
| SNORD19B | TCEAL2 | -0.3057 | 6.03E-10 |
| SNORD19B | CLEC7A | -0.3057 | 6.04E-10 |
| SNORD19B | DCHS2 | -0.3057 | 6.04E-10 |
| SNORD19B | CMAH | -0.3056 | 6.10E-10 |
| SNORD19B | ITGA4 | -0.3055 | 6.20E-10 |
| SNORD19B | EPYC | -0.3055 | 6.21E-10 |
| SNORD19B | SH2D4A | 0.3054 | 6.26E-10 |
| SNORD19B | PTK2 | 0.3053 | 6.37E-10 |
| SNORD19B | ARID5A | -0.3053 | 6.38E-10 |
| SNORD19B | MGC12916 | -0.3052 | 6.49E-10 |
| SNORD19B | ARHGAP1 | -0.3051 | 6.51E-10 |
| SNORD19B | KRT7 | 0.3051 | 6.51E-10 |
| SNORD19B | MMD | -0.3051 | 6.55E-10 |
| SNORD19B | LILRA1 | -0.3051 | 6.57E-10 |
| SNORD19B | NCF1C | -0.305 | 6.66E-10 |
| SNORD19B | PCDHGA9 | -0.3049 | 6.67E-10 |
| SNORD19B | ANXA1 | -0.3049 | 6.69E-10 |
| SNORD19B | ABHD14B | 0.3049 | 6.71E-10 |
| SNORD19B | GGA1 | 0.3048 | 6.80E-10 |
| SNORD19B | CCDC24 | 0.3047 | 6.89E-10 |
| SNORD19B | SERPINF1 | -0.3047 | 6.90E-10 |
| SNORD19B | RASL12 | -0.3047 | 6.93E-10 |
| SNORD19B | ODF3B | -0.3046 | 6.97E-10 |
| SNORD19B | ADH6 | 0.3046 | 6.99E-10 |
| SNORD19B | DNAJA4 | 0.3046 | 6.99E-10 |
| SNORD19B | JMJD7 | 0.3046 | 7.00E-10 |
| SNORD19B | GRK5 | -0.3046 | 7.03E-10 |
| SNORD19B | MFSD9 | 0.3046 | 7.03E-10 |
| SNORD19B | NHEDC2 | -0.3045 | 7.10E-10 |
| SNORD19B | PSMB9 | -0.3045 | 7.10E-10 |
| SNORD19B | UNC5A | -0.3045 | 7.10E-10 |
| SNORD19B | KLF7 | -0.3044 | 7.20E-10 |
| SNORD19B | NRXN1 | -0.3043 | 7.24E-10 |
| SNORD19B | SMOC2 | -0.3043 | 7.25E-10 |
| SNORD19B | NFXL1 | 0.3043 | 7.26E-10 |
| SNORD19B | TLR2 | -0.3043 | 7.26E-10 |
| SNORD19B | ALOX5 | 0.3043 | 7.28E-10 |
| SNORD19B | PLXND1 | -0.3042 | 7.35E-10 |
| SNORD19B | PTHLH | -0.3042 | 7.36E-10 |
| SNORD19B | TAP2 | -0.3042 | 7.37E-10 |
| SNORD19B | PDE10A | 0.3042 | 7.42E-10 |
| SNORD19B | KLK10 | -0.3041 | 7.47E-10 |
| SNORD19B | PCGF3 | 0.3041 | 7.48E-10 |
| SNORD19B | NAT9 | 0.3041 | 7.50E-10 |
| SNORD19B | CAPZA2 | -0.304 | 7.53E-10 |
| SNORD19B | GORASP1 | 0.304 | 7.53E-10 |
| SNORD19B | LOC344595 | -0.304 | 7.54E-10 |
| SNORD19B | RFTN1 | -0.304 | 7.60E-10 |
| SNORD19B | RPL14 | 0.304 | 7.60E-10 |
| SNORD19B | MYBPC1 | 0.3039 | 7.63E-10 |
| SNORD19B | TCEAL7 | -0.3039 | 7.65E-10 |
| SNORD19B | INPP5F | -0.3039 | 7.66E-10 |
| SNORD19B | PITPNM2 | -0.3039 | 7.68E-10 |
| SNORD19B | SYNPO2 | -0.3039 | 7.68E-10 |
| SNORD19B | DNM2 | 0.3038 | 7.76E-10 |
| SNORD19B | CD248 | -0.3038 | 7.79E-10 |
| SNORD19B | KIAA1432 | -0.3038 | 7.79E-10 |
| SNORD19B | CMA1 | -0.3037 | 7.93E-10 |
| SNORD19B | GRID2IP | 0.3036 | 7.94E-10 |
| SNORD19B | IL6 | -0.3036 | 7.95E-10 |
| SNORD19B | SYT6 | -0.3036 | 7.96E-10 |
| SNORD19B | ZNF254 | 0.3035 | 8.06E-10 |
| SNORD19B | NFIC | -0.3035 | 8.09E-10 |
| SNORD19B | SELK | 0.3035 | 8.10E-10 |
| SNORD19B | CGN | 0.3034 | 8.19E-10 |
| SNORD19B | TESC | 0.3034 | 8.24E-10 |
| SNORD19B | HRH1 | -0.3034 | 8.26E-10 |
| SNORD19B | DTNA | -0.3033 | 8.29E-10 |
| SNORD19B | GPR132 | -0.3033 | 8.32E-10 |
| SNORD19B | THPO | -0.3032 | 8.44E-10 |
| SNORD19B | C19orf35 | -0.303 | 8.63E-10 |
| SNORD19B | MRGPRF | -0.303 | 8.63E-10 |
| SNORD19B | ELMO1 | -0.303 | 8.66E-10 |
| SNORD19B | PLCE1 | 0.303 | 8.70E-10 |
| SNORD19B | CRH | 0.303 | 8.71E-10 |
| SNORD19B | GRAMD1C | 0.303 | 8.71E-10 |
| SNORD19B | PLXDC1 | -0.3029 | 8.80E-10 |
| SNORD19B | SPN | -0.3029 | 8.80E-10 |
| SNORD19B | CNTLN | -0.3025 | 9.22E-10 |
| SNORD19B | STARD13 | -0.3025 | 9.28E-10 |
| SNORD19B | NLK | 0.3024 | 9.36E-10 |
| SNORD19B | SIGLEC11 | -0.3024 | 9.37E-10 |
| SNORD19B | SLC11A1 | -0.3024 | 9.38E-10 |
| SNORD19B | PCBP3 | -0.3023 | 9.51E-10 |
| SNORD19B | TBX18 | -0.3023 | 9.52E-10 |
| SNORD19B | AKAP8 | 0.3023 | 9.53E-10 |
| SNORD19B | EFHA2 | -0.3023 | 9.57E-10 |
| SNORD19B | RNF123 | 0.3021 | 9.74E-10 |
| SNORD19B | PILRA | -0.3021 | 9.77E-10 |
| SNORD19B | PER3 | -0.3021 | 9.82E-10 |
| SNORD19B | PPFIA2 | -0.302 | 9.84E-10 |
| SNORD19B | EMP1 | -0.302 | 9.85E-10 |
| SNORD19B | METTL7A | 0.302 | 9.86E-10 |
| SNORD19B | TBC1D3 | 0.302 | 9.86E-10 |
| SNORD19B | LRRC2 | -0.302 | 9.88E-10 |
| SNORD19B | ZNF692 | 0.3019 | 1.00E-09 |
| SNORD19B | FRMD4B | -0.3018 | 1.01E-09 |
| SNORD19B | CYP21A2 | -0.3018 | 1.02E-09 |
| SNORD19B | HIVEP1 | -0.3018 | 1.02E-09 |
| SNORD19B | SLC37A1 | 0.3018 | 1.02E-09 |
| SNORD19B | CNTNAP1 | -0.3017 | 1.03E-09 |
| SNORD19B | HOXD11 | -0.3017 | 1.03E-09 |
| SNORD19B | ACTR3 | -0.3016 | 1.04E-09 |
| SNORD19B | FREM2 | 0.3016 | 1.04E-09 |
| SNORD19B | ST8SIA1 | -0.3016 | 1.04E-09 |
| SNORD19B | ADAMTSL1 | -0.3015 | 1.06E-09 |
| SNORD19B | GTF2H1 | -0.3015 | 1.06E-09 |
| SNORD19B | RNH1 | -0.3015 | 1.06E-09 |
| SNORD19B | STAT1 | -0.3014 | 1.07E-09 |
| SNORD19B | MXRA5 | -0.3013 | 1.08E-09 |
| SNORD19B | RBM15B | 0.3013 | 1.08E-09 |
| SNORD19B | MAP4K3 | 0.3013 | 1.09E-09 |
| SNORD19B | SH2B3 | -0.3013 | 1.09E-09 |
| SNORD19B | FNBP1L | 0.3012 | 1.10E-09 |
| SNORD19B | SVEP1 | -0.3012 | 1.10E-09 |
| SNORD19B | KIAA0195 | 0.301 | 1.12E-09 |
| SNORD19B | RPSA | 0.3011 | 1.12E-09 |
| SNORD19B | YWHAH | -0.3011 | 1.12E-09 |
| SNORD19B | PLCD4 | -0.301 | 1.13E-09 |
| SNORD19B | C20orf46 | 0.3008 | 1.15E-09 |
| SNORD19B | MGP | -0.3009 | 1.15E-09 |
| SNORD19B | QTRT1 | 0.3009 | 1.15E-09 |
| SNORD19B | TMEM45B | 0.3007 | 1.18E-09 |
| SNORD19B | LILRB5 | -0.3006 | 1.19E-09 |
| SNORD19B | MEF2D | -0.3006 | 1.19E-09 |
| SNORD19B | ISLR2 | -0.3005 | 1.20E-09 |
| SNORD19B | GZMA | -0.3005 | 1.21E-09 |
| SNORD19B | TM6SF1 | -0.3005 | 1.21E-09 |
| SNORD19B | NBR2 | 0.3004 | 1.22E-09 |
| SNORD19B | ABLIM2 | -0.3004 | 1.23E-09 |
| SNORD19B | PDE5A | -0.3004 | 1.23E-09 |
| SNORD19B | FCRLB | 0.3003 | 1.24E-09 |
| SNORD19B | IFT20 | 0.3003 | 1.24E-09 |
| SNORD19B | TMC7 | 0.3003 | 1.24E-09 |
| SNORD19B | RBM6 | 0.3002 | 1.26E-09 |
| SNORD19B | CXXC1 | 0.3 | 1.28E-09 |
| SNORD113-9 | MEG3 | 0.486 | 1.10E-24 |
| SNORD113-9 | CPXM1 | 0.4482 | 8.12E-21 |
| SNORD113-9 | COL1A1 | 0.4226 | 1.87E-18 |
| SNORD113-9 | COL6A1 | 0.4175 | 5.21E-18 |
| SNORD113-9 | PCOLCE | 0.4174 | 5.31E-18 |
| SNORD113-9 | COL1A2 | 0.4144 | 9.62E-18 |
| SNORD113-9 | CTSK | 0.4126 | 1.38E-17 |
| SNORD113-9 | ADAMTS14 | 0.4123 | 1.45E-17 |
| SNORD113-9 | COL6A3 | 0.4112 | 1.82E-17 |
| SNORD113-9 | COL3A1 | 0.4106 | 2.05E-17 |
| SNORD113-9 | MXRA8 | 0.4104 | 2.14E-17 |
| SNORD113-9 | COL5A1 | 0.408 | 3.40E-17 |
| SNORD113-9 | THY1 | 0.4054 | 5.58E-17 |
| SNORD113-9 | COL6A2 | 0.4032 | 8.60E-17 |
| SNORD113-9 | GLT8D2 | 0.4019 | 1.09E-16 |
| SNORD113-9 | FNDC1 | 0.3977 | 2.39E-16 |
| SNORD113-9 | ADAMTS2 | 0.395 | 3.96E-16 |
| SNORD113-9 | SPON2 | 0.3935 | 5.28E-16 |
| SNORD113-9 | ISLR | 0.3933 | 5.43E-16 |
| SNORD113-9 | FAP | 0.3918 | 7.13E-16 |
| SNORD113-9 | MMP2 | 0.388 | 1.44E-15 |
| SNORD113-9 | SPARC | 0.387 | 1.72E-15 |
| SNORD113-9 | CD248 | 0.3869 | 1.74E-15 |
| SNORD113-9 | PDGFRB | 0.3865 | 1.88E-15 |
| SNORD113-9 | LRRC15 | 0.3861 | 2.04E-15 |
| SNORD113-9 | TIMP2 | 0.3848 | 2.56E-15 |
| SNORD113-9 | RARRES2 | 0.3843 | 2.78E-15 |
| SNORD113-9 | HTRA3 | 0.383 | 3.51E-15 |
| SNORD113-9 | COL5A2 | 0.382 | 4.24E-15 |
| SNORD113-9 | TWIST2 | 0.3815 | 4.64E-15 |
| SNORD113-9 | EMILIN1 | 0.3794 | 6.61E-15 |
| SNORD113-9 | SSC5D | 0.3788 | 7.46E-15 |
| SNORD113-9 | AEBP1 | 0.3778 | 8.90E-15 |
| SNORD113-9 | WNT2 | 0.3776 | 9.08E-15 |
| SNORD113-9 | ASPN | 0.3764 | 1.12E-14 |
| SNORD113-9 | ODZ3 | 0.3757 | 1.28E-14 |
| SNORD113-9 | CSMD2 | 0.3748 | 1.49E-14 |
| SNORD113-9 | TMEM119 | 0.3742 | 1.65E-14 |
| SNORD113-9 | PXDN | 0.3738 | 1.78E-14 |
| SNORD113-9 | OLFML1 | 0.3736 | 1.84E-14 |
| SNORD113-9 | SULF1 | 0.3734 | 1.89E-14 |
| SNORD113-9 | ITGA11 | 0.373 | 2.03E-14 |
| SNORD113-9 | SGCD | 0.373 | 2.03E-14 |
| SNORD113-9 | PRR16 | 0.3728 | 2.11E-14 |
| SNORD113-9 | NTM | 0.3727 | 2.14E-14 |
| SNORD113-9 | NID2 | 0.3714 | 2.68E-14 |
| SNORD113-9 | CRISPLD2 | 0.3704 | 3.15E-14 |
| SNORD113-9 | RCN3 | 0.3703 | 3.24E-14 |
| SNORD113-9 | SCARF2 | 0.3698 | 3.54E-14 |
| SNORD113-9 | CTGF | 0.369 | 4.03E-14 |
| SNORD113-9 | TMEM90B | 0.369 | 4.04E-14 |
| SNORD113-9 | CLEC11A | 0.3689 | 4.07E-14 |
| SNORD113-9 | DPT | 0.3689 | 4.11E-14 |
| SNORD113-9 | VCAN | 0.3686 | 4.30E-14 |
| SNORD113-9 | MFRP | 0.3681 | 4.68E-14 |
| SNORD113-9 | GREM1 | 0.368 | 4.74E-14 |
| SNORD113-9 | SGIP1 | 0.3677 | 4.98E-14 |
| SNORD113-9 | FMO1 | 0.3677 | 5.05E-14 |
| SNORD113-9 | NNMT | 0.3676 | 5.06E-14 |
| SNORD113-9 | CTHRC1 | 0.3672 | 5.44E-14 |
| SNORD113-9 | ZNF521 | 0.3669 | 5.76E-14 |
| SNORD113-9 | ADAMTS10 | 0.3665 | 6.17E-14 |
| SNORD113-9 | LUM | 0.3651 | 7.70E-14 |
| SNORD113-9 | DCN | 0.3646 | 8.43E-14 |
| SNORD113-9 | MRC2 | 0.3644 | 8.76E-14 |
| SNORD113-9 | COL8A1 | 0.3643 | 8.88E-14 |
| SNORD113-9 | VENTX | 0.3638 | 9.56E-14 |
| SNORD113-9 | FAM180A | 0.3638 | 9.63E-14 |
| SNORD113-9 | FIBIN | 0.362 | 1.30E-13 |
| SNORD113-9 | CCDC80 | 0.3617 | 1.37E-13 |
| SNORD113-9 | LRRC32 | 0.3616 | 1.39E-13 |
| SNORD113-9 | FSTL1 | 0.3614 | 1.44E-13 |
| SNORD113-9 | WISP1 | 0.3612 | 1.47E-13 |
| SNORD113-9 | ADAMTS16 | 0.3609 | 1.56E-13 |
| SNORD113-9 | C5orf62 | 0.3609 | 1.56E-13 |
| SNORD113-9 | MMP11 | 0.3607 | 1.61E-13 |
| SNORD113-9 | TCEAL7 | 0.3597 | 1.91E-13 |
| SNORD113-9 | CDH11 | 0.3594 | 2.00E-13 |
| SNORD113-9 | P4HA3 | 0.3591 | 2.09E-13 |
| SNORD113-9 | PPAPDC1A | 0.3591 | 2.09E-13 |
| SNORD113-9 | DACT1 | 0.3588 | 2.20E-13 |
| SNORD113-9 | TWIST1 | 0.3568 | 3.06E-13 |
| SNORD113-9 | SPON1 | 0.3566 | 3.16E-13 |
| SNORD113-9 | ZNF469 | 0.3558 | 3.59E-13 |
| SNORD113-9 | FBN1 | 0.3556 | 3.72E-13 |
| SNORD113-9 | PDPN | 0.3549 | 4.18E-13 |
| SNORD113-9 | ADAM12 | 0.3546 | 4.35E-13 |
| SNORD113-9 | MRGPRF | 0.3537 | 5.01E-13 |
| SNORD113-9 | C1QTNF1 | 0.3537 | 5.04E-13 |
| SNORD113-9 | POSTN | 0.3534 | 5.28E-13 |
| SNORD113-9 | SFRP2 | 0.3524 | 6.17E-13 |
| SNORD113-9 | PODNL1 | 0.3521 | 6.47E-13 |
| SNORD113-9 | TNFAIP6 | 0.352 | 6.58E-13 |
| SNORD113-9 | SFRP4 | 0.352 | 6.62E-13 |
| SNORD113-9 | ITGBL1 | 0.3519 | 6.70E-13 |
| SNORD113-9 | BGN | 0.3517 | 6.94E-13 |
| SNORD113-9 | JAM3 | 0.351 | 7.70E-13 |
| SNORD113-9 | PPAPDC3 | 0.351 | 7.80E-13 |
| SNORD113-9 | HSPB2 | 0.3507 | 8.08E-13 |
| SNORD113-9 | COL10A1 | 0.3496 | 9.68E-13 |
| SNORD113-9 | CCL11 | 0.3494 | 1.00E-12 |
| SNORD113-9 | MAGEL2 | 0.3488 | 1.10E-12 |
| SNORD113-9 | MMP23B | 0.3484 | 1.16E-12 |
| SNORD113-9 | HSPA12B | 0.3475 | 1.35E-12 |
| SNORD113-9 | PRRX1 | 0.3466 | 1.55E-12 |
| SNORD113-9 | C10orf72 | 0.3463 | 1.63E-12 |
| SNORD113-9 | GPC6 | 0.3463 | 1.64E-12 |
| SNORD113-9 | MSC | 0.3461 | 1.67E-12 |
| SNORD113-9 | SERPINF1 | 0.3454 | 1.87E-12 |
| SNORD113-9 | OLFML2B | 0.3452 | 1.92E-12 |
| SNORD113-9 | VIM | 0.3452 | 1.94E-12 |
| SNORD113-9 | COL11A1 | 0.3449 | 2.04E-12 |
| SNORD113-9 | BMP1 | 0.3447 | 2.08E-12 |
| SNORD113-9 | IGDCC4 | 0.3444 | 2.18E-12 |
| SNORD113-9 | LOX | 0.3444 | 2.19E-12 |
| SNORD113-9 | GGT5 | 0.3441 | 2.29E-12 |
| SNORD113-9 | KIAA1755 | 0.344 | 2.33E-12 |
| SNORD113-9 | MFAP5 | 0.3438 | 2.42E-12 |
| SNORD113-9 | FMOD | 0.3432 | 2.65E-12 |
| SNORD113-9 | STK32B | 0.3424 | 2.97E-12 |
| SNORD113-9 | RFX8 | 0.3424 | 2.99E-12 |
| SNORD113-9 | VASN | 0.3424 | 2.99E-12 |
| SNORD113-9 | LMCD1 | 0.3423 | 3.05E-12 |
| SNORD113-9 | CYR61 | 0.3421 | 3.12E-12 |
| SNORD113-9 | FBXL7 | 0.3418 | 3.27E-12 |
| SNORD113-9 | PDLIM3 | 0.3418 | 3.29E-12 |
| SNORD113-9 | CALD1 | 0.3413 | 3.55E-12 |
| SNORD113-9 | COL6A6 | 0.34 | 4.30E-12 |
| SNORD113-9 | ALDH1L2 | 0.3397 | 4.50E-12 |
| SNORD113-9 | RAB3IL1 | 0.3393 | 4.81E-12 |
| SNORD113-9 | SNAI1 | 0.3391 | 4.95E-12 |
| SNORD113-9 | OLFML3 | 0.3391 | 4.98E-12 |
| SNORD113-9 | CHST11 | 0.3386 | 5.35E-12 |
| SNORD113-9 | COLEC12 | 0.3386 | 5.39E-12 |
| SNORD113-9 | TM6SF2 | 0.3385 | 5.45E-12 |
| SNORD113-9 | PTPRN | 0.3381 | 5.76E-12 |
| SNORD113-9 | SLC11A1 | 0.3379 | 5.96E-12 |
| SNORD113-9 | TGFB3 | 0.3377 | 6.12E-12 |
| SNORD113-9 | BNC2 | 0.3377 | 6.19E-12 |
| SNORD113-9 | KCNE4 | 0.3371 | 6.72E-12 |
| SNORD113-9 | TAGLN | 0.337 | 6.81E-12 |
| SNORD113-9 | FAM26E | 0.3369 | 6.98E-12 |
| SNORD113-9 | LOXL2 | 0.3366 | 7.25E-12 |
| SNORD113-9 | UBE2QL1 | 0.3364 | 7.45E-12 |
| SNORD113-9 | OSCAR | 0.3356 | 8.44E-12 |
| SNORD113-9 | PNMAL2 | 0.3348 | 9.48E-12 |
| SNORD113-9 | CHRD | 0.3347 | 9.69E-12 |
| SNORD113-9 | ANPEP | 0.3344 | 1.01E-11 |
| SNORD113-9 | COL18A1 | 0.3341 | 1.05E-11 |
| SNORD113-9 | FPR1 | 0.3337 | 1.12E-11 |
| SNORD113-9 | ADAMTS12 | 0.3335 | 1.15E-11 |
| SNORD113-9 | TRIL | 0.333 | 1.25E-11 |
| SNORD113-9 | TSPAN4 | 0.3315 | 1.55E-11 |
| SNORD113-9 | HIC1 | 0.3313 | 1.60E-11 |
| SNORD113-9 | CHI3L1 | 0.3313 | 1.61E-11 |
| SNORD113-9 | PENK | 0.331 | 1.67E-11 |
| SNORD113-9 | COL16A1 | 0.3305 | 1.79E-11 |
| SNORD113-9 | RNF212 | 0.3303 | 1.85E-11 |
| SNORD113-9 | COL5A3 | 0.3293 | 2.15E-11 |
| SNORD113-9 | AMPH | 0.329 | 2.24E-11 |
| SNORD113-9 | CHN1 | 0.329 | 2.24E-11 |
| SNORD113-9 | GLIS1 | 0.3289 | 2.29E-11 |
| SNORD113-9 | GALNTL2 | 0.3287 | 2.35E-11 |
| SNORD113-9 | OMD | 0.328 | 2.60E-11 |
| SNORD113-9 | GFPT2 | 0.328 | 2.62E-11 |
| SNORD113-9 | PRICKLE1 | 0.3278 | 2.67E-11 |
| SNORD113-9 | CPXM2 | 0.3278 | 2.69E-11 |
| SNORD113-9 | SLC2A3 | 0.3278 | 2.70E-11 |
| SNORD113-9 | FKBP10 | 0.326 | 3.52E-11 |
| SNORD113-9 | FGF5 | 0.3257 | 3.67E-11 |
| SNORD113-9 | FGF7 | 0.3256 | 3.68E-11 |
| SNORD113-9 | FAM110B | 0.3255 | 3.76E-11 |
| SNORD113-9 | FAM176A | 0.3255 | 3.77E-11 |
| SNORD113-9 | COMP | 0.3249 | 4.09E-11 |
| SNORD113-9 | LAMA2 | 0.3248 | 4.19E-11 |
| SNORD113-9 | DOK5 | 0.3247 | 4.24E-11 |
| SNORD113-9 | MT1M | 0.3245 | 4.37E-11 |
| SNORD113-9 | ALPK2 | 0.3243 | 4.48E-11 |
| SNORD113-9 | GUCA1A | 0.3241 | 4.59E-11 |
| SNORD113-9 | PNMA2 | 0.3241 | 4.63E-11 |
| SNORD113-9 | THBS2 | 0.3238 | 4.81E-11 |
| SNORD113-9 | MFGE8 | 0.3235 | 5.04E-11 |
| SNORD113-9 | COL12A1 | 0.3234 | 5.06E-11 |
| SNORD113-9 | ATP10A | 0.3232 | 5.25E-11 |
| SNORD113-9 | PDGFRA | 0.3226 | 5.70E-11 |
| SNORD113-9 | PTGIR | 0.3225 | 5.77E-11 |
| SNORD113-9 | IFFO1 | 0.322 | 6.25E-11 |
| SNORD113-9 | MGC45800 | 0.3218 | 6.38E-11 |
| SNORD113-9 | FAM101A | 0.3218 | 6.39E-11 |
| SNORD113-9 | LOC399959 | 0.3216 | 6.64E-11 |
| SNORD113-9 | RSPO3 | 0.3215 | 6.69E-11 |
| SNORD113-9 | FILIP1L | 0.3214 | 6.80E-11 |
| SNORD113-9 | FAM19A5 | 0.3213 | 6.84E-11 |
| SNORD113-9 | ANXA6 | 0.321 | 7.18E-11 |
| SNORD113-9 | LOC100130872 | 0.3209 | 7.34E-11 |
| SNORD113-9 | FN1 | 0.3205 | 7.77E-11 |
| SNORD113-9 | CXCL12 | 0.3203 | 7.94E-11 |
| SNORD113-9 | FAM20C | 0.3201 | 8.14E-11 |
| SNORD113-9 | COX7A1 | 0.3197 | 8.67E-11 |
| SNORD113-9 | LIMS3 | 0.3194 | 9.04E-11 |
| SNORD113-9 | LGI2 | 0.3186 | 1.02E-10 |
| SNORD113-9 | HAPLN4 | 0.3185 | 1.03E-10 |
| SNORD113-9 | ECM1 | 0.3182 | 1.07E-10 |
| SNORD113-9 | ADAMTS4 | 0.3181 | 1.09E-10 |
| SNORD113-9 | GAS7 | 0.318 | 1.10E-10 |
| SNORD113-9 | TOM1 | 0.3177 | 1.15E-10 |
| SNORD113-9 | CD163 | 0.3176 | 1.17E-10 |
| SNORD113-9 | SLC16A2 | 0.3173 | 1.21E-10 |
| SNORD113-9 | SORCS2 | 0.3173 | 1.21E-10 |
| SNORD113-9 | GAS1 | 0.3173 | 1.22E-10 |
| SNORD113-9 | THBS1 | 0.3169 | 1.29E-10 |
| SNORD113-9 | TNN | 0.3162 | 1.42E-10 |
| SNORD113-9 | CRYAB | 0.3161 | 1.43E-10 |
| SNORD113-9 | MEIS3 | 0.316 | 1.46E-10 |
| SNORD113-9 | PCBP3 | 0.316 | 1.46E-10 |
| SNORD113-9 | PDGFC | 0.316 | 1.46E-10 |
| SNORD113-9 | ZFPM2 | 0.3158 | 1.49E-10 |
| SNORD113-9 | GLIPR2 | 0.3156 | 1.53E-10 |
| SNORD113-9 | AXL | 0.3155 | 1.56E-10 |
| SNORD113-9 | LRRC17 | 0.3155 | 1.56E-10 |
| SNORD113-9 | CNRIP1 | 0.3154 | 1.59E-10 |
| SNORD113-9 | TGFBI | 0.3153 | 1.62E-10 |
| SNORD113-9 | COL15A1 | 0.3152 | 1.63E-10 |
| SNORD113-9 | MSRB3 | 0.3152 | 1.63E-10 |
| SNORD113-9 | HEPH | 0.3151 | 1.66E-10 |
| SNORD113-9 | LAMA4 | 0.315 | 1.69E-10 |
| SNORD113-9 | GPR77 | 0.3147 | 1.75E-10 |
| SNORD113-9 | C5AR1 | 0.3144 | 1.81E-10 |
| SNORD113-9 | C13orf33 | 0.3143 | 1.85E-10 |
| SNORD113-9 | GXYLT2 | 0.314 | 1.93E-10 |
| SNORD113-9 | FGFR1 | 0.314 | 1.94E-10 |
| SNORD113-9 | CD300LB | 0.3139 | 1.95E-10 |
| SNORD113-9 | ANTXR1 | 0.3138 | 1.99E-10 |
| SNORD113-9 | MAP1A | 0.3136 | 2.04E-10 |
| SNORD113-9 | FGF14 | 0.3135 | 2.07E-10 |
| SNORD113-9 | VSIG4 | 0.3132 | 2.16E-10 |
| SNORD113-9 | KCNA1 | 0.313 | 2.22E-10 |
| SNORD113-9 | ANGPTL2 | 0.3129 | 2.25E-10 |
| SNORD113-9 | MOXD1 | 0.3127 | 2.31E-10 |
| SNORD113-9 | PODN | 0.3125 | 2.38E-10 |
| SNORD113-9 | STAB1 | 0.3123 | 2.44E-10 |
| SNORD113-9 | TMEM176A | 0.3123 | 2.46E-10 |
| SNORD113-9 | LGALS12 | 0.312 | 2.55E-10 |
| SNORD113-9 | BOC | 0.3118 | 2.62E-10 |
| SNORD113-9 | ADAMTSL1 | 0.3113 | 2.79E-10 |
| SNORD113-9 | ITGAX | 0.3113 | 2.82E-10 |
| SNORD113-9 | GPX8 | 0.311 | 2.93E-10 |
| SNORD113-9 | PLXDC1 | 0.3109 | 2.97E-10 |
| SNORD113-9 | DIRC1 | 0.3107 | 3.03E-10 |
| SNORD113-9 | NRP2 | 0.3107 | 3.07E-10 |
| SNORD113-9 | SHANK1 | 0.3105 | 3.13E-10 |
| SNORD113-9 | TNFSF4 | 0.3105 | 3.15E-10 |
| SNORD113-9 | CPZ | 0.3102 | 3.25E-10 |
| SNORD113-9 | GYPC | 0.3102 | 3.28E-10 |
| SNORD113-9 | HS3ST3A1 | 0.3101 | 3.32E-10 |
| SNORD113-9 | NBLA00301 | 0.3097 | 3.48E-10 |
| SNORD113-9 | IL6 | 0.3093 | 3.70E-10 |
| SNORD113-9 | DACT3 | 0.3092 | 3.74E-10 |
| SNORD113-9 | HAS1 | 0.3092 | 3.76E-10 |
| SNORD113-9 | MT1L | 0.3091 | 3.79E-10 |
| SNORD113-9 | MT2A | 0.3087 | 4.00E-10 |
| SNORD113-9 | ARSI | 0.3087 | 4.02E-10 |
| SNORD113-9 | EFEMP1 | 0.3085 | 4.14E-10 |
| SNORD113-9 | FAM20A | 0.3084 | 4.15E-10 |
| SNORD113-9 | FAM167A | 0.3082 | 4.30E-10 |
| SNORD113-9 | KANK4 | 0.3082 | 4.30E-10 |
| SNORD113-9 | PLOD1 | 0.3082 | 4.31E-10 |
| SNORD113-9 | MMP9 | 0.3081 | 4.34E-10 |
| SNORD113-9 | FXYD6 | 0.3078 | 4.56E-10 |
| SNORD113-9 | PAPSS2 | 0.3077 | 4.57E-10 |
| SNORD113-9 | C1QTNF6 | 0.3075 | 4.71E-10 |
| SNORD113-9 | LILRB3 | 0.3074 | 4.79E-10 |
| SNORD113-9 | PDZRN3 | 0.3074 | 4.80E-10 |
| SNORD113-9 | LILRA2 | 0.307 | 5.05E-10 |
| SNORD113-9 | MXRA5 | 0.3069 | 5.13E-10 |
| SNORD113-9 | IGFL2 | 0.3067 | 5.28E-10 |
| SNORD113-9 | PDLIM2 | 0.3066 | 5.36E-10 |
| SNORD113-9 | C13orf18 | 0.3063 | 5.53E-10 |
| SNORD113-9 | LYVE1 | 0.3058 | 5.92E-10 |
| SNORD113-9 | WBSCR17 | 0.3058 | 5.96E-10 |
| SNORD113-9 | MYL9 | 0.3054 | 6.24E-10 |
| SNORD113-9 | ADAM33 | 0.3047 | 6.90E-10 |
| SNORD113-9 | HTRA1 | 0.3046 | 6.97E-10 |
| SNORD113-9 | SYT11 | 0.3045 | 7.09E-10 |
| SNORD113-9 | KIAA1644 | 0.3045 | 7.10E-10 |
| SNORD113-9 | CSDC2 | 0.3044 | 7.15E-10 |
| SNORD113-9 | PDGFRL | 0.3044 | 7.16E-10 |
| SNORD113-9 | DKK3 | 0.3041 | 7.49E-10 |
| SNORD113-9 | TMEM176B | 0.3041 | 7.51E-10 |
| SNORD113-9 | PLXND1 | 0.3039 | 7.67E-10 |
| SNORD113-9 | TMEM158 | 0.3039 | 7.68E-10 |
| SNORD113-9 | MRC1 | 0.3036 | 7.95E-10 |
| SNORD113-9 | NTNG2 | 0.3036 | 8.01E-10 |
| SNORD113-9 | ECM2 | 0.3033 | 8.27E-10 |
| SNORD113-9 | OPCML | 0.303 | 8.67E-10 |
| SNORD113-9 | OGN | 0.3029 | 8.81E-10 |
| SNORD113-9 | XIRP1 | 0.3028 | 8.90E-10 |
| SNORD113-9 | LOXL3 | 0.3027 | 9.07E-10 |
| SNORD113-9 | ST6GALNAC5 | 0.3026 | 9.17E-10 |
| SNORD113-9 | EPYC | 0.3025 | 9.26E-10 |
| SNORD113-9 | MFAP4 | 0.3018 | 1.01E-09 |
| SNORD113-9 | IFITM2 | 0.3018 | 1.02E-09 |
| SNORD113-9 | ACTA2 | 0.3014 | 1.07E-09 |
| SNORD113-9 | DPYSL2 | 0.3011 | 1.11E-09 |
| SNORD113-9 | COL14A1 | 0.3009 | 1.15E-09 |
| SNORD113-9 | GLT1D1 | 0.3006 | 1.19E-09 |
| SNORD113-9 | TRPV2 | 0.3003 | 1.23E-09 |
| SNORD113-9 | SCN1B | 0.3002 | 1.25E-09 |
| SNORD113-9 | APCDD1L | 0.3002 | 1.26E-09 |
| SNORD113-9 | GNG2 | 0.3002 | 1.26E-09 |
| SNORD113-9 | GDF10 | 0.3001 | 1.27E-09 |
| SNORD114-1 | INHBA | 0.4391 | 5.91E-20 |
| SNORD114-1 | ALDH1L2 | 0.4241 | 1.39E-18 |
| SNORD114-1 | COL3A1 | 0.4208 | 2.67E-18 |
| SNORD114-1 | COL5A1 | 0.4181 | 4.65E-18 |
| SNORD114-1 | EIF5B | 0.4152 | 8.21E-18 |
| SNORD114-1 | VCAN | 0.4128 | 1.34E-17 |
| SNORD114-1 | COL1A1 | 0.4105 | 2.09E-17 |
| SNORD114-1 | ADAMTS12 | 0.4098 | 2.38E-17 |
| SNORD114-1 | ANKRD9 | -0.4086 | 3.02E-17 |
| SNORD114-1 | ZNF469 | 0.4081 | 3.31E-17 |
| SNORD114-1 | ODZ3 | 0.4077 | 3.56E-17 |
| SNORD114-1 | PRRX1 | 0.4073 | 3.86E-17 |
| SNORD114-1 | COL1A2 | 0.4067 | 4.32E-17 |
| SNORD114-1 | COL6A3 | 0.4049 | 6.22E-17 |
| SNORD114-1 | FLRT2 | 0.4039 | 7.46E-17 |
| SNORD114-1 | COL5A2 | 0.403 | 8.94E-17 |
| SNORD114-1 | ADAMTS2 | 0.4029 | 9.04E-17 |
| SNORD114-1 | LUM | 0.3978 | 2.38E-16 |
| SNORD114-1 | GLT8D2 | 0.3974 | 2.56E-16 |
| SNORD114-1 | COL8A1 | 0.397 | 2.77E-16 |
| SNORD114-1 | ZNF414 | -0.3962 | 3.21E-16 |
| SNORD114-1 | C19orf6 | -0.3959 | 3.36E-16 |
| SNORD114-1 | WISP1 | 0.3959 | 3.37E-16 |
| SNORD114-1 | CDH11 | 0.3958 | 3.43E-16 |
| SNORD114-1 | ZNF521 | 0.3924 | 6.46E-16 |
| SNORD114-1 | NSUN5P2 | -0.3922 | 6.68E-16 |
| SNORD114-1 | CDR1 | 0.3918 | 7.23E-16 |
| SNORD114-1 | SPON1 | 0.3885 | 1.31E-15 |
| SNORD114-1 | DDR2 | 0.3884 | 1.33E-15 |
| SNORD114-1 | CALD1 | 0.3866 | 1.84E-15 |
| SNORD114-1 | FAM26E | 0.3863 | 1.96E-15 |
| SNORD114-1 | NSUN5P1 | -0.3853 | 2.34E-15 |
| SNORD114-1 | PXDN | 0.3853 | 2.35E-15 |
| SNORD114-1 | SULF1 | 0.3846 | 2.66E-15 |
| SNORD114-1 | PDGFRB | 0.3834 | 3.27E-15 |
| SNORD114-1 | SGCD | 0.3823 | 4.03E-15 |
| SNORD114-1 | CDK5RAP3 | -0.3818 | 4.33E-15 |
| SNORD114-1 | PRKG1 | 0.3815 | 4.57E-15 |
| SNORD114-1 | ADAM12 | 0.3811 | 4.99E-15 |
| SNORD114-1 | DCN | 0.3801 | 5.93E-15 |
| SNORD114-1 | CPXM1 | 0.3798 | 6.18E-15 |
| SNORD114-1 | DDTL | -0.3791 | 7.08E-15 |
| SNORD114-1 | FKBP9 | 0.3784 | 7.92E-15 |
| SNORD114-1 | CCDC130 | -0.3784 | 7.99E-15 |
| SNORD114-1 | FBN1 | 0.3781 | 8.35E-15 |
| SNORD114-1 | ITGA11 | 0.3775 | 9.35E-15 |
| SNORD114-1 | KANK4 | 0.376 | 1.21E-14 |
| SNORD114-1 | MYO5A | 0.3758 | 1.25E-14 |
| SNORD114-1 | TCF4 | 0.3758 | 1.25E-14 |
| SNORD114-1 | DKK2 | 0.3752 | 1.40E-14 |
| SNORD114-1 | AKAP8L | -0.3749 | 1.45E-14 |
| SNORD114-1 | CTSK | 0.3749 | 1.45E-14 |
| SNORD114-1 | MGC45800 | 0.3748 | 1.48E-14 |
| SNORD114-1 | GREM1 | 0.3746 | 1.53E-14 |
| SNORD114-1 | LOX | 0.3744 | 1.59E-14 |
| SNORD114-1 | C19orf60 | -0.3743 | 1.61E-14 |
| SNORD114-1 | NID2 | 0.374 | 1.71E-14 |
| SNORD114-1 | NTM | 0.374 | 1.72E-14 |
| SNORD114-1 | ALPK2 | 0.3735 | 1.86E-14 |
| SNORD114-1 | P4HA3 | 0.3719 | 2.46E-14 |
| SNORD114-1 | FAP | 0.3709 | 2.92E-14 |
| SNORD114-1 | PLBD2 | 0.3705 | 3.10E-14 |
| SNORD114-1 | THBS2 | 0.3704 | 3.15E-14 |
| SNORD114-1 | GPC6 | 0.3698 | 3.52E-14 |
| SNORD114-1 | LRRC14 | -0.3696 | 3.60E-14 |
| SNORD114-1 | MST1P2 | -0.369 | 4.05E-14 |
| SNORD114-1 | LRRC15 | 0.3688 | 4.16E-14 |
| SNORD114-1 | TNFAIP6 | 0.3687 | 4.20E-14 |
| SNORD114-1 | OLFML1 | 0.3687 | 4.22E-14 |
| SNORD114-1 | SPARC | 0.3685 | 4.40E-14 |
| SNORD114-1 | FNDC1 | 0.3684 | 4.46E-14 |
| SNORD114-1 | POU2F2 | 0.3682 | 4.60E-14 |
| SNORD114-1 | PAPPA | 0.3681 | 4.65E-14 |
| SNORD114-1 | COL11A1 | 0.3678 | 4.97E-14 |
| SNORD114-1 | COL10A1 | 0.3676 | 5.10E-14 |
| SNORD114-1 | KIAA1875 | -0.3676 | 5.13E-14 |
| SNORD114-1 | OSBPL8 | 0.3674 | 5.26E-14 |
| SNORD114-1 | EDNRA | 0.3671 | 5.58E-14 |
| SNORD114-1 | LPAR4 | 0.3664 | 6.21E-14 |
| SNORD114-1 | IL7R | 0.3661 | 6.53E-14 |
| SNORD114-1 | MSRB3 | 0.3661 | 6.55E-14 |
| SNORD114-1 | MN1 | 0.3658 | 6.84E-14 |
| SNORD114-1 | ZFPM2 | 0.3653 | 7.46E-14 |
| SNORD114-1 | ERC1 | 0.3651 | 7.78E-14 |
| SNORD114-1 | SNRNP70 | -0.3647 | 8.25E-14 |
| SNORD114-1 | SSC5D | 0.3645 | 8.53E-14 |
| SNORD114-1 | POSTN | 0.364 | 9.35E-14 |
| SNORD114-1 | SFTPC | 0.3631 | 1.08E-13 |
| SNORD114-1 | ECM2 | 0.3627 | 1.17E-13 |
| SNORD114-1 | FKBP9L | 0.3624 | 1.21E-13 |
| SNORD114-1 | ECHDC2 | -0.3622 | 1.26E-13 |
| SNORD114-1 | AES | -0.3616 | 1.40E-13 |
| SNORD114-1 | ZNF653 | -0.3615 | 1.41E-13 |
| SNORD114-1 | MFAP5 | 0.3608 | 1.57E-13 |
| SNORD114-1 | ASPN | 0.3596 | 1.94E-13 |
| SNORD114-1 | IGDCC4 | 0.359 | 2.13E-13 |
| SNORD114-1 | AEBP1 | 0.3584 | 2.33E-13 |
| SNORD114-1 | SGIP1 | 0.3562 | 3.35E-13 |
| SNORD114-1 | PRR16 | 0.356 | 3.48E-13 |
| SNORD114-1 | MAP1A | 0.3558 | 3.60E-13 |
| SNORD114-1 | MRPL34 | -0.3556 | 3.72E-13 |
| SNORD114-1 | ZNF76 | -0.3554 | 3.83E-13 |
| SNORD114-1 | GLI2 | 0.3547 | 4.26E-13 |
| SNORD114-1 | LPPR4 | 0.3543 | 4.56E-13 |
| SNORD114-1 | PDPN | 0.3543 | 4.57E-13 |
| SNORD114-1 | C1orf63 | -0.3538 | 4.97E-13 |
| SNORD114-1 | CALU | 0.3536 | 5.13E-13 |
| SNORD114-1 | FAM180A | 0.3528 | 5.84E-13 |
| SNORD114-1 | BCAT1 | 0.3525 | 6.12E-13 |
| SNORD114-1 | THBS1 | 0.3524 | 6.19E-13 |
| SNORD114-1 | CCL11 | 0.3523 | 6.33E-13 |
| SNORD114-1 | CSMD2 | 0.3516 | 7.08E-13 |
| SNORD114-1 | KCTD10 | 0.3512 | 7.54E-13 |
| SNORD114-1 | SAFB2 | -0.3512 | 7.56E-13 |
| SNORD114-1 | STK11 | -0.3509 | 7.88E-13 |
| SNORD114-1 | COL12A1 | 0.3502 | 8.81E-13 |
| SNORD114-1 | TWIST2 | 0.3502 | 8.87E-13 |
| SNORD114-1 | CHST15 | 0.3501 | 8.94E-13 |
| SNORD114-1 | GUCY1B3 | 0.35 | 9.11E-13 |
| SNORD114-1 | DIRC1 | 0.3498 | 9.39E-13 |
| SNORD114-1 | OLFML3 | 0.3489 | 1.07E-12 |
| SNORD114-1 | C10orf72 | 0.3489 | 1.09E-12 |
| SNORD114-1 | CTHRC1 | 0.3487 | 1.12E-12 |
| SNORD114-1 | RUNX1T1 | 0.3484 | 1.17E-12 |
| SNORD114-1 | SVEP1 | 0.3484 | 1.17E-12 |
| SNORD114-1 | BNC2 | 0.3482 | 1.20E-12 |
| SNORD114-1 | COL5A3 | 0.3482 | 1.21E-12 |
| SNORD114-1 | CCDC80 | 0.3473 | 1.39E-12 |
| SNORD114-1 | NR2F6 | -0.347 | 1.46E-12 |
| SNORD114-1 | HAPLN4 | 0.3468 | 1.51E-12 |
| SNORD114-1 | SFRS16 | -0.3468 | 1.51E-12 |
| SNORD114-1 | GFPT2 | 0.3466 | 1.54E-12 |
| SNORD114-1 | CH25H | 0.346 | 1.70E-12 |
| SNORD114-1 | COL15A1 | 0.3457 | 1.79E-12 |
| SNORD114-1 | MXRA5 | 0.3456 | 1.81E-12 |
| SNORD114-1 | FGF7 | 0.3455 | 1.84E-12 |
| SNORD114-1 | HEPH | 0.3455 | 1.86E-12 |
| SNORD114-1 | FIBIN | 0.3452 | 1.94E-12 |
| SNORD114-1 | C19orf36 | -0.3449 | 2.01E-12 |
| SNORD114-1 | COL14A1 | 0.3448 | 2.06E-12 |
| SNORD114-1 | PLEKHH3 | -0.3442 | 2.26E-12 |
| SNORD114-1 | C19orf20 | -0.3438 | 2.40E-12 |
| SNORD114-1 | ISLR | 0.3435 | 2.53E-12 |
| SNORD114-1 | GAS7 | 0.3434 | 2.58E-12 |
| SNORD114-1 | FILIP1L | 0.3433 | 2.62E-12 |
| SNORD114-1 | CD248 | 0.3428 | 2.81E-12 |
| SNORD114-1 | NCRNA00105 | -0.3428 | 2.82E-12 |
| SNORD114-1 | ADAMTS16 | 0.3428 | 2.83E-12 |
| SNORD114-1 | SLC39A14 | 0.3424 | 2.98E-12 |
| SNORD114-1 | KIRREL | 0.3424 | 3.00E-12 |
| SNORD114-1 | SNAI1 | 0.3422 | 3.09E-12 |
| SNORD114-1 | LRRC32 | 0.3421 | 3.13E-12 |
| SNORD114-1 | TTLL3 | -0.3418 | 3.28E-12 |
| SNORD114-1 | LPP | 0.3416 | 3.37E-12 |
| SNORD114-1 | MZF1 | -0.3414 | 3.48E-12 |
| SNORD114-1 | HOOK2 | -0.3411 | 3.68E-12 |
| SNORD114-1 | PPP1R16A | -0.3409 | 3.76E-12 |
| SNORD114-1 | ETV1 | 0.3409 | 3.79E-12 |
| SNORD114-1 | HS3ST3A1 | 0.3409 | 3.79E-12 |
| SNORD114-1 | ST6GALNAC5 | 0.3408 | 3.83E-12 |
| SNORD114-1 | ZNF692 | -0.3407 | 3.87E-12 |
| SNORD114-1 | ABCC9 | 0.3404 | 4.04E-12 |
| SNORD114-1 | DKK3 | 0.3404 | 4.08E-12 |
| SNORD114-1 | LOC146880 | -0.3403 | 4.15E-12 |
| SNORD114-1 | RPLP0P2 | 0.3402 | 4.18E-12 |
| SNORD114-1 | EDIL3 | 0.3396 | 4.62E-12 |
| SNORD114-1 | MIB2 | -0.3396 | 4.62E-12 |
| SNORD114-1 | EIF5A2 | 0.3395 | 4.64E-12 |
| SNORD114-1 | BGN | 0.3394 | 4.75E-12 |
| SNORD114-1 | FGF5 | 0.3394 | 4.76E-12 |
| SNORD114-1 | SDHAP1 | -0.3391 | 4.97E-12 |
| SNORD114-1 | ANPEP | 0.339 | 5.06E-12 |
| SNORD114-1 | LAMA2 | 0.3389 | 5.11E-12 |
| SNORD114-1 | GUCY1A3 | 0.3387 | 5.30E-12 |
| SNORD114-1 | SFRP2 | 0.3386 | 5.33E-12 |
| SNORD114-1 | ANTXR1 | 0.3386 | 5.38E-12 |
| SNORD114-1 | TGFB3 | 0.3384 | 5.50E-12 |
| SNORD114-1 | THY1 | 0.3384 | 5.52E-12 |
| SNORD114-1 | FMOD | 0.338 | 5.90E-12 |
| SNORD114-1 | CHSY1 | 0.3376 | 6.28E-12 |
| SNORD114-1 | PRDX2 | -0.3375 | 6.34E-12 |
| SNORD114-1 | GPR176 | 0.3371 | 6.76E-12 |
| SNORD114-1 | BICC1 | 0.3367 | 7.12E-12 |
| SNORD114-1 | GNB4 | 0.3366 | 7.27E-12 |
| SNORD114-1 | PDLIM3 | 0.3366 | 7.29E-12 |
| SNORD114-1 | TMEM119 | 0.3362 | 7.67E-12 |
| SNORD114-1 | MRI1 | -0.3359 | 8.05E-12 |
| SNORD114-1 | DACT1 | 0.3359 | 8.11E-12 |
| SNORD114-1 | PID1 | 0.3357 | 8.36E-12 |
| SNORD114-1 | NRP2 | 0.3356 | 8.43E-12 |
| SNORD114-1 | CRISPLD2 | 0.3349 | 9.41E-12 |
| SNORD114-1 | PAPSS2 | 0.3345 | 9.91E-12 |
| SNORD114-1 | SACS | 0.3344 | 1.01E-11 |
| SNORD114-1 | CHN1 | 0.3341 | 1.05E-11 |
| SNORD114-1 | MMP2 | 0.3342 | 1.05E-11 |
| SNORD114-1 | PALLD | 0.3338 | 1.10E-11 |
| SNORD114-1 | MAP1B | 0.3338 | 1.11E-11 |
| SNORD114-1 | ADAMTS14 | 0.3337 | 1.12E-11 |
| SNORD114-1 | C7orf10 | 0.3337 | 1.12E-11 |
| SNORD114-1 | TWIST1 | 0.3334 | 1.18E-11 |
| SNORD114-1 | PDGFC | 0.3331 | 1.22E-11 |
| SNORD114-1 | RCN3 | 0.333 | 1.24E-11 |
| SNORD114-1 | TMEM90B | 0.333 | 1.25E-11 |
| SNORD114-1 | FN1 | 0.3327 | 1.31E-11 |
| SNORD114-1 | C1S | 0.3321 | 1.42E-11 |
| SNORD114-1 | CCIN | 0.3319 | 1.46E-11 |
| SNORD114-1 | GLIPR1 | 0.3317 | 1.52E-11 |
| SNORD114-1 | FAM193B | -0.3315 | 1.55E-11 |
| SNORD114-1 | FAT4 | 0.3314 | 1.57E-11 |
| SNORD114-1 | SGTB | 0.3312 | 1.63E-11 |
| SNORD114-1 | ARSI | 0.3311 | 1.65E-11 |
| SNORD114-1 | CC2D1A | -0.3311 | 1.66E-11 |
| SNORD114-1 | SLC30A4 | 0.3311 | 1.66E-11 |
| SNORD114-1 | TRAM2 | 0.3309 | 1.69E-11 |
| SNORD114-1 | NOTCH2 | 0.3307 | 1.76E-11 |
| SNORD114-1 | RGL2 | -0.3303 | 1.87E-11 |
| SNORD114-1 | HEXDC | -0.3301 | 1.93E-11 |
| SNORD114-1 | OLFML2B | 0.3297 | 2.05E-11 |
| SNORD114-1 | CCDC8 | 0.3295 | 2.09E-11 |
| SNORD114-1 | NUAK1 | 0.3294 | 2.11E-11 |
| SNORD114-1 | SNHG12 | -0.3294 | 2.13E-11 |
| SNORD114-1 | ZEB2 | 0.3292 | 2.19E-11 |
| SNORD114-1 | ATG16L2 | -0.3292 | 2.20E-11 |
| SNORD114-1 | STK17B | 0.329 | 2.24E-11 |
| SNORD114-1 | NID1 | 0.329 | 2.27E-11 |
| SNORD114-1 | ARSJ | 0.3289 | 2.29E-11 |
| SNORD114-1 | C6orf136 | -0.3286 | 2.40E-11 |
| SNORD114-1 | GPX8 | 0.3283 | 2.52E-11 |
| SNORD114-1 | WNT2 | 0.3281 | 2.58E-11 |
| SNORD114-1 | PPAPDC1A | 0.328 | 2.59E-11 |
| SNORD114-1 | ADAT3 | -0.3274 | 2.87E-11 |
| SNORD114-1 | KAL1 | 0.3273 | 2.88E-11 |
| SNORD114-1 | BACH2 | 0.3273 | 2.91E-11 |
| SNORD114-1 | TLL1 | 0.3272 | 2.95E-11 |
| SNORD114-1 | PTGFR | 0.3271 | 2.97E-11 |
| SNORD114-1 | HSPA13 | 0.3271 | 2.99E-11 |
| SNORD114-1 | ARL4C | 0.327 | 3.03E-11 |
| SNORD114-1 | MSC | 0.3268 | 3.13E-11 |
| SNORD114-1 | COL6A1 | 0.3261 | 3.43E-11 |
| SNORD114-1 | FSTL1 | 0.3261 | 3.47E-11 |
| SNORD114-1 | NEGR1 | 0.326 | 3.51E-11 |
| SNORD114-1 | OSBPL6 | 0.326 | 3.52E-11 |
| SNORD114-1 | COL6A2 | 0.3259 | 3.54E-11 |
| SNORD114-1 | ARSB | 0.3257 | 3.67E-11 |
| SNORD114-1 | ADCK5 | -0.3256 | 3.70E-11 |
| SNORD114-1 | LTBP2 | 0.3255 | 3.78E-11 |
| SNORD114-1 | POLRMT | -0.3254 | 3.79E-11 |
| SNORD114-1 | PILRB | -0.3254 | 3.82E-11 |
| SNORD114-1 | PCDHGA12 | 0.3252 | 3.95E-11 |
| SNORD114-1 | SEC62 | 0.3251 | 3.96E-11 |
| SNORD114-1 | PABPC4L | 0.3251 | 4.01E-11 |
| SNORD114-1 | ZAK | 0.3246 | 4.31E-11 |
| SNORD114-1 | PPFIA2 | 0.3244 | 4.38E-11 |
| SNORD114-1 | ITGA4 | 0.3244 | 4.39E-11 |
| SNORD114-1 | SHANK1 | 0.3243 | 4.47E-11 |
| SNORD114-1 | MYLK | 0.3243 | 4.50E-11 |
| SNORD114-1 | OMD | 0.3237 | 4.89E-11 |
| SNORD114-1 | PDZRN3 | 0.3236 | 4.94E-11 |
| SNORD114-1 | PABPC1L | -0.3235 | 5.03E-11 |
| SNORD114-1 | EGFL8 | -0.3234 | 5.09E-11 |
| SNORD114-1 | COL8A2 | 0.3234 | 5.11E-11 |
| SNORD114-1 | CHST11 | 0.3232 | 5.22E-11 |
| SNORD114-1 | TIMP2 | 0.3231 | 5.34E-11 |
| SNORD114-1 | PRRX2 | 0.3227 | 5.64E-11 |
| SNORD114-1 | HGF | 0.3226 | 5.69E-11 |
| SNORD114-1 | PCOLCE | 0.3225 | 5.76E-11 |
| SNORD114-1 | JAM3 | 0.3224 | 5.92E-11 |
| SNORD114-1 | RAB3IL1 | 0.3221 | 6.17E-11 |
| SNORD114-1 | DSE | 0.322 | 6.23E-11 |
| SNORD114-1 | FAM108A1 | -0.322 | 6.27E-11 |
| SNORD114-1 | F2R | 0.3219 | 6.35E-11 |
| SNORD114-1 | ITGAV | 0.3218 | 6.39E-11 |
| SNORD114-1 | PLS3 | 0.3217 | 6.52E-11 |
| SNORD114-1 | FBXL7 | 0.3216 | 6.63E-11 |
| SNORD114-1 | CSAD | -0.3209 | 7.35E-11 |
| SNORD114-1 | AXL | 0.3207 | 7.51E-11 |
| SNORD114-1 | FKBP14 | 0.3206 | 7.57E-11 |
| SNORD114-1 | GAS1 | 0.3206 | 7.64E-11 |
| SNORD114-1 | ZMAT3 | 0.3205 | 7.72E-11 |
| SNORD114-1 | CTGF | 0.3205 | 7.74E-11 |
| SNORD114-1 | PALM2 | 0.3202 | 8.05E-11 |
| SNORD114-1 | PRDM1 | 0.3201 | 8.18E-11 |
| SNORD114-1 | FAM167A | 0.32 | 8.34E-11 |
| SNORD114-1 | KIAA1644 | 0.32 | 8.34E-11 |
| SNORD114-1 | CPEB1 | 0.3199 | 8.37E-11 |
| SNORD114-1 | KAT2A | -0.3199 | 8.40E-11 |
| SNORD114-1 | PLXDC2 | 0.3198 | 8.48E-11 |
| SNORD114-1 | EBF1 | 0.3197 | 8.62E-11 |
| SNORD114-1 | AP1S2 | 0.3197 | 8.66E-11 |
| SNORD114-1 | C1orf159 | -0.3196 | 8.78E-11 |
| SNORD114-1 | PCDH17 | 0.3194 | 9.03E-11 |
| SNORD114-1 | PDE3A | 0.3193 | 9.12E-11 |
| SNORD114-1 | ZNF193 | -0.3193 | 9.13E-11 |
| SNORD114-1 | NUDT11 | 0.3185 | 1.03E-10 |
| SNORD114-1 | C6orf59 | 0.318 | 1.10E-10 |
| SNORD114-1 | CHKB | -0.318 | 1.10E-10 |
| SNORD114-1 | MYH9 | 0.318 | 1.11E-10 |
| SNORD114-1 | HEG1 | 0.3178 | 1.13E-10 |
| SNORD114-1 | CHSY3 | 0.3177 | 1.15E-10 |
| SNORD114-1 | MYADM | 0.3176 | 1.16E-10 |
| SNORD114-1 | FKBP10 | 0.3176 | 1.17E-10 |
| SNORD114-1 | QTRT1 | -0.3176 | 1.17E-10 |
| SNORD114-1 | FBLN2 | 0.3172 | 1.23E-10 |
| SNORD114-1 | TBC1D3B | -0.3169 | 1.28E-10 |
| SNORD114-1 | NEIL1 | -0.3169 | 1.29E-10 |
| SNORD114-1 | C3orf47 | -0.3168 | 1.30E-10 |
| SNORD114-1 | GLIS1 | 0.3168 | 1.30E-10 |
| SNORD114-1 | ARHGAP31 | 0.3167 | 1.32E-10 |
| SNORD114-1 | LEF1 | 0.3166 | 1.33E-10 |
| SNORD114-1 | TRPS1 | 0.3166 | 1.34E-10 |
| SNORD114-1 | VCAM1 | 0.3164 | 1.38E-10 |
| SNORD114-1 | ITGA1 | 0.316 | 1.45E-10 |
| SNORD114-1 | SELO | -0.316 | 1.45E-10 |
| SNORD114-1 | GXYLT2 | 0.3156 | 1.55E-10 |
| SNORD114-1 | STOM | 0.3154 | 1.58E-10 |
| SNORD114-1 | TNC | 0.3154 | 1.59E-10 |
| SNORD114-1 | ALKBH7 | -0.3152 | 1.62E-10 |
| SNORD114-1 | MXRA8 | 0.3153 | 1.62E-10 |
| SNORD114-1 | S100A1 | -0.3152 | 1.63E-10 |
| SNORD114-1 | VIM | 0.3152 | 1.63E-10 |
| SNORD114-1 | LOXL2 | 0.3152 | 1.64E-10 |
| SNORD114-1 | DPT | 0.3151 | 1.66E-10 |
| SNORD114-1 | CHI3L1 | 0.3149 | 1.70E-10 |
| SNORD114-1 | ASAM | 0.3147 | 1.74E-10 |
| SNORD114-1 | HMCN1 | 0.3145 | 1.79E-10 |
| SNORD114-1 | MRC2 | 0.3145 | 1.80E-10 |
| SNORD114-1 | RGS4 | 0.3143 | 1.86E-10 |
| SNORD114-1 | ITPR2 | 0.3142 | 1.87E-10 |
| SNORD114-1 | AMPH | 0.3142 | 1.89E-10 |
| SNORD114-1 | COLEC12 | 0.3141 | 1.91E-10 |
| SNORD114-1 | LOC399959 | 0.3141 | 1.91E-10 |
| SNORD114-1 | RASGRF2 | 0.3138 | 1.99E-10 |
| SNORD114-1 | TMEM26 | 0.3138 | 1.99E-10 |
| SNORD114-1 | PMEPA1 | 0.3136 | 2.05E-10 |
| SNORD114-1 | ZEB1 | 0.3134 | 2.09E-10 |
| SNORD114-1 | FANCL | -0.3134 | 2.11E-10 |
| SNORD114-1 | PRICKLE1 | 0.3134 | 2.11E-10 |
| SNORD114-1 | SULF2 | 0.3134 | 2.11E-10 |
| SNORD114-1 | MSN | 0.3133 | 2.12E-10 |
| SNORD114-1 | STK32B | 0.3133 | 2.13E-10 |
| SNORD114-1 | 7-Sep | 0.3132 | 2.14E-10 |
| SNORD114-1 | GPR1 | 0.3128 | 2.29E-10 |
| SNORD114-1 | IP6K2 | -0.3127 | 2.33E-10 |
| SNORD114-1 | CORO1C | 0.3125 | 2.38E-10 |
| SNORD114-1 | GPR183 | 0.3124 | 2.43E-10 |
| SNORD114-1 | ENPEP | 0.3122 | 2.48E-10 |
| SNORD114-1 | CAMK2A | 0.3121 | 2.50E-10 |
| SNORD114-1 | AHSA2 | -0.3121 | 2.51E-10 |
| SNORD114-1 | CSGALNACT2 | 0.3116 | 2.71E-10 |
| SNORD114-1 | FAM70A | 0.3116 | 2.71E-10 |
| SNORD114-1 | RBM6 | -0.3113 | 2.80E-10 |
| SNORD114-1 | ENGASE | -0.3112 | 2.83E-10 |
| SNORD114-1 | FGFR1 | 0.3112 | 2.86E-10 |
| SNORD114-1 | SERPINF1 | 0.3108 | 2.99E-10 |
| SNORD114-1 | C21orf34 | 0.3108 | 3.02E-10 |
| SNORD114-1 | HOXB6 | -0.3105 | 3.14E-10 |
| SNORD114-1 | TGFBI | 0.3103 | 3.21E-10 |
| SNORD114-1 | AKAP2 | 0.31 | 3.34E-10 |
| SNORD114-1 | CRB3 | -0.3099 | 3.40E-10 |
| SNORD114-1 | ELK3 | 0.3099 | 3.42E-10 |
| SNORD114-1 | LATS2 | 0.3098 | 3.47E-10 |
| SNORD114-1 | SIRPA | 0.3097 | 3.48E-10 |
| SNORD114-1 | ADAM19 | 0.3095 | 3.57E-10 |
| SNORD114-1 | WBSCR17 | 0.3095 | 3.60E-10 |
| SNORD114-1 | KIF26B | 0.3095 | 3.61E-10 |
| SNORD114-1 | RECK | 0.3093 | 3.67E-10 |
| SNORD114-1 | SLC2A3 | 0.3093 | 3.69E-10 |
| SNORD114-1 | ADAMTSL1 | 0.3088 | 3.95E-10 |
| SNORD114-1 | SCXB | -0.3087 | 4.03E-10 |
| SNORD114-1 | P4HTM | -0.3085 | 4.14E-10 |
| SNORD114-1 | KIAA0907 | -0.3084 | 4.16E-10 |
| SNORD114-1 | HS3ST3B1 | 0.3083 | 4.25E-10 |
| SNORD114-1 | PTRF | 0.3082 | 4.28E-10 |
| SNORD114-1 | CPXM2 | 0.3081 | 4.34E-10 |
| SNORD114-1 | RHPN1 | -0.3081 | 4.34E-10 |
| SNORD114-1 | TAF6L | -0.3081 | 4.34E-10 |
| SNORD114-1 | BAT1 | -0.3079 | 4.48E-10 |
| SNORD114-1 | CCNL2 | -0.3078 | 4.51E-10 |
| SNORD114-1 | DHPS | -0.3078 | 4.55E-10 |
| SNORD114-1 | ME3 | -0.3075 | 4.75E-10 |
| SNORD114-1 | AKAP8 | -0.3074 | 4.78E-10 |
| SNORD114-1 | SFRP4 | 0.3073 | 4.82E-10 |
| SNORD114-1 | FOXC2 | 0.3072 | 4.89E-10 |
| SNORD114-1 | MMP16 | 0.3068 | 5.18E-10 |
| SNORD114-1 | CLIC4 | 0.3066 | 5.33E-10 |
| SNORD114-1 | WIPF1 | 0.3065 | 5.42E-10 |
| SNORD114-1 | PLXNC1 | 0.3063 | 5.52E-10 |
| SNORD114-1 | RSPO3 | 0.3062 | 5.62E-10 |
| SNORD114-1 | GNG2 | 0.306 | 5.82E-10 |
| SNORD114-1 | SCARF2 | 0.3057 | 6.04E-10 |
| SNORD114-1 | GTPBP3 | -0.3056 | 6.15E-10 |
| SNORD114-1 | GULP1 | 0.3054 | 6.25E-10 |
| SNORD114-1 | PTPN11 | 0.3053 | 6.33E-10 |
| SNORD114-1 | FAM126A | 0.3053 | 6.36E-10 |
| SNORD114-1 | PODNL1 | 0.3053 | 6.37E-10 |
| SNORD114-1 | ROR2 | 0.3053 | 6.39E-10 |
| SNORD114-1 | MRPS25 | -0.3052 | 6.41E-10 |
| SNORD114-1 | RBM24 | 0.3052 | 6.45E-10 |
| SNORD114-1 | DNM1P35 | -0.3052 | 6.46E-10 |
| SNORD114-1 | TM6SF2 | 0.3051 | 6.49E-10 |
| SNORD114-1 | EVC | 0.305 | 6.60E-10 |
| SNORD114-1 | LOC256880 | -0.305 | 6.61E-10 |
| SNORD114-1 | RIMBP2 | 0.3046 | 6.97E-10 |
| SNORD114-1 | CNTN1 | 0.3046 | 7.02E-10 |
| SNORD114-1 | AMT | -0.3044 | 7.16E-10 |
| SNORD114-1 | EPYC | 0.3043 | 7.24E-10 |
| SNORD114-1 | C19orf29 | -0.3043 | 7.26E-10 |
| SNORD114-1 | GLIS2 | 0.3039 | 7.64E-10 |
| SNORD114-1 | ATP8B2 | 0.3039 | 7.66E-10 |
| SNORD114-1 | NPEPL1 | -0.3038 | 7.83E-10 |
| SNORD114-1 | HSPA12A | 0.3037 | 7.85E-10 |
| SNORD114-1 | LOC100131434 | -0.3037 | 7.90E-10 |
| SNORD114-1 | SFI1 | -0.3036 | 7.97E-10 |
| SNORD114-1 | DPP4 | 0.3035 | 8.09E-10 |
| SNORD114-1 | GUCA1A | 0.3033 | 8.28E-10 |
| SNORD114-1 | MACF1 | 0.3032 | 8.45E-10 |
| SNORD114-1 | LGI2 | 0.3032 | 8.47E-10 |
| SNORD114-1 | INO80B | -0.3031 | 8.59E-10 |
| SNORD114-1 | HTR2A | 0.303 | 8.63E-10 |
| SNORD114-1 | CYBRD1 | 0.3029 | 8.75E-10 |
| SNORD114-1 | PDE5A | 0.3029 | 8.75E-10 |
| SNORD114-1 | OAT | 0.3028 | 8.84E-10 |
| SNORD114-1 | SRRT | -0.3024 | 9.37E-10 |
| SNORD114-1 | CWF19L2 | 0.3023 | 9.48E-10 |
| SNORD114-1 | SCG2 | 0.3022 | 9.64E-10 |
| SNORD114-1 | WDR83 | -0.3022 | 9.69E-10 |
| SNORD114-1 | DSEL | 0.302 | 9.93E-10 |
| SNORD114-1 | FAM198B | 0.3019 | 9.99E-10 |
| SNORD114-1 | SLIT2 | 0.3018 | 1.02E-09 |
| SNORD114-1 | PROS1 | 0.3017 | 1.03E-09 |
| SNORD114-1 | AP1G2 | -0.3016 | 1.04E-09 |
| SNORD114-1 | TCEAL7 | 0.3016 | 1.04E-09 |
| SNORD114-1 | COPZ2 | 0.3011 | 1.12E-09 |
| SNORD114-1 | LOC145474 | 0.301 | 1.13E-09 |
| SNORD114-1 | EFEMP1 | 0.3009 | 1.15E-09 |
| SNORD114-1 | EFCAB4A | -0.3008 | 1.16E-09 |
| SNORD114-1 | OSMR | 0.3008 | 1.16E-09 |
| SNORD114-1 | FMO1 | 0.3006 | 1.19E-09 |
| SNORD114-1 | OGFOD1 | 0.3005 | 1.20E-09 |
| SNORD114-1 | ADAP2 | 0.3003 | 1.25E-09 |
| SNORD114-1 | C15orf17 | -0.3002 | 1.25E-09 |
| SNORD114-1 | RPP21 | -0.3002 | 1.26E-09 |
| U3 | TPM2 | -0.3149 | 1.70E-10 |
| U3 | LY6H | -0.3033 | 8.27E-10 |
| U49A | HSD17B6 | -0.5093 | 2.52E-27 |
| U49A | LOC728264 | -0.4996 | 3.31E-26 |
| U49A | NBLA00301 | -0.4771 | 9.91E-24 |
| U49A | C1orf70 | -0.4771 | 9.92E-24 |
| U49A | CNN1 | -0.477 | 1.01E-23 |
| U49A | GATA5 | -0.4768 | 1.07E-23 |
| U49A | ADRB3 | -0.4717 | 3.63E-23 |
| U49A | CHRDL2 | -0.4664 | 1.26E-22 |
| U49A | LMOD1 | -0.4647 | 1.90E-22 |
| U49A | LIMS2 | -0.464 | 2.21E-22 |
| U49A | DES | -0.4632 | 2.68E-22 |
| U49A | HAND2 | -0.4626 | 3.10E-22 |
| U49A | DACT3 | -0.4624 | 3.23E-22 |
| U49A | PCP4 | -0.4593 | 6.71E-22 |
| U49A | TAGLN | -0.457 | 1.11E-21 |
| U49A | ACTG2 | -0.457 | 1.13E-21 |
| U49A | MRGPRF | -0.4556 | 1.54E-21 |
| U49A | PSD | -0.4556 | 1.55E-21 |
| U49A | SGCA | -0.4533 | 2.59E-21 |
| U49A | PODN | -0.4524 | 3.20E-21 |
| U49A | GSTM5 | -0.4521 | 3.39E-21 |
| U49A | HSPB7 | -0.451 | 4.37E-21 |
| U49A | HSPB6 | -0.4505 | 4.93E-21 |
| U49A | TCEAL2 | -0.4499 | 5.58E-21 |
| U49A | ASB5 | -0.4498 | 5.74E-21 |
| U49A | CSDC2 | -0.4489 | 6.90E-21 |
| U49A | PLA2G5 | -0.4477 | 9.09E-21 |
| U49A | CILP | -0.4474 | 9.77E-21 |
| U49A | MYL9 | -0.4472 | 1.02E-20 |
| U49A | POPDC2 | -0.4471 | 1.04E-20 |
| U49A | PLN | -0.4463 | 1.24E-20 |
| U49A | P2RX1 | -0.4431 | 2.52E-20 |
| U49A | MYH11 | -0.4429 | 2.59E-20 |
| U49A | ITGA7 | -0.4425 | 2.83E-20 |
| U49A | MGP | -0.4422 | 3.04E-20 |
| U49A | SOD3 | -0.4414 | 3.59E-20 |
| U49A | CYP21A2 | -0.4414 | 3.63E-20 |
| U49A | LMCD1 | -0.441 | 3.93E-20 |
| U49A | BOC | -0.4407 | 4.24E-20 |
| U49A | PYGM | -0.4403 | 4.56E-20 |
| U49A | ADAM33 | -0.4402 | 4.64E-20 |
| U49A | ACTA2 | -0.4392 | 5.85E-20 |
| U49A | WISP2 | -0.4383 | 7.07E-20 |
| U49A | C4A | -0.4378 | 7.85E-20 |
| U49A | LEFTY2 | -0.437 | 9.22E-20 |
| U49A | NXPH3 | -0.4364 | 1.06E-19 |
| U49A | FBXL22 | -0.4351 | 1.38E-19 |
| U49A | CLIP3 | -0.4345 | 1.57E-19 |
| U49A | DMPK | -0.4342 | 1.67E-19 |
| U49A | C20orf200 | -0.4342 | 1.70E-19 |
| U49A | KCNMB1 | -0.4337 | 1.86E-19 |
| U49A | FXYD6 | -0.433 | 2.19E-19 |
| U49A | TPM2 | -0.4324 | 2.48E-19 |
| U49A | SCRG1 | -0.4315 | 2.95E-19 |
| U49A | OGN | -0.4306 | 3.57E-19 |
| U49A | MTMR9L | -0.4304 | 3.73E-19 |
| U49A | RARRES2 | -0.4275 | 6.81E-19 |
| U49A | TCEAL6 | -0.4258 | 9.73E-19 |
| U49A | PDZRN3 | -0.4242 | 1.36E-18 |
| U49A | PRDM6 | -0.4237 | 1.50E-18 |
| U49A | EMILIN1 | -0.4236 | 1.52E-18 |
| U49A | MFAP4 | -0.4232 | 1.66E-18 |
| U49A | ITGB1BP2 | -0.4224 | 1.95E-18 |
| U49A | PKIG | -0.4218 | 2.19E-18 |
| U49A | KANK2 | -0.42 | 3.15E-18 |
| U49A | ACTC1 | -0.4185 | 4.29E-18 |
| U49A | DRP2 | -0.4183 | 4.46E-18 |
| U49A | PPAPDC3 | -0.4175 | 5.24E-18 |
| U49A | SELM | -0.4157 | 7.51E-18 |
| U49A | HRNBP3 | -0.4136 | 1.13E-17 |
| U49A | HSPB2 | -0.4135 | 1.16E-17 |
| U49A | HAND1 | -0.4129 | 1.29E-17 |
| U49A | C1orf133 | -0.4112 | 1.81E-17 |
| U49A | FGF7 | -0.41 | 2.30E-17 |
| U49A | SFRP4 | -0.4095 | 2.53E-17 |
| U49A | WBSCR17 | -0.4091 | 2.72E-17 |
| U49A | EIF5B | 0.408 | 3.42E-17 |
| U49A | PTGIS | -0.4078 | 3.52E-17 |
| U49A | SORBS1 | -0.4074 | 3.80E-17 |
| U49A | IFFO1 | -0.4067 | 4.34E-17 |
| U49A | SYNM | -0.4067 | 4.35E-17 |
| U49A | RAMP1 | -0.4062 | 4.82E-17 |
| U49A | MRVI1 | -0.4057 | 5.31E-17 |
| U49A | SMOC2 | -0.4055 | 5.48E-17 |
| U49A | KBTBD10 | -0.4055 | 5.49E-17 |
| U49A | CPXM2 | -0.4054 | 5.56E-17 |
| U49A | ANGPTL1 | -0.4053 | 5.73E-17 |
| U49A | HPSE2 | -0.4048 | 6.31E-17 |
| U49A | HSPA4 | 0.4042 | 7.08E-17 |
| U49A | GPR133 | -0.4042 | 7.09E-17 |
| U49A | PDZD4 | -0.4038 | 7.59E-17 |
| U49A | COX7A1 | -0.4034 | 8.14E-17 |
| U49A | RSPO2 | -0.4029 | 9.01E-17 |
| U49A | SPEG | -0.4029 | 9.07E-17 |
| U49A | CASQ2 | -0.4027 | 9.30E-17 |
| U49A | ADAMTS10 | -0.4023 | 1.01E-16 |
| U49A | PNMAL2 | -0.4021 | 1.05E-16 |
| U49A | SYNPO2 | -0.4016 | 1.16E-16 |
| U49A | PDLIM3 | -0.4013 | 1.23E-16 |
| U49A | RASL12 | -0.4006 | 1.39E-16 |
| U49A | PGM5 | -0.4005 | 1.43E-16 |
| U49A | PRELP | -0.4001 | 1.53E-16 |
| U49A | ZCCHC24 | -0.3996 | 1.69E-16 |
| U49A | ISLR | -0.3987 | 1.99E-16 |
| U49A | LOC401093 | -0.3987 | 1.99E-16 |
| U49A | TACR2 | -0.3984 | 2.10E-16 |
| U49A | C14orf180 | -0.3976 | 2.45E-16 |
| U49A | C6orf225 | -0.3972 | 2.65E-16 |
| U49A | JPH2 | -0.3972 | 2.67E-16 |
| U49A | BHMT2 | -0.3971 | 2.71E-16 |
| U49A | KLF17 | -0.3958 | 3.42E-16 |
| U49A | OMD | -0.3954 | 3.71E-16 |
| U49A | LOC572558 | -0.3946 | 4.26E-16 |
| U49A | TPSAB1 | -0.3946 | 4.27E-16 |
| U49A | VTN | -0.3943 | 4.58E-16 |
| U49A | GRIK5 | -0.3941 | 4.74E-16 |
| U49A | TNS1 | -0.3938 | 5.02E-16 |
| U49A | ELN | -0.3932 | 5.51E-16 |
| U49A | CSRP1 | -0.3932 | 5.56E-16 |
| U49A | TPSB2 | -0.3923 | 6.55E-16 |
| U49A | 2-Mar | -0.3922 | 6.64E-16 |
| U49A | FAM19A5 | -0.3921 | 6.82E-16 |
| U49A | MYOM1 | -0.3916 | 7.43E-16 |
| U49A | PDZRN4 | -0.3902 | 9.59E-16 |
| U49A | HIC1 | -0.3902 | 9.62E-16 |
| U49A | TENC1 | -0.3881 | 1.41E-15 |
| U49A | GYPC | -0.388 | 1.45E-15 |
| U49A | NEGR1 | -0.3876 | 1.55E-15 |
| U49A | NCAM1 | -0.3875 | 1.56E-15 |
| U49A | ZNF775 | -0.3869 | 1.74E-15 |
| U49A | CLEC3A | -0.3866 | 1.85E-15 |
| U49A | LARP1 | 0.3866 | 1.86E-15 |
| U49A | SLC2A4 | -0.3854 | 2.30E-15 |
| U49A | COMP | -0.3848 | 2.57E-15 |
| U49A | LOC399959 | -0.3847 | 2.60E-15 |
| U49A | CACNA1C | -0.3846 | 2.65E-15 |
| U49A | TCEAL3 | -0.3845 | 2.70E-15 |
| U49A | SFRP2 | -0.3838 | 3.08E-15 |
| U49A | GAS6 | -0.3835 | 3.23E-15 |
| U49A | ATP1A2 | -0.3835 | 3.25E-15 |
| U49A | TMOD1 | -0.3834 | 3.29E-15 |
| U49A | LTBP3 | -0.3819 | 4.30E-15 |
| U49A | FLNC | -0.3818 | 4.40E-15 |
| U49A | HSPD1 | 0.3816 | 4.53E-15 |
| U49A | PCBP3 | -0.3815 | 4.63E-15 |
| U49A | IGF1 | -0.3811 | 4.95E-15 |
| U49A | MUSTN1 | -0.3808 | 5.17E-15 |
| U49A | TBX4 | -0.3803 | 5.66E-15 |
| U49A | DPT | -0.3803 | 5.74E-15 |
| U49A | DCN | -0.3799 | 6.12E-15 |
| U49A | HDC | -0.3797 | 6.27E-15 |
| U49A | GATA6 | -0.3797 | 6.38E-15 |
| U49A | TCEAL7 | -0.3795 | 6.51E-15 |
| U49A | OLFML3 | -0.3792 | 6.93E-15 |
| U49A | RASGRP2 | -0.3791 | 6.99E-15 |
| U49A | TBX5 | -0.3787 | 7.52E-15 |
| U49A | CADM3 | -0.3772 | 9.82E-15 |
| U49A | CTSG | -0.3767 | 1.07E-14 |
| U49A | CDO1 | -0.3766 | 1.08E-14 |
| U49A | CYS1 | -0.3765 | 1.10E-14 |
| U49A | ZCCHC5 | -0.3757 | 1.27E-14 |
| U49A | NPAS4 | -0.3755 | 1.33E-14 |
| U49A | PRUNE2 | -0.3754 | 1.34E-14 |
| U49A | PDE1A | -0.3753 | 1.37E-14 |
| U49A | PKDCC | -0.3752 | 1.38E-14 |
| U49A | MRAS | -0.3749 | 1.45E-14 |
| U49A | PPP1R12B | -0.3744 | 1.59E-14 |
| U49A | MEG3 | -0.3742 | 1.65E-14 |
| U49A | MMP23B | -0.374 | 1.70E-14 |
| U49A | PPP1R12C | -0.3738 | 1.76E-14 |
| U49A | C7 | -0.3737 | 1.79E-14 |
| U49A | ARHGAP20 | -0.3737 | 1.80E-14 |
| U49A | RGN | -0.3734 | 1.90E-14 |
| U49A | LOC100126784 | -0.3732 | 1.97E-14 |
| U49A | CRYGN | -0.3731 | 2.01E-14 |
| U49A | NFATC4 | -0.373 | 2.04E-14 |
| U49A | PLEKHO1 | -0.3728 | 2.11E-14 |
| U49A | NAP1L3 | -0.3726 | 2.16E-14 |
| U49A | ADCY2 | -0.3721 | 2.39E-14 |
| U49A | CHRD | -0.372 | 2.42E-14 |
| U49A | LMO3 | -0.3717 | 2.52E-14 |
| U49A | ITGA10 | -0.3714 | 2.69E-14 |
| U49A | XPNPEP2 | -0.3712 | 2.76E-14 |
| U49A | AOC3 | -0.3712 | 2.79E-14 |
| U49A | ANGPTL7 | -0.3706 | 3.09E-14 |
| U49A | FIBIN | -0.3705 | 3.13E-14 |
| U49A | SCT | -0.37 | 3.37E-14 |
| U49A | DMGDH | -0.3696 | 3.66E-14 |
| U49A | MXRA8 | -0.3694 | 3.75E-14 |
| U49A | MFRP | -0.368 | 4.79E-14 |
| U49A | TCF21 | -0.3679 | 4.86E-14 |
| U49A | PTH1R | -0.3678 | 4.93E-14 |
| U49A | GPRASP1 | -0.3677 | 5.03E-14 |
| U49A | LTC4S | -0.3675 | 5.21E-14 |
| U49A | C2orf40 | -0.3672 | 5.42E-14 |
| U49A | BNC2 | -0.3671 | 5.56E-14 |
| U49A | DIO3 | -0.3671 | 5.59E-14 |
| U49A | PPP1R14A | -0.3665 | 6.11E-14 |
| U49A | RSPO1 | -0.3658 | 6.89E-14 |
| U49A | HEPH | -0.3649 | 7.99E-14 |
| U49A | KIAA1755 | -0.3646 | 8.44E-14 |
| U49A | TGFB1I1 | -0.3644 | 8.68E-14 |
| U49A | CNPY4 | -0.3644 | 8.75E-14 |
| U49A | CACNA1H | -0.364 | 9.32E-14 |
| U49A | MEOX2 | -0.3636 | 1.00E-13 |
| U49A | GADD45B | -0.3633 | 1.04E-13 |
| U49A | LRRN4CL | -0.3634 | 1.04E-13 |
| U49A | COLEC12 | -0.3633 | 1.06E-13 |
| U49A | VENTX | -0.3631 | 1.09E-13 |
| U49A | RCAN2 | -0.3629 | 1.12E-13 |
| U49A | CDK7 | 0.3626 | 1.17E-13 |
| U49A | CHRDL1 | -0.3627 | 1.17E-13 |
| U49A | USHBP1 | -0.3625 | 1.20E-13 |
| U49A | TCEAL5 | -0.3624 | 1.22E-13 |
| U49A | COL16A1 | -0.3622 | 1.26E-13 |
| U49A | KLHL38 | -0.3622 | 1.26E-13 |
| U49A | LY6H | -0.3622 | 1.26E-13 |
| U49A | SSC5D | -0.3618 | 1.34E-13 |
| U49A | SNED1 | -0.3618 | 1.35E-13 |
| U49A | PLA2G2A | -0.3617 | 1.36E-13 |
| U49A | GEFT | -0.3614 | 1.43E-13 |
| U49A | JAM3 | -0.3614 | 1.43E-13 |
| U49A | PLEKHA4 | -0.3612 | 1.47E-13 |
| U49A | ANKRD53 | -0.3612 | 1.49E-13 |
| U49A | HSPA12B | -0.361 | 1.52E-13 |
| U49A | AEBP1 | -0.361 | 1.54E-13 |
| U49A | SGCD | -0.3605 | 1.67E-13 |
| U49A | AGTR1 | -0.3603 | 1.72E-13 |
| U49A | CCL21 | -0.3602 | 1.75E-13 |
| U49A | ASB2 | -0.36 | 1.80E-13 |
| U49A | ANXA6 | -0.3598 | 1.86E-13 |
| U49A | ANXA13 | -0.3597 | 1.91E-13 |
| U49A | CYP27A1 | -0.3595 | 1.97E-13 |
| U49A | CMA1 | -0.3592 | 2.06E-13 |
| U49A | PI16 | -0.359 | 2.12E-13 |
| U49A | LOC283731 | -0.3586 | 2.26E-13 |
| U49A | SCARF2 | -0.3576 | 2.66E-13 |
| U49A | MYOZ2 | -0.3574 | 2.77E-13 |
| U49A | CCAR1 | 0.3574 | 2.78E-13 |
| U49A | CC2D2B | -0.3573 | 2.80E-13 |
| U49A | PPIAL4C | 0.3571 | 2.90E-13 |
| U49A | SERPINF1 | -0.357 | 2.93E-13 |
| U49A | RBPMS2 | -0.3569 | 3.02E-13 |
| U49A | CLEC11A | -0.356 | 3.50E-13 |
| U49A | GPIHBP1 | -0.3558 | 3.61E-13 |
| U49A | ANGPTL2 | -0.3557 | 3.63E-13 |
| U49A | FBXL7 | -0.3555 | 3.74E-13 |
| U49A | CAMK2A | -0.3545 | 4.40E-13 |
| U49A | GPR88 | -0.3545 | 4.45E-13 |
| U49A | EIF5AL1 | 0.3544 | 4.50E-13 |
| U49A | KCNA6 | -0.3542 | 4.62E-13 |
| U49A | KCNK3 | -0.3539 | 4.88E-13 |
| U49A | RNF112 | -0.3537 | 5.07E-13 |
| U49A | NRXN2 | -0.3536 | 5.08E-13 |
| U49A | MMP2 | -0.3536 | 5.15E-13 |
| U49A | GAS1 | -0.3531 | 5.55E-13 |
| U49A | MFGE8 | -0.353 | 5.62E-13 |
| U49A | KLF2 | -0.3529 | 5.75E-13 |
| U49A | HPD | -0.3527 | 5.89E-13 |
| U49A | A2M | -0.3525 | 6.08E-13 |
| U49A | FHL1 | -0.3523 | 6.30E-13 |
| U49A | CNRIP1 | -0.3523 | 6.31E-13 |
| U49A | FXYD1 | -0.3522 | 6.44E-13 |
| U49A | IGFBP7 | -0.3521 | 6.49E-13 |
| U49A | THBS4 | -0.3516 | 7.06E-13 |
| U49A | WDR86 | -0.351 | 7.70E-13 |
| U49A | CPXM1 | -0.3508 | 8.04E-13 |
| U49A | TMEM149 | -0.3508 | 8.07E-13 |
| U49A | SPARCL1 | -0.3507 | 8.19E-13 |
| U49A | CA3 | -0.3505 | 8.46E-13 |
| U49A | ZNF521 | -0.3497 | 9.48E-13 |
| U49A | STAT5A | -0.3497 | 9.57E-13 |
| U49A | HIF3A | -0.3496 | 9.68E-13 |
| U49A | MAP3K3 | -0.3495 | 9.86E-13 |
| U49A | TMEM35 | -0.3491 | 1.05E-12 |
| U49A | ATP8B4 | -0.349 | 1.06E-12 |
| U49A | FLJ42875 | -0.3487 | 1.12E-12 |
| U49A | DLK1 | -0.3485 | 1.15E-12 |
| U49A | GPR124 | -0.3481 | 1.23E-12 |
| U49A | CCL19 | -0.3479 | 1.27E-12 |
| U49A | ROM1 | -0.3479 | 1.27E-12 |
| U49A | LDB3 | -0.3477 | 1.30E-12 |
| U49A | LOXL1 | -0.3477 | 1.31E-12 |
| U49A | CXCL12 | -0.3477 | 1.32E-12 |
| U49A | RSPO3 | -0.3477 | 1.32E-12 |
| U49A | ADAMTS8 | -0.3476 | 1.33E-12 |
| U49A | JAM2 | -0.3476 | 1.33E-12 |
| U49A | CACNB4 | -0.3474 | 1.37E-12 |
| U49A | SCN1B | -0.3473 | 1.40E-12 |
| U49A | ADH1B | -0.3472 | 1.41E-12 |
| U49A | NTNG2 | -0.3469 | 1.47E-12 |
| U49A | KCNE4 | -0.3463 | 1.62E-12 |
| U49A | LOC283867 | -0.3463 | 1.63E-12 |
| U49A | TNFSF12 | -0.346 | 1.71E-12 |
| U49A | LYL1 | -0.3459 | 1.72E-12 |
| U49A | TMEM100 | -0.3458 | 1.77E-12 |
| U49A | TSC22D3 | -0.3458 | 1.77E-12 |
| U49A | DUSP26 | -0.345 | 2.00E-12 |
| U49A | NPM1 | 0.3443 | 2.23E-12 |
| U49A | CCDC80 | -0.3442 | 2.25E-12 |
| U49A | DGKB | -0.344 | 2.33E-12 |
| U49A | STAB2 | -0.344 | 2.33E-12 |
| U49A | CPA3 | -0.3439 | 2.38E-12 |
| U49A | ABCB4 | -0.3438 | 2.41E-12 |
| U49A | EID2B | -0.3438 | 2.41E-12 |
| U49A | TCERG1 | 0.3436 | 2.49E-12 |
| U49A | CACNB2 | -0.3435 | 2.53E-12 |
| U49A | TPSG1 | -0.3432 | 2.64E-12 |
| U49A | RCVRN | -0.3432 | 2.66E-12 |
| U49A | BAZ1A | 0.3429 | 2.75E-12 |
| U49A | TIMP2 | -0.3424 | 2.97E-12 |
| U49A | MSRB3 | -0.3423 | 3.04E-12 |
| U49A | IL16 | -0.3422 | 3.07E-12 |
| U49A | G2E3 | 0.3422 | 3.10E-12 |
| U49A | PRPH | -0.3422 | 3.10E-12 |
| U49A | KCNJ8 | -0.342 | 3.17E-12 |
| U49A | NBL1 | -0.3419 | 3.21E-12 |
| U49A | ARID5A | -0.3418 | 3.30E-12 |
| U49A | LTF | -0.3414 | 3.49E-12 |
| U49A | CTSK | -0.3413 | 3.53E-12 |
| U49A | GGT5 | -0.3412 | 3.57E-12 |
| U49A | PTGER3 | -0.341 | 3.72E-12 |
| U49A | NOLC1 | 0.3408 | 3.84E-12 |
| U49A | MYLK | -0.3407 | 3.90E-12 |
| U49A | PRR24 | -0.3406 | 3.93E-12 |
| U49A | CRYAB | -0.3405 | 3.99E-12 |
| U49A | RANBP3L | -0.3403 | 4.11E-12 |
| U49A | CLDN5 | -0.3402 | 4.19E-12 |
| U49A | MAGEH1 | -0.34 | 4.34E-12 |
| U49A | C16orf86 | -0.34 | 4.35E-12 |
| U49A | DHH | -0.3399 | 4.37E-12 |
| U49A | ABI3BP | -0.3399 | 4.38E-12 |
| U49A | SOBP | -0.3399 | 4.39E-12 |
| U49A | COL6A2 | -0.3398 | 4.47E-12 |
| U49A | ZNF575 | -0.3397 | 4.52E-12 |
| U49A | PTGS1 | -0.3395 | 4.67E-12 |
| U49A | FBLN2 | -0.3395 | 4.69E-12 |
| U49A | GDF5 | -0.3394 | 4.74E-12 |
| U49A | C8orf84 | -0.3393 | 4.82E-12 |
| U49A | SPON1 | -0.339 | 5.02E-12 |
| U49A | KIAA1644 | -0.3389 | 5.10E-12 |
| U49A | VIPR2 | -0.3385 | 5.41E-12 |
| U49A | EFEMP2 | -0.3382 | 5.69E-12 |
| U49A | ABCB5 | -0.3382 | 5.70E-12 |
| U49A | RGS22 | -0.3378 | 6.04E-12 |
| U49A | LOC441204 | -0.3378 | 6.07E-12 |
| U49A | SHC2 | -0.3375 | 6.34E-12 |
| U49A | DIO3OS | -0.3371 | 6.70E-12 |
| U49A | ADAMTS16 | -0.3368 | 7.03E-12 |
| U49A | GJC2 | -0.3366 | 7.21E-12 |
| U49A | KERA | -0.3364 | 7.43E-12 |
| U49A | GLIS2 | -0.3363 | 7.63E-12 |
| U49A | NAALADL1 | -0.3359 | 8.01E-12 |
| U49A | FGFR1 | -0.3357 | 8.31E-12 |
| U49A | GNAO1 | -0.3354 | 8.67E-12 |
| U49A | FAM20C | -0.3354 | 8.68E-12 |
| U49A | ENTPD1 | -0.3354 | 8.69E-12 |
| U49A | CKMT2 | -0.3353 | 8.82E-12 |
| U49A | NEXN | -0.3352 | 8.89E-12 |
| U49A | EGLN2 | -0.3352 | 9.02E-12 |
| U49A | PBXIP1 | -0.3351 | 9.13E-12 |
| U49A | C15orf59 | -0.335 | 9.18E-12 |
| U49A | FBXO32 | -0.335 | 9.18E-12 |
| U49A | IL17B | -0.3349 | 9.36E-12 |
| U49A | WDR36 | 0.3349 | 9.42E-12 |
| U49A | P4HA3 | -0.3348 | 9.57E-12 |
| U49A | DPYSL3 | -0.3347 | 9.71E-12 |
| U49A | CRISPLD2 | -0.3346 | 9.74E-12 |
| U49A | SIGLECP3 | -0.3346 | 9.74E-12 |
| U49A | KCNMA1 | -0.3345 | 1.00E-11 |
| U49A | C1QBP | 0.3343 | 1.02E-11 |
| U49A | C1QTNF4 | -0.3344 | 1.02E-11 |
| U49A | NOP58 | 0.3343 | 1.02E-11 |
| U49A | SLC1A7 | -0.3341 | 1.06E-11 |
| U49A | C1R | -0.3339 | 1.09E-11 |
| U49A | FBLN5 | -0.3338 | 1.10E-11 |
| U49A | FCN1 | -0.3336 | 1.14E-11 |
| U49A | SMPD1 | -0.3334 | 1.18E-11 |
| U49A | ADCY5 | -0.3333 | 1.19E-11 |
| U49A | RAB23 | -0.3333 | 1.19E-11 |
| U49A | MYH3 | -0.333 | 1.24E-11 |
| U49A | TSPAN2 | -0.333 | 1.24E-11 |
| U49A | HRASLS5 | -0.3329 | 1.27E-11 |
| U49A | TPM1 | -0.3329 | 1.27E-11 |
| U49A | DACT1 | -0.3328 | 1.29E-11 |
| U49A | PTGDS | -0.3327 | 1.30E-11 |
| U49A | KANK3 | -0.3325 | 1.34E-11 |
| U49A | ANTXR2 | -0.3322 | 1.41E-11 |
| U49A | C3orf18 | -0.3321 | 1.42E-11 |
| U49A | TNXB | -0.3319 | 1.47E-11 |
| U49A | ZFPM2 | -0.3317 | 1.51E-11 |
| U49A | KCNIP1 | -0.3312 | 1.62E-11 |
| U49A | SPTBN4 | -0.3313 | 1.62E-11 |
| U49A | HLF | -0.3311 | 1.66E-11 |
| U49A | DARS | 0.3306 | 1.77E-11 |
| U49A | TMEM119 | -0.3305 | 1.80E-11 |
| U49A | PHYHIP | -0.3304 | 1.83E-11 |
| U49A | ITIH3 | -0.3304 | 1.84E-11 |
| U49A | FAIM2 | -0.3303 | 1.85E-11 |
| U49A | C10orf10 | -0.3303 | 1.86E-11 |
| U49A | DHX33 | 0.3301 | 1.92E-11 |
| U49A | ABHD8 | -0.3299 | 1.97E-11 |
| U49A | REEP1 | -0.3298 | 2.00E-11 |
| U49A | SPON2 | -0.3296 | 2.06E-11 |
| U49A | SYNE1 | -0.3294 | 2.13E-11 |
| U49A | ADIPOQ | -0.3292 | 2.19E-11 |
| U49A | PHKG1 | -0.3292 | 2.20E-11 |
| U49A | PLEKHO2 | -0.3291 | 2.22E-11 |
| U49A | SOX10 | -0.3291 | 2.22E-11 |
| U49A | DPEP1 | -0.3289 | 2.27E-11 |
| U49A | PLAC9 | -0.3289 | 2.28E-11 |
| U49A | TMEM229A | -0.3288 | 2.33E-11 |
| U49A | ABCE1 | 0.3286 | 2.37E-11 |
| U49A | GPR26 | -0.3286 | 2.40E-11 |
| U49A | GPR17 | -0.3285 | 2.42E-11 |
| U49A | MYBBP1A | 0.3284 | 2.45E-11 |
| U49A | TSPAN4 | -0.3283 | 2.48E-11 |
| U49A | FAM180A | -0.3283 | 2.51E-11 |
| U49A | NPY6R | -0.3283 | 2.51E-11 |
| U49A | FNDC1 | -0.3282 | 2.55E-11 |
| U49A | COL8A2 | -0.3281 | 2.56E-11 |
| U49A | CHRM2 | -0.3279 | 2.66E-11 |
| U49A | C5orf4 | -0.3278 | 2.68E-11 |
| U49A | SH3BGR | -0.3278 | 2.70E-11 |
| U49A | GNG7 | -0.3277 | 2.74E-11 |
| U49A | RNASE4 | -0.3275 | 2.82E-11 |
| U49A | ADORA1 | -0.3274 | 2.87E-11 |
| U49A | ST6GALNAC5 | -0.3272 | 2.92E-11 |
| U49A | ILK | -0.3272 | 2.94E-11 |
| U49A | C13orf18 | -0.3268 | 3.13E-11 |
| U49A | CRYM | -0.3264 | 3.28E-11 |
| U49A | TXLNB | -0.3263 | 3.34E-11 |
| U49A | C20orf103 | -0.3261 | 3.44E-11 |
| U49A | C8orf85 | -0.326 | 3.48E-11 |
| U49A | SFTPC | 0.3259 | 3.52E-11 |
| U49A | GALNTL1 | -0.3259 | 3.57E-11 |
| U49A | LOC144571 | -0.3257 | 3.64E-11 |
| U49A | ANKRD35 | -0.3255 | 3.76E-11 |
| U49A | CCL14 | -0.3254 | 3.83E-11 |
| U49A | CYR61 | -0.3253 | 3.88E-11 |
| U49A | F10 | -0.3252 | 3.96E-11 |
| U49A | HPGDS | -0.325 | 4.05E-11 |
| U49A | PDCD11 | 0.3249 | 4.11E-11 |
| U49A | PENK | -0.3249 | 4.13E-11 |
| U49A | CCDC149 | -0.3248 | 4.14E-11 |
| U49A | C10orf72 | -0.3247 | 4.20E-11 |
| U49A | FKBP7 | -0.3247 | 4.23E-11 |
| U49A | TCOF1 | 0.3247 | 4.24E-11 |
| U49A | TMEM130 | -0.3247 | 4.25E-11 |
| U49A | LRRC2 | -0.3246 | 4.31E-11 |
| U49A | CNTNAP1 | -0.3243 | 4.49E-11 |
| U49A | GAS7 | -0.324 | 4.67E-11 |
| U49A | NNMT | -0.3235 | 4.99E-11 |
| U49A | GNAZ | -0.3235 | 5.04E-11 |
| U49A | PABPC5 | -0.3235 | 5.05E-11 |
| U49A | PCOLCE | -0.3234 | 5.09E-11 |
| U49A | SRL | -0.3234 | 5.10E-11 |
| U49A | FAM65C | -0.3233 | 5.15E-11 |
| U49A | PIK3R6 | -0.3232 | 5.25E-11 |
| U49A | HAAO | -0.3231 | 5.35E-11 |
| U49A | POLR1B | 0.3231 | 5.35E-11 |
| U49A | INMT | -0.3229 | 5.48E-11 |
| U49A | EIF3E | 0.3225 | 5.80E-11 |
| U49A | DIRAS3 | -0.3224 | 5.86E-11 |
| U49A | ADCY4 | -0.3223 | 5.98E-11 |
| U49A | LRRC17 | -0.3223 | 5.99E-11 |
| U49A | NHLRC4 | -0.3222 | 6.02E-11 |
| U49A | VASN | -0.3221 | 6.11E-11 |
| U49A | NDN | -0.3221 | 6.16E-11 |
| U49A | MYL6 | -0.322 | 6.20E-11 |
| U49A | YWHAE | 0.3217 | 6.47E-11 |
| U49A | DCHS2 | -0.3215 | 6.72E-11 |
| U49A | AGPAT5 | 0.3214 | 6.76E-11 |
| U49A | MYOCD | -0.3212 | 6.96E-11 |
| U49A | ADRA2A | -0.3211 | 7.11E-11 |
| U49A | C10orf2 | 0.3211 | 7.11E-11 |
| U49A | EIF5A | 0.3211 | 7.12E-11 |
| U49A | PTGFR | -0.3207 | 7.46E-11 |
| U49A | PRICKLE2 | -0.3207 | 7.50E-11 |
| U49A | HTRA3 | -0.3206 | 7.67E-11 |
| U49A | ADC | -0.3205 | 7.68E-11 |
| U49A | FAM69C | -0.3205 | 7.71E-11 |
| U49A | THRA | -0.3205 | 7.74E-11 |
| U49A | ADARB1 | -0.32 | 8.34E-11 |
| U49A | KIAA1614 | -0.3198 | 8.51E-11 |
| U49A | LY96 | -0.3187 | 9.91E-11 |
| U49A | HOPX | -0.3187 | 1.00E-10 |
| U49A | DOK5 | -0.3185 | 1.02E-10 |
| U49A | HTRA1 | -0.3184 | 1.04E-10 |
| U49A | AXIN2 | -0.3182 | 1.07E-10 |
| U49A | ASPN | -0.3181 | 1.08E-10 |
| U49A | GAPT | -0.3179 | 1.11E-10 |
| U49A | ACACB | -0.3178 | 1.13E-10 |
| U49A | DAPK3 | -0.3177 | 1.15E-10 |
| U49A | KCNH2 | -0.3177 | 1.15E-10 |
| U49A | LOC100130872 | -0.3171 | 1.25E-10 |
| U49A | CTGF | -0.3169 | 1.29E-10 |
| U49A | EPM2A | -0.3168 | 1.31E-10 |
| U49A | PDLIM7 | -0.3168 | 1.31E-10 |
| U49A | TNFSF4 | -0.3165 | 1.37E-10 |
| U49A | ITGBL1 | -0.3163 | 1.39E-10 |
| U49A | DCLK2 | -0.316 | 1.45E-10 |
| U49A | TPO | -0.316 | 1.46E-10 |
| U49A | ADCYAP1 | -0.3159 | 1.47E-10 |
| U49A | CCDC34 | 0.3158 | 1.51E-10 |
| U49A | MAGEL2 | -0.3157 | 1.53E-10 |
| U49A | CCR10 | -0.3155 | 1.56E-10 |
| U49A | GPR162 | -0.3155 | 1.57E-10 |
| U49A | FMOD | -0.3154 | 1.58E-10 |
| U49A | RCN3 | -0.3154 | 1.59E-10 |
| U49A | C1QTNF2 | -0.3153 | 1.61E-10 |
| U49A | SYPL2 | -0.3152 | 1.62E-10 |
| U49A | RENBP | -0.3149 | 1.69E-10 |
| U49A | CLEC3B | -0.3148 | 1.72E-10 |
| U49A | LOC100128239 | -0.3147 | 1.74E-10 |
| U49A | SYT11 | -0.3147 | 1.75E-10 |
| U49A | LYRM7 | 0.3144 | 1.82E-10 |
| U49A | DCHS1 | -0.3141 | 1.90E-10 |
| U49A | LOC644538 | -0.3141 | 1.90E-10 |
| U49A | STMN4 | -0.3141 | 1.91E-10 |
| U49A | RYR2 | -0.3139 | 1.95E-10 |
| U49A | C1QTNF1 | -0.3137 | 2.01E-10 |
| U49A | CYBRD1 | -0.3137 | 2.01E-10 |
| U49A | AOX1 | -0.3135 | 2.07E-10 |
| U49A | COPZ2 | -0.3133 | 2.14E-10 |
| U49A | CPEB1 | -0.3132 | 2.15E-10 |
| U49A | PRPH2 | -0.3131 | 2.20E-10 |
| U49A | THAP8 | -0.313 | 2.22E-10 |
| U49A | SCN2B | -0.3129 | 2.24E-10 |
| U49A | PNMAL1 | -0.3128 | 2.28E-10 |
| U49A | PPFIA2 | -0.3128 | 2.28E-10 |
| U49A | FAM129C | -0.3125 | 2.39E-10 |
| U49A | SH3BGRL | -0.3124 | 2.42E-10 |
| U49A | SLC24A3 | -0.3123 | 2.43E-10 |
| U49A | RDH5 | -0.3123 | 2.44E-10 |
| U49A | MITF | -0.3121 | 2.53E-10 |
| U49A | LRRC32 | -0.312 | 2.57E-10 |
| U49A | CDH11 | -0.3119 | 2.58E-10 |
| U49A | CCDC81 | -0.3118 | 2.62E-10 |
| U49A | SORCS1 | -0.3118 | 2.63E-10 |
| U49A | EXTL1 | -0.3118 | 2.64E-10 |
| U49A | F7 | -0.3116 | 2.69E-10 |
| U49A | PLXNA4 | -0.3115 | 2.74E-10 |
| U49A | ROR2 | -0.3115 | 2.74E-10 |
| U49A | PDK2 | -0.3114 | 2.75E-10 |
| U49A | UNC5A | -0.3115 | 2.75E-10 |
| U49A | LOC283174 | -0.3113 | 2.81E-10 |
| U49A | CTSF | -0.3111 | 2.90E-10 |
| U49A | PDE1B | -0.3109 | 2.96E-10 |
| U49A | GEM | -0.3108 | 3.00E-10 |
| U49A | KCNA1 | -0.3108 | 3.03E-10 |
| U49A | VIT | -0.3107 | 3.05E-10 |
| U49A | C17orf60 | -0.3106 | 3.10E-10 |
| U49A | COMMD6 | -0.3105 | 3.12E-10 |
| U49A | LOC100192378 | -0.3102 | 3.28E-10 |
| U49A | C14orf73 | -0.31 | 3.38E-10 |
| U49A | TGFB3 | -0.3099 | 3.42E-10 |
| U49A | ISLR2 | -0.3098 | 3.44E-10 |
| U49A | SCAMP1 | 0.3098 | 3.47E-10 |
| U49A | PRDM8 | -0.3096 | 3.56E-10 |
| U49A | ENPP3 | -0.3094 | 3.63E-10 |
| U49A | POU2F1 | 0.3093 | 3.67E-10 |
| U49A | ZDHHC15 | -0.3092 | 3.73E-10 |
| U49A | CNGA3 | -0.3092 | 3.76E-10 |
| U49A | CALD1 | -0.3091 | 3.80E-10 |
| U49A | LEPREL2 | -0.3091 | 3.82E-10 |
| U49A | SLC26A10 | -0.3089 | 3.89E-10 |
| U49A | MS4A2 | -0.3089 | 3.90E-10 |
| U49A | SPOP | -0.3089 | 3.92E-10 |
| U49A | MAP1A | -0.3088 | 3.96E-10 |
| U49A | SLMO2 | 0.3087 | 3.99E-10 |
| U49A | SPINK2 | -0.3087 | 4.03E-10 |
| U49A | PRND | -0.3086 | 4.07E-10 |
| U49A | SYDE1 | -0.3086 | 4.07E-10 |
| U49A | NCOR1 | 0.3085 | 4.15E-10 |
| U49A | GFM2 | 0.3084 | 4.19E-10 |
| U49A | C11orf68 | -0.3082 | 4.29E-10 |
| U49A | WDR43 | 0.3079 | 4.47E-10 |
| U49A | WNT9B | -0.3077 | 4.63E-10 |
| U49A | KIAA2022 | -0.3076 | 4.64E-10 |
| U49A | PALLD | -0.3076 | 4.69E-10 |
| U49A | DDIT4L | -0.307 | 5.04E-10 |
| U49A | CYP46A1 | -0.3069 | 5.09E-10 |
| U49A | CUEDC1 | -0.3068 | 5.19E-10 |
| U49A | CBX7 | -0.3068 | 5.20E-10 |
| U49A | FXYD2 | -0.3068 | 5.21E-10 |
| U49A | CD22 | -0.3065 | 5.40E-10 |
| U49A | PRSS36 | -0.3065 | 5.43E-10 |
| U49A | GALNTL2 | -0.3065 | 5.44E-10 |
| U49A | DARC | -0.3064 | 5.46E-10 |
| U49A | PTPN5 | -0.3063 | 5.54E-10 |
| U49A | SMTN | -0.3062 | 5.60E-10 |
| U49A | RD3 | -0.3062 | 5.61E-10 |
| U49A | FGF10 | -0.3062 | 5.65E-10 |
| U49A | STK32B | -0.3061 | 5.70E-10 |
| U49A | LDLRAD2 | -0.3053 | 6.37E-10 |
| U49A | ANGPTL6 | -0.3052 | 6.42E-10 |
| U49A | SSPN | -0.3051 | 6.52E-10 |
| U49A | C21orf122 | -0.3049 | 6.71E-10 |
| U49A | MYOC | -0.3048 | 6.77E-10 |
| U49A | OSBPL5 | -0.3048 | 6.82E-10 |
| U49A | CCL11 | -0.3047 | 6.85E-10 |
| U49A | TFAM | 0.3044 | 7.18E-10 |
| U49A | STAB1 | -0.3044 | 7.20E-10 |
| U49A | LOH3CR2A | -0.3043 | 7.30E-10 |
| U49A | TMEM204 | -0.3042 | 7.40E-10 |
| U49A | CCDC154 | -0.3041 | 7.51E-10 |
| U49A | OLFML1 | -0.304 | 7.56E-10 |
| U49A | POU6F1 | -0.3039 | 7.63E-10 |
| U49A | GIMAP1 | -0.3038 | 7.79E-10 |
| U49A | SLITRK3 | -0.3036 | 8.04E-10 |
| U49A | CCDC107 | -0.3035 | 8.11E-10 |
| U49A | CD99 | -0.3034 | 8.26E-10 |
| U49A | ZNF428 | -0.3031 | 8.49E-10 |
| U49A | PEG3 | -0.3031 | 8.60E-10 |
| U49A | LAMB2 | -0.303 | 8.62E-10 |
| U49A | AHCTF1 | 0.3029 | 8.81E-10 |
| U49A | NUP50 | 0.3028 | 8.84E-10 |
| U49A | C20orf166 | -0.3028 | 8.92E-10 |
| U49A | CMAH | -0.3027 | 8.96E-10 |
| U49A | PHF1 | -0.3027 | 8.96E-10 |
| U49A | CX3CL1 | -0.3027 | 9.00E-10 |
| U49A | ANTXR1 | -0.3027 | 9.05E-10 |
| U49A | MEIS3 | -0.3026 | 9.16E-10 |
| U49A | TMEM50B | -0.3025 | 9.26E-10 |
| U49A | HHIPL1 | -0.3024 | 9.33E-10 |
| U49A | PRG4 | -0.3022 | 9.59E-10 |
| U49A | HPR | -0.3022 | 9.61E-10 |
| U49A | ERVFRDE1 | -0.3021 | 9.74E-10 |
| U49A | MXRA7 | -0.302 | 9.91E-10 |
| U49A | AKAP6 | -0.3019 | 1.01E-09 |
| U49A | CCDC74A | -0.3018 | 1.01E-09 |
| U49A | KIRREL3 | -0.3018 | 1.02E-09 |
| U49A | GJA5 | -0.3017 | 1.03E-09 |
| U49A | C13orf33 | -0.3016 | 1.04E-09 |
| U49A | ZEB1 | -0.3016 | 1.04E-09 |
| U49A | LRFN5 | -0.3016 | 1.05E-09 |
| U49A | OXER1 | -0.3014 | 1.07E-09 |
| U49A | C1QTNF7 | -0.3013 | 1.08E-09 |
| U49A | MAP6 | -0.3012 | 1.10E-09 |
| U49A | NUCB1 | -0.3012 | 1.11E-09 |
| U49A | NUDT10 | -0.301 | 1.13E-09 |
| U49A | RAI2 | -0.301 | 1.13E-09 |
| U49A | ZBTB16 | -0.3009 | 1.14E-09 |
| U49A | LCN10 | -0.3008 | 1.15E-09 |
| U49A | ABCA9 | -0.3008 | 1.16E-09 |
| U49A | SFRP1 | -0.3008 | 1.16E-09 |
| U49A | ACOX2 | -0.3007 | 1.17E-09 |
| U49A | FGF16 | -0.3005 | 1.20E-09 |
| U49A | F13A1 | -0.3004 | 1.23E-09 |
| U49A | HUNK | -0.3004 | 1.23E-09 |
| U49A | LOC283663 | -0.3003 | 1.23E-09 |
| U49A | TSPAN18 | -0.3004 | 1.23E-09 |
| U49A | GAB3 | -0.3003 | 1.24E-09 |
| U49A | RBM24 | -0.3003 | 1.24E-09 |
