# Supplementary material for: Small nucleolar RNAs signature (SNORS) identified clinical outcome and prognosis of bladder cancer (BLCA)
Source: Cancer Cell Int. 2020 Jul 10;20:299. doi: 10.1186/s12935-020-01393-7 (PMC7350589; doi:10.1186/s12935-020-01393-7)
Supplement: Supplementary file 11 — Additional file 11: Table S9. The input for correlation network of candidate snoRNAs associated mRNAs in TCGA-BLCA cohort. [file 12935_2020_1393_MOESM11_ESM.docx]

**Additional file 11: Table S9 The input for correlation network of candidate snoRNAs associated mRNAs in TCGA-BLCA cohort (n = 392)**

| id | Gene Symbol | Coefficient | P-value | cor | HR | Table | HR.95L | HR.95H | pvalue |
| --- | --- | --- | --- | --- | --- | --- | --- | --- | --- |
| SNORD113-9 | COL1A1 | 0.4226 | 1.87E-18 | 1 | 1.000069718 | -1 | 1.000015973 | 1.000123466 | 0.011007254 |
| SNORD113-9 | COL3A1 | 0.4106 | 2.05E-17 | 1 | 1.000078169 | -1 | 1.000011086 | 1.000145256 | 0.022378028 |
| SNORD113-9 | MMP9 | 0.3081 | 4.34E-10 | 1 | 1.000079629 | -1 | 1.000021742 | 1.000137519 | 0.007014473 |
| SNORD113-9 | COL1A2 | 0.4144 | 9.62E-18 | 1 | 1.000148497 | -1 | 1.000038427 | 1.00025858 | 0.00818701 |
| SNORD113-9 | SPARC | 0.387 | 1.72E-15 | 1 | 1.000175584 | -1 | 1.000047509 | 1.000303676 | 0.007208304 |
| SNORD113-9 | BGN | 0.3517 | 6.94E-13 | 1 | 1.000220423 | -1 | 1.000040002 | 1.000400876 | 0.016639878 |
| SNORD113-9 | VIM | 0.3452 | 1.94E-12 | 1 | 1.000274 | -1 | 1.000081691 | 1.000466346 | 0.005227817 |
| SNORD113-9 | COL6A1 | 0.4175 | 5.21E-18 | 1 | 1.000284727 | -1 | 1.000055672 | 1.000513833 | 0.014833984 |
| SNORD113-9 | LUM | 0.3651 | 7.70E-14 | 1 | 1.000341435 | -1 | 1.000064347 | 1.0006186 | 0.015726709 |
| SNORD113-9 | CTSK | 0.4126 | 1.38E-17 | 1 | 1.000347461 | -1 | 1.000102254 | 1.000592729 | 0.005479058 |
| SNORD113-9 | FN1 | 0.3205 | 7.77E-11 | 1 | 1.000398967 | -1 | 1.000214583 | 1.000583385 | 2.22E-05 |
| SNORD113-9 | COMP | 0.3249 | 4.09E-11 | 1 | 1.000404967 | -1 | 1.00001741 | 1.000792674 | 0.04055705 |
| SNORD113-9 | CTGF | 0.369 | 4.03E-14 | 1 | 1.000535855 | -1 | 1.000004875 | 1.001067117 | 0.047932992 |
| SNORD113-9 | AEBP1 | 0.3778 | 8.90E-15 | 1 | 1.000594119 | -1 | 1.000119758 | 1.001068705 | 0.014091262 |
| SNORD113-9 | TGFBI | 0.3153 | 1.62E-10 | 1 | 1.000611752 | -1 | 1.000147722 | 1.001075997 | 0.009763254 |
| SNORD113-9 | ISLR | 0.3933 | 5.43E-16 | 1 | 1.00070913 | -1 | 1.000181785 | 1.001236754 | 0.008393017 |
| SNORD113-9 | POSTN | 0.3534 | 5.28E-13 | 1 | 1.000818864 | -1 | 1.000124785 | 1.001513425 | 0.020751298 |
| SNORD113-9 | CPXM1 | 0.4482 | 8.12E-21 | 1 | 1.000819904 | -1 | 1.000128426 | 1.001511861 | 0.020118534 |
| SNORD113-9 | TIMP2 | 0.3848 | 2.56E-15 | 1 | 1.000856295 | -1 | 1.000116092 | 1.001597045 | 0.023360066 |
| SNORD113-9 | HTRA1 | 0.3046 | 6.97E-10 | 1 | 1.000943158 | -1 | 1.000357176 | 1.001529484 | 0.00160393 |
| SNORD113-9 | SFRP4 | 0.352 | 6.62E-13 | 1 | 1.000946581 | -1 | 1.000137484 | 1.001756332 | 0.02183854 |
| SNORD113-9 | THBS1 | 0.3169 | 1.29E-10 | 1 | 1.000984444 | -1 | 1.000020005 | 1.001949813 | 0.045431382 |
| SNORD113-9 | EFEMP1 | 0.3085 | 4.14E-10 | 1 | 1.001042204 | -1 | 1.000131664 | 1.001953574 | 0.024863226 |
| SNORD113-9 | COL5A1 | 0.408 | 3.40E-17 | 1 | 1.001101528 | -1 | 1.000368188 | 1.001835406 | 0.003234319 |
| SNORD113-9 | CD248 | 0.3869 | 1.74E-15 | 1 | 1.001173103 | -1 | 1.000160253 | 1.00218698 | 0.023192395 |
| SNORD113-9 | CTHRC1 | 0.3672 | 5.44E-14 | 1 | 1.001199033 | -1 | 1.00020497 | 1.002194084 | 0.018062272 |
| SNORD113-9 | ANPEP | 0.3344 | 1.01E-11 | 1 | 1.001207009 | -1 | 1.000077257 | 1.002338038 | 0.036253322 |
| SNORD113-9 | COL6A3 | 0.4112 | 1.82E-17 | 1 | 1.001311415 | -1 | 1.000320688 | 1.002303123 | 0.009464593 |
| SNORD113-9 | COL5A2 | 0.382 | 4.24E-15 | 1 | 1.001364635 | -1 | 1.000489469 | 1.002240567 | 0.002236437 |
| SNORD113-9 | DCN | 0.3646 | 8.43E-14 | 1 | 1.001422659 | -1 | 1.000168141 | 1.00267875 | 0.026226486 |
| SNORD113-9 | EMILIN1 | 0.3794 | 6.61E-15 | 1 | 1.001431989 | -1 | 1.000062854 | 1.002802999 | 0.040363822 |
| SNORD113-9 | SERPINF1 | 0.3454 | 1.87E-12 | 1 | 1.001495138 | -1 | 1.000512751 | 1.00247849 | 0.002847673 |
| SNORD113-9 | FSTL1 | 0.3614 | 1.44E-13 | 1 | 1.001666146 | -1 | 1.000170982 | 1.003163544 | 0.028941262 |
| SNORD113-9 | PLOD1 | 0.3082 | 4.31E-10 | 1 | 1.001842448 | -1 | 1.000343593 | 1.00334355 | 0.015966819 |
| SNORD113-9 | OLFML3 | 0.3391 | 4.98E-12 | 1 | 1.001932997 | -1 | 1.000065016 | 1.003804469 | 0.042535054 |
| SNORD113-9 | FKBP10 | 0.326 | 3.52E-11 | 1 | 1.002043907 | -1 | 1.000907488 | 1.003181616 | 0.000420785 |
| SNORD113-9 | ECM1 | 0.3182 | 1.07E-10 | 1 | 1.002275755 | -1 | 1.000787026 | 1.003766698 | 0.00272391 |
| SNORD113-9 | THY1 | 0.4054 | 5.58E-17 | 1 | 1.002449433 | -1 | 1.000591368 | 1.004310949 | 0.009751286 |
| SNORD113-9 | MXRA5 | 0.3069 | 5.13E-10 | 1 | 1.002499336 | -1 | 1.000309082 | 1.004694386 | 0.025293315 |
| SNORD113-9 | ANXA6 | 0.321 | 7.18E-11 | 1 | 1.002522529 | -1 | 1.000249507 | 1.004800717 | 0.029601765 |
| SNORD113-9 | DPT | 0.3689 | 4.11E-14 | 1 | 1.002610769 | -1 | 1.000095484 | 1.005132379 | 0.041904781 |
| SNORD113-9 | CXCL12 | 0.3203 | 7.94E-11 | 1 | 1.003031717 | -1 | 1.0010126 | 1.005054906 | 0.003235942 |
| SNORD113-9 | CCDC80 | 0.3617 | 1.37E-13 | 1 | 1.003184088 | -1 | 1.000364839 | 1.006011282 | 0.026828356 |
| SNORD113-9 | COL11A1 | 0.3449 | 2.04E-12 | 1 | 1.003204197 | -1 | 1.000796703 | 1.005617482 | 0.009064642 |
| SNORD113-9 | IGFL2 | 0.3067 | 5.28E-10 | 1 | 1.003351898 | -1 | 1.001172101 | 1.005536441 | 0.00256452 |
| SNORD113-9 | COL18A1 | 0.3341 | 1.05E-11 | 1 | 1.003462052 | -1 | 1.001036083 | 1.0058939 | 0.005134475 |
| SNORD113-9 | SPON2 | 0.3935 | 5.28E-16 | 1 | 1.003507903 | -1 | 1.000001226 | 1.007026876 | 0.049919811 |
| SNORD113-9 | OLFML2B | 0.3452 | 1.92E-12 | 1 | 1.003526527 | -1 | 1.00082148 | 1.006238884 | 0.010581524 |
| SNORD113-9 | DKK3 | 0.3041 | 7.49E-10 | 1 | 1.004156656 | -1 | 1.00057304 | 1.007753108 | 0.022964569 |
| SNORD113-9 | APCDD1L | 0.3002 | 1.26E-09 | 1 | 1.004279033 | -1 | 1.000009182 | 1.008567116 | 0.049508317 |
| SNORD113-9 | ITGA11 | 0.373 | 2.03E-14 | 1 | 1.004323725 | -1 | 1.000105859 | 1.00855938 | 0.04450946 |
| SNORD113-9 | PXDN | 0.3738 | 1.78E-14 | 1 | 1.004414984 | -1 | 1.001406387 | 1.007432621 | 0.003999754 |
| SNORD113-9 | MFAP5 | 0.3438 | 2.42E-12 | 1 | 1.004541981 | -1 | 1.001402799 | 1.007691004 | 0.00454263 |
| SNORD113-9 | COL15A1 | 0.3152 | 1.63E-10 | 1 | 1.00456293 | -1 | 1.000745854 | 1.008394565 | 0.019087754 |
| SNORD113-9 | PODN | 0.3125 | 2.38E-10 | 1 | 1.004898743 | -1 | 1.000491957 | 1.00932494 | 0.029309199 |
| SNORD113-9 | PDGFRB | 0.3865 | 1.88E-15 | 1 | 1.004917066 | -1 | 1.002010771 | 1.007831791 | 0.000902339 |
| SNORD113-9 | MSC | 0.3461 | 1.67E-12 | 1 | 1.005440651 | -1 | 1.001943591 | 1.008949917 | 0.002271482 |
| SNORD113-9 | FIBIN | 0.362 | 1.30E-13 | 1 | 1.005483698 | -1 | 1.000095012 | 1.010901419 | 0.046084749 |
| SNORD113-9 | GAS1 | 0.3173 | 1.22E-10 | 1 | 1.005521175 | -1 | 1.001340688 | 1.009719114 | 0.009590332 |
| SNORD113-9 | CRISPLD2 | 0.3704 | 3.15E-14 | 1 | 1.005828473 | -1 | 1.001181009 | 1.010497511 | 0.013913936 |
| SNORD113-9 | COL16A1 | 0.3305 | 1.79E-11 | 1 | 1.005832915 | -1 | 1.001718496 | 1.009964235 | 0.005419526 |
| SNORD113-9 | COX7A1 | 0.3197 | 8.67E-11 | 1 | 1.0059047 | -1 | 1.0001388 | 1.011703841 | 0.04471937 |
| SNORD113-9 | TMEM158 | 0.3039 | 7.68E-10 | 1 | 1.005993058 | -1 | 1.00327708 | 1.008716387 | 1.48E-05 |
| SNORD113-9 | SLC2A3 | 0.3278 | 2.70E-11 | 1 | 1.006031275 | -1 | 1.002826112 | 1.009246682 | 0.000221327 |
| SNORD113-9 | LRRC15 | 0.3861 | 2.04E-15 | 1 | 1.006228078 | -1 | 1.000994422 | 1.011489098 | 0.019620572 |
| SNORD113-9 | C1QTNF6 | 0.3075 | 4.71E-10 | 1 | 1.006862491 | -1 | 1.00262985 | 1.011113 | 0.001463059 |
| SNORD113-9 | FNDC1 | 0.3977 | 2.39E-16 | 1 | 1.006911911 | -1 | 1.000853549 | 1.013006947 | 0.025282956 |
| SNORD113-9 | LRRC32 | 0.3616 | 1.39E-13 | 1 | 1.00701501 | -1 | 1.001385683 | 1.012675984 | 0.014521111 |
| SNORD113-9 | FAM20C | 0.3201 | 8.14E-11 | 1 | 1.007035826 | -1 | 1.002068345 | 1.012027931 | 0.005454086 |
| SNORD113-9 | TRPV2 | 0.3003 | 1.23E-09 | 1 | 1.007109415 | -1 | 1.000509354 | 1.013753014 | 0.034707077 |
| SNORD113-9 | WISP1 | 0.3612 | 1.47E-13 | 1 | 1.007839005 | -1 | 1.001547358 | 1.014170176 | 0.014530217 |
| SNORD113-9 | GFPT2 | 0.328 | 2.62E-11 | 1 | 1.007866619 | -1 | 1.002496357 | 1.01326565 | 0.00404505 |
| SNORD113-9 | DPYSL2 | 0.3011 | 1.11E-09 | 1 | 1.008296552 | -1 | 1.002951209 | 1.013670383 | 0.002314785 |
| SNORD113-9 | TGFB3 | 0.3377 | 6.12E-12 | 1 | 1.008855499 | -1 | 1.001615323 | 1.01614801 | 0.016432038 |
| SNORD113-9 | MOXD1 | 0.3127 | 2.31E-10 | 1 | 1.008966361 | -1 | 1.00153758 | 1.016450244 | 0.017911378 |
| SNORD113-9 | TWIST2 | 0.3815 | 4.64E-15 | 1 | 1.008996517 | -1 | 1.000415368 | 1.017651271 | 0.039853737 |
| SNORD113-9 | GPX8 | 0.311 | 2.93E-10 | 1 | 1.00907949 | -1 | 1.001680373 | 1.016533262 | 0.016079864 |
| SNORD113-9 | ADAM12 | 0.3546 | 4.35E-13 | 1 | 1.009131107 | -1 | 1.001736365 | 1.016580437 | 0.015423381 |
| SNORD113-9 | CDH11 | 0.3594 | 2.00E-13 | 1 | 1.009244457 | -1 | 1.001585393 | 1.01696209 | 0.01790679 |
| SNORD113-9 | COL8A1 | 0.3643 | 8.88E-14 | 1 | 1.009255381 | -1 | 1.003637967 | 1.014904235 | 0.001215816 |
| SNORD113-9 | COL5A3 | 0.3293 | 2.15E-11 | 1 | 1.009501993 | -1 | 1.003816575 | 1.015219612 | 0.001031068 |
| SNORD113-9 | LOX | 0.3444 | 2.19E-12 | 1 | 1.009933472 | -1 | 1.004064872 | 1.015836373 | 0.00088655 |
| SNORD113-9 | LRRC17 | 0.3155 | 1.56E-10 | 1 | 1.010295767 | -1 | 1.001460636 | 1.019208843 | 0.022274581 |
| SNORD113-9 | FBN1 | 0.3556 | 3.72E-13 | 1 | 1.010620672 | -1 | 1.003721573 | 1.017567192 | 0.002504261 |
| SNORD113-9 | TWIST1 | 0.3568 | 3.06E-13 | 1 | 1.010715213 | -1 | 1.003365738 | 1.018118521 | 0.004205262 |
| SNORD113-9 | OGN | 0.3029 | 8.81E-10 | 1 | 1.010731615 | -1 | 1.00018996 | 1.021384376 | 0.045991472 |
| SNORD113-9 | PDGFRA | 0.3226 | 5.70E-11 | 1 | 1.010855409 | -1 | 1.002655526 | 1.019122351 | 0.009373181 |
| SNORD113-9 | LGI2 | 0.3186 | 1.02E-10 | 1 | 1.011401414 | -1 | 1.001085819 | 1.021823304 | 0.03020106 |
| SNORD113-9 | NRP2 | 0.3107 | 3.07E-10 | 1 | 1.012174485 | -1 | 1.002662959 | 1.02177624 | 0.012003863 |
| SNORD113-9 | TNN | 0.3162 | 1.42E-10 | 1 | 1.012698453 | -1 | 1.005788838 | 1.019655536 | 0.000303371 |
| SNORD113-9 | ADAMTS14 | 0.4123 | 1.45E-17 | 1 | 1.013343436 | -1 | 1.003814737 | 1.022962586 | 0.005962441 |
| SNORD113-9 | GLT8D2 | 0.4019 | 1.09E-16 | 1 | 1.013953288 | -1 | 1.002843024 | 1.025186641 | 0.013701644 |
| SNORD113-9 | PDZRN3 | 0.3074 | 4.80E-10 | 1 | 1.013987992 | -1 | 1.001459669 | 1.026673046 | 0.028530659 |
| SNORD113-9 | PODNL1 | 0.3521 | 6.47E-13 | 1 | 1.015257607 | -1 | 1.001838296 | 1.028856665 | 0.025714293 |
| SNORD113-9 | DACT1 | 0.3588 | 2.20E-13 | 1 | 1.015889456 | -1 | 1.004810652 | 1.027090413 | 0.004835992 |
| SNORD113-9 | ARSI | 0.3087 | 4.02E-10 | 1 | 1.0161697 | -1 | 1.006368769 | 1.026066081 | 0.001179334 |
| SNORD113-9 | RAB3IL1 | 0.3393 | 4.81E-12 | 1 | 1.016473481 | -1 | 1.002627198 | 1.030510981 | 0.019548563 |
| SNORD113-9 | NID2 | 0.3714 | 2.68E-14 | 1 | 1.017909 | -1 | 1.009169059 | 1.026724634 | 5.47E-05 |
| SNORD113-9 | CSDC2 | 0.3044 | 7.15E-10 | 1 | 1.017982646 | -1 | 1.000720498 | 1.035542561 | 0.041101621 |
| SNORD113-9 | KCNE4 | 0.3371 | 6.72E-12 | 1 | 1.022875931 | -1 | 1.006910368 | 1.039094644 | 0.004833136 |
| SNORD113-9 | ZNF469 | 0.3558 | 3.59E-13 | 1 | 1.023037438 | -1 | 1.003799612 | 1.042643956 | 0.018697359 |
| SNORD113-9 | PDGFC | 0.316 | 1.46E-10 | 1 | 1.023385952 | -1 | 1.008500904 | 1.038490698 | 0.001985947 |
| SNORD113-9 | ADAMTS12 | 0.3335 | 1.15E-11 | 1 | 1.024952841 | -1 | 1.009044264 | 1.041112232 | 0.002014699 |
| SNORD113-9 | ZNF521 | 0.3669 | 5.76E-14 | 1 | 1.026246924 | -1 | 1.000057698 | 1.053121986 | 0.049490855 |
| SNORD113-9 | P4HA3 | 0.3591 | 2.09E-13 | 1 | 1.027945298 | -1 | 1.004916686 | 1.051501632 | 0.017114521 |
| SNORD113-9 | HS3ST3A1 | 0.3101 | 3.32E-10 | 1 | 1.028622855 | -1 | 1.004204875 | 1.053634575 | 0.021319276 |
| SNORD113-9 | GAS7 | 0.318 | 1.10E-10 | 1 | 1.028801708 | -1 | 1.011444329 | 1.046456956 | 0.001072727 |
| SNORD113-9 | ITGBL1 | 0.3519 | 6.70E-13 | 1 | 1.029588809 | -1 | 1.008184159 | 1.051447899 | 0.006520643 |
| SNORD113-9 | OMD | 0.328 | 2.60E-11 | 1 | 1.02992045 | -1 | 1.004643296 | 1.055833585 | 0.020052937 |
| SNORD113-9 | GUCA1A | 0.3241 | 4.59E-11 | 1 | 1.030217867 | -1 | 1.007879036 | 1.053051821 | 0.007776241 |
| SNORD113-9 | OLFML1 | 0.3736 | 1.84E-14 | 1 | 1.030479979 | -1 | 1.003080182 | 1.058628219 | 0.028989072 |
| SNORD113-9 | MAP1A | 0.3136 | 2.04E-10 | 1 | 1.030633575 | -1 | 1.010629905 | 1.051033182 | 0.00255013 |
| SNORD113-9 | GXYLT2 | 0.314 | 1.93E-10 | 1 | 1.030773216 | -1 | 1.009857544 | 1.052122082 | 0.003757758 |
| SNORD113-9 | PRR16 | 0.3728 | 2.11E-14 | 1 | 1.031268074 | -1 | 1.007512054 | 1.055584235 | 0.0096153 |
| SNORD113-9 | PLXDC1 | 0.3109 | 2.97E-10 | 1 | 1.035834185 | -1 | 1.005338494 | 1.067254924 | 0.020933819 |
| SNORD113-9 | FAM110B | 0.3255 | 3.76E-11 | 1 | 1.036176782 | -1 | 1.011424806 | 1.061534499 | 0.003965875 |
| SNORD113-9 | LAMA2 | 0.3248 | 4.19E-11 | 1 | 1.036295446 | -1 | 1.015214231 | 1.057814419 | 0.000674068 |
| SNORD113-9 | KANK4 | 0.3082 | 4.30E-10 | 1 | 1.03846335 | -1 | 1.019580491 | 1.057695924 | 5.55E-05 |
| SNORD113-9 | GLIS1 | 0.3289 | 2.29E-11 | 1 | 1.039512498 | -1 | 1.002910024 | 1.077450826 | 0.034103565 |
| SNORD113-9 | ADAMTS16 | 0.3609 | 1.56E-13 | 1 | 1.03989703 | -1 | 1.013896201 | 1.066564636 | 0.00246029 |
| SNORD113-9 | HSPB2 | 0.3507 | 8.08E-13 | 1 | 1.045277968 | -1 | 1.003534793 | 1.088757497 | 0.03319967 |
| SNORD113-9 | ECM2 | 0.3033 | 8.27E-10 | 1 | 1.045658733 | -1 | 1.011229437 | 1.081260242 | 0.008957217 |
| SNORD113-9 | SGCD | 0.373 | 2.03E-14 | 1 | 1.052210708 | -1 | 1.007025413 | 1.09942347 | 0.023051635 |
| SNORD113-9 | ADAMTSL1 | 0.3113 | 2.79E-10 | 1 | 1.053811409 | -1 | 1.019914031 | 1.088835385 | 0.001677915 |
| SNORD113-9 | PDLIM2 | 0.3066 | 5.36E-10 | 1 | 1.077041372 | -1 | 1.032933196 | 1.123033051 | 0.00050379 |
| SNORD113-9 | STK32B | 0.3424 | 2.97E-12 | 1 | 1.077544258 | -1 | 1.029155729 | 1.128207903 | 0.001443008 |
| SNORD113-9 | ALDH1L2 | 0.3397 | 4.50E-12 | 1 | 1.083772372 | -1 | 1.044840881 | 1.124154477 | 1.63E-05 |
| SNORD113-9 | RFX8 | 0.3424 | 2.99E-12 | 1 | 1.097553265 | -1 | 1.030679795 | 1.168765678 | 0.00370668 |
| SNORD113-9 | FAM180A | 0.3638 | 9.63E-14 | 1 | 1.13783635 | -1 | 1.056095936 | 1.225903362 | 0.000686564 |
| SNORD113-9 | UBE2QL1 | 0.3364 | 7.45E-12 | 1 | 1.141344663 | -1 | 1.06992342 | 1.217533531 | 6.07E-05 |
| SNORD114-1 | DDTL | -0.3791 | 7.08E-15 | -1 | 0.935211514 | 1 | 0.886694078 | 0.986383689 | 0.013725599 |
| SNORD114-1 | TTLL3 | -0.3418 | 3.28E-12 | -1 | 0.93790748 | 1 | 0.905832418 | 0.971118303 | 0.000305372 |
| SNORD114-1 | NSUN5P1 | -0.3853 | 2.34E-15 | -1 | 0.960912376 | 1 | 0.93421853 | 0.988368958 | 0.005539389 |
| SNORD114-1 | SFI1 | -0.3036 | 7.97E-10 | -1 | 0.961777457 | 1 | 0.925175642 | 0.999827313 | 0.048988926 |
| SNORD114-1 | MZF1 | -0.3414 | 3.48E-12 | -1 | 0.962682409 | 1 | 0.93693485 | 0.989137528 | 0.005967046 |
| SNORD114-1 | ECHDC2 | -0.3622 | 1.26E-13 | -1 | 0.965698173 | 1 | 0.946155558 | 0.985644437 | 0.000819329 |
| SNORD114-1 | NEIL1 | -0.3169 | 1.29E-10 | -1 | 0.96915919 | 1 | 0.944494099 | 0.994468399 | 0.017233444 |
| SNORD114-1 | C1orf159 | -0.3196 | 8.78E-11 | -1 | 0.9715012 | 1 | 0.945684451 | 0.998022734 | 0.03537908 |
| SNORD114-1 | AMT | -0.3044 | 7.16E-10 | -1 | 0.97534267 | 1 | 0.954421778 | 0.996722146 | 0.024024379 |
| SNORD114-1 | ME3 | -0.3075 | 4.75E-10 | -1 | 0.975582124 | 1 | 0.96000646 | 0.991410496 | 0.002608091 |
| SNORD114-1 | RPP21 | -0.3002 | 1.26E-09 | -1 | 0.977705585 | 1 | 0.962378519 | 0.993276754 | 0.00516197 |
| SNORD114-1 | FAM193B | -0.3315 | 1.55E-11 | -1 | 0.97781171 | 1 | 0.963748649 | 0.99207998 | 0.002399236 |
| SNORD114-1 | CCDC130 | -0.3784 | 7.99E-15 | -1 | 0.981268759 | 1 | 0.967075423 | 0.995670404 | 0.01097003 |
| SNORD114-1 | HEXDC | -0.3301 | 1.93E-11 | -1 | 0.982744975 | 1 | 0.96881516 | 0.996875075 | 0.016863751 |
| SNORD114-1 | CSAD | -0.3209 | 7.35E-11 | -1 | 0.983385295 | 1 | 0.967333644 | 0.999703304 | 0.046009794 |
| SNORD114-1 | AHSA2 | -0.3121 | 2.51E-10 | -1 | 0.984417927 | 1 | 0.96996454 | 0.999086684 | 0.037429947 |
| SNORD114-1 | C6orf136 | -0.3286 | 2.40E-11 | -1 | 0.98452113 | 1 | 0.972598984 | 0.996589417 | 0.012088063 |
| SNORD114-1 | RBM6 | -0.3113 | 2.80E-10 | -1 | 0.987150993 | 1 | 0.977842309 | 0.996548293 | 0.007467633 |
| SNORD114-1 | KIAA0907 | -0.3084 | 4.16E-10 | -1 | 0.98794209 | 1 | 0.977414993 | 0.998582568 | 0.02645438 |
| SNORD114-1 | IP6K2 | -0.3127 | 2.33E-10 | -1 | 0.98878383 | 1 | 0.981936108 | 0.995679306 | 0.001466821 |
| SNORD114-1 | ENGASE | -0.3112 | 2.83E-10 | -1 | 0.988993536 | 1 | 0.980915162 | 0.99713844 | 0.00817483 |
| SNORD114-1 | ZNF692 | -0.3407 | 3.87E-12 | -1 | 0.99020294 | 1 | 0.9809582 | 0.999534804 | 0.039668023 |
| SNORD114-1 | CRB3 | -0.3099 | 3.40E-10 | -1 | 0.991625124 | 1 | 0.984759293 | 0.998538825 | 0.017670318 |
| SNORD114-1 | CCNL2 | -0.3078 | 4.51E-10 | -1 | 0.995585923 | 1 | 0.992139566 | 0.999044251 | 0.012404408 |
| SNORD114-1 | NR2F6 | -0.347 | 1.46E-12 | -1 | 0.996378288 | 1 | 0.993237536 | 0.999528972 | 0.024293953 |
| SNORD114-1 | AES | -0.3616 | 1.40E-13 | -1 | 0.998267401 | 1 | 0.996943882 | 0.999592677 | 0.01041202 |
| SNORD114-1 | COL1A1 | 0.4105 | 2.09E-17 | 1 | 1.000069718 | -1 | 1.000015973 | 1.000123466 | 0.011007254 |
| SNORD114-1 | COL3A1 | 0.4208 | 2.67E-18 | 1 | 1.000078169 | -1 | 1.000011086 | 1.000145256 | 0.022378028 |
| SNORD114-1 | COL1A2 | 0.4067 | 4.32E-17 | 1 | 1.000148497 | -1 | 1.000038427 | 1.00025858 | 0.00818701 |
| SNORD114-1 | SPARC | 0.3685 | 4.40E-14 | 1 | 1.000175584 | -1 | 1.000047509 | 1.000303676 | 0.007208304 |
| SNORD114-1 | BGN | 0.3394 | 4.75E-12 | 1 | 1.000220423 | -1 | 1.000040002 | 1.000400876 | 0.016639878 |
| SNORD114-1 | VIM | 0.3152 | 1.63E-10 | 1 | 1.000274 | -1 | 1.000081691 | 1.000466346 | 0.005227817 |
| SNORD114-1 | COL6A1 | 0.3261 | 3.43E-11 | 1 | 1.000284727 | -1 | 1.000055672 | 1.000513833 | 0.014833984 |
| SNORD114-1 | LUM | 0.3978 | 2.38E-16 | 1 | 1.000341435 | -1 | 1.000064347 | 1.0006186 | 0.015726709 |
| SNORD114-1 | CTSK | 0.3749 | 1.45E-14 | 1 | 1.000347461 | -1 | 1.000102254 | 1.000592729 | 0.005479058 |
| SNORD114-1 | FN1 | 0.3327 | 1.31E-11 | 1 | 1.000398967 | -1 | 1.000214583 | 1.000583385 | 2.22E-05 |
| SNORD114-1 | CTGF | 0.3205 | 7.74E-11 | 1 | 1.000535855 | -1 | 1.000004875 | 1.001067117 | 0.047932992 |
| SNORD114-1 | AEBP1 | 0.3584 | 2.33E-13 | 1 | 1.000594119 | -1 | 1.000119758 | 1.001068705 | 0.014091262 |
| SNORD114-1 | TGFBI | 0.3103 | 3.21E-10 | 1 | 1.000611752 | -1 | 1.000147722 | 1.001075997 | 0.009763254 |
| SNORD114-1 | ISLR | 0.3435 | 2.53E-12 | 1 | 1.00070913 | -1 | 1.000181785 | 1.001236754 | 0.008393017 |
| SNORD114-1 | POSTN | 0.364 | 9.35E-14 | 1 | 1.000818864 | -1 | 1.000124785 | 1.001513425 | 0.020751298 |
| SNORD114-1 | CPXM1 | 0.3798 | 6.18E-15 | 1 | 1.000819904 | -1 | 1.000128426 | 1.001511861 | 0.020118534 |
| SNORD114-1 | TIMP2 | 0.3231 | 5.34E-11 | 1 | 1.000856295 | -1 | 1.000116092 | 1.001597045 | 0.023360066 |
| SNORD114-1 | SFRP4 | 0.3073 | 4.82E-10 | 1 | 1.000946581 | -1 | 1.000137484 | 1.001756332 | 0.02183854 |
| SNORD114-1 | THBS1 | 0.3524 | 6.19E-13 | 1 | 1.000984444 | -1 | 1.000020005 | 1.001949813 | 0.045431382 |
| SNORD114-1 | EFEMP1 | 0.3009 | 1.15E-09 | 1 | 1.001042204 | -1 | 1.000131664 | 1.001953574 | 0.024863226 |
| SNORD114-1 | COL5A1 | 0.4181 | 4.65E-18 | 1 | 1.001101528 | -1 | 1.000368188 | 1.001835406 | 0.003234319 |
| SNORD114-1 | CD248 | 0.3428 | 2.81E-12 | 1 | 1.001173103 | -1 | 1.000160253 | 1.00218698 | 0.023192395 |
| SNORD114-1 | CTHRC1 | 0.3487 | 1.12E-12 | 1 | 1.001199033 | -1 | 1.00020497 | 1.002194084 | 0.018062272 |
| SNORD114-1 | ANPEP | 0.339 | 5.06E-12 | 1 | 1.001207009 | -1 | 1.000077257 | 1.002338038 | 0.036253322 |
| SNORD114-1 | COL6A3 | 0.4049 | 6.22E-17 | 1 | 1.001311415 | -1 | 1.000320688 | 1.002303123 | 0.009464593 |
| SNORD114-1 | COL5A2 | 0.403 | 8.94E-17 | 1 | 1.001364635 | -1 | 1.000489469 | 1.002240567 | 0.002236437 |
| SNORD114-1 | DCN | 0.3801 | 5.93E-15 | 1 | 1.001422659 | -1 | 1.000168141 | 1.00267875 | 0.026226486 |
| SNORD114-1 | SERPINF1 | 0.3108 | 2.99E-10 | 1 | 1.001495138 | -1 | 1.000512751 | 1.00247849 | 0.002847673 |
| SNORD114-1 | FSTL1 | 0.3261 | 3.47E-11 | 1 | 1.001666146 | -1 | 1.000170982 | 1.003163544 | 0.028941262 |
| SNORD114-1 | CLIC4 | 0.3066 | 5.33E-10 | 1 | 1.001771503 | -1 | 1.000207711 | 1.003337741 | 0.02638299 |
| SNORD114-1 | TNC | 0.3154 | 1.59E-10 | 1 | 1.001874973 | -1 | 1.000608224 | 1.003143326 | 0.003708906 |
| SNORD114-1 | OLFML3 | 0.3489 | 1.07E-12 | 1 | 1.001932997 | -1 | 1.000065016 | 1.003804469 | 0.042535054 |
| SNORD114-1 | FKBP10 | 0.3176 | 1.17E-10 | 1 | 1.002043907 | -1 | 1.000907488 | 1.003181616 | 0.000420785 |
| SNORD114-1 | MYADM | 0.3176 | 1.16E-10 | 1 | 1.00220069 | -1 | 1.000519045 | 1.003885162 | 0.01030041 |
| SNORD114-1 | SULF2 | 0.3134 | 2.11E-10 | 1 | 1.002276044 | -1 | 1.000830843 | 1.003723332 | 0.002014913 |
| SNORD114-1 | THY1 | 0.3384 | 5.52E-12 | 1 | 1.002449433 | -1 | 1.000591368 | 1.004310949 | 0.009751286 |
| SNORD114-1 | PLS3 | 0.3217 | 6.52E-11 | 1 | 1.002471342 | -1 | 1.000237566 | 1.004710106 | 0.030107936 |
| SNORD114-1 | MXRA5 | 0.3456 | 1.81E-12 | 1 | 1.002499336 | -1 | 1.000309082 | 1.004694386 | 0.025293315 |
| SNORD114-1 | DPT | 0.3151 | 1.66E-10 | 1 | 1.002610769 | -1 | 1.000095484 | 1.005132379 | 0.041904781 |
| SNORD114-1 | CALU | 0.3536 | 5.13E-13 | 1 | 1.00312716 | -1 | 1.001521692 | 1.004735202 | 0.000133151 |
| SNORD114-1 | CCDC80 | 0.3473 | 1.39E-12 | 1 | 1.003184088 | -1 | 1.000364839 | 1.006011282 | 0.026828356 |
| SNORD114-1 | COL11A1 | 0.3678 | 4.97E-14 | 1 | 1.003204197 | -1 | 1.000796703 | 1.005617482 | 0.009064642 |
| SNORD114-1 | OLFML2B | 0.3297 | 2.05E-11 | 1 | 1.003526527 | -1 | 1.00082148 | 1.006238884 | 0.010581524 |
| SNORD114-1 | DKK3 | 0.3404 | 4.08E-12 | 1 | 1.004156656 | -1 | 1.00057304 | 1.007753108 | 0.022964569 |
| SNORD114-1 | ITGA11 | 0.3775 | 9.35E-15 | 1 | 1.004323725 | -1 | 1.000105859 | 1.00855938 | 0.04450946 |
| SNORD114-1 | CORO1C | 0.3125 | 2.38E-10 | 1 | 1.004379489 | -1 | 1.000331155 | 1.008444206 | 0.033952073 |
| SNORD114-1 | PXDN | 0.3853 | 2.35E-15 | 1 | 1.004414984 | -1 | 1.001406387 | 1.007432621 | 0.003999754 |
| SNORD114-1 | MFAP5 | 0.3608 | 1.57E-13 | 1 | 1.004541981 | -1 | 1.001402799 | 1.007691004 | 0.00454263 |
| SNORD114-1 | COL15A1 | 0.3457 | 1.79E-12 | 1 | 1.00456293 | -1 | 1.000745854 | 1.008394565 | 0.019087754 |
| SNORD114-1 | PDGFRB | 0.3834 | 3.27E-15 | 1 | 1.004917066 | -1 | 1.002010771 | 1.007831791 | 0.000902339 |
| SNORD114-1 | ARL4C | 0.327 | 3.03E-11 | 1 | 1.005075669 | -1 | 1.002078929 | 1.008081371 | 0.00089024 |
| SNORD114-1 | MSC | 0.3268 | 3.13E-11 | 1 | 1.005440651 | -1 | 1.001943591 | 1.008949917 | 0.002271482 |
| SNORD114-1 | FIBIN | 0.3452 | 1.94E-12 | 1 | 1.005483698 | -1 | 1.000095012 | 1.010901419 | 0.046084749 |
| SNORD114-1 | GAS1 | 0.3206 | 7.64E-11 | 1 | 1.005521175 | -1 | 1.001340688 | 1.009719114 | 0.009590332 |
| SNORD114-1 | CRISPLD2 | 0.3349 | 9.41E-12 | 1 | 1.005828473 | -1 | 1.001181009 | 1.010497511 | 0.013913936 |
| SNORD114-1 | SLC2A3 | 0.3093 | 3.69E-10 | 1 | 1.006031275 | -1 | 1.002826112 | 1.009246682 | 0.000221327 |
| SNORD114-1 | LRRC15 | 0.3688 | 4.16E-14 | 1 | 1.006228078 | -1 | 1.000994422 | 1.011489098 | 0.019620572 |
| SNORD114-1 | F2R | 0.3219 | 6.35E-11 | 1 | 1.006727641 | -1 | 1.000595321 | 1.012897543 | 0.031486051 |
| SNORD114-1 | FNDC1 | 0.3684 | 4.46E-14 | 1 | 1.006911911 | -1 | 1.000853549 | 1.013006947 | 0.025282956 |
| SNORD114-1 | CNTN1 | 0.3046 | 7.02E-10 | 1 | 1.006980005 | -1 | 1.00357237 | 1.01039921 | 5.77E-05 |
| SNORD114-1 | LRRC32 | 0.3421 | 3.13E-12 | 1 | 1.00701501 | -1 | 1.001385683 | 1.012675984 | 0.014521111 |
| SNORD114-1 | GLIS2 | 0.3039 | 7.64E-10 | 1 | 1.007769055 | -1 | 1.000152247 | 1.015443869 | 0.045576725 |
| SNORD114-1 | WISP1 | 0.3959 | 3.37E-16 | 1 | 1.007839005 | -1 | 1.001547358 | 1.014170176 | 0.014530217 |
| SNORD114-1 | GFPT2 | 0.3466 | 1.54E-12 | 1 | 1.007866619 | -1 | 1.002496357 | 1.01326565 | 0.00404505 |
| SNORD114-1 | FKBP9 | 0.3784 | 7.92E-15 | 1 | 1.008576148 | -1 | 1.004715332 | 1.0124518 | 1.28E-05 |
| SNORD114-1 | ATP8B2 | 0.3039 | 7.66E-10 | 1 | 1.008623581 | -1 | 1.002755704 | 1.014525796 | 0.003921925 |
| SNORD114-1 | RBM24 | 0.3052 | 6.45E-10 | 1 | 1.008687508 | -1 | 1.00074962 | 1.016688358 | 0.031884357 |
| SNORD114-1 | TGFB3 | 0.3384 | 5.50E-12 | 1 | 1.008855499 | -1 | 1.001615323 | 1.01614801 | 0.016432038 |
| SNORD114-1 | HEG1 | 0.3178 | 1.13E-10 | 1 | 1.008994835 | -1 | 1.000729031 | 1.017328912 | 0.032875056 |
| SNORD114-1 | TWIST2 | 0.3502 | 8.87E-13 | 1 | 1.008996517 | -1 | 1.000415368 | 1.017651271 | 0.039853737 |
| SNORD114-1 | GPX8 | 0.3283 | 2.52E-11 | 1 | 1.00907949 | -1 | 1.001680373 | 1.016533262 | 0.016079864 |
| SNORD114-1 | COPZ2 | 0.3011 | 1.12E-09 | 1 | 1.009116916 | -1 | 1.004446429 | 1.01380912 | 0.000125877 |
| SNORD114-1 | ADAM12 | 0.3811 | 4.99E-15 | 1 | 1.009131107 | -1 | 1.001736365 | 1.016580437 | 0.015423381 |
| SNORD114-1 | CDH11 | 0.3958 | 3.43E-16 | 1 | 1.009244457 | -1 | 1.001585393 | 1.01696209 | 0.01790679 |
| SNORD114-1 | COL8A1 | 0.397 | 2.77E-16 | 1 | 1.009255381 | -1 | 1.003637967 | 1.014904235 | 0.001215816 |
| SNORD114-1 | COL5A3 | 0.3482 | 1.21E-12 | 1 | 1.009501993 | -1 | 1.003816575 | 1.015219612 | 0.001031068 |
| SNORD114-1 | KIRREL | 0.3424 | 3.00E-12 | 1 | 1.009797896 | -1 | 1.000430799 | 1.019252698 | 0.04031128 |
| SNORD114-1 | LOX | 0.3744 | 1.59E-14 | 1 | 1.009933472 | -1 | 1.004064872 | 1.015836373 | 0.00088655 |
| SNORD114-1 | CHSY1 | 0.3376 | 6.28E-12 | 1 | 1.009994764 | -1 | 1.000331604 | 1.01975127 | 0.042605348 |
| SNORD114-1 | TRAM2 | 0.3309 | 1.69E-11 | 1 | 1.009996415 | -1 | 1.001097384 | 1.018974552 | 0.02760443 |
| SNORD114-1 | FBN1 | 0.3781 | 8.35E-15 | 1 | 1.010620672 | -1 | 1.003721573 | 1.017567192 | 0.002504261 |
| SNORD114-1 | TWIST1 | 0.3334 | 1.18E-11 | 1 | 1.010715213 | -1 | 1.003365738 | 1.018118521 | 0.004205262 |
| SNORD114-1 | LGI2 | 0.3032 | 8.47E-10 | 1 | 1.011401414 | -1 | 1.001085819 | 1.021823304 | 0.03020106 |
| SNORD114-1 | CHST15 | 0.3501 | 8.94E-13 | 1 | 1.011555817 | -1 | 1.002018506 | 1.021183906 | 0.017446133 |
| SNORD114-1 | NRP2 | 0.3356 | 8.43E-12 | 1 | 1.012174485 | -1 | 1.002662959 | 1.02177624 | 0.012003863 |
| SNORD114-1 | 7-Sep | 0.3132 | 2.14E-10 | 1 | 1.012637629 | -1 | 1.004141012 | 1.02120614 | 0.003486697 |
| SNORD114-1 | ADAMTS14 | 0.3337 | 1.12E-11 | 1 | 1.013343436 | -1 | 1.003814737 | 1.022962586 | 0.005962441 |
| SNORD114-1 | CCDC8 | 0.3295 | 2.09E-11 | 1 | 1.013688562 | -1 | 1.004370517 | 1.023093056 | 0.003907497 |
| SNORD114-1 | GLT8D2 | 0.3974 | 2.56E-16 | 1 | 1.013953288 | -1 | 1.002843024 | 1.025186641 | 0.013701644 |
| SNORD114-1 | PDZRN3 | 0.3236 | 4.94E-11 | 1 | 1.013987992 | -1 | 1.001459669 | 1.026673046 | 0.028530659 |
| SNORD114-1 | MACF1 | 0.3032 | 8.45E-10 | 1 | 1.0147593 | -1 | 1.000238812 | 1.029490583 | 0.04632325 |
| SNORD114-1 | PODNL1 | 0.3053 | 6.37E-10 | 1 | 1.015257607 | -1 | 1.001838296 | 1.028856665 | 0.025714293 |
| SNORD114-1 | DACT1 | 0.3359 | 8.11E-12 | 1 | 1.015889456 | -1 | 1.004810652 | 1.027090413 | 0.004835992 |
| SNORD114-1 | LEF1 | 0.3166 | 1.33E-10 | 1 | 1.015968674 | -1 | 1.004083267 | 1.02799477 | 0.0083231 |
| SNORD114-1 | GNB4 | 0.3366 | 7.27E-12 | 1 | 1.016060405 | -1 | 1.000273735 | 1.032096224 | 0.046127881 |
| SNORD114-1 | ARSI | 0.3311 | 1.65E-11 | 1 | 1.0161697 | -1 | 1.006368769 | 1.026066081 | 0.001179334 |
| SNORD114-1 | MAP1B | 0.3338 | 1.11E-11 | 1 | 1.016179517 | -1 | 1.007622392 | 1.024809313 | 0.000199301 |
| SNORD114-1 | TRPS1 | 0.3166 | 1.34E-10 | 1 | 1.016287047 | -1 | 1.000713421 | 1.032103037 | 0.04031779 |
| SNORD114-1 | PDE5A | 0.3029 | 8.75E-10 | 1 | 1.016300708 | -1 | 1.007239422 | 1.02544351 | 0.000402301 |
| SNORD114-1 | RAB3IL1 | 0.3221 | 6.17E-11 | 1 | 1.016473481 | -1 | 1.002627198 | 1.030510981 | 0.019548563 |
| SNORD114-1 | HGF | 0.3226 | 5.69E-11 | 1 | 1.016739451 | -1 | 1.003207048 | 1.030454395 | 0.015168482 |
| SNORD114-1 | GPR1 | 0.3128 | 2.29E-10 | 1 | 1.016750591 | -1 | 1.000639406 | 1.033121182 | 0.041510066 |
| SNORD114-1 | ERC1 | 0.3651 | 7.78E-14 | 1 | 1.016924235 | -1 | 1.004024082 | 1.029990135 | 0.009980269 |
| SNORD114-1 | PID1 | 0.3357 | 8.36E-12 | 1 | 1.017556078 | -1 | 1.000211727 | 1.035201191 | 0.047244786 |
| SNORD114-1 | NID2 | 0.374 | 1.71E-14 | 1 | 1.017909 | -1 | 1.009169059 | 1.026724634 | 5.47E-05 |
| SNORD114-1 | ZEB1 | 0.3134 | 2.09E-10 | 1 | 1.017965631 | -1 | 1.001220171 | 1.034991159 | 0.035373462 |
| SNORD114-1 | FKBP14 | 0.3206 | 7.57E-11 | 1 | 1.020614654 | -1 | 1.004563401 | 1.036922379 | 0.011638983 |
| SNORD114-1 | CSGALNACT2 | 0.3116 | 2.71E-10 | 1 | 1.021514978 | -1 | 1.006159894 | 1.037104397 | 0.005875513 |
| SNORD114-1 | ZNF469 | 0.4081 | 3.31E-17 | 1 | 1.023037438 | -1 | 1.003799612 | 1.042643956 | 0.018697359 |
| SNORD114-1 | PDGFC | 0.3331 | 1.22E-11 | 1 | 1.023385952 | -1 | 1.008500904 | 1.038490698 | 0.001985947 |
| SNORD114-1 | LATS2 | 0.3098 | 3.47E-10 | 1 | 1.023494331 | -1 | 1.002824297 | 1.044590411 | 0.025687728 |
| SNORD114-1 | EVC | 0.305 | 6.60E-10 | 1 | 1.024138725 | -1 | 1.003014771 | 1.04570756 | 0.024893765 |
| SNORD114-1 | ADAMTS12 | 0.4098 | 2.38E-17 | 1 | 1.024952841 | -1 | 1.009044264 | 1.041112232 | 0.002014699 |
| SNORD114-1 | EDNRA | 0.3671 | 5.58E-14 | 1 | 1.025294447 | -1 | 1.011227797 | 1.039556771 | 0.000394038 |
| SNORD114-1 | ZNF521 | 0.3924 | 6.46E-16 | 1 | 1.026246924 | -1 | 1.000057698 | 1.053121986 | 0.049490855 |
| SNORD114-1 | ITGA1 | 0.316 | 1.45E-10 | 1 | 1.027488354 | -1 | 1.008280188 | 1.047062444 | 0.004856483 |
| SNORD114-1 | P4HA3 | 0.3719 | 2.46E-14 | 1 | 1.027945298 | -1 | 1.004916686 | 1.051501632 | 0.017114521 |
| SNORD114-1 | ETV1 | 0.3409 | 3.79E-12 | 1 | 1.028137964 | -1 | 1.005066731 | 1.051738795 | 0.016556143 |
| SNORD114-1 | HS3ST3A1 | 0.3409 | 3.79E-12 | 1 | 1.028622855 | -1 | 1.004204875 | 1.053634575 | 0.021319276 |
| SNORD114-1 | GAS7 | 0.3434 | 2.58E-12 | 1 | 1.028801708 | -1 | 1.011444329 | 1.046456956 | 0.001072727 |
| SNORD114-1 | EBF1 | 0.3197 | 8.62E-11 | 1 | 1.028854336 | -1 | 1.000622516 | 1.057882696 | 0.04509128 |
| SNORD114-1 | OMD | 0.3237 | 4.89E-11 | 1 | 1.02992045 | -1 | 1.004643296 | 1.055833585 | 0.020052937 |
| SNORD114-1 | GUCA1A | 0.3033 | 8.28E-10 | 1 | 1.030217867 | -1 | 1.007879036 | 1.053051821 | 0.007776241 |
| SNORD114-1 | OLFML1 | 0.3687 | 4.22E-14 | 1 | 1.030479979 | -1 | 1.003080182 | 1.058628219 | 0.028989072 |
| SNORD114-1 | MAP1A | 0.3558 | 3.60E-13 | 1 | 1.030633575 | -1 | 1.010629905 | 1.051033182 | 0.00255013 |
| SNORD114-1 | OGFOD1 | 0.3005 | 1.20E-09 | 1 | 1.030726163 | -1 | 1.009416766 | 1.052485415 | 0.004521211 |
| SNORD114-1 | GXYLT2 | 0.3156 | 1.55E-10 | 1 | 1.030773216 | -1 | 1.009857544 | 1.052122082 | 0.003757758 |
| SNORD114-1 | PRR16 | 0.356 | 3.48E-13 | 1 | 1.031268074 | -1 | 1.007512054 | 1.055584235 | 0.0096153 |
| SNORD114-1 | NUDT11 | 0.3185 | 1.03E-10 | 1 | 1.032490134 | -1 | 1.014578582 | 1.050717899 | 0.000342369 |
| SNORD114-1 | ARHGAP31 | 0.3167 | 1.32E-10 | 1 | 1.033584387 | -1 | 1.006733448 | 1.061151476 | 0.013906648 |
| SNORD114-1 | LAMA2 | 0.3389 | 5.11E-12 | 1 | 1.036295446 | -1 | 1.015214231 | 1.057814419 | 0.000674068 |
| SNORD114-1 | SLIT2 | 0.3018 | 1.02E-09 | 1 | 1.037081917 | -1 | 1.005345017 | 1.069820692 | 0.021668345 |
| SNORD114-1 | KANK4 | 0.376 | 1.21E-14 | 1 | 1.03846335 | -1 | 1.019580491 | 1.057695924 | 5.55E-05 |
| SNORD114-1 | GLIS1 | 0.3168 | 1.30E-10 | 1 | 1.039512498 | -1 | 1.002910024 | 1.077450826 | 0.034103565 |
| SNORD114-1 | ADAMTS16 | 0.3428 | 2.83E-12 | 1 | 1.03989703 | -1 | 1.013896201 | 1.066564636 | 0.00246029 |
| SNORD114-1 | TCF4 | 0.3758 | 1.25E-14 | 1 | 1.041731611 | -1 | 1.022267867 | 1.061565941 | 2.15E-05 |
| SNORD114-1 | SACS | 0.3344 | 1.01E-11 | 1 | 1.041931761 | -1 | 1.00440665 | 1.080858828 | 0.028169659 |
| SNORD114-1 | GUCY1B3 | 0.35 | 9.11E-13 | 1 | 1.042378189 | -1 | 1.017833118 | 1.067515164 | 0.000640534 |
| SNORD114-1 | GUCY1A3 | 0.3387 | 5.30E-12 | 1 | 1.042550856 | -1 | 1.011174057 | 1.074901279 | 0.007524906 |
| SNORD114-1 | ECM2 | 0.3627 | 1.17E-13 | 1 | 1.045658733 | -1 | 1.011229437 | 1.081260242 | 0.008957217 |
| SNORD114-1 | MYO5A | 0.3758 | 1.25E-14 | 1 | 1.045872503 | -1 | 1.020372951 | 1.072009298 | 0.000368867 |
| SNORD114-1 | SGCD | 0.3823 | 4.03E-15 | 1 | 1.052210708 | -1 | 1.007025413 | 1.09942347 | 0.023051635 |
| SNORD114-1 | ARSB | 0.3257 | 3.67E-11 | 1 | 1.053360168 | -1 | 1.009287187 | 1.099357702 | 0.017131188 |
| SNORD114-1 | ADAMTSL1 | 0.3088 | 3.95E-10 | 1 | 1.053811409 | -1 | 1.019914031 | 1.088835385 | 0.001677915 |
| SNORD114-1 | PRKG1 | 0.3815 | 4.57E-15 | 1 | 1.053910662 | -1 | 1.013964256 | 1.095430807 | 0.007735949 |
| SNORD114-1 | HSPA12A | 0.3037 | 7.85E-10 | 1 | 1.058348705 | -1 | 1.003228712 | 1.116497133 | 0.037701354 |
| SNORD114-1 | CHSY3 | 0.3177 | 1.15E-10 | 1 | 1.058417527 | -1 | 1.006430582 | 1.113089845 | 0.027146089 |
| SNORD114-1 | ENPEP | 0.3122 | 2.48E-10 | 1 | 1.059874137 | -1 | 1.01899034 | 1.102398269 | 0.003764335 |
| SNORD114-1 | FAM126A | 0.3053 | 6.36E-10 | 1 | 1.063242673 | -1 | 1.029141589 | 1.098473713 | 0.00022687 |
| SNORD114-1 | PAPPA | 0.3681 | 4.65E-14 | 1 | 1.065194301 | -1 | 1.022793531 | 1.109352831 | 0.00230797 |
| SNORD114-1 | DSEL | 0.302 | 9.93E-10 | 1 | 1.067426199 | -1 | 1.009386182 | 1.128803536 | 0.022167782 |
| SNORD114-1 | GLI2 | 0.3547 | 4.26E-13 | 1 | 1.072698017 | -1 | 1.030067772 | 1.117092551 | 0.000694428 |
| SNORD114-1 | OSBPL6 | 0.326 | 3.52E-11 | 1 | 1.074239369 | -1 | 1.01799205 | 1.133594533 | 0.009058715 |
| SNORD114-1 | MMP16 | 0.3068 | 5.18E-10 | 1 | 1.074735849 | -1 | 1.007708019 | 1.146222043 | 0.028259503 |
| SNORD114-1 | STK32B | 0.3133 | 2.13E-10 | 1 | 1.077544258 | -1 | 1.029155729 | 1.128207903 | 0.001443008 |
| SNORD114-1 | SVEP1 | 0.3484 | 1.17E-12 | 1 | 1.079799408 | -1 | 1.004932847 | 1.160243458 | 0.036243452 |
| SNORD114-1 | SLC30A4 | 0.3311 | 1.66E-11 | 1 | 1.080355603 | -1 | 1.010912302 | 1.154569221 | 0.022599106 |
| SNORD114-1 | GULP1 | 0.3054 | 6.25E-10 | 1 | 1.083551569 | -1 | 1.032655214 | 1.136956447 | 0.001079164 |
| SNORD114-1 | ALDH1L2 | 0.4241 | 1.39E-18 | 1 | 1.083772372 | -1 | 1.044840881 | 1.124154477 | 1.63E-05 |
| SNORD114-1 | FLRT2 | 0.4039 | 7.46E-17 | 1 | 1.098051471 | -1 | 1.049632687 | 1.148703777 | 4.80E-05 |
| SNORD114-1 | FAT4 | 0.3314 | 1.57E-11 | 1 | 1.101079344 | -1 | 1.000246121 | 1.212077404 | 0.049415869 |
| SNORD114-1 | SGTB | 0.3312 | 1.63E-11 | 1 | 1.111538133 | -1 | 1.048904051 | 1.177912336 | 0.000352314 |
| SNORD114-1 | ABCC9 | 0.3404 | 4.04E-12 | 1 | 1.128437867 | -1 | 1.063917583 | 1.196870923 | 5.76E-05 |
| SNORD114-1 | FAM180A | 0.3528 | 5.84E-13 | 1 | 1.13783635 | -1 | 1.056095936 | 1.225903362 | 0.000686564 |
| SNORD114-1 | TMEM26 | 0.3138 | 1.99E-10 | 1 | 1.158222364 | -1 | 1.035615573 | 1.295344606 | 0.010082621 |
| SNORD114-1 | PCDHGA12 | 0.3252 | 3.95E-11 | 1 | 1.255316837 | -1 | 1.034506786 | 1.523257636 | 0.021242084 |
| SNORD19B | TSGA10 | 0.3182 | 1.07E-10 | 1 | 0.861527304 | 1 | 0.747346265 | 0.993153147 | 0.039910473 |
| SNORD19B | SLC23A3 | 0.3081 | 4.33E-10 | 1 | 0.875956486 | 1 | 0.785388699 | 0.976968176 | 0.017386922 |
| SNORD19B | ZNF799 | 0.342 | 3.20E-12 | 1 | 0.895028688 | 1 | 0.814885502 | 0.983053878 | 0.020500515 |
| SNORD19B | LRFN2 | 0.3717 | 2.53E-14 | 1 | 0.910854935 | 1 | 0.832029075 | 0.997148703 | 0.043197867 |
| SNORD19B | CCDC125 | 0.307 | 5.08E-10 | 1 | 0.934524175 | 1 | 0.88564058 | 0.986105936 | 0.013496921 |
| SNORD19B | C3orf62 | 0.3238 | 4.81E-11 | 1 | 0.943544566 | 1 | 0.907716732 | 0.980786533 | 0.00325873 |
| SNORD19B | SLC22A5 | 0.3535 | 5.18E-13 | 1 | 0.944339487 | 1 | 0.894063785 | 0.997442332 | 0.040197744 |
| SNORD19B | ZNF443 | 0.3378 | 6.01E-12 | 1 | 0.947623334 | 1 | 0.908732277 | 0.988178814 | 0.011865044 |
| SNORD19B | PLA2G6 | 0.3133 | 2.14E-10 | 1 | 0.947662283 | 1 | 0.916554182 | 0.979826202 | 0.001595535 |
| SNORD19B | RALGPS1 | 0.3063 | 5.53E-10 | 1 | 0.947923073 | 1 | 0.899525679 | 0.998924404 | 0.045477301 |
| SNORD19B | GRAMD1C | 0.303 | 8.71E-10 | 1 | 0.957014679 | 1 | 0.917188129 | 0.998570596 | 0.042772863 |
| SNORD19B | ZNF254 | 0.3035 | 8.06E-10 | 1 | 0.960355853 | 1 | 0.930049579 | 0.991649676 | 0.013417091 |
| SNORD19B | NSUN5P1 | 0.3103 | 3.23E-10 | 1 | 0.960912376 | 1 | 0.93421853 | 0.988368958 | 0.005539389 |
| SNORD19B | PLEKHH1 | 0.3786 | 7.63E-15 | 1 | 0.964067281 | 1 | 0.936978872 | 0.991938827 | 0.011850139 |
| SNORD19B | NBR2 | 0.3004 | 1.22E-09 | 1 | 0.964172917 | 1 | 0.941536303 | 0.987353766 | 0.002613298 |
| SNORD19B | SAMD12 | 0.32 | 8.26E-11 | 1 | 0.964281668 | 1 | 0.931122951 | 0.998621218 | 0.041625491 |
| SNORD19B | AOC2 | 0.3154 | 1.59E-10 | 1 | 0.965029484 | 1 | 0.936087054 | 0.994866772 | 0.021950598 |
| SNORD19B | NSUN6 | 0.3632 | 1.06E-13 | 1 | 0.966413405 | 1 | 0.945009171 | 0.98830244 | 0.002792973 |
| SNORD19B | NEIL1 | 0.3316 | 1.53E-11 | 1 | 0.96915919 | 1 | 0.944494099 | 0.994468399 | 0.017233444 |
| SNORD19B | PLIN5 | 0.3957 | 3.49E-16 | 1 | 0.970703081 | 1 | 0.949186164 | 0.992707762 | 0.009324747 |
| SNORD19B | C1orf159 | 0.3129 | 2.26E-10 | 1 | 0.9715012 | 1 | 0.945684451 | 0.998022734 | 0.03537908 |
| SNORD19B | ACP6 | 0.3287 | 2.34E-11 | 1 | 0.972361658 | 1 | 0.952027134 | 0.99313051 | 0.009343332 |
| SNORD19B | TMC7 | 0.3003 | 1.24E-09 | 1 | 0.973477854 | 1 | 0.956038958 | 0.991234849 | 0.003562256 |
| SNORD19B | PDE10A | 0.3042 | 7.42E-10 | 1 | 0.973969159 | 1 | 0.94863625 | 0.999978573 | 0.049814017 |
| SNORD19B | MFAP3L | 0.359 | 2.12E-13 | 1 | 0.974043857 | 1 | 0.954932454 | 0.993537742 | 0.009289363 |
| SNORD19B | NPAS2 | 0.3448 | 2.06E-12 | 1 | 0.976208158 | 1 | 0.9601431 | 0.992542016 | 0.004452658 |
| SNORD19B | SETMAR | 0.3411 | 3.67E-12 | 1 | 0.97651451 | 1 | 0.958959218 | 0.99439118 | 0.01023911 |
| SNORD19B | GGA1 | 0.3048 | 6.80E-10 | 1 | 0.976523227 | 1 | 0.957455604 | 0.99597058 | 0.01821235 |
| SNORD19B | CCDC24 | 0.3047 | 6.89E-10 | 1 | 0.977533278 | 1 | 0.959758443 | 0.995637306 | 0.015226565 |
| SNORD19B | PGPEP1 | 0.3185 | 1.02E-10 | 1 | 0.977903957 | 1 | 0.958777863 | 0.997411586 | 0.026613446 |
| SNORD19B | OVGP1 | 0.3615 | 1.41E-13 | 1 | 0.978369561 | 1 | 0.957281622 | 0.999922045 | 0.049185958 |
| SNORD19B | LYPD6 | 0.3801 | 5.90E-15 | 1 | 0.978599893 | 1 | 0.960147869 | 0.997406526 | 0.02592433 |
| SNORD19B | ZSCAN16 | 0.3275 | 2.81E-11 | 1 | 0.979295205 | 1 | 0.965072935 | 0.99372707 | 0.005062663 |
| SNORD19B | ZNF823 | 0.3861 | 2.01E-15 | 1 | 0.979913186 | 1 | 0.96537492 | 0.994670394 | 0.007798519 |
| SNORD19B | HNF1B | 0.3135 | 2.06E-10 | 1 | 0.980657992 | 1 | 0.964799477 | 0.996777175 | 0.018873019 |
| SNORD19B | ZNF44 | 0.318 | 1.10E-10 | 1 | 0.981219527 | 1 | 0.964163309 | 0.998577473 | 0.034084166 |
| SNORD19B | DEGS2 | 0.325 | 4.04E-11 | 1 | 0.984089357 | 1 | 0.973319718 | 0.994978161 | 0.004281118 |
| SNORD19B | NADSYN1 | 0.3769 | 1.04E-14 | 1 | 0.98499812 | 1 | 0.973761626 | 0.996364274 | 0.009817659 |
| SNORD19B | SPIRE2 | 0.3907 | 8.73E-16 | 1 | 0.985339875 | 1 | 0.972101691 | 0.998758338 | 0.032355245 |
| SNORD19B | WBP1 | 0.3219 | 6.37E-11 | 1 | 0.985528183 | 1 | 0.972287186 | 0.998949502 | 0.034664229 |
| SNORD19B | SH3YL1 | 0.3607 | 1.62E-13 | 1 | 0.98619957 | 1 | 0.973621721 | 0.998939907 | 0.033844481 |
| SNORD19B | PCGF3 | 0.3041 | 7.48E-10 | 1 | 0.986490398 | 1 | 0.977002807 | 0.996070122 | 0.005805833 |
| SNORD19B | RBM6 | 0.3002 | 1.26E-09 | 1 | 0.987150993 | 1 | 0.977842309 | 0.996548293 | 0.007467633 |
| SNORD19B | PIK3C2B | 0.3832 | 3.43E-15 | 1 | 0.987600572 | 1 | 0.977134238 | 0.998179014 | 0.021718347 |
| SNORD19B | ELOVL6 | 0.4025 | 9.75E-17 | 1 | 0.987768289 | 1 | 0.977443707 | 0.998201929 | 0.021695218 |
| SNORD19B | ZNF440 | 0.3386 | 5.32E-12 | 1 | 0.988055582 | 1 | 0.976713969 | 0.999528894 | 0.041354266 |
| SNORD19B | PLEKHA6 | 0.3096 | 3.54E-10 | 1 | 0.9881113 | 1 | 0.978419872 | 0.997898722 | 0.017395281 |
| SNORD19B | STXBP2 | 0.3765 | 1.10E-14 | 1 | 0.988160444 | 1 | 0.978537567 | 0.997877952 | 0.017059222 |
| SNORD19B | PPFIBP2 | 0.399 | 1.88E-16 | 1 | 0.988705763 | 1 | 0.980597579 | 0.996880989 | 0.006861425 |
| SNORD19B | HIP1R | 0.3576 | 2.69E-13 | 1 | 0.988738633 | 1 | 0.979612303 | 0.997949987 | 0.016679474 |
| SNORD19B | IP6K2 | 0.4408 | 4.10E-20 | 1 | 0.98878383 | 1 | 0.981936108 | 0.995679306 | 0.001466821 |
| SNORD19B | ENGASE | 0.3577 | 2.66E-13 | 1 | 0.988993536 | 1 | 0.980915162 | 0.99713844 | 0.00817483 |
| SNORD19B | HSD17B2 | 0.3577 | 2.63E-13 | 1 | 0.989709344 | 1 | 0.981721272 | 0.997762414 | 0.012358394 |
| SNORD19B | ARHGEF10L | 0.336 | 7.89E-12 | 1 | 0.989971032 | 1 | 0.980257608 | 0.999780706 | 0.04511736 |
| SNORD19B | ZNF692 | 0.3019 | 1.00E-09 | 1 | 0.99020294 | 1 | 0.9809582 | 0.999534804 | 0.039668023 |
| SNORD19B | ZNF737 | 0.3245 | 4.35E-11 | 1 | 0.990394744 | 1 | 0.981647849 | 0.999219577 | 0.032969109 |
| SNORD19B | DENND2D | 0.3142 | 1.89E-10 | 1 | 0.990974228 | 1 | 0.98364912 | 0.998353885 | 0.016612232 |
| SNORD19B | CRB3 | 0.3732 | 1.95E-14 | 1 | 0.991625124 | 1 | 0.984759293 | 0.998538825 | 0.017670318 |
| SNORD19B | CYP4F12 | 0.3629 | 1.11E-13 | 1 | 0.9920628 | 1 | 0.985071641 | 0.999103576 | 0.027208552 |
| SNORD19B | FAAH | 0.3064 | 5.47E-10 | 1 | 0.992217205 | 1 | 0.985648383 | 0.998829805 | 0.021141046 |
| SNORD19B | TOX3 | 0.4019 | 1.09E-16 | 1 | 0.992908757 | 1 | 0.986229857 | 0.999632889 | 0.038772754 |
| SNORD19B | CHMP4C | 0.333 | 1.24E-11 | 1 | 0.993227636 | 1 | 0.98719262 | 0.999299545 | 0.028866949 |
| SNORD19B | PLXNB1 | 0.4136 | 1.14E-17 | 1 | 0.993373703 | 1 | 0.988296001 | 0.998477494 | 0.011000002 |
| SNORD19B | CD2AP | 0.3168 | 1.30E-10 | 1 | 0.993668124 | 1 | 0.98822553 | 0.999140693 | 0.023405904 |
| SNORD19B | CARD11 | 0.3109 | 2.98E-10 | 1 | 0.993997031 | 1 | 0.989675624 | 0.998337307 | 0.006758004 |
| SNORD19B | DNM2 | 0.3038 | 7.76E-10 | 1 | 0.994240733 | 1 | 0.988712165 | 0.999800215 | 0.042336812 |
| SNORD19B | BCAS1 | 0.3212 | 7.03E-11 | 1 | 0.994580023 | 1 | 0.989790398 | 0.999392826 | 0.02734491 |
| SNORD19B | SLC44A3 | 0.3714 | 2.65E-14 | 1 | 0.994614232 | 1 | 0.989604059 | 0.999649771 | 0.036089726 |
| SNORD19B | CAPN5 | 0.3474 | 1.38E-12 | 1 | 0.994798904 | 1 | 0.990258257 | 0.999360372 | 0.025477636 |
| SNORD19B | STAP2 | 0.3068 | 5.21E-10 | 1 | 0.994914909 | 1 | 0.991480817 | 0.998360895 | 0.003854132 |
| SNORD19B | GOLT1A | 0.3789 | 7.31E-15 | 1 | 0.994992803 | 1 | 0.990686636 | 0.999317688 | 0.02330475 |
| SNORD19B | SH3GLB2 | 0.3753 | 1.36E-14 | 1 | 0.995584954 | 1 | 0.99198605 | 0.999196915 | 0.016630309 |
| SNORD19B | FOXA1 | 0.3273 | 2.88E-11 | 1 | 0.996107592 | 1 | 0.99318062 | 0.99904319 | 0.009389486 |
| SNORD19B | BTBD16 | 0.3369 | 6.93E-12 | 1 | 0.996333092 | 1 | 0.993717292 | 0.998955779 | 0.00616468 |
| SNORD19B | NR2F6 | 0.3546 | 4.33E-13 | 1 | 0.996378288 | 1 | 0.993237536 | 0.999528972 | 0.024293953 |
| SNORD19B | FAM3B | 0.3303 | 1.86E-11 | 1 | 0.996625094 | 1 | 0.993270497 | 0.999991021 | 0.049392959 |
| SNORD19B | ORMDL3 | 0.3974 | 2.53E-16 | 1 | 0.996703914 | 1 | 0.993932764 | 0.999482791 | 0.020117224 |
| SNORD19B | ALOX5 | 0.3043 | 7.28E-10 | 1 | 0.99684762 | 1 | 0.994139209 | 0.999563409 | 0.022932871 |
| SNORD19B | FER1L4 | 0.4202 | 3.04E-18 | 1 | 0.997168122 | 1 | 0.995150387 | 0.999189948 | 0.006067375 |
| SNORD19B | SRC | 0.3424 | 2.99E-12 | 1 | 0.997294245 | 1 | 0.994790397 | 0.999804395 | 0.034644529 |
| SNORD19B | TBX3 | 0.3881 | 1.42E-15 | 1 | 0.997406421 | 1 | 0.995544398 | 0.999271926 | 0.006451396 |
| SNORD19B | PPARG | 0.4294 | 4.61E-19 | 1 | 0.997806053 | 1 | 0.995850851 | 0.999765094 | 0.028183524 |
| SNORD19B | CAPS | 0.3276 | 2.75E-11 | 1 | 0.997923826 | 1 | 0.995957072 | 0.999894463 | 0.038940018 |
| SNORD19B | PSMB9 | -0.3045 | 7.10E-10 | -1 | 0.997973894 | 1 | 0.996164788 | 0.999786285 | 0.028462913 |
| SNORD19B | ACSL5 | 0.3234 | 5.07E-11 | 1 | 0.997992061 | 1 | 0.996205542 | 0.999781784 | 0.027899437 |
| SNORD19B | SSH3 | 0.3495 | 9.84E-13 | 1 | 0.998302362 | 1 | 0.996711648 | 0.999895616 | 0.036773971 |
| SNORD19B | FBP1 | 0.3908 | 8.66E-16 | 1 | 0.998504597 | 1 | 0.997418799 | 0.999591578 | 0.007020932 |
| SNORD19B | IDH1 | 0.3577 | 2.65E-13 | 1 | 0.99880503 | 1 | 0.997721681 | 0.999889556 | 0.03081598 |
| SNORD19B | ELF3 | 0.3277 | 2.72E-11 | 1 | 0.998965548 | 1 | 0.997936179 | 0.999995979 | 0.049113091 |
| SNORD19B | GATA3 | 0.4004 | 1.46E-16 | 1 | 0.999088972 | 1 | 0.998339109 | 0.999839399 | 0.017348788 |
| SNORD19B | VSIG2 | 0.3836 | 3.18E-15 | 1 | 0.999233242 | 1 | 0.998568167 | 0.99989876 | 0.023945486 |
| SNORD19B | HPGD | 0.3489 | 1.08E-12 | 1 | 0.999251685 | 1 | 0.998505179 | 0.999998749 | 0.049617845 |
| SNORD19B | S100P | 0.3199 | 8.42E-11 | 1 | 0.999894192 | 1 | 0.999790057 | 0.999998338 | 0.046454596 |
| SNORD19B | COL1A1 | -0.3712 | 2.79E-14 | -1 | 1.000069718 | -1 | 1.000015973 | 1.000123466 | 0.011007254 |
| SNORD19B | COL3A1 | -0.3648 | 8.19E-14 | -1 | 1.000078169 | -1 | 1.000011086 | 1.000145256 | 0.022378028 |
| SNORD19B | MMP9 | -0.3346 | 9.87E-12 | -1 | 1.000079629 | -1 | 1.000021742 | 1.000137519 | 0.007014473 |
| SNORD19B | COL1A2 | -0.3591 | 2.09E-13 | -1 | 1.000148497 | -1 | 1.000038427 | 1.00025858 | 0.00818701 |
| SNORD19B | SPARC | -0.3451 | 1.98E-12 | -1 | 1.000175584 | -1 | 1.000047509 | 1.000303676 | 0.007208304 |
| SNORD19B | LGALS1 | -0.3556 | 3.69E-13 | -1 | 1.000197448 | -1 | 1.000077305 | 1.000317605 | 0.001276501 |
| SNORD19B | BGN | -0.3461 | 1.68E-12 | -1 | 1.000220423 | -1 | 1.000040002 | 1.000400876 | 0.016639878 |
| SNORD19B | VIM | -0.3697 | 3.57E-14 | -1 | 1.000274 | -1 | 1.000081691 | 1.000466346 | 0.005227817 |
| SNORD19B | COL6A1 | -0.4193 | 3.62E-18 | -1 | 1.000284727 | -1 | 1.000055672 | 1.000513833 | 0.014833984 |
| SNORD19B | LUM | -0.3216 | 6.64E-11 | -1 | 1.000341435 | -1 | 1.000064347 | 1.0006186 | 0.015726709 |
| SNORD19B | CTSK | -0.346 | 1.71E-12 | -1 | 1.000347461 | -1 | 1.000102254 | 1.000592729 | 0.005479058 |
| SNORD19B | CES1 | -0.3769 | 1.04E-14 | -1 | 1.000359448 | -1 | 1.000093155 | 1.000625812 | 0.008151532 |
| SNORD19B | FN1 | -0.3646 | 8.48E-14 | -1 | 1.000398967 | -1 | 1.000214583 | 1.000583385 | 2.22E-05 |
| SNORD19B | COMP | -0.3093 | 3.69E-10 | -1 | 1.000404967 | -1 | 1.00001741 | 1.000792674 | 0.04055705 |
| SNORD19B | MMP14 | -0.3085 | 4.10E-10 | -1 | 1.000425549 | -1 | 1.000039045 | 1.000812201 | 0.030927414 |
| SNORD19B | IGFBP6 | -0.3383 | 5.64E-12 | -1 | 1.000439001 | -1 | 1.000017656 | 1.000860523 | 0.041140225 |
| SNORD19B | GZMA | -0.3005 | 1.21E-09 | -1 | 1.000465834 | -1 | 1.00000483 | 1.00092705 | 0.047646899 |
| SNORD19B | UPK3A | 0.3082 | 4.28E-10 | 1 | 1.000493765 | -1 | 1.000186386 | 1.000801239 | 0.001639841 |
| SNORD19B | CTGF | -0.3456 | 1.81E-12 | -1 | 1.000535855 | -1 | 1.000004875 | 1.001067117 | 0.047932992 |
| SNORD19B | AEBP1 | -0.4075 | 3.76E-17 | -1 | 1.000594119 | -1 | 1.000119758 | 1.001068705 | 0.014091262 |
| SNORD19B | TGFBI | -0.4426 | 2.75E-20 | -1 | 1.000611752 | -1 | 1.000147722 | 1.001075997 | 0.009763254 |
| SNORD19B | ANXA1 | -0.3049 | 6.69E-10 | -1 | 1.000652496 | -1 | 1.000335463 | 1.000969629 | 5.47E-05 |
| SNORD19B | ISLR | -0.3815 | 4.64E-15 | -1 | 1.00070913 | -1 | 1.000181785 | 1.001236754 | 0.008393017 |
| SNORD19B | RBP1 | -0.3298 | 2.01E-11 | -1 | 1.000714905 | -1 | 1.000005243 | 1.001425071 | 0.048330595 |
| SNORD19B | TUBA1A | -0.3428 | 2.80E-12 | -1 | 1.000718114 | -1 | 1.000259321 | 1.001177118 | 0.00215368 |
| SNORD19B | GRB7 | 0.3417 | 3.35E-12 | 1 | 1.000769465 | -1 | 1.000155238 | 1.001384068 | 0.014068459 |
| SNORD19B | POSTN | -0.3844 | 2.74E-15 | -1 | 1.000818864 | -1 | 1.000124785 | 1.001513425 | 0.020751298 |
| SNORD19B | CPXM1 | -0.3549 | 4.15E-13 | -1 | 1.000819904 | -1 | 1.000128426 | 1.001511861 | 0.020118534 |
| SNORD19B | ANXA2 | -0.3697 | 3.55E-14 | -1 | 1.00082985 | -1 | 1.000303703 | 1.001356274 | 0.001989688 |
| SNORD19B | TIMP2 | -0.3954 | 3.72E-16 | -1 | 1.000856295 | -1 | 1.000116092 | 1.001597045 | 0.023360066 |
| SNORD19B | HTRA1 | -0.3902 | 9.70E-16 | -1 | 1.000943158 | -1 | 1.000357176 | 1.001529484 | 0.00160393 |
| SNORD19B | SFRP4 | -0.3719 | 2.46E-14 | -1 | 1.000946581 | -1 | 1.000137484 | 1.001756332 | 0.02183854 |
| SNORD19B | THBS1 | -0.3343 | 1.03E-11 | -1 | 1.000984444 | -1 | 1.000020005 | 1.001949813 | 0.045431382 |
| SNORD19B | EFEMP1 | -0.4012 | 1.24E-16 | -1 | 1.001042204 | -1 | 1.000131664 | 1.001953574 | 0.024863226 |
| SNORD19B | COL5A1 | -0.3704 | 3.16E-14 | -1 | 1.001101528 | -1 | 1.000368188 | 1.001835406 | 0.003234319 |
| SNORD19B | CD248 | -0.3038 | 7.79E-10 | -1 | 1.001173103 | -1 | 1.000160253 | 1.00218698 | 0.023192395 |
| SNORD19B | TM4SF1 | -0.31 | 3.34E-10 | -1 | 1.001183049 | -1 | 1.000549511 | 1.001816988 | 0.00025125 |
| SNORD19B | CTHRC1 | -0.4064 | 4.61E-17 | -1 | 1.001199033 | -1 | 1.00020497 | 1.002194084 | 0.018062272 |
| SNORD19B | COL6A3 | -0.3705 | 3.14E-14 | -1 | 1.001311415 | -1 | 1.000320688 | 1.002303123 | 0.009464593 |
| SNORD19B | ZYX | -0.3792 | 6.88E-15 | -1 | 1.001318829 | -1 | 1.000034808 | 1.002604499 | 0.044100879 |
| SNORD19B | COL5A2 | -0.3769 | 1.03E-14 | -1 | 1.001364635 | -1 | 1.000489469 | 1.002240567 | 0.002236437 |
| SNORD19B | UCHL1 | -0.3421 | 3.13E-12 | -1 | 1.001371497 | -1 | 1.000580559 | 1.00216306 | 0.00067487 |
| SNORD19B | ANXA5 | -0.4697 | 5.92E-23 | -1 | 1.001410558 | -1 | 1.000592849 | 1.002228935 | 0.000719697 |
| SNORD19B | DCN | -0.3719 | 2.44E-14 | -1 | 1.001422659 | -1 | 1.000168141 | 1.00267875 | 0.026226486 |
| SNORD19B | EMILIN1 | -0.3663 | 6.32E-14 | -1 | 1.001431989 | -1 | 1.000062854 | 1.002802999 | 0.040363822 |
| SNORD19B | SERPINF1 | -0.3047 | 6.90E-10 | -1 | 1.001495138 | -1 | 1.000512751 | 1.00247849 | 0.002847673 |
| SNORD19B | RGS2 | -0.3591 | 2.09E-13 | -1 | 1.001627564 | -1 | 1.000262379 | 1.002994613 | 0.01944119 |
| SNORD19B | DEGS1 | -0.4249 | 1.18E-18 | -1 | 1.001654105 | -1 | 1.000170763 | 1.003139648 | 0.028831559 |
| SNORD19B | FSTL1 | -0.3342 | 1.03E-11 | -1 | 1.001666146 | -1 | 1.000170982 | 1.003163544 | 0.028941262 |
| SNORD19B | TUBB6 | -0.4022 | 1.04E-16 | -1 | 1.001751412 | -1 | 1.000187186 | 1.003318085 | 0.028184225 |
| SNORD19B | CLIC4 | -0.4835 | 2.04E-24 | -1 | 1.001771503 | -1 | 1.000207711 | 1.003337741 | 0.02638299 |
| SNORD19B | F13A1 | -0.3351 | 9.13E-12 | -1 | 1.001773974 | -1 | 1.000019765 | 1.00353126 | 0.047471766 |
| SNORD19B | DPYSL3 | -0.4127 | 1.35E-17 | -1 | 1.001812992 | -1 | 1.000313411 | 1.003314822 | 0.017789704 |
| SNORD19B | TNC | -0.4681 | 8.51E-23 | -1 | 1.001874973 | -1 | 1.000608224 | 1.003143326 | 0.003708906 |
| SNORD19B | PTHLH | -0.3042 | 7.36E-10 | -1 | 1.001885808 | -1 | 1.000690663 | 1.003082381 | 0.001976938 |
| SNORD19B | PYGB | -0.3503 | 8.60E-13 | -1 | 1.00189071 | -1 | 1.000476091 | 1.003307329 | 0.008787446 |
| SNORD19B | OLFML3 | -0.4358 | 1.19E-19 | -1 | 1.001932997 | -1 | 1.000065016 | 1.003804469 | 0.042535054 |
| SNORD19B | GDPD3 | 0.3098 | 3.44E-10 | 1 | 1.002014383 | -1 | 1.000429898 | 1.003601378 | 0.012692932 |
| SNORD19B | GSN | -0.3542 | 4.67E-13 | -1 | 1.002078317 | -1 | 1.000738915 | 1.003419512 | 0.002347388 |
| SNORD19B | MYADM | -0.3606 | 1.64E-13 | -1 | 1.00220069 | -1 | 1.000519045 | 1.003885162 | 0.01030041 |
| SNORD19B | REEP6 | 0.3798 | 6.20E-15 | 1 | 1.002252025 | -1 | 1.000835212 | 1.003670844 | 0.001829104 |
| SNORD19B | ECM1 | -0.3856 | 2.24E-15 | -1 | 1.002275755 | -1 | 1.000787026 | 1.003766698 | 0.00272391 |
| SNORD19B | SULF2 | -0.3889 | 1.23E-15 | -1 | 1.002276044 | -1 | 1.000830843 | 1.003723332 | 0.002014913 |
| SNORD19B | NFE2L1 | -0.3071 | 4.98E-10 | -1 | 1.002340296 | -1 | 1.000427037 | 1.004257214 | 0.016487698 |
| SNORD19B | THY1 | -0.3155 | 1.57E-10 | -1 | 1.002449433 | -1 | 1.000591368 | 1.004310949 | 0.009751286 |
| SNORD19B | MXRA5 | -0.3013 | 1.08E-09 | -1 | 1.002499336 | -1 | 1.000309082 | 1.004694386 | 0.025293315 |
| SNORD19B | ANXA6 | -0.4403 | 4.53E-20 | -1 | 1.002522529 | -1 | 1.000249507 | 1.004800717 | 0.029601765 |
| SNORD19B | EMP3 | -0.434 | 1.76E-19 | -1 | 1.002524856 | -1 | 1.000848655 | 1.004203863 | 0.003141517 |
| SNORD19B | LRRC8A | -0.3163 | 1.40E-10 | -1 | 1.002545982 | -1 | 1.000234928 | 1.004862376 | 0.030814811 |
| SNORD19B | DPT | -0.4068 | 4.30E-17 | -1 | 1.002610769 | -1 | 1.000095484 | 1.005132379 | 0.041904781 |
| SNORD19B | PTGIS | -0.3069 | 5.12E-10 | -1 | 1.002853946 | -1 | 1.000000217 | 1.005715819 | 0.049982561 |
| SNORD19B | CXCL12 | -0.3432 | 2.65E-12 | -1 | 1.003031717 | -1 | 1.0010126 | 1.005054906 | 0.003235942 |
| SNORD19B | SMOC2 | -0.3043 | 7.25E-10 | -1 | 1.003067873 | -1 | 1.000215916 | 1.005927962 | 0.034981168 |
| SNORD19B | TPP1 | -0.3527 | 5.95E-13 | -1 | 1.003071111 | -1 | 1.000231517 | 1.005918766 | 0.034005358 |
| SNORD19B | CALU | -0.321 | 7.19E-11 | -1 | 1.00312716 | -1 | 1.001521692 | 1.004735202 | 0.000133151 |
| SNORD19B | SNAI2 | -0.3114 | 2.79E-10 | -1 | 1.003133212 | -1 | 1.001597175 | 1.004671606 | 6.30E-05 |
| SNORD19B | CCDC80 | -0.4516 | 3.84E-21 | -1 | 1.003184088 | -1 | 1.000364839 | 1.006011282 | 0.026828356 |
| SNORD19B | COL11A1 | -0.329 | 2.26E-11 | -1 | 1.003204197 | -1 | 1.000796703 | 1.005617482 | 0.009064642 |
| SNORD19B | COL18A1 | -0.342 | 3.20E-12 | -1 | 1.003462052 | -1 | 1.001036083 | 1.0058939 | 0.005134475 |
| SNORD19B | SPON2 | -0.4008 | 1.34E-16 | -1 | 1.003507903 | -1 | 1.000001226 | 1.007026876 | 0.049919811 |
| SNORD19B | OLFML2B | -0.3215 | 6.72E-11 | -1 | 1.003526527 | -1 | 1.00082148 | 1.006238884 | 0.010581524 |
| SNORD19B | LAMP2 | -0.319 | 9.56E-11 | -1 | 1.003668428 | -1 | 1.000871611 | 1.00647306 | 0.010114376 |
| SNORD19B | KLF10 | -0.3387 | 5.24E-12 | -1 | 1.003832937 | -1 | 1.000083666 | 1.007596264 | 0.045093123 |
| SNORD19B | MAP7D1 | -0.4348 | 1.49E-19 | -1 | 1.004004262 | -1 | 1.000896488 | 1.007121687 | 0.011521252 |
| SNORD19B | FAM129A | -0.4264 | 8.62E-19 | -1 | 1.004259506 | -1 | 1.000154438 | 1.008381423 | 0.041965975 |
| SNORD19B | APCDD1L | -0.439 | 6.06E-20 | -1 | 1.004279033 | -1 | 1.000009182 | 1.008567116 | 0.049508317 |
| SNORD19B | ITGA11 | -0.3348 | 9.52E-12 | -1 | 1.004323725 | -1 | 1.000105859 | 1.00855938 | 0.04450946 |
| SNORD19B | CORO1C | -0.4683 | 8.12E-23 | -1 | 1.004379489 | -1 | 1.000331155 | 1.008444206 | 0.033952073 |
| SNORD19B | PXDN | -0.3719 | 2.45E-14 | -1 | 1.004414984 | -1 | 1.001406387 | 1.007432621 | 0.003999754 |
| SNORD19B | CAPN2 | -0.433 | 2.16E-19 | -1 | 1.004439796 | -1 | 1.001439602 | 1.007448978 | 0.003701837 |
| SNORD19B | SRPX | -0.3869 | 1.77E-15 | -1 | 1.004455117 | -1 | 1.002346783 | 1.006567885 | 3.38E-05 |
| SNORD19B | EMP1 | -0.302 | 9.85E-10 | -1 | 1.004506263 | -1 | 1.002764417 | 1.006251134 | 3.82E-07 |
| SNORD19B | MFAP5 | -0.4147 | 9.19E-18 | -1 | 1.004541981 | -1 | 1.001402799 | 1.007691004 | 0.00454263 |
| SNORD19B | MARVELD1 | -0.3891 | 1.18E-15 | -1 | 1.004801702 | -1 | 1.00167614 | 1.007937016 | 0.00258209 |
| SNORD19B | PNMA1 | -0.4023 | 1.02E-16 | -1 | 1.004810572 | -1 | 1.000849694 | 1.008787125 | 0.017245619 |
| SNORD19B | PDGFRB | -0.3543 | 4.54E-13 | -1 | 1.004917066 | -1 | 1.002010771 | 1.007831791 | 0.000902339 |
| SNORD19B | FAM46B | -0.4224 | 1.95E-18 | -1 | 1.00530598 | -1 | 1.000100366 | 1.010538689 | 0.045732606 |
| SNORD19B | LRP1 | -0.4066 | 4.43E-17 | -1 | 1.005375837 | -1 | 1.001568691 | 1.009197455 | 0.005610574 |
| SNORD19B | MSC | -0.3697 | 3.57E-14 | -1 | 1.005440651 | -1 | 1.001943591 | 1.008949917 | 0.002271482 |
| SNORD19B | FIBIN | -0.4311 | 3.26E-19 | -1 | 1.005483698 | -1 | 1.000095012 | 1.010901419 | 0.046084749 |
| SNORD19B | SPHK1 | -0.436 | 1.16E-19 | -1 | 1.005505098 | -1 | 1.001032606 | 1.009997573 | 0.015790448 |
| SNORD19B | GAS1 | -0.4489 | 6.96E-21 | -1 | 1.005521175 | -1 | 1.001340688 | 1.009719114 | 0.009590332 |
| SNORD19B | NFIL3 | -0.389 | 1.19E-15 | -1 | 1.005670797 | -1 | 1.000685142 | 1.010681291 | 0.025742761 |
| SNORD19B | RNH1 | -0.3015 | 1.06E-09 | -1 | 1.005728444 | -1 | 1.000555366 | 1.010928267 | 0.02993253 |
| SNORD19B | HSPG2 | -0.3291 | 2.23E-11 | -1 | 1.005728538 | -1 | 1.001732822 | 1.009740192 | 0.004917741 |
| SNORD19B | CRISPLD2 | -0.3156 | 1.54E-10 | -1 | 1.005828473 | -1 | 1.001181009 | 1.010497511 | 0.013913936 |
| SNORD19B | COL16A1 | -0.4471 | 1.03E-20 | -1 | 1.005832915 | -1 | 1.001718496 | 1.009964235 | 0.005419526 |
| SNORD19B | OXCT1 | -0.3204 | 7.86E-11 | -1 | 1.005939872 | -1 | 1.00041287 | 1.011497409 | 0.035134173 |
| SNORD19B | TMEM158 | -0.3777 | 8.98E-15 | -1 | 1.005993058 | -1 | 1.00327708 | 1.008716387 | 1.48E-05 |
| SNORD19B | SLC2A3 | -0.3166 | 1.34E-10 | -1 | 1.006031275 | -1 | 1.002826112 | 1.009246682 | 0.000221327 |
| SNORD19B | ATP2B4 | -0.3095 | 3.61E-10 | -1 | 1.006220826 | -1 | 1.002123972 | 1.010334429 | 0.002889614 |
| SNORD19B | LRRC15 | -0.3689 | 4.07E-14 | -1 | 1.006228078 | -1 | 1.000994422 | 1.011489098 | 0.019620572 |
| SNORD19B | FHL3 | -0.3867 | 1.81E-15 | -1 | 1.006442169 | -1 | 1.001471478 | 1.011437531 | 0.011020741 |
| SNORD19B | ENPP1 | -0.341 | 3.70E-12 | -1 | 1.006489944 | -1 | 1.00244546 | 1.010550747 | 0.001638989 |
| SNORD19B | GPR68 | -0.3969 | 2.78E-16 | -1 | 1.006576087 | -1 | 1.000733801 | 1.012452481 | 0.027317751 |
| SNORD19B | PAM | -0.3904 | 9.27E-16 | -1 | 1.006736279 | -1 | 1.002780357 | 1.010707807 | 0.000831394 |
| SNORD19B | KITLG | -0.3735 | 1.87E-14 | -1 | 1.006773557 | -1 | 1.001325569 | 1.012251187 | 0.014749887 |
| SNORD19B | TGFB1I1 | -0.4218 | 2.21E-18 | -1 | 1.006795205 | -1 | 1.001063734 | 1.012559491 | 0.020074187 |
| SNORD19B | GLG1 | -0.3453 | 1.91E-12 | -1 | 1.006874722 | -1 | 1.00076956 | 1.013017128 | 0.027253556 |
| SNORD19B | FNDC1 | -0.3767 | 1.07E-14 | -1 | 1.006911911 | -1 | 1.000853549 | 1.013006947 | 0.025282956 |
| SNORD19B | CNTN1 | -0.4122 | 1.48E-17 | -1 | 1.006980005 | -1 | 1.00357237 | 1.01039921 | 5.77E-05 |
| SNORD19B | ANKH | -0.4035 | 8.09E-17 | -1 | 1.006987176 | -1 | 1.001391258 | 1.012614365 | 0.014327309 |
| SNORD19B | LRRC32 | -0.3142 | 1.89E-10 | -1 | 1.00701501 | -1 | 1.001385683 | 1.012675984 | 0.014521111 |
| SNORD19B | FAM20C | -0.4373 | 8.75E-20 | -1 | 1.007035826 | -1 | 1.002068345 | 1.012027931 | 0.005454086 |
| SNORD19B | MIAT | -0.3167 | 1.32E-10 | -1 | 1.007047413 | -1 | 1.000332131 | 1.013807775 | 0.03966285 |
| SNORD19B | ANGPTL1 | -0.3437 | 2.44E-12 | -1 | 1.007244289 | -1 | 1.00179894 | 1.012719237 | 0.009059391 |
| SNORD19B | GNG4 | -0.3075 | 4.72E-10 | -1 | 1.007400012 | -1 | 1.000616756 | 1.014229253 | 0.032449526 |
| SNORD19B | ME1 | -0.3188 | 9.90E-11 | -1 | 1.007490893 | -1 | 1.003327227 | 1.011671839 | 0.000412358 |
| SNORD19B | RAP1GAP | 0.3294 | 2.14E-11 | 1 | 1.007757677 | -1 | 1.001160546 | 1.014398279 | 0.021105603 |
| SNORD19B | GLIS2 | -0.3881 | 1.41E-15 | -1 | 1.007769055 | -1 | 1.000152247 | 1.015443869 | 0.045576725 |
| SNORD19B | WISP1 | -0.3743 | 1.61E-14 | -1 | 1.007839005 | -1 | 1.001547358 | 1.014170176 | 0.014530217 |
| SNORD19B | GFPT2 | -0.44 | 4.84E-20 | -1 | 1.007866619 | -1 | 1.002496357 | 1.01326565 | 0.00404505 |
| SNORD19B | BNC1 | -0.3083 | 4.22E-10 | -1 | 1.007889797 | -1 | 1.001513949 | 1.014306234 | 0.015216212 |
| SNORD19B | AHNAK2 | -0.5132 | 8.63E-28 | -1 | 1.008284758 | -1 | 1.003453461 | 1.013139315 | 0.000760571 |
| SNORD19B | DPYSL2 | -0.3671 | 5.52E-14 | -1 | 1.008296552 | -1 | 1.002951209 | 1.013670383 | 0.002314785 |
| SNORD19B | LHFP | -0.3078 | 4.50E-10 | -1 | 1.008322507 | -1 | 1.004347658 | 1.012313087 | 3.91E-05 |
| SNORD19B | ATP8B2 | -0.3842 | 2.84E-15 | -1 | 1.008623581 | -1 | 1.002755704 | 1.014525796 | 0.003921925 |
| SNORD19B | RBM24 | -0.3421 | 3.14E-12 | -1 | 1.008687508 | -1 | 1.00074962 | 1.016688358 | 0.031884357 |
| SNORD19B | TGFB3 | -0.4577 | 9.61E-22 | -1 | 1.008855499 | -1 | 1.001615323 | 1.01614801 | 0.016432038 |
| SNORD19B | HEG1 | -0.3714 | 2.68E-14 | -1 | 1.008994835 | -1 | 1.000729031 | 1.017328912 | 0.032875056 |
| SNORD19B | TWIST2 | -0.3848 | 2.54E-15 | -1 | 1.008996517 | -1 | 1.000415368 | 1.017651271 | 0.039853737 |
| SNORD19B | GPX8 | -0.4199 | 3.23E-18 | -1 | 1.00907949 | -1 | 1.001680373 | 1.016533262 | 0.016079864 |
| SNORD19B | COPZ2 | -0.4654 | 1.61E-22 | -1 | 1.009116916 | -1 | 1.004446429 | 1.01380912 | 0.000125877 |
| SNORD19B | ADAM12 | -0.3799 | 6.08E-15 | -1 | 1.009131107 | -1 | 1.001736365 | 1.016580437 | 0.015423381 |
| SNORD19B | KLF13 | -0.3638 | 9.65E-14 | -1 | 1.009239836 | -1 | 1.000137005 | 1.018425517 | 0.046635116 |
| SNORD19B | CDH11 | -0.3387 | 5.25E-12 | -1 | 1.009244457 | -1 | 1.001585393 | 1.01696209 | 0.01790679 |
| SNORD19B | KLF9 | -0.3414 | 3.49E-12 | -1 | 1.009372277 | -1 | 1.001766809 | 1.017035487 | 0.015631469 |
| SNORD19B | COL5A3 | -0.3416 | 3.38E-12 | -1 | 1.009501993 | -1 | 1.003816575 | 1.015219612 | 0.001031068 |
| SNORD19B | CERK | -0.374 | 1.71E-14 | -1 | 1.009556074 | -1 | 1.003048307 | 1.016106064 | 0.00394647 |
| SNORD19B | SLFN11 | -0.3577 | 2.62E-13 | -1 | 1.00964125 | -1 | 1.000068448 | 1.019305683 | 0.048376545 |
| SNORD19B | AMOTL1 | -0.3625 | 1.19E-13 | -1 | 1.009711497 | -1 | 1.001769975 | 1.017715975 | 0.016443954 |
| SNORD19B | SGCB | -0.4101 | 2.25E-17 | -1 | 1.009741398 | -1 | 1.000736963 | 1.018826853 | 0.033908663 |
| SNORD19B | KIRREL | -0.356 | 3.48E-13 | -1 | 1.009797896 | -1 | 1.000430799 | 1.019252698 | 0.04031128 |
| SNORD19B | WWC3 | -0.332 | 1.45E-11 | -1 | 1.009863241 | -1 | 1.003839097 | 1.015923536 | 0.001303692 |
| SNORD19B | LOX | -0.4341 | 1.71E-19 | -1 | 1.009933472 | -1 | 1.004064872 | 1.015836373 | 0.00088655 |
| SNORD19B | TRAM2 | -0.377 | 1.01E-14 | -1 | 1.009996415 | -1 | 1.001097384 | 1.018974552 | 0.02760443 |
| SNORD19B | TTC7A | -0.3173 | 1.22E-10 | -1 | 1.010016322 | -1 | 1.000700752 | 1.019418611 | 0.035019158 |
| SNORD19B | WWTR1 | -0.4396 | 5.33E-20 | -1 | 1.010062657 | -1 | 1.00171395 | 1.018480945 | 0.018061453 |
| SNORD19B | CUEDC1 | -0.3533 | 5.34E-13 | -1 | 1.010445701 | -1 | 1.000831255 | 1.020152509 | 0.033146538 |
| SNORD19B | TEAD4 | -0.3739 | 1.73E-14 | -1 | 1.010475191 | -1 | 1.004336024 | 1.016651884 | 0.000803759 |
| SNORD19B | FBN1 | -0.3907 | 8.86E-16 | -1 | 1.010620672 | -1 | 1.003721573 | 1.017567192 | 0.002504261 |
| SNORD19B | TWIST1 | -0.3224 | 5.87E-11 | -1 | 1.010715213 | -1 | 1.003365738 | 1.018118521 | 0.004205262 |
| SNORD19B | OGN | -0.3485 | 1.15E-12 | -1 | 1.010731615 | -1 | 1.00018996 | 1.021384376 | 0.045991472 |
| SNORD19B | PCOLCE2 | -0.3366 | 7.27E-12 | -1 | 1.010813212 | -1 | 1.003463294 | 1.018216965 | 0.003871042 |
| SNORD19B | SSFA2 | -0.311 | 2.94E-10 | -1 | 1.011073914 | -1 | 1.006279599 | 1.01589107 | 5.59E-06 |
| SNORD19B | ANXA2P2 | -0.3695 | 3.72E-14 | -1 | 1.011108992 | -1 | 1.003020896 | 1.019262309 | 0.007016393 |
| SNORD19B | NFIC | -0.3035 | 8.09E-10 | -1 | 1.01138415 | -1 | 1.002603384 | 1.020241818 | 0.010947563 |
| SNORD19B | LAYN | -0.3825 | 3.83E-15 | -1 | 1.011400886 | -1 | 1.000154656 | 1.022773575 | 0.046914602 |
| SNORD19B | LGI2 | -0.3076 | 4.63E-10 | -1 | 1.011401414 | -1 | 1.001085819 | 1.021823304 | 0.03020106 |
| SNORD19B | ATP6V1B2 | -0.3136 | 2.04E-10 | -1 | 1.01140965 | -1 | 1.002347432 | 1.0205538 | 0.01349043 |
| SNORD19B | CHST15 | -0.4415 | 3.55E-20 | -1 | 1.011555817 | -1 | 1.002018506 | 1.021183906 | 0.017446133 |
| SNORD19B | TPST1 | -0.3257 | 3.65E-11 | -1 | 1.011835522 | -1 | 1.006141786 | 1.017561479 | 4.38E-05 |
| SNORD19B | P4HA2 | -0.326 | 3.50E-11 | -1 | 1.011890063 | -1 | 1.003087307 | 1.020770068 | 0.008014933 |
| SNORD19B | UBLCP1 | -0.3442 | 2.24E-12 | -1 | 1.012149675 | -1 | 1.001599067 | 1.022811421 | 0.023894604 |
| SNORD19B | NRP2 | -0.4532 | 2.68E-21 | -1 | 1.012174485 | -1 | 1.002662959 | 1.02177624 | 0.012003863 |
| SNORD19B | NPC1 | -0.3339 | 1.08E-11 | -1 | 1.012850797 | -1 | 1.004160348 | 1.021616458 | 0.003681196 |
| SNORD19B | LRIG1 | -0.4144 | 9.63E-18 | -1 | 1.012855123 | -1 | 1.004040056 | 1.021747584 | 0.004183314 |
| SNORD19B | EGR2 | -0.3877 | 1.52E-15 | -1 | 1.013024159 | -1 | 1.003986282 | 1.022143394 | 0.004654112 |
| SNORD19B | FAM43A | -0.3064 | 5.49E-10 | -1 | 1.013073796 | -1 | 1.005809794 | 1.020390258 | 0.000403521 |
| SNORD19B | MAPRE2 | -0.3502 | 8.81E-13 | -1 | 1.013305144 | -1 | 1.003760034 | 1.022941022 | 0.006197222 |
| SNORD19B | ADAMTS14 | -0.4036 | 7.85E-17 | -1 | 1.013343436 | -1 | 1.003814737 | 1.022962586 | 0.005962441 |
| SNORD19B | TPD52L1 | -0.3195 | 8.87E-11 | -1 | 1.013630751 | -1 | 1.007593322 | 1.019704356 | 8.92E-06 |
| SNORD19B | CCDC8 | -0.3285 | 2.42E-11 | -1 | 1.013688562 | -1 | 1.004370517 | 1.023093056 | 0.003907497 |
| SNORD19B | GLT8D2 | -0.3798 | 6.26E-15 | -1 | 1.013953288 | -1 | 1.002843024 | 1.025186641 | 0.013701644 |
| SNORD19B | PDZRN3 | -0.3742 | 1.64E-14 | -1 | 1.013987992 | -1 | 1.001459669 | 1.026673046 | 0.028530659 |
| SNORD19B | ITPRIP | -0.4006 | 1.41E-16 | -1 | 1.014648799 | -1 | 1.003769164 | 1.025646356 | 0.008194806 |
| SNORD19B | MACF1 | -0.4021 | 1.06E-16 | -1 | 1.0147593 | -1 | 1.000238812 | 1.029490583 | 0.04632325 |
| SNORD19B | SLC24A3 | -0.3273 | 2.91E-11 | -1 | 1.015173864 | -1 | 1.001349254 | 1.029189338 | 0.031342282 |
| SNORD19B | PODNL1 | -0.3822 | 4.08E-15 | -1 | 1.015257607 | -1 | 1.001838296 | 1.028856665 | 0.025714293 |
| SNORD19B | KCNJ8 | -0.3631 | 1.08E-13 | -1 | 1.015615055 | -1 | 1.000604713 | 1.030850571 | 0.041396285 |
| SNORD19B | RECQL | -0.3101 | 3.29E-10 | -1 | 1.015622592 | -1 | 1.002881615 | 1.028525435 | 0.016097422 |
| SNORD19B | SEC23A | -0.3468 | 1.50E-12 | -1 | 1.015808858 | -1 | 1.003473516 | 1.028295833 | 0.011862115 |
| SNORD19B | NTRK2 | -0.3376 | 6.24E-12 | -1 | 1.015831803 | -1 | 1.004993824 | 1.026786659 | 0.004102352 |
| SNORD19B | SYDE1 | -0.3543 | 4.57E-13 | -1 | 1.015859251 | -1 | 1.00474569 | 1.02709574 | 0.005054847 |
| SNORD19B | DACT1 | -0.4091 | 2.76E-17 | -1 | 1.015889456 | -1 | 1.004810652 | 1.027090413 | 0.004835992 |
| SNORD19B | NR3C1 | -0.4228 | 1.80E-18 | -1 | 1.015932342 | -1 | 1.000581212 | 1.031518992 | 0.041875016 |
| SNORD19B | CD109 | -0.458 | 8.93E-22 | -1 | 1.016025706 | -1 | 1.009837031 | 1.022252308 | 3.39E-07 |
| SNORD19B | ACVR1 | -0.4312 | 3.14E-19 | -1 | 1.016026112 | -1 | 1.004073691 | 1.028120813 | 0.008455841 |
| SNORD19B | GNB4 | -0.4813 | 3.52E-24 | -1 | 1.016060405 | -1 | 1.000273735 | 1.032096224 | 0.046127881 |
| SNORD19B | ARSI | -0.4754 | 1.47E-23 | -1 | 1.0161697 | -1 | 1.006368769 | 1.026066081 | 0.001179334 |
| SNORD19B | MAP1B | -0.4028 | 9.25E-17 | -1 | 1.016179517 | -1 | 1.007622392 | 1.024809313 | 0.000199301 |
| SNORD19B | TRPS1 | -0.3756 | 1.31E-14 | -1 | 1.016287047 | -1 | 1.000713421 | 1.032103037 | 0.04031779 |
| SNORD19B | PDE5A | -0.3004 | 1.23E-09 | -1 | 1.016300708 | -1 | 1.007239422 | 1.02544351 | 0.000402301 |
| SNORD19B | MXRA7 | -0.4034 | 8.21E-17 | -1 | 1.016323806 | -1 | 1.008080583 | 1.024634435 | 9.74E-05 |
| SNORD19B | RAB3IL1 | -0.4271 | 7.44E-19 | -1 | 1.016473481 | -1 | 1.002627198 | 1.030510981 | 0.019548563 |
| SNORD19B | HGF | -0.3532 | 5.47E-13 | -1 | 1.016739451 | -1 | 1.003207048 | 1.030454395 | 0.015168482 |
| SNORD19B | GPR1 | -0.4244 | 1.30E-18 | -1 | 1.016750591 | -1 | 1.000639406 | 1.033121182 | 0.041510066 |
| SNORD19B | ERC1 | -0.3749 | 1.47E-14 | -1 | 1.016924235 | -1 | 1.004024082 | 1.029990135 | 0.009980269 |
| SNORD19B | COPS8 | -0.3309 | 1.70E-11 | -1 | 1.017398796 | -1 | 1.001677851 | 1.033366475 | 0.02993425 |
| SNORD19B | IKBIP | -0.3085 | 4.12E-10 | -1 | 1.017467419 | -1 | 1.0020811 | 1.033089985 | 0.025922571 |
| SNORD19B | PID1 | -0.3411 | 3.65E-12 | -1 | 1.017556078 | -1 | 1.000211727 | 1.035201191 | 0.047244786 |
| SNORD19B | NID2 | -0.3082 | 4.30E-10 | -1 | 1.017909 | -1 | 1.009169059 | 1.026724634 | 5.47E-05 |
| SNORD19B | NDE1 | -0.3316 | 1.53E-11 | -1 | 1.017923581 | -1 | 1.004274224 | 1.03175845 | 0.009903045 |
| SNORD19B | ZEB1 | -0.3683 | 4.51E-14 | -1 | 1.017965631 | -1 | 1.001220171 | 1.034991159 | 0.035373462 |
| SNORD19B | CSDC2 | -0.3964 | 3.09E-16 | -1 | 1.017982646 | -1 | 1.000720498 | 1.035542561 | 0.041101621 |
| SNORD19B | CDK6 | -0.3562 | 3.39E-13 | -1 | 1.018209956 | -1 | 1.006323191 | 1.030237127 | 0.002595107 |
| SNORD19B | ATP10D | -0.3062 | 5.62E-10 | -1 | 1.018443447 | -1 | 1.002412938 | 1.034730316 | 0.023964864 |
| SNORD19B | FAM69A | -0.4073 | 3.91E-17 | -1 | 1.019096044 | -1 | 1.003749661 | 1.034677058 | 0.014549309 |
| SNORD19B | PPFIBP1 | -0.3327 | 1.30E-11 | -1 | 1.019150392 | -1 | 1.003793113 | 1.034742626 | 0.014338254 |
| SNORD19B | CYTH3 | -0.3347 | 9.67E-12 | -1 | 1.019378209 | -1 | 1.0089373 | 1.029927164 | 0.000258337 |
| SNORD19B | SLC6A9 | -0.3143 | 1.86E-10 | -1 | 1.019597621 | -1 | 1.004467071 | 1.034956087 | 0.010950897 |
| SNORD19B | ARHGEF17 | -0.3604 | 1.69E-13 | -1 | 1.019639533 | -1 | 1.002224134 | 1.037357555 | 0.026916845 |
| SNORD19B | TNFAIP8L3 | -0.4294 | 4.57E-19 | -1 | 1.020055388 | -1 | 1.004535503 | 1.035815053 | 0.011134134 |
| SNORD19B | ISLR2 | -0.3005 | 1.20E-09 | -1 | 1.020073087 | -1 | 1.002838605 | 1.037603755 | 0.022253816 |
| SNORD19B | DMWD | -0.3156 | 1.55E-10 | -1 | 1.020141828 | -1 | 1.001301042 | 1.039337128 | 0.036023093 |
| SNORD19B | CACNA2D1 | -0.3821 | 4.17E-15 | -1 | 1.02054639 | -1 | 1.00438631 | 1.036966478 | 0.012510873 |
| SNORD19B | TBXAS1 | -0.3091 | 3.81E-10 | -1 | 1.021370853 | -1 | 1.008723766 | 1.034176505 | 0.000880098 |
| SNORD19B | CSGALNACT2 | -0.3818 | 4.36E-15 | -1 | 1.021514978 | -1 | 1.006159894 | 1.037104397 | 0.005875513 |
| SNORD19B | HOXD11 | -0.3017 | 1.03E-09 | -1 | 1.021654661 | -1 | 1.000936573 | 1.042801587 | 0.040411198 |
| SNORD19B | BAG2 | -0.4665 | 1.24E-22 | -1 | 1.021958226 | -1 | 1.002967236 | 1.041308808 | 0.023235162 |
| SNORD19B | BACE1 | -0.3809 | 5.08E-15 | -1 | 1.022021528 | -1 | 1.005294636 | 1.039026734 | 0.00967706 |
| SNORD19B | CDO1 | -0.3068 | 5.17E-10 | -1 | 1.022450709 | -1 | 1.003083705 | 1.042191642 | 0.022874337 |
| SNORD19B | STXBP1 | -0.3712 | 2.74E-14 | -1 | 1.022556373 | -1 | 1.007054825 | 1.038296536 | 0.004210255 |
| SNORD19B | TMCC2 | -0.3501 | 9.02E-13 | -1 | 1.022791097 | -1 | 1.009885762 | 1.035861349 | 0.000504502 |
| SNORD19B | ZNF532 | -0.4445 | 1.82E-20 | -1 | 1.022868802 | -1 | 1.005057379 | 1.040995876 | 0.011642108 |
| SNORD19B | KCNE4 | -0.3487 | 1.12E-12 | -1 | 1.022875931 | -1 | 1.006910368 | 1.039094644 | 0.004833136 |
| SNORD19B | ZNF469 | -0.4009 | 1.33E-16 | -1 | 1.023037438 | -1 | 1.003799612 | 1.042643956 | 0.018697359 |
| SNORD19B | DNAJB4 | -0.4113 | 1.78E-17 | -1 | 1.023334449 | -1 | 1.010047067 | 1.03679663 | 0.000541866 |
| SNORD19B | PDGFC | -0.424 | 1.42E-18 | -1 | 1.023385952 | -1 | 1.008500904 | 1.038490698 | 0.001985947 |
| SNORD19B | LATS2 | -0.3402 | 4.19E-12 | -1 | 1.023494331 | -1 | 1.002824297 | 1.044590411 | 0.025687728 |
| SNORD19B | CPNE8 | -0.3089 | 3.91E-10 | -1 | 1.023636991 | -1 | 1.005473956 | 1.042128127 | 0.010539624 |
| SNORD19B | CLIP4 | -0.367 | 5.66E-14 | -1 | 1.023660036 | -1 | 1.003838547 | 1.043872914 | 0.019078569 |
| SNORD19B | RASSF8 | -0.3516 | 7.08E-13 | -1 | 1.023668098 | -1 | 1.003227888 | 1.044524765 | 0.023018636 |
| SNORD19B | NCAM1 | -0.4233 | 1.60E-18 | -1 | 1.023897067 | -1 | 1.013773407 | 1.034121822 | 3.19E-06 |
| SNORD19B | SGMS2 | -0.3588 | 2.20E-13 | -1 | 1.023956668 | -1 | 1.001672441 | 1.046736653 | 0.034960581 |
| SNORD19B | SLC22A3 | -0.4539 | 2.30E-21 | -1 | 1.024050191 | -1 | 1.002929965 | 1.045615178 | 0.025409419 |
| SNORD19B | EVC | -0.3174 | 1.21E-10 | -1 | 1.024138725 | -1 | 1.003014771 | 1.04570756 | 0.024893765 |
| SNORD19B | SAV1 | -0.3668 | 5.87E-14 | -1 | 1.024724036 | -1 | 1.00519963 | 1.044627672 | 0.012833689 |
| SNORD19B | ADAMTS12 | -0.3608 | 1.59E-13 | -1 | 1.024952841 | -1 | 1.009044264 | 1.041112232 | 0.002014699 |
| SNORD19B | PLSCR4 | -0.4451 | 1.61E-20 | -1 | 1.025003698 | -1 | 1.002187281 | 1.048339569 | 0.031539945 |
| SNORD19B | GPR37 | -0.3467 | 1.53E-12 | -1 | 1.025218125 | -1 | 1.009957855 | 1.040708975 | 0.001134159 |
| SNORD19B | EDNRA | -0.3556 | 3.71E-13 | -1 | 1.025294447 | -1 | 1.011227797 | 1.039556771 | 0.000394038 |
| SNORD19B | TCHH | -0.3197 | 8.70E-11 | -1 | 1.025312274 | -1 | 1.004058504 | 1.047015941 | 0.019338445 |
| SNORD19B | ZNF521 | -0.3533 | 5.38E-13 | -1 | 1.026246924 | -1 | 1.000057698 | 1.053121986 | 0.049490855 |
| SNORD19B | PCDHGC3 | -0.3908 | 8.57E-16 | -1 | 1.02747654 | -1 | 1.012867916 | 1.042295865 | 0.000207303 |
| SNORD19B | ITGA1 | -0.3307 | 1.76E-11 | -1 | 1.027488354 | -1 | 1.008280188 | 1.047062444 | 0.004856483 |
| SNORD19B | P4HA3 | -0.4066 | 4.41E-17 | -1 | 1.027945298 | -1 | 1.004916686 | 1.051501632 | 0.017114521 |
| SNORD19B | LMO1 | -0.3613 | 1.45E-13 | -1 | 1.028157131 | -1 | 1.005127325 | 1.051714605 | 0.016286223 |
| SNORD19B | HS3ST3A1 | -0.4042 | 7.09E-17 | -1 | 1.028622855 | -1 | 1.004204875 | 1.053634575 | 0.021319276 |
| SNORD19B | GAS7 | -0.3494 | 9.95E-13 | -1 | 1.028801708 | -1 | 1.011444329 | 1.046456956 | 0.001072727 |
| SNORD19B | TCP11L1 | -0.3811 | 4.98E-15 | -1 | 1.029022625 | -1 | 1.004374282 | 1.054275864 | 0.020732881 |
| SNORD19B | NIN | -0.3428 | 2.80E-12 | -1 | 1.029891686 | -1 | 1.003703125 | 1.056763558 | 0.025011384 |
| SNORD19B | OMD | -0.4113 | 1.79E-17 | -1 | 1.02992045 | -1 | 1.004643296 | 1.055833585 | 0.020052937 |
| SNORD19B | GUCA1A | -0.3797 | 6.33E-15 | -1 | 1.030217867 | -1 | 1.007879036 | 1.053051821 | 0.007776241 |
| SNORD19B | MAP1A | -0.3714 | 2.69E-14 | -1 | 1.030633575 | -1 | 1.010629905 | 1.051033182 | 0.00255013 |
| SNORD19B | GXYLT2 | -0.4247 | 1.22E-18 | -1 | 1.030773216 | -1 | 1.009857544 | 1.052122082 | 0.003757758 |
| SNORD19B | NFATC1 | -0.3218 | 6.45E-11 | -1 | 1.031086304 | -1 | 1.002373261 | 1.060621833 | 0.033631037 |
| SNORD19B | PRR16 | -0.3478 | 1.29E-12 | -1 | 1.031268074 | -1 | 1.007512054 | 1.055584235 | 0.0096153 |
| SNORD19B | CCDC88A | -0.3584 | 2.34E-13 | -1 | 1.03143743 | -1 | 1.002005126 | 1.061734261 | 0.036119713 |
| SNORD19B | PTPRD | -0.3366 | 7.20E-12 | -1 | 1.031606701 | -1 | 1.001578233 | 1.062535456 | 0.038961373 |
| SNORD19B | EHBP1 | -0.3068 | 5.20E-10 | -1 | 1.032260217 | -1 | 1.019388337 | 1.04529463 | 7.07E-07 |
| SNORD19B | NUDT11 | -0.355 | 4.06E-13 | -1 | 1.032490134 | -1 | 1.014578582 | 1.050717899 | 0.000342369 |
| SNORD19B | MDGA1 | -0.379 | 7.20E-15 | -1 | 1.032579438 | -1 | 1.009898766 | 1.055769481 | 0.004666323 |
| SNORD19B | FAIM2 | -0.3184 | 1.04E-10 | -1 | 1.032933196 | -1 | 1.003320445 | 1.06341996 | 0.029011524 |
| SNORD19B | AGTR1 | -0.3107 | 3.03E-10 | -1 | 1.03306388 | -1 | 1.00246086 | 1.064601145 | 0.033992589 |
| SNORD19B | ARHGAP31 | -0.334 | 1.07E-11 | -1 | 1.033584387 | -1 | 1.006733448 | 1.061151476 | 0.013906648 |
| SNORD19B | C15orf59 | -0.3238 | 4.83E-11 | -1 | 1.034300384 | -1 | 1.00120317 | 1.068491707 | 0.042110284 |
| SNORD19B | PITPNM2 | -0.3039 | 7.68E-10 | -1 | 1.034781758 | -1 | 1.005482287 | 1.064935007 | 0.019646429 |
| SNORD19B | GRK5 | -0.3046 | 7.03E-10 | -1 | 1.035408677 | -1 | 1.021607714 | 1.049396078 | 3.73E-07 |
| SNORD19B | PLXDC1 | -0.3029 | 8.80E-10 | -1 | 1.035834185 | -1 | 1.005338494 | 1.067254924 | 0.020933819 |
| SNORD19B | B4GALNT1 | -0.368 | 4.74E-14 | -1 | 1.036184212 | -1 | 1.013138044 | 1.059754617 | 0.001952647 |
| SNORD19B | CYS1 | -0.3827 | 3.74E-15 | -1 | 1.036999535 | -1 | 1.007249551 | 1.067628211 | 0.014431305 |
| SNORD19B | SLIT2 | -0.358 | 2.49E-13 | -1 | 1.037081917 | -1 | 1.005345017 | 1.069820692 | 0.021668345 |
| SNORD19B | RASSF9 | -0.3924 | 6.45E-16 | -1 | 1.03754212 | -1 | 1.001855932 | 1.074499453 | 0.039036981 |
| SNORD19B | KATNAL1 | -0.4782 | 7.60E-24 | -1 | 1.038415958 | -1 | 1.002928011 | 1.075159623 | 0.033606625 |
| SNORD19B | KANK4 | -0.3562 | 3.38E-13 | -1 | 1.03846335 | -1 | 1.019580491 | 1.057695924 | 5.55E-05 |
| SNORD19B | GLIS1 | -0.3688 | 4.13E-14 | -1 | 1.039512498 | -1 | 1.002910024 | 1.077450826 | 0.034103565 |
| SNORD19B | ADAMTS16 | -0.3802 | 5.83E-15 | -1 | 1.03989703 | -1 | 1.013896201 | 1.066564636 | 0.00246029 |
| SNORD19B | EID3 | -0.4034 | 8.29E-17 | -1 | 1.04041858 | -1 | 1.001308442 | 1.081056321 | 0.042677621 |
| SNORD19B | TCF4 | -0.3593 | 2.02E-13 | -1 | 1.041731611 | -1 | 1.022267867 | 1.061565941 | 2.15E-05 |
| SNORD19B | SPSB4 | -0.3326 | 1.33E-11 | -1 | 1.041847931 | -1 | 1.022078116 | 1.06200015 | 2.74E-05 |
| SNORD19B | SACS | -0.3847 | 2.63E-15 | -1 | 1.041931761 | -1 | 1.00440665 | 1.080858828 | 0.028169659 |
| SNORD19B | GUCY1A3 | -0.3223 | 5.94E-11 | -1 | 1.042550856 | -1 | 1.011174057 | 1.074901279 | 0.007524906 |
| SNORD19B | SEMA3A | -0.3906 | 8.90E-16 | -1 | 1.044125942 | -1 | 1.00313427 | 1.086792681 | 0.0345917 |
| SNORD19B | HSPB2 | -0.3413 | 3.55E-12 | -1 | 1.045277968 | -1 | 1.003534793 | 1.088757497 | 0.03319967 |
| SNORD19B | ADCY9 | -0.3938 | 4.99E-16 | -1 | 1.045399955 | -1 | 1.010643823 | 1.081351353 | 0.010062053 |
| SNORD19B | ECM2 | -0.3533 | 5.38E-13 | -1 | 1.045658733 | -1 | 1.011229437 | 1.081260242 | 0.008957217 |
| SNORD19B | MYO5A | -0.3555 | 3.75E-13 | -1 | 1.045872503 | -1 | 1.020372951 | 1.072009298 | 0.000368867 |
| SNORD19B | UNC5C | -0.3081 | 4.36E-10 | -1 | 1.046325261 | -1 | 1.011411832 | 1.082443885 | 0.008914959 |
| SNORD19B | RGMA | -0.3757 | 1.27E-14 | -1 | 1.047081232 | -1 | 1.009547086 | 1.086010868 | 0.013506683 |
| SNORD19B | ZNF385B | -0.3396 | 4.61E-12 | -1 | 1.049168525 | -1 | 1.000222701 | 1.100509509 | 0.048941702 |
| SNORD19B | MAP7D3 | -0.3332 | 1.22E-11 | -1 | 1.049225105 | -1 | 1.019184165 | 1.080151516 | 0.00118669 |
| SNORD19B | RNF217 | -0.3407 | 3.90E-12 | -1 | 1.04984894 | -1 | 1.022790239 | 1.0776235 | 0.000260811 |
| SNORD19B | SGCD | -0.4034 | 8.26E-17 | -1 | 1.052210708 | -1 | 1.007025413 | 1.09942347 | 0.023051635 |
| SNORD19B | ARSB | -0.3608 | 1.59E-13 | -1 | 1.053360168 | -1 | 1.009287187 | 1.099357702 | 0.017131188 |
| SNORD19B | ADAMTSL1 | -0.3015 | 1.06E-09 | -1 | 1.053811409 | -1 | 1.019914031 | 1.088835385 | 0.001677915 |
| SNORD19B | PRKG1 | -0.355 | 4.09E-13 | -1 | 1.053910662 | -1 | 1.013964256 | 1.095430807 | 0.007735949 |
| SNORD19B | ZFHX4 | -0.4009 | 1.31E-16 | -1 | 1.055839873 | -1 | 1.001452456 | 1.113180991 | 0.044035361 |
| SNORD19B | PTGER3 | -0.3724 | 2.25E-14 | -1 | 1.058224385 | -1 | 1.017931811 | 1.100111851 | 0.004272546 |
| SNORD19B | HSPA12A | -0.3545 | 4.40E-13 | -1 | 1.058348705 | -1 | 1.003228712 | 1.116497133 | 0.037701354 |
| SNORD19B | CHSY3 | -0.398 | 2.29E-16 | -1 | 1.058417527 | -1 | 1.006430582 | 1.113089845 | 0.027146089 |
| SNORD19B | HPGDS | -0.3176 | 1.16E-10 | -1 | 1.061095722 | -1 | 1.010382681 | 1.114354148 | 0.017627511 |
| SNORD19B | FAM126A | -0.3606 | 1.64E-13 | -1 | 1.063242673 | -1 | 1.029141589 | 1.098473713 | 0.00022687 |
| SNORD19B | ADCY7 | -0.3797 | 6.35E-15 | -1 | 1.063786767 | -1 | 1.026356331 | 1.102582263 | 0.00071587 |
| SNORD19B | BMPER | -0.3255 | 3.76E-11 | -1 | 1.063999607 | -1 | 1.018603909 | 1.111418436 | 0.005294489 |
| SNORD19B | CCPG1 | -0.4093 | 2.62E-17 | -1 | 1.07052631 | -1 | 1.008888244 | 1.135930157 | 0.024295089 |
| SNORD19B | GLI2 | -0.4057 | 5.26E-17 | -1 | 1.072698017 | -1 | 1.030067772 | 1.117092551 | 0.000694428 |
| SNORD19B | SETBP1 | -0.3781 | 8.33E-15 | -1 | 1.073261141 | -1 | 1.035361145 | 1.112548489 | 0.000115994 |
| SNORD19B | RBMS3 | -0.4129 | 1.29E-17 | -1 | 1.073541091 | -1 | 1.020188103 | 1.129684292 | 0.006363342 |
| SNORD19B | OSBPL6 | -0.4287 | 5.33E-19 | -1 | 1.074239369 | -1 | 1.01799205 | 1.133594533 | 0.009058715 |
| SNORD19B | PDLIM2 | -0.3278 | 2.70E-11 | -1 | 1.077041372 | -1 | 1.032933196 | 1.123033051 | 0.00050379 |
| SNORD19B | STK32B | -0.3928 | 5.93E-16 | -1 | 1.077544258 | -1 | 1.029155729 | 1.128207903 | 0.001443008 |
| SNORD19B | ZHX3 | -0.3487 | 1.12E-12 | -1 | 1.077765058 | -1 | 1.035678784 | 1.121561567 | 0.000228745 |
| SNORD19B | CCL26 | -0.4233 | 1.61E-18 | -1 | 1.07867663 | -1 | 1.024165726 | 1.136088859 | 0.004203574 |
| SNORD19B | SVEP1 | -0.3012 | 1.10E-09 | -1 | 1.079799408 | -1 | 1.004932847 | 1.160243458 | 0.036243452 |
| SNORD19B | SLC30A4 | -0.3478 | 1.28E-12 | -1 | 1.080355603 | -1 | 1.010912302 | 1.154569221 | 0.022599106 |
| SNORD19B | KCNIP3 | -0.3806 | 5.40E-15 | -1 | 1.081665272 | -1 | 1.008391768 | 1.160263103 | 0.028273892 |
| SNORD19B | GULP1 | -0.3269 | 3.07E-11 | -1 | 1.083551569 | -1 | 1.032655214 | 1.136956447 | 0.001079164 |
| SNORD19B | ALDH1L2 | -0.3742 | 1.65E-14 | -1 | 1.083772372 | -1 | 1.044840881 | 1.124154477 | 1.63E-05 |
| SNORD19B | NTRK1 | -0.3558 | 3.59E-13 | -1 | 1.085000788 | -1 | 1.036893828 | 1.135339684 | 0.000422343 |
| SNORD19B | DYRK3 | -0.3804 | 5.62E-15 | -1 | 1.092387778 | -1 | 1.033527188 | 1.154600548 | 0.001766635 |
| SNORD19B | HDAC4 | -0.343 | 2.70E-12 | -1 | 1.096391926 | -1 | 1.031619944 | 1.165230725 | 0.003057139 |
| SNORD19B | FLRT2 | -0.3746 | 1.55E-14 | -1 | 1.098051471 | -1 | 1.049632687 | 1.148703777 | 4.80E-05 |
| SNORD19B | FAT4 | -0.3266 | 3.20E-11 | -1 | 1.101079344 | -1 | 1.000246121 | 1.212077404 | 0.049415869 |
| SNORD19B | SGTB | -0.439 | 5.99E-20 | -1 | 1.111538133 | -1 | 1.048904051 | 1.177912336 | 0.000352314 |
| SNORD19B | FGF1 | -0.3496 | 9.73E-13 | -1 | 1.122022051 | -1 | 1.053604862 | 1.194881998 | 0.000334923 |
| SNORD19B | ABCC9 | -0.3849 | 2.53E-15 | -1 | 1.128437867 | -1 | 1.063917583 | 1.196870923 | 5.76E-05 |
| SNORD19B | IGF1 | -0.3357 | 8.30E-12 | -1 | 1.133605582 | -1 | 1.051316647 | 1.222335458 | 0.001108278 |
| SNORD19B | KCNC4 | -0.3844 | 2.74E-15 | -1 | 1.135772714 | -1 | 1.03297127 | 1.248804972 | 0.008535544 |
| SNORD19B | FAM180A | -0.3885 | 1.31E-15 | -1 | 1.13783635 | -1 | 1.056095936 | 1.225903362 | 0.000686564 |
| SNORD19B | NAV3 | -0.4318 | 2.78E-19 | -1 | 1.146771233 | -1 | 1.044659701 | 1.25886378 | 0.003999622 |
| SNORD19B | WNT2B | -0.3243 | 4.46E-11 | -1 | 1.151669724 | -1 | 1.027369565 | 1.291008802 | 0.015378636 |
| SNORD19B | ADAMTS6 | -0.3805 | 5.52E-15 | -1 | 1.160398769 | -1 | 1.008045079 | 1.335778857 | 0.03830799 |
| SNORD19B | PLCL1 | -0.3166 | 1.35E-10 | -1 | 1.167292328 | -1 | 1.029752699 | 1.323202533 | 0.015592253 |
| SNORD19B | PCDHGA12 | -0.3259 | 3.53E-11 | -1 | 1.255316837 | -1 | 1.034506786 | 1.523257636 | 0.021242084 |
| U49A | SLC1A7 | -0.3341 | 1.06E-11 | -1 | 0.929946012 | 1 | 0.873990711 | 0.989483727 | 0.021798406 |
| U49A | CTSK | -0.3413 | 3.53E-12 | -1 | 1.000347461 | -1 | 1.000102254 | 1.000592729 | 0.005479058 |
| U49A | COMP | -0.3848 | 2.57E-15 | -1 | 1.000404967 | -1 | 1.00001741 | 1.000792674 | 0.04055705 |
| U49A | CTGF | -0.3169 | 1.29E-10 | -1 | 1.000535855 | -1 | 1.000004875 | 1.001067117 | 0.047932992 |
| U49A | AEBP1 | -0.361 | 1.54E-13 | -1 | 1.000594119 | -1 | 1.000119758 | 1.001068705 | 0.014091262 |
| U49A | ISLR | -0.3987 | 1.99E-16 | -1 | 1.00070913 | -1 | 1.000181785 | 1.001236754 | 0.008393017 |
| U49A | CPXM1 | -0.3508 | 8.04E-13 | -1 | 1.000819904 | -1 | 1.000128426 | 1.001511861 | 0.020118534 |
| U49A | TIMP2 | -0.3424 | 2.97E-12 | -1 | 1.000856295 | -1 | 1.000116092 | 1.001597045 | 0.023360066 |
| U49A | HTRA1 | -0.3184 | 1.04E-10 | -1 | 1.000943158 | -1 | 1.000357176 | 1.001529484 | 0.00160393 |
| U49A | SFRP4 | -0.4095 | 2.53E-17 | -1 | 1.000946581 | -1 | 1.000137484 | 1.001756332 | 0.02183854 |
| U49A | CD99 | -0.3034 | 8.26E-10 | -1 | 1.000971781 | -1 | 1.000028155 | 1.001916297 | 0.043542155 |
| U49A | NPM1 | 0.3443 | 2.23E-12 | 1 | 1.001167006 | -1 | 1.000387951 | 1.001946669 | 0.003318885 |
| U49A | DCN | -0.3799 | 6.12E-15 | -1 | 1.001422659 | -1 | 1.000168141 | 1.00267875 | 0.026226486 |
| U49A | EMILIN1 | -0.4236 | 1.52E-18 | -1 | 1.001431989 | -1 | 1.000062854 | 1.002802999 | 0.040363822 |
| U49A | SERPINF1 | -0.357 | 2.93E-13 | -1 | 1.001495138 | -1 | 1.000512751 | 1.00247849 | 0.002847673 |
| U49A | F13A1 | -0.3004 | 1.23E-09 | -1 | 1.001773974 | -1 | 1.000019765 | 1.00353126 | 0.047471766 |
| U49A | DPYSL3 | -0.3347 | 9.71E-12 | -1 | 1.001812992 | -1 | 1.000313411 | 1.003314822 | 0.017789704 |
| U49A | OLFML3 | -0.3792 | 6.93E-15 | -1 | 1.001932997 | -1 | 1.000065016 | 1.003804469 | 0.042535054 |
| U49A | ANXA6 | -0.3598 | 1.86E-13 | -1 | 1.002522529 | -1 | 1.000249507 | 1.004800717 | 0.029601765 |
| U49A | DPT | -0.3803 | 5.74E-15 | -1 | 1.002610769 | -1 | 1.000095484 | 1.005132379 | 0.041904781 |
| U49A | LAMB2 | -0.303 | 8.62E-10 | -1 | 1.002774226 | -1 | 1.000170311 | 1.005384921 | 0.036767674 |
| U49A | PTGIS | -0.4078 | 3.52E-17 | -1 | 1.002853946 | -1 | 1.000000217 | 1.005715819 | 0.049982561 |
| U49A | CXCL12 | -0.3477 | 1.32E-12 | -1 | 1.003031717 | -1 | 1.0010126 | 1.005054906 | 0.003235942 |
| U49A | SMOC2 | -0.4055 | 5.48E-17 | -1 | 1.003067873 | -1 | 1.000215916 | 1.005927962 | 0.034981168 |
| U49A | NOLC1 | 0.3408 | 3.84E-12 | 1 | 1.003153617 | -1 | 1.000014259 | 1.006302831 | 0.04896683 |
| U49A | CCDC80 | -0.3442 | 2.25E-12 | -1 | 1.003184088 | -1 | 1.000364839 | 1.006011282 | 0.026828356 |
| U49A | SPON2 | -0.3296 | 2.06E-11 | -1 | 1.003507903 | -1 | 1.000001226 | 1.007026876 | 0.049919811 |
| U49A | AXIN2 | -0.3182 | 1.07E-10 | -1 | 1.004893719 | -1 | 1.001209826 | 1.008591167 | 0.009181845 |
| U49A | PODN | -0.4524 | 3.20E-21 | -1 | 1.004898743 | -1 | 1.000491957 | 1.00932494 | 0.029309199 |
| U49A | ELN | -0.3932 | 5.51E-16 | -1 | 1.005225025 | -1 | 1.001679312 | 1.00878329 | 0.003844346 |
| U49A | FIBIN | -0.3705 | 3.13E-14 | -1 | 1.005483698 | -1 | 1.000095012 | 1.010901419 | 0.046084749 |
| U49A | GAS1 | -0.3531 | 5.55E-13 | -1 | 1.005521175 | -1 | 1.001340688 | 1.009719114 | 0.009590332 |
| U49A | CRISPLD2 | -0.3346 | 9.74E-12 | -1 | 1.005828473 | -1 | 1.001181009 | 1.010497511 | 0.013913936 |
| U49A | COL16A1 | -0.3622 | 1.26E-13 | -1 | 1.005832915 | -1 | 1.001718496 | 1.009964235 | 0.005419526 |
| U49A | COX7A1 | -0.4034 | 8.14E-17 | -1 | 1.0059047 | -1 | 1.0001388 | 1.011703841 | 0.04471937 |
| U49A | NDN | -0.3221 | 6.16E-11 | -1 | 1.005933171 | -1 | 1.000375898 | 1.011521314 | 0.036355367 |
| U49A | ABCE1 | 0.3286 | 2.37E-11 | 1 | 1.006728838 | -1 | 1.002555813 | 1.010919232 | 0.001554046 |
| U49A | TGFB1I1 | -0.3644 | 8.68E-14 | -1 | 1.006795205 | -1 | 1.001063734 | 1.012559491 | 0.020074187 |
| U49A | FNDC1 | -0.3282 | 2.55E-11 | -1 | 1.006911911 | -1 | 1.000853549 | 1.013006947 | 0.025282956 |
| U49A | SMPD1 | -0.3334 | 1.18E-11 | -1 | 1.006951982 | -1 | 1.000087615 | 1.013863465 | 0.047136976 |
| U49A | LRRC32 | -0.312 | 2.57E-10 | -1 | 1.00701501 | -1 | 1.001385683 | 1.012675984 | 0.014521111 |
| U49A | FAM20C | -0.3354 | 8.68E-12 | -1 | 1.007035826 | -1 | 1.002068345 | 1.012027931 | 0.005454086 |
| U49A | EFEMP2 | -0.3382 | 5.69E-12 | -1 | 1.007080759 | -1 | 1.002559483 | 1.011622425 | 0.00211623 |
| U49A | ANGPTL1 | -0.4053 | 5.73E-17 | -1 | 1.007244289 | -1 | 1.00179894 | 1.012719237 | 0.009059391 |
| U49A | GLIS2 | -0.3363 | 7.63E-12 | -1 | 1.007769055 | -1 | 1.000152247 | 1.015443869 | 0.045576725 |
| U49A | RBM24 | -0.3003 | 1.24E-09 | -1 | 1.008687508 | -1 | 1.00074962 | 1.016688358 | 0.031884357 |
| U49A | ANKRD35 | -0.3255 | 3.76E-11 | -1 | 1.008822944 | -1 | 1.001053432 | 1.016652758 | 0.025955512 |
| U49A | TGFB3 | -0.3099 | 3.42E-10 | -1 | 1.008855499 | -1 | 1.001615323 | 1.01614801 | 0.016432038 |
| U49A | COPZ2 | -0.3133 | 2.14E-10 | -1 | 1.009116916 | -1 | 1.004446429 | 1.01380912 | 0.000125877 |
| U49A | CDH11 | -0.3119 | 2.58E-10 | -1 | 1.009244457 | -1 | 1.001585393 | 1.01696209 | 0.01790679 |
| U49A | LRRC17 | -0.3223 | 5.99E-11 | -1 | 1.010295767 | -1 | 1.001460636 | 1.019208843 | 0.022274581 |
| U49A | CUEDC1 | -0.3068 | 5.19E-10 | -1 | 1.010445701 | -1 | 1.000831255 | 1.020152509 | 0.033146538 |
| U49A | KCNH2 | -0.3177 | 1.15E-10 | -1 | 1.010457919 | -1 | 1.005515127 | 1.015425009 | 3.21E-05 |
| U49A | OGN | -0.4306 | 3.57E-19 | -1 | 1.010731615 | -1 | 1.00018996 | 1.021384376 | 0.045991472 |
| U49A | CNPY4 | -0.3644 | 8.75E-14 | -1 | 1.010857411 | -1 | 1.002377002 | 1.019409567 | 0.011994554 |
| U49A | PDZRN3 | -0.4242 | 1.36E-18 | -1 | 1.013987992 | -1 | 1.001459669 | 1.026673046 | 0.028530659 |
| U49A | NFATC4 | -0.373 | 2.04E-14 | -1 | 1.014681472 | -1 | 1.00043936 | 1.029126332 | 0.043293182 |
| U49A | SLC24A3 | -0.3123 | 2.43E-10 | -1 | 1.015173864 | -1 | 1.001349254 | 1.029189338 | 0.031342282 |
| U49A | KCNJ8 | -0.342 | 3.17E-12 | -1 | 1.015615055 | -1 | 1.000604713 | 1.030850571 | 0.041396285 |
| U49A | SYDE1 | -0.3086 | 4.07E-10 | -1 | 1.015859251 | -1 | 1.00474569 | 1.02709574 | 0.005054847 |
| U49A | DACT1 | -0.3328 | 1.29E-11 | -1 | 1.015889456 | -1 | 1.004810652 | 1.027090413 | 0.004835992 |
| U49A | MXRA7 | -0.302 | 9.91E-10 | -1 | 1.016323806 | -1 | 1.008080583 | 1.024634435 | 9.74E-05 |
| U49A | GFM2 | 0.3084 | 4.19E-10 | 1 | 1.017359447 | -1 | 1.001108557 | 1.033874137 | 0.036186985 |
| U49A | NCOR1 | 0.3085 | 4.15E-10 | 1 | 1.017924605 | -1 | 1.001223111 | 1.034904698 | 0.035310325 |
| U49A | ZEB1 | -0.3016 | 1.04E-09 | -1 | 1.017965631 | -1 | 1.001220171 | 1.034991159 | 0.035373462 |
| U49A | CSDC2 | -0.4489 | 6.90E-21 | -1 | 1.017982646 | -1 | 1.000720498 | 1.035542561 | 0.041101621 |
| U49A | ISLR2 | -0.3098 | 3.44E-10 | -1 | 1.020073087 | -1 | 1.002838605 | 1.037603755 | 0.022253816 |
| U49A | DIRAS3 | -0.3224 | 5.86E-11 | -1 | 1.021611949 | -1 | 1.002748548 | 1.040830203 | 0.024536685 |
| U49A | TNXB | -0.3319 | 1.47E-11 | -1 | 1.021798677 | -1 | 1.004844414 | 1.039039002 | 0.011534652 |
| U49A | CDO1 | -0.3766 | 1.08E-14 | -1 | 1.022450709 | -1 | 1.003083705 | 1.042191642 | 0.022874337 |
| U49A | KCNE4 | -0.3463 | 1.62E-12 | -1 | 1.022875931 | -1 | 1.006910368 | 1.039094644 | 0.004833136 |
| U49A | NCAM1 | -0.3875 | 1.56E-15 | -1 | 1.023897067 | -1 | 1.013773407 | 1.034121822 | 3.19E-06 |
| U49A | HIF3A | -0.3496 | 9.68E-13 | -1 | 1.026079538 | -1 | 1.000438528 | 1.052377722 | 0.046160834 |
| U49A | ZNF521 | -0.3497 | 9.48E-13 | -1 | 1.026246924 | -1 | 1.000057698 | 1.053121986 | 0.049490855 |
| U49A | P4HA3 | -0.3348 | 9.57E-12 | -1 | 1.027945298 | -1 | 1.004916686 | 1.051501632 | 0.017114521 |
| U49A | TMOD1 | -0.3834 | 3.29E-15 | -1 | 1.028241237 | -1 | 1.004659807 | 1.052376172 | 0.018637572 |
| U49A | GAS7 | -0.324 | 4.67E-11 | -1 | 1.028801708 | -1 | 1.011444329 | 1.046456956 | 0.001072727 |
| U49A | ITGBL1 | -0.3163 | 1.39E-10 | -1 | 1.029588809 | -1 | 1.008184159 | 1.051447899 | 0.006520643 |
| U49A | OMD | -0.3954 | 3.71E-16 | -1 | 1.02992045 | -1 | 1.004643296 | 1.055833585 | 0.020052937 |
| U49A | TMEM100 | -0.3458 | 1.77E-12 | -1 | 1.030259648 | -1 | 1.000641086 | 1.060754907 | 0.045174554 |
| U49A | OLFML1 | -0.304 | 7.56E-10 | -1 | 1.030479979 | -1 | 1.003080182 | 1.058628219 | 0.028989072 |
| U49A | MAP1A | -0.3088 | 3.96E-10 | -1 | 1.030633575 | -1 | 1.010629905 | 1.051033182 | 0.00255013 |
| U49A | DCLK2 | -0.316 | 1.45E-10 | -1 | 1.03263195 | -1 | 1.0056534 | 1.060334251 | 0.017438162 |
| U49A | FAIM2 | -0.3303 | 1.85E-11 | -1 | 1.032933196 | -1 | 1.003320445 | 1.06341996 | 0.029011524 |
| U49A | AGTR1 | -0.3603 | 1.72E-13 | -1 | 1.03306388 | -1 | 1.00246086 | 1.064601145 | 0.033992589 |
| U49A | NXPH3 | -0.4364 | 1.06E-19 | -1 | 1.033541438 | -1 | 1.012894822 | 1.05460891 | 0.001353304 |
| U49A | C15orf59 | -0.335 | 9.18E-12 | -1 | 1.034300384 | -1 | 1.00120317 | 1.068491707 | 0.042110284 |
| U49A | HUNK | -0.3004 | 1.23E-09 | -1 | 1.036091795 | -1 | 1.008850892 | 1.064068254 | 0.009102331 |
| U49A | GPIHBP1 | -0.3558 | 3.61E-13 | -1 | 1.03632614 | -1 | 1.000841322 | 1.073069072 | 0.044720674 |
| U49A | CYS1 | -0.3765 | 1.10E-14 | -1 | 1.036999535 | -1 | 1.007249551 | 1.067628211 | 0.014431305 |
| U49A | ADAMTS16 | -0.3368 | 7.03E-12 | -1 | 1.03989703 | -1 | 1.013896201 | 1.066564636 | 0.00246029 |
| U49A | HSPB2 | -0.4135 | 1.16E-17 | -1 | 1.045277968 | -1 | 1.003534793 | 1.088757497 | 0.03319967 |
| U49A | SGCD | -0.3605 | 1.67E-13 | -1 | 1.052210708 | -1 | 1.007025413 | 1.09942347 | 0.023051635 |
| U49A | HHIPL1 | -0.3024 | 9.33E-10 | -1 | 1.05263659 | -1 | 1.002794623 | 1.104955854 | 0.038197907 |
| U49A | G2E3 | 0.3422 | 3.10E-12 | 1 | 1.057311908 | -1 | 1.009789094 | 1.107071245 | 0.017542553 |
| U49A | PTGER3 | -0.341 | 3.72E-12 | -1 | 1.058224385 | -1 | 1.017931811 | 1.100111851 | 0.004272546 |
| U49A | HPGDS | -0.325 | 4.05E-11 | -1 | 1.061095722 | -1 | 1.010382681 | 1.114354148 | 0.017627511 |
| U49A | PRND | -0.3086 | 4.07E-10 | -1 | 1.070003687 | -1 | 1.035203799 | 1.105973425 | 6.05E-05 |
| U49A | STK32B | -0.3061 | 5.70E-10 | -1 | 1.077544258 | -1 | 1.029155729 | 1.128207903 | 0.001443008 |
| U49A | CKMT2 | -0.3353 | 8.82E-12 | -1 | 1.084272987 | -1 | 1.025673652 | 1.14622025 | 0.004314412 |
| U49A | C1QTNF7 | -0.3013 | 1.08E-09 | -1 | 1.092707722 | -1 | 1.016225785 | 1.174945749 | 0.016633428 |
| U49A | SNED1 | -0.3618 | 1.35E-13 | -1 | 1.094734869 | -1 | 1.023627208 | 1.17078212 | 0.008254688 |
| U49A | IGF1 | -0.3811 | 4.95E-15 | -1 | 1.133605582 | -1 | 1.051316647 | 1.222335458 | 0.001108278 |
| U49A | FAM180A | -0.3283 | 2.51E-11 | -1 | 1.13783635 | -1 | 1.056095936 | 1.225903362 | 0.000686564 |
| U3 | TPM2 | -0.3149 | 1.70E-10 | -1 | 1.00030259 | -1 | 0.999924349 | 1.000680974 | 0.116905151 |
| U3 | LY6H | -0.3033 | 8.27E-10 | -1 | 0.99723006 | 1 | 0.947343792 | 1.049743293 | 0.915634053 |
