# Supplementary material for: Small nucleolar RNAs signature (SNORS) identified clinical outcome and prognosis of bladder cancer (BLCA)
Source: Cancer Cell Int. 2020 Jul 10;20:299. doi: 10.1186/s12935-020-01393-7 (PMC7350589; doi:10.1186/s12935-020-01393-7)
Supplement: Supplementary file 15 — Additional file 15: Table S13. Correlation between candidate snoRNAs and alternative splicing variants (AS) mRNA in TCGA-BLCA cohort. [file 12935_2020_1393_MOESM15_ESM.docx]

**Additional file 15: Table S13 Correlation between candidate snoRNAs and alternative splicing variants (AS) in TCGA-BLCA cohort (n = 392)**

| id | Gene Symbol | Splice Type | Exon | PSI Value | Coefficient | P-value |
| --- | --- | --- | --- | --- | --- | --- |
| SNORD19B | PALLD | Alternate Terminator | 26 | 0.0443 | -0.4765 | 1.47E-23 |
| SNORD19B | PALLD | Alternate Terminator | 10 | 0.0443 | 0.4765 | 1.49E-23 |
| SNORD19B | MXRA7 | Alternate Promoter | 3.1 | 0.1488 | 0.4575 | 1.27E-21 |
| SNORD19B | MXRA7 | Alternate Promoter | 1 | 0.1525 | -0.4475 | 1.20E-20 |
| SNORD19B | FBLN1 | Alternate Terminator | 22 | 0.1336 | -0.4431 | 3.09E-20 |
| SNORD19B | FBLN1 | Alternate Terminator | 17 | 0.1406 | 0.4415 | 4.38E-20 |
| SNORD19B | IFITM3 | Alternate Promoter | 3.1 | 0.078 | -0.4345 | 1.97E-19 |
| SNORD19B | IFITM3 | Alternate Promoter | 2 | 0.0779 | 0.4331 | 2.64E-19 |
| SNORD19B | AP1B1 | Exon Skip | 24 | 0.138 | -0.4305 | 4.54E-19 |
| SNORD19B | EXOC1 | Exon Skip | 11 | 0.2108 | 0.4263 | 2.68E-18 |
| SNORD19B | DACT3 | Alternate Terminator | 4.2 | 0.2496 | 0.4133 | 1.44E-17 |
| SNORD19B | DACT3 | Alternate Terminator | 5 | 0.2496 | -0.4133 | 1.44E-17 |
| SNORD19B | SLK | Exon Skip | 13 | 0.2312 | 0.413 | 1.71E-17 |
| SNORD19B | NCOR2 | Alternate Donor Sites | 46.2 | 0.1251 | 0.4096 | 2.97E-17 |
| SNORD19B | FILIP1L | Alternate Terminator | 5 | 0.0798 | 0.4096 | 3.00E-17 |
| SNORD19B | TUBB6 | Alternate Terminator | 5.5 | 0.1519 | -0.4072 | 4.74E-17 |
| SNORD19B | FILIP1L | Alternate Terminator | 7.5 | 0.0826 | -0.4071 | 4.82E-17 |
| SNORD19B | TUBB6 | Alternate Terminator | 6 | 0.1516 | 0.4061 | 5.92E-17 |
| SNORD19B | CERCAM | Retained Intron | 3.2 | 0.2229 | 0.4015 | 1.40E-16 |
| SNORD19B | LRRFIP2 | Exon Skip | 20 | 0.1841 | -0.4041 | 1.47E-16 |
| SNORD19B | ATP8B2 | Alternate Terminator | 16 | 0.1182 | 0.3992 | 2.18E-16 |
| SNORD19B | PTGER3 | Alternate Terminator | 8 | 0.2932 | 0.399 | 2.25E-16 |
| SNORD19B | ATP8B2 | Alternate Terminator | 31 | 0.1193 | -0.3971 | 3.20E-16 |
| SNORD19B | PAK1 | Alternate Promoter | 2 | 0.2532 | -0.4004 | 4.55E-16 |
| SNORD19B | PAK1 | Alternate Promoter | 1 | 0.2532 | 0.4004 | 4.55E-16 |
| SNORD19B | PTGER3 | Alternate Terminator | 2.3 | 0.1095 | -0.3941 | 5.64E-16 |
| SNORD19B | RASSF1 | Alternate Promoter | 1 | 0.0565 | -0.3941 | 6.09E-16 |
| SNORD19B | SLC16A1 | Alternate Terminator | 5.2 | 0.2241 | 0.3929 | 6.91E-16 |
| SNORD19B | SLC16A1 | Alternate Terminator | 6 | 0.2241 | -0.3929 | 6.91E-16 |
| SNORD19B | SIGIRR | Alternate Promoter | 2 | 0.2546 | -0.3896 | 1.63E-15 |
| SNORD19B | SIGIRR | Alternate Promoter | 1.1 | 0.2546 | 0.3896 | 1.63E-15 |
| SNORD19B | FKBP5 | Alternate Terminator | 10.2 | 0.0708 | 0.3879 | 1.73E-15 |
| SNORD19B | FKBP5 | Alternate Terminator | 13 | 0.0708 | -0.3879 | 1.73E-15 |
| SNORD19B | LTBP1 | Alternate Promoter | 5.1 | 0.3029 | -0.4096 | 2.90E-15 |
| SNORD19B | RNF128 | Alternate Promoter | 2 | 0.3345 | -0.3965 | 3.76E-15 |
| SNORD19B | RNF128 | Alternate Promoter | 1 | 0.3345 | 0.3965 | 3.76E-15 |
| SNORD19B | LTBP1 | Alternate Promoter | 1 | 0.3011 | 0.4075 | 4.13E-15 |
| SNORD19B | P4HA3 | Alternate Terminator | 16 | 0.2276 | 0.3825 | 4.90E-15 |
| SNORD19B | P4HA3 | Alternate Terminator | 14.2 | 0.2276 | -0.3825 | 4.91E-15 |
| SNORD19B | TAGLN | Alternate Promoter | 2.1 | 0.0444 | 0.3814 | 5.52E-15 |
| SNORD19B | TAGLN | Alternate Promoter | 1.1 | 0.0444 | -0.3812 | 5.65E-15 |
| SNORD19B | IL1R1 | Alternate Promoter | 3 | 0.185 | 0.4207 | 6.78E-15 |
| SNORD19B | KLHL5 | Alternate Terminator | 11.2 | 0.089 | 0.3793 | 8.00E-15 |
| SNORD19B | IFITM2 | Alternate Promoter | 1 | 0.12 | 0.3773 | 1.12E-14 |
| SNORD19B | IFITM2 | Alternate Promoter | 2.1 | 0.12 | -0.3773 | 1.12E-14 |
| SNORD19B | GOLM1 | Alternate Promoter | 2 | 0.1542 | 0.3876 | 1.12E-14 |
| SNORD19B | GOLM1 | Alternate Promoter | 1 | 0.1542 | -0.3876 | 1.12E-14 |
| SNORD19B | RTEL1 | Alternate Terminator | 35.2 | 0.186 | 0.3773 | 1.13E-14 |
| SNORD19B | RTEL1 | Alternate Terminator | 36 | 0.186 | -0.3773 | 1.13E-14 |
| SNORD19B | GALNT10 | Alternate Terminator | 15 | 0.0434 | -0.3767 | 1.24E-14 |
| SNORD19B | GALNT10 | Alternate Terminator | 8.2 | 0.0434 | 0.3767 | 1.24E-14 |
| SNORD19B | TPM1 | Alternate Promoter | 1 | 0.2568 | -0.3747 | 1.77E-14 |
| SNORD19B | EMC8 | Exon Skip | 4 | 0.0477 | 0.3724 | 2.60E-14 |
| SNORD19B | ATP5C1 | Exon Skip | 9 | 0.0667 | 0.3717 | 2.94E-14 |
| SNORD19B | ST3GAL4 | Alternate Promoter | 5.1 | 0.2102 | 0.3729 | 3.01E-14 |
| SNORD19B | DCN | Alternate Terminator | 6 | 0.0813 | 0.3707 | 3.48E-14 |
| SNORD19B | BMP1 | Exon Skip | 17.1:17.2 | 0.1224 | 0.3716 | 3.76E-14 |
| SNORD19B | DBN1 | Exon Skip | 14 | 0.0798 | -0.3722 | 3.94E-14 |
| SNORD19B | FCER1G | Alternate Terminator | 5 | 0.1003 | -0.3697 | 4.12E-14 |
| SNORD19B | FCER1G | Alternate Terminator | 6 | 0.1003 | 0.3697 | 4.12E-14 |
| SNORD19B | FBN1 | Alternate Terminator | 66 | 0.0581 | -0.3684 | 5.14E-14 |
| SNORD19B | FBN1 | Alternate Terminator | 2.2 | 0.0577 | 0.3683 | 5.21E-14 |
| SNORD19B | PPHLN1 | Alternate Terminator | 19 | 0.0715 | -0.3651 | 9.00E-14 |
| SNORD19B | PTGER3 | Alternate Terminator | 12 | 0.1315 | -0.3645 | 9.93E-14 |
| SNORD19B | KLHL5 | Alternate Terminator | 12 | 0.0935 | -0.3644 | 1.01E-13 |
| SNORD19B | MBNL1 | Exon Skip | 8 | 0.2028 | 0.3878 | 1.41E-13 |
| SNORD19B | CAPZB | Exon Skip | 11 | 0.071 | -0.3619 | 1.52E-13 |
| SNORD19B | IL1R1 | Alternate Promoter | 2 | 0.1804 | -0.4003 | 1.64E-13 |
| SNORD19B | ATF4 | Retained Intron | 2.2 | 0.0718 | 0.3614 | 1.66E-13 |
| SNORD19B | RPS24 | Alternate Acceptor Sites | 5.1 | 0.2184 | 0.3613 | 1.69E-13 |
| SNORD19B | RASSF8 | Alternate Terminator | 9 | 0.1639 | 0.3605 | 1.92E-13 |
| SNORD19B | RASSF8 | Alternate Terminator | 8 | 0.1639 | -0.3605 | 1.92E-13 |
| SNORD19B | RBP1 | Alternate Terminator | 3 | 0.0721 | 0.3604 | 1.94E-13 |
| SNORD19B | OST4 | Retained Intron | 1.2 | 0.0045 | -0.3598 | 2.17E-13 |
| SNORD19B | MBNL2 | Exon Skip | 7 | 0.1813 | 0.391 | 2.80E-13 |
| SNORD19B | AP2S1 | Alternate Promoter | 2 | 0.0442 | -0.3581 | 2.85E-13 |
| SNORD19B | ST3GAL4 | Alternate Promoter | 2 | 0.1107 | -0.359 | 3.03E-13 |
| SNORD19B | CYB561A3 | Alternate Acceptor Sites | 6.6 | 0.231 | -0.3576 | 3.07E-13 |
| SNORD19B | KIAA0930 | Alternate Promoter | 5.1 | 0.1193 | -0.3601 | 3.89E-13 |
| SNORD19B | CCDC74A | Retained Intron | 4.2 | 0.1957 | 0.3654 | 3.96E-13 |
| SNORD19B | LEPRE1 | Retained Intron | 14.2:14.3 | 0.0918 | 0.356 | 3.97E-13 |
| SNORD19B | RCOR3 | Exon Skip | 5 | 0.1743 | 0.376 | 4.27E-13 |
| SNORD19B | TPM2 | Exon Skip | 7 | 0.1728 | 0.3604 | 6.03E-13 |
| SNORD19B | MAP3K7 | Exon Skip | 11 | 0.2178 | 0.356 | 6.45E-13 |
| SNORD19B | PPFIBP2 | Alternate Promoter | 8 | 0.3152 | 0.3713 | 6.95E-13 |
| SNORD19B | MAP2K7 | Exon Skip | 2 | 0.2106 | -0.3763 | 7.08E-13 |
| SNORD19B | PIK3R2 | Alternate Terminator | 16.2 | 0.0983 | 0.3518 | 7.83E-13 |
| SNORD19B | IL18BP | Retained Intron | 1.7 | 0.2266 | 0.3561 | 7.85E-13 |
| SNORD19B | PIK3R2 | Alternate Terminator | 25 | 0.0982 | -0.3512 | 8.53E-13 |
| SNORD19B | KLC1 | Exon Skip | 16 | 0.1581 | 0.3515 | 9.34E-13 |
| SNORD19B | RBP1 | Alternate Terminator | 6 | 0.0754 | -0.35 | 1.05E-12 |
| SNORD19B | NCOR2 | Exon Skip | 46.1:46.2 | 0.1108 | 0.3561 | 1.10E-12 |
| SNORD19B | LIMCH1 | Alternate Promoter | 5 | 0.1512 | -0.3924 | 1.14E-12 |
| SNORD19B | CSMD2 | Alternate Terminator | 50 | 0.2016 | 0.3486 | 1.30E-12 |
| SNORD19B | MYL6 | Alternate Terminator | 5 | 0.0291 | -0.3475 | 1.54E-12 |
| SNORD19B | S100PBP | Alternate Terminator | 9 | 0.0943 | -0.3469 | 1.68E-12 |
| SNORD19B | S100PBP | Alternate Terminator | 7 | 0.0943 | 0.3469 | 1.68E-12 |
| SNORD19B | GULP1 | Alternate Terminator | 15 | 0.1892 | -0.3469 | 1.68E-12 |
| SNORD19B | GULP1 | Alternate Terminator | 9.2 | 0.1892 | 0.3469 | 1.69E-12 |
| SNORD19B | SYTL2 | Exon Skip | 13 | 0.1923 | 0.3637 | 1.91E-12 |
| SNORD19B | CFLAR | Alternate Terminator | 14 | 0.0386 | 0.3451 | 2.24E-12 |
| SNORD19B | MYL6 | Alternate Terminator | 7 | 0.0191 | 0.3446 | 2.41E-12 |
| SNORD19B | SPINK1 | Alternate Terminator | 3.2 | 0.1516 | -0.3505 | 2.44E-12 |
| SNORD19B | SPINK1 | Alternate Terminator | 4 | 0.1516 | 0.3505 | 2.45E-12 |
| SNORD19B | IL1RAP | Alternate Terminator | 14 | 0.0781 | 0.3443 | 2.55E-12 |
| SNORD19B | CA12 | Exon Skip | 9 | 0.2719 | 0.3785 | 2.63E-12 |
| SNORD19B | DCN | Alternate Terminator | 12 | 0.0814 | -0.3435 | 2.86E-12 |
| SNORD19B | HLX | Retained Intron | 2.2 | 0.1587 | 0.3441 | 2.95E-12 |
| SNORD19B | ROR2 | Alternate Terminator | 14 | 0.0167 | -0.3503 | 3.06E-12 |
| SNORD19B | ROR2 | Alternate Terminator | 10.2 | 0.0167 | 0.3503 | 3.06E-12 |
| SNORD19B | SCIMP | Alternate Terminator | 5 | 0.1806 | 0.3429 | 3.15E-12 |
| SNORD19B | SCIMP | Alternate Terminator | 6 | 0.1806 | -0.3429 | 3.15E-12 |
| SNORD19B | KIAA0930 | Alternate Promoter | 1 | 0.1234 | 0.3462 | 3.39E-12 |
| SNORD19B | S100A13 | Alternate Promoter | 6 | 0.1896 | -0.3422 | 3.51E-12 |
| SNORD19B | MXRA8 | Alternate Promoter | 1 | 0.233 | 0.3442 | 3.56E-12 |
| SNORD19B | MXRA8 | Alternate Promoter | 2 | 0.233 | -0.3442 | 3.56E-12 |
| SNORD19B | TMEM161B | Alternate Terminator | 13 | 0.1731 | 0.3416 | 3.86E-12 |
| SNORD19B | AP2S1 | Alternate Promoter | 1.1 | 0.0444 | 0.3411 | 4.15E-12 |
| SNORD19B | DDAH1 | Alternate Promoter | 1 | 0.2346 | -0.3577 | 4.30E-12 |
| SNORD19B | BICD2 | Retained Intron | 7.2 | 0.173 | 0.3421 | 4.60E-12 |
| SNORD19B | PDLIM7 | Alternate Acceptor Sites | 10.1 | 0.1252 | 0.3403 | 4.69E-12 |
| SNORD19B | CYB561A3 | Retained Intron | 6.5 | 0.1629 | 0.3424 | 5.30E-12 |
| SNORD19B | TMEM161B | Alternate Terminator | 12.2 | 0.1717 | -0.3395 | 5.30E-12 |
| SNORD19B | LTBP3 | Alternate Promoter | 1.1 | 0.2116 | -0.3389 | 5.82E-12 |
| SNORD19B | SVIL | Exon Skip | 21 | 0.2558 | -0.3729 | 5.84E-12 |
| SNORD19B | ITGB3 | Alternate Terminator | 15 | 0.2547 | -0.3411 | 6.45E-12 |
| SNORD19B | DDAH1 | Alternate Promoter | 3 | 0.241 | 0.3546 | 6.70E-12 |
| SNORD19B | TPM1 | Alternate Promoter | 5.1 | 0.1086 | 0.3375 | 7.12E-12 |
| SNORD19B | PHLDB2 | Alternate Promoter | 3 | 0.2257 | -0.3463 | 7.26E-12 |
| SNORD19B | PTGER3 | Alternate Terminator | 4 | 0.1094 | -0.3371 | 7.58E-12 |
| SNORD19B | VSTM4 | Alternate Terminator | 9 | 0.1472 | -0.337 | 7.75E-12 |
| SNORD19B | VSTM4 | Alternate Terminator | 3 | 0.1472 | 0.337 | 7.75E-12 |
| SNORD19B | LAT2 | Alternate Terminator | 15 | 0.0985 | -0.3368 | 7.96E-12 |
| SNORD19B | LAT2 | Alternate Terminator | 14 | 0.0985 | 0.3368 | 7.96E-12 |
| SNORD19B | SPAG9 | Exon Skip | 30 | 0.1851 | -0.3389 | 8.40E-12 |
| SNORD19B | PTGIR | Alternate Terminator | 4.2 | 0.2147 | -0.3355 | 9.62E-12 |
| SNORD19B | PTGIR | Alternate Terminator | 3 | 0.2147 | 0.3355 | 9.62E-12 |
| SNORD19B | RSRC1 | Alternate Promoter | 2.1 | 0.0574 | 0.346 | 9.87E-12 |
| SNORD19B | RSRC1 | Alternate Promoter | 1 | 0.0574 | -0.346 | 9.87E-12 |
| SNORD19B | RASGEF1B | Alternate Terminator | 15 | 0.0796 | -0.335 | 1.05E-11 |
| SNORD19B | RASGEF1B | Alternate Terminator | 5.2 | 0.0796 | 0.335 | 1.05E-11 |
| SNORD19B | ZNF843 | Alternate Terminator | 3 | 0.0546 | 0.3348 | 1.07E-11 |
| SNORD19B | ZNF843 | Alternate Terminator | 2.2 | 0.0546 | -0.3348 | 1.07E-11 |
| SNORD19B | HMHA1 | Alternate Promoter | 15.1 | 0.1073 | 0.3348 | 1.07E-11 |
| SNORD19B | HMHA1 | Alternate Promoter | 2 | 0.0915 | -0.3348 | 1.08E-11 |
| SNORD19B | WRB | Alternate Donor Sites | 1.2 | 0.0536 | -0.3575 | 1.10E-11 |
| SNORD19B | C7orf10 | Alternate Terminator | 16 | 0.1956 | -0.3346 | 1.10E-11 |
| SNORD19B | C7orf10 | Alternate Terminator | 7 | 0.1956 | 0.3346 | 1.10E-11 |
| SNORD19B | PELI3 | Alternate Terminator | 7.2 | 0.089 | 0.334 | 1.21E-11 |
| SNORD19B | PELI3 | Alternate Terminator | 8 | 0.089 | -0.334 | 1.21E-11 |
| SNORD19B | WNT2B | Alternate Promoter | 1 | 0.2302 | 0.3379 | 1.25E-11 |
| SNORD19B | WNT2B | Alternate Promoter | 3 | 0.2302 | -0.3379 | 1.25E-11 |
| SNORD19B | NT5C3B | Exon Skip | 3 | 0.1412 | 0.336 | 1.30E-11 |
| SNORD19B | BCAP29 | Alternate Terminator | 11 | 0.0861 | 0.3334 | 1.32E-11 |
| SNORD19B | LIMA1 | Alternate Promoter | 4.1 | 0.2648 | -0.3333 | 1.34E-11 |
| SNORD19B | METTL22 | Exon Skip | 4 | 0.1998 | -0.3361 | 1.36E-11 |
| SNORD19B | CAPN2 | Alternate Promoter | 2 | 0.1586 | -0.3331 | 1.39E-11 |
| SNORD19B | CAPN2 | Alternate Promoter | 1 | 0.1586 | 0.3331 | 1.39E-11 |
| SNORD19B | RAD51C | Alternate Terminator | 10 | 0.1355 | -0.3329 | 1.42E-11 |
| SNORD19B | SARDH | Alternate Terminator | 24 | 0.2117 | -0.3334 | 1.59E-11 |
| SNORD19B | SEC23A | Alternate Terminator | 23 | 0.1123 | -0.332 | 1.64E-11 |
| SNORD19B | SEC23A | Alternate Terminator | 4.2 | 0.1122 | 0.3318 | 1.67E-11 |
| SNORD19B | TBC1D9B | Alternate Promoter | 1 | 0.1807 | -0.3335 | 1.77E-11 |
| SNORD19B | TBC1D9B | Alternate Promoter | 15 | 0.1807 | 0.3335 | 1.77E-11 |
| SNORD19B | ZDHHC7 | Exon Skip | 4 | 0.1578 | -0.3374 | 1.94E-11 |
| SNORD19B | SERPINH1 | Alternate Promoter | 2.1 | 0.0278 | 0.3307 | 1.98E-11 |
| SNORD19B | ENY2 | Alternate Donor Sites | 1.2:1.3 | 0.0927 | -0.3307 | 1.98E-11 |
| SNORD19B | RALBP1 | Alternate Promoter | 1 | 0.1994 | 0.3358 | 2.05E-11 |
| SNORD19B | RALBP1 | Alternate Promoter | 2 | 0.1994 | -0.3358 | 2.05E-11 |
| SNORD19B | RAI14 | Exon Skip | 18 | 0.1727 | 0.338 | 2.16E-11 |
| SNORD19B | CCSER2 | Exon Skip | 11 | 0.1576 | -0.3444 | 2.54E-11 |
| SNORD19B | ESAM | Alternate Terminator | 7 | 0.0475 | -0.328 | 2.92E-11 |
| SNORD19B | ESAM | Alternate Terminator | 8 | 0.0475 | 0.328 | 2.94E-11 |
| SNORD19B | KLHDC2 | Retained Intron | 7.2 | 0.1577 | 0.3277 | 3.06E-11 |
| SNORD19B | TMEM91 | Alternate Promoter | 1 | 0.1388 | -0.3277 | 3.07E-11 |
| SNORD19B | ACOXL | Alternate Terminator | 21 | 0.2058 | 0.3281 | 3.07E-11 |
| SNORD19B | NCK1 | Alternate Promoter | 1.1 | 0.1861 | 0.3371 | 3.15E-11 |
| SNORD19B | NCK1 | Alternate Promoter | 3 | 0.1861 | -0.3371 | 3.15E-11 |
| SNORD19B | FAM73B | Alternate Donor Sites | 15.3 | 0.1882 | 0.3286 | 3.18E-11 |
| SNORD19B | KIAA1217 | Exon Skip | 23:24 | 0.1274 | 0.3273 | 3.24E-11 |
| SNORD19B | PLXNC1 | Alternate Terminator | 32 | 0.1084 | -0.327 | 3.37E-11 |
| SNORD19B | PLXNC1 | Alternate Terminator | 31.2 | 0.1084 | 0.327 | 3.37E-11 |
| SNORD19B | CD55 | Alternate Terminator | 15 | 0.0838 | 0.3262 | 3.78E-11 |
| SNORD19B | CD55 | Alternate Terminator | 10.2 | 0.0838 | -0.3262 | 3.78E-11 |
| SNORD19B | DTNA | Exon Skip | 31:32.1 | 0.3764 | 0.3718 | 3.90E-11 |
| SNORD19B | PCED1A | Alternate Promoter | 1 | 0.1425 | -0.3258 | 4.04E-11 |
| SNORD19B | PCED1A | Alternate Promoter | 2 | 0.1425 | 0.3258 | 4.04E-11 |
| SNORD19B | PI4KA | Exon Skip | 37:38:39:40:41:42:43 | 0.1853 | -0.3415 | 4.04E-11 |
| SNORD19B | LDHA | Exon Skip | 3 | 0.1161 | 0.3254 | 4.25E-11 |
| SNORD19B | TECR | Exon Skip | 2 | 0.0577 | -0.3254 | 4.27E-11 |
| SNORD19B | ITPKB | Alternate Terminator | 3 | 0.0902 | 0.3253 | 4.32E-11 |
| SNORD19B | ITPKB | Alternate Terminator | 9 | 0.0902 | -0.3253 | 4.32E-11 |
| SNORD19B | EDF1 | Retained Intron | 4.2 | 0.141 | 0.3251 | 4.48E-11 |
| SNORD19B | 9-Sep | Alternate Promoter | 1 | 0.2105 | 0.3251 | 4.76E-11 |
| SNORD19B | IFRD2 | Retained Intron | 7.2 | 0.069 | 0.325 | 4.78E-11 |
| SNORD19B | C2ORF15 | Alternate Promoter | 1 | 0.0872 | 0.3245 | 4.88E-11 |
| SNORD19B | C2ORF15 | Alternate Promoter | 6 | 0.0872 | -0.3245 | 4.88E-11 |
| SNORD19B | HMGA2 | Alternate Terminator | 9.3 | 0.3302 | 0.3251 | 4.98E-11 |
| SNORD19B | CXorf40A | Alternate Promoter | 1.1 | 0.1697 | -0.3245 | 5.43E-11 |
| SNORD19B | RAD51C | Alternate Terminator | 2.2 | 0.1363 | 0.3236 | 5.58E-11 |
| SNORD19B | MID1 | Alternate Promoter | 4 | 0.2772 | 0.3267 | 5.60E-11 |
| SNORD19B | TAGLN | Retained Intron | 1.2 | 0.1416 | 0.3369 | 5.65E-11 |
| SNORD19B | C17orf70 | Alternate Promoter | 1 | 0.1511 | -0.3233 | 5.75E-11 |
| SNORD19B | C17orf70 | Alternate Promoter | 5.1 | 0.1511 | 0.3233 | 5.75E-11 |
| SNORD19B | PLIN5 | Alternate Terminator | 8 | 0.1081 | 0.3236 | 5.85E-11 |
| SNORD19B | PLIN5 | Alternate Terminator | 2.3 | 0.1081 | -0.3236 | 5.86E-11 |
| SNORD19B | SAR1B | Alternate Promoter | 7.1 | 0.2441 | 0.3229 | 6.12E-11 |
| SNORD19B | KLHL21 | Retained Intron | 4.2:4.3 | 0.196 | 0.322 | 7.02E-11 |
| SNORD19B | RUNX1 | Alternate Promoter | 1.1 | 0.0995 | -0.3218 | 7.22E-11 |
| SNORD19B | LIMCH1 | Exon Skip | 28 | 0.1576 | 0.3529 | 7.59E-11 |
| SNORD19B | CSMD2 | Alternate Terminator | 17.2 | 0.0772 | -0.3213 | 7.72E-11 |
| SNORD19B | KIF1B | Alternate Terminator | 25 | 0.1151 | -0.321 | 8.02E-11 |
| SNORD19B | KIF1B | Alternate Terminator | 52.2 | 0.1153 | 0.321 | 8.02E-11 |
| SNORD19B | RNF130 | Alternate Terminator | 10 | 0.0662 | 0.3208 | 8.29E-11 |
| SNORD19B | RNF130 | Alternate Terminator | 9 | 0.0662 | -0.3208 | 8.29E-11 |
| SNORD19B | ACOXL | Alternate Terminator | 11 | 0.1562 | -0.3212 | 8.30E-11 |
| SNORD19B | ARL16 | Exon Skip | 4 | 0.1659 | 0.3204 | 8.77E-11 |
| SNORD19B | EXOC7 | Exon Skip | 7:8.1:8.2 | 0.1464 | 0.3203 | 9.87E-11 |
| SNORD19B | PTK2 | Alternate Promoter | 30 | 0.1519 | -0.3208 | 1.04E-10 |
| SNORD19B | SVEP1 | Alternate Terminator | 28.2 | 0.1893 | 0.3189 | 1.09E-10 |
| SNORD19B | ITGB3 | Alternate Terminator | 19 | 0.279 | 0.3216 | 1.10E-10 |
| SNORD19B | ILK | Retained Intron | 5.4 | 0.0623 | 0.3186 | 1.13E-10 |
| SNORD19B | CD33 | Alternate Terminator | 7 | 0.2026 | -0.3185 | 1.15E-10 |
| SNORD19B | CD33 | Alternate Terminator | 6.3 | 0.2026 | 0.3185 | 1.15E-10 |
| SNORD19B | ABCC9 | Alternate Terminator | 43 | 0.0189 | 0.3184 | 1.16E-10 |
| SNORD19B | TRIM69 | Alternate Promoter | 3.1 | 0.0972 | 0.318 | 1.23E-10 |
| SNORD19B | SEC31A | Exon Skip | 26.1:26.2 | 0.118 | 0.3183 | 1.24E-10 |
| SNORD19B | KIF13A | Exon Skip | 40 | 0.2188 | 0.3202 | 1.25E-10 |
| SNORD19B | TPM1 | Alternate Promoter | 4 | 0.213 | 0.3171 | 1.39E-10 |
| SNORD19B | ROGDI | Alternate Donor Sites | 4.2 | 0.1615 | 0.3175 | 1.39E-10 |
| SNORD19B | PHLDB2 | Alternate Promoter | 1 | 0.3291 | 0.3256 | 1.39E-10 |
| SNORD19B | KLC1 | Exon Skip | 13.3:15 | 0.1352 | -0.3168 | 1.45E-10 |
| SNORD19B | TBC1D23 | Exon Skip | 15 | 0.2079 | -0.3273 | 1.47E-10 |
| SNORD19B | KPNA1 | Exon Skip | 6 | 0.1634 | 0.324 | 1.54E-10 |
| SNORD19B | ACSL5 | Alternate Promoter | 1 | 0.0992 | 0.319 | 1.56E-10 |
| SNORD19B | P2RY6 | Alternate Promoter | 3.1 | 0.2871 | 0.3244 | 1.63E-10 |
| SNORD19B | MEFV | Exon Skip | 6 | 0.1116 | 0.3171 | 1.64E-10 |
| SNORD19B | MYH11 | Exon Skip | 42 | 0.2198 | -0.3349 | 1.64E-10 |
| SNORD19B | C2ORF15 | Alternate Terminator | 5.2 | 0.0917 | 0.3146 | 1.97E-10 |
| SNORD19B | EVI5L | Exon Skip | 12 | 0.181 | -0.3252 | 2.07E-10 |
| SNORD19B | SAR1B | Alternate Promoter | 2 | 0.1931 | -0.3142 | 2.09E-10 |
| SNORD19B | MOGS | Retained Intron | 3.3 | 0.0612 | 0.3139 | 2.17E-10 |
| SNORD19B | ARPC1B | Exon Skip | 8 | 0.0892 | 0.3139 | 2.19E-10 |
| SNORD19B | SVEP1 | Alternate Terminator | 49 | 0.1879 | -0.3137 | 2.24E-10 |
| SNORD19B | ELP3 | Alternate Promoter | 2 | 0.2379 | -0.3263 | 2.25E-10 |
| SNORD19B | ELP3 | Alternate Promoter | 1 | 0.2379 | 0.3263 | 2.25E-10 |
| SNORD19B | ABCC9 | Alternate Terminator | 7.2 | 0.0185 | -0.3135 | 2.30E-10 |
| SNORD19B | STEAP4 | Alternate Terminator | 6 | 0.1652 | -0.3126 | 2.61E-10 |
| SNORD19B | STEAP4 | Alternate Terminator | 4.2 | 0.1652 | 0.3126 | 2.61E-10 |
| SNORD19B | TRAPPC2 | Retained Intron | 6.2 | 0.0921 | -0.3124 | 2.70E-10 |
| SNORD19B | VDAC3 | Exon Skip | 5.2 | 0.0766 | -0.3124 | 2.81E-10 |
| SNORD19B | PDE4DIP | Alternate Acceptor Sites | 52.1 | 0.1059 | 0.319 | 2.86E-10 |
| SNORD19B | MND1 | Exon Skip | 5:06 | 0.0986 | 0.3122 | 2.88E-10 |
| SNORD19B | ARHGEF4 | Alternate Terminator | 14.3 | 0.2695 | -0.3115 | 3.05E-10 |
| SNORD19B | ARHGEF4 | Alternate Terminator | 4.2 | 0.2695 | 0.3115 | 3.05E-10 |
| SNORD19B | TTLL5 | Alternate Terminator | 5.2 | 0.048 | 0.3112 | 3.15E-10 |
| SNORD19B | TAGLN | Alternate Donor Sites | 1.2:1.3 | 0.0477 | 0.3109 | 3.27E-10 |
| SNORD19B | PSTPIP1 | Exon Skip | 3 | 0.2082 | 0.3113 | 3.27E-10 |
| SNORD19B | RASA4 | Alternate Promoter | 2.1 | 0.175 | -0.3166 | 3.38E-10 |
| SNORD19B | RASA4 | Alternate Promoter | 1.1 | 0.175 | 0.3166 | 3.38E-10 |
| SNORD19B | TTC7A | Alternate Promoter | 3 | 0.2353 | -0.313 | 3.57E-10 |
| SNORD19B | TTC7A | Alternate Promoter | 1 | 0.2353 | 0.313 | 3.57E-10 |
| SNORD19B | TRAK1 | Alternate Promoter | 5 | 0.2376 | 0.3194 | 3.57E-10 |
| SNORD19B | ZBTB25 | Alternate Terminator | 7.3 | 0.1089 | 0.3102 | 3.65E-10 |
| SNORD19B | ZBTB25 | Alternate Terminator | 8 | 0.1089 | -0.3101 | 3.65E-10 |
| SNORD19B | TRIM69 | Alternate Promoter | 1 | 0.0989 | -0.3101 | 3.65E-10 |
| SNORD19B | TBC1D1 | Alternate Terminator | 23 | 0.1102 | -0.3099 | 3.79E-10 |
| SNORD19B | TBC1D1 | Alternate Terminator | 3 | 0.1102 | 0.3099 | 3.79E-10 |
| SNORD19B | NFYA | Exon Skip | 3 | 0.1789 | -0.328 | 3.79E-10 |
| SNORD19B | ACOT9 | Exon Skip | 6 | 0.2181 | -0.3222 | 3.84E-10 |
| SNORD19B | ATP2A2 | Retained Intron | 21.2:21.3 | 0.0729 | 0.3093 | 4.07E-10 |
| SNORD19B | SERPINF1 | Alternate Acceptor Sites | 4.1 | 0.2027 | 0.3128 | 4.09E-10 |
| SNORD19B | APEX1 | Alternate Donor Sites | 2.3 | 0.0698 | 0.3092 | 4.35E-10 |
| SNORD19B | EDF1 | Retained Intron | 4.2:4.3 | 0.0311 | 0.3085 | 4.59E-10 |
| SNORD19B | PPM1F | Alternate Terminator | 9 | 0.0729 | -0.308 | 4.92E-10 |
| SNORD19B | PPM1F | Alternate Terminator | 8.2 | 0.0729 | 0.308 | 4.92E-10 |
| SNORD19B | HDAC10 | Exon Skip | 14 | 0.1031 | -0.3071 | 5.55E-10 |
| SNORD19B | ARHGEF11 | Exon Skip | 39 | 0.2057 | -0.3118 | 5.73E-10 |
| SNORD19B | C2ORF15 | Alternate Terminator | 11.2 | 0.0942 | -0.3068 | 5.74E-10 |
| SNORD19B | CXorf40A | Alternate Promoter | 2 | 0.181 | 0.3075 | 5.78E-10 |
| SNORD19B | LTBP3 | Alternate Promoter | 17 | 0.1538 | 0.3067 | 5.80E-10 |
| SNORD19B | CLEC3A | Alternate Terminator | 3 | 0.2746 | -0.3088 | 5.98E-10 |
| SNORD19B | CLEC3A | Alternate Terminator | 5 | 0.2746 | 0.3088 | 5.98E-10 |
| SNORD19B | H2AFV | Alternate Terminator | 4.2 | 0.0189 | 0.3063 | 6.15E-10 |
| SNORD19B | 9-Sep | Alternate Terminator | 24.5 | 0.0469 | -0.3058 | 6.60E-10 |
| SNORD19B | 9-Sep | Alternate Terminator | 16.2 | 0.0469 | 0.3058 | 6.60E-10 |
| SNORD19B | ILK | Retained Intron | 5.2 | 0.0428 | 0.3057 | 6.63E-10 |
| SNORD19B | RPS21 | Alternate Acceptor Sites | 3.3:3.4 | 0.1525 | 0.3149 | 6.88E-10 |
| SNORD19B | TMEM108 | Alternate Terminator | 8 | 0.2977 | -0.3048 | 7.49E-10 |
| SNORD19B | TMEM108 | Alternate Terminator | 6 | 0.2977 | 0.3048 | 7.49E-10 |
| SNORD19B | WDR62 | Retained Intron | 25.6 | 0.1879 | 0.3044 | 7.90E-10 |
| SNORD19B | SPHK2 | Alternate Promoter | 1.1 | 0.2605 | -0.333 | 8.41E-10 |
| SNORD19B | ENAH | Exon Skip | 13 | 0.2285 | 0.3074 | 8.78E-10 |
| SNORD19B | AAAS | Exon Skip | 7 | 0.0182 | 0.3037 | 9.14E-10 |
| SNORD19B | ARHGEF10L | Exon Skip | 18 | 0.1592 | 0.3081 | 9.35E-10 |
| SNORD19B | SLC39A1 | Alternate Promoter | 1 | 0.1145 | 0.3031 | 9.41E-10 |
| SNORD19B | SAR1B | Alternate Promoter | 1 | 0.0831 | -0.3031 | 9.42E-10 |
| SNORD19B | FAM178A | Alternate Terminator | 20 | 0.096 | -0.3021 | 1.07E-09 |
| SNORD19B | IMPA2 | Alternate Terminator | 11 | 0.084 | -0.302 | 1.09E-09 |
| SNORD19B | IMPA2 | Alternate Terminator | 8 | 0.084 | 0.302 | 1.09E-09 |
| SNORD19B | TMEM120A | Exon Skip | 10 | 0.1077 | -0.3028 | 1.20E-09 |
| SNORD19B | CDC37 | Retained Intron | 4.2 | 0.1362 | 0.3011 | 1.23E-09 |
| SNORD19B | SRC | Alternate Promoter | 1 | 0.1944 | 0.3045 | 1.23E-09 |
| SNORD19B | STRA6 | Alternate Donor Sites | 18.2 | 0.2535 | 0.3006 | 1.31E-09 |
| SNORD19B | PLA2G2A | Exon Skip | 2 | 0.1212 | -0.3399 | 1.33E-09 |
| SNORD19B | BTN2A1 | Alternate Acceptor Sites | 9.1 | 0.1429 | 0.3012 | 1.41E-09 |
| SNORD19B | SRC | Alternate Promoter | 2 | 0.1941 | -0.3021 | 1.68E-09 |
| SNORD19B | NNMT | Alternate Donor Sites | 5.2 | 0.0039 | 0.3005 | 1.70E-09 |
| SNORD19B | MID1 | Alternate Promoter | 3.1 | 0.2248 | -0.3016 | 1.70E-09 |
| SNORD19B | DNMT3B | Exon Skip | 22:23 | 0.1615 | 0.3096 | 1.75E-09 |
| SNORD19B | TRIM29 | Alternate Promoter | 2.1 | 0.1071 | -0.3009 | 1.78E-09 |
| SNORD19B | BCAR3 | Alternate Promoter | 11 | 0.2473 | 0.3149 | 1.79E-09 |
| SNORD19B | TRAK1 | Alternate Promoter | 1 | 0.2362 | -0.3068 | 1.84E-09 |
| SNORD19B | OSR1 | Alternate Promoter | 2.1 | 0.0991 | 0.3068 | 1.94E-09 |
| SNORD19B | OSR1 | Alternate Promoter | 1 | 0.0991 | -0.3068 | 1.94E-09 |
| SNORD19B | TMUB2 | Exon Skip | 2.2:2.3:2.4:2.5:3 | 0.1553 | 0.3136 | 2.10E-09 |
| SNORD19B | SPHK2 | Alternate Promoter | 3.1 | 0.2598 | 0.3251 | 2.17E-09 |
| SNORD19B | TPM2 | Exon Skip | 6 | 0.2679 | 0.3109 | 2.38E-09 |
| SNORD19B | SYTL2 | Alternate Promoter | 10.1 | 0.1833 | -0.3097 | 2.51E-09 |
| SNORD19B | SGSM2 | Retained Intron | 5.2 | 0.1988 | 0.3048 | 2.62E-09 |
| SNORD19B | LYST | Alternate Promoter | 2.1 | 0.1983 | 0.3107 | 3.01E-09 |
| SNORD19B | LYST | Alternate Promoter | 1 | 0.1983 | -0.3107 | 3.01E-09 |
| SNORD19B | EXOC7 | Exon Skip | 8.1:8.2 | 0.2146 | 0.3081 | 3.02E-09 |
| SNORD19B | FAM65A | Alternate Promoter | 1 | 0.2187 | -0.327 | 3.70E-09 |
| SNORD19B | RUNX2 | Alternate Promoter | 1.1 | 0.3026 | -0.3201 | 3.74E-09 |
| SNORD19B | PSMC3IP | Alternate Donor Sites | 4.2:4.3 | 0.2452 | 0.3117 | 4.05E-09 |
| SNORD19B | NEDD4L | Exon Skip | 18 | 0.231 | 0.3051 | 4.18E-09 |
| SNORD19B | RUNX2 | Alternate Promoter | 2.1 | 0.3025 | 0.3168 | 5.53E-09 |
| SNORD19B | FAM65A | Alternate Promoter | 3 | 0.2218 | 0.3217 | 6.77E-09 |
| SNORD19B | PODNL1 | Alternate Promoter | 1 | 0.2812 | 0.3012 | 7.43E-09 |
| SNORD19B | PLCD4 | Exon Skip | 13 | 0.2295 | 0.325 | 9.24E-09 |
| SNORD19B | EXOC7 | Alternate Acceptor Sites | 8.1 | 0.2364 | 0.3035 | 9.26E-09 |
| SNORD19B | ATP2C1 | Alternate Promoter | 1 | 0.2436 | -0.3004 | 1.20E-08 |
| SNORD19B | PI4KA | Alternate Promoter | 1 | 0.2538 | -0.3011 | 1.35E-08 |
| SNORD19B | PI4KA | Alternate Promoter | 33.1 | 0.2538 | 0.3011 | 1.35E-08 |
| SNORD19B | PUS10 | Alternate Promoter | 1 | 0.1634 | 0.3173 | 1.38E-08 |
| SNORD19B | PUS10 | Alternate Promoter | 2.1 | 0.1634 | -0.3173 | 1.38E-08 |
| SNORD19B | ARHGEF10L | Alternate Promoter | 1 | 0.2527 | 0.3172 | 3.03E-08 |
| SNORD19B | TLE2 | Retained Intron | 10.2:10.3 | 0.2241 | 0.302 | 4.58E-08 |
| SNORD19B | ARHGEF10L | Alternate Promoter | 10 | 0.2409 | -0.3049 | 1.06E-07 |
| SNORD113-9 | CSMD2 | Alternate Terminator | 50 | 0.2016 | -0.3543 | 5.27E-13 |
| SNORD113-9 | CSMD2 | Alternate Terminator | 78 | 0.112 | 0.3738 | 2.05E-14 |
| SNORD113-9 | PTGER3 | Alternate Terminator | 12 | 0.1315 | 0.3117 | 2.95E-10 |
| SNORD113-9 | PTGER3 | Alternate Terminator | 4 | 0.1094 | 0.3322 | 1.58E-11 |
| SNORD113-9 | PTGER3 | Alternate Terminator | 8 | 0.2932 | -0.3128 | 2.52E-10 |
| SNORD113-9 | ATP5C1 | Exon Skip | 9 | 0.0667 | -0.3038 | 8.56E-10 |
| SNORD113-9 | KIAA1217 | Exon Skip | 23:24 | 0.1274 | -0.3054 | 6.97E-10 |
| SNORD113-9 | VSTM4 | Alternate Terminator | 9 | 0.1472 | 0.3116 | 2.97E-10 |
| SNORD113-9 | VSTM4 | Alternate Terminator | 3 | 0.1472 | -0.3116 | 2.97E-10 |
| SNORD113-9 | P4HA3 | Alternate Terminator | 16 | 0.2276 | -0.3058 | 6.94E-10 |
| SNORD114-1 | GNG5 | Exon Skip | 2 | 0.0228 | 0.4354 | 7.12E-19 |
| SNORD114-1 | ALG3 | Alternate Promoter | 1.1 | 0.0642 | 0.4188 | 5.43E-18 |
| SNORD114-1 | ALG3 | Alternate Promoter | 2.1 | 0.0642 | -0.4188 | 5.43E-18 |
| SNORD114-1 | RPL36 | Alternate Donor Sites | 4.2:4.3 | 0.0045 | -0.4082 | 3.89E-17 |
| SNORD114-1 | HIRA | Alternate Promoter | 3 | 0.2206 | 0.4002 | 7.39E-16 |
| SNORD114-1 | HIRA | Alternate Promoter | 1 | 0.2206 | -0.4002 | 7.39E-16 |
| SNORD114-1 | EIF3G | Alternate Donor Sites | 3.3 | 0.0208 | -0.3865 | 2.22E-15 |
| SNORD114-1 | TPM4 | Retained Intron | 10.2 | 0.0043 | -0.3826 | 4.47E-15 |
| SNORD114-1 | ALDOA | Alternate Donor Sites | 12.2 | 0.0123 | -0.3817 | 5.24E-15 |
| SNORD114-1 | B2M | Exon Skip | 1.2:2.1:2.2 | 0.0041 | -0.3674 | 6.08E-14 |
| SNORD114-1 | TMEM8B | Alternate Promoter | 4.1 | 0.1322 | 0.3614 | 1.79E-13 |
| SNORD114-1 | TMEM8B | Alternate Promoter | 1 | 0.1252 | -0.3565 | 3.95E-13 |
| SNORD114-1 | DENND5B | Alternate Terminator | 12.2 | 0.1356 | -0.3529 | 6.52E-13 |
| SNORD114-1 | DENND5B | Alternate Terminator | 25 | 0.1356 | 0.3529 | 6.52E-13 |
| SNORD114-1 | RPLP0 | Retained Intron | 1.2 | 0.0354 | -0.3485 | 1.31E-12 |
| SNORD114-1 | EDEM2 | Alternate Promoter | 1 | 0.1791 | -0.3454 | 2.13E-12 |
| SNORD114-1 | ZBTB17 | Retained Intron | 9.2 | 0.112 | -0.3456 | 2.20E-12 |
| SNORD114-1 | NDRG1 | Exon Skip | 2.2:2.3:3:4:5.1:5.2:6:7.1  7.2:8:10:11:12:13:14:15:16.1 | 0.0337 | 0.3451 | 2.25E-12 |
| SNORD114-1 | ADAMTS2 | Alternate Terminator | 11 | 0.1358 | -0.339 | 5.73E-12 |
| SNORD114-1 | ADAMTS2 | Alternate Terminator | 23 | 0.1358 | 0.339 | 5.73E-12 |
| SNORD114-1 | CREM | Alternate Promoter | 1 | 0.1853 | -0.3391 | 6.39E-12 |
| SNORD114-1 | POM121 | Alternate Terminator | 17 | 0.108 | 0.3379 | 6.75E-12 |
| SNORD114-1 | POM121 | Alternate Terminator | 18 | 0.108 | -0.3379 | 6.75E-12 |
| SNORD114-1 | EDEM2 | Alternate Promoter | 6 | 0.1782 | 0.3351 | 1.02E-11 |
| SNORD114-1 | HMG20B | Alternate Acceptor Sites | 4.1 | 0.0826 | -0.3348 | 1.13E-11 |
| SNORD114-1 | DDX5 | Exon Skip | 4.2:5:6.1:6.2:7:8.1 | 0.0047 | 0.3344 | 1.14E-11 |
| SNORD114-1 | FBN1 | Alternate Terminator | 66 | 0.0581 | 0.3323 | 1.56E-11 |
| SNORD114-1 | FBN1 | Alternate Terminator | 2.2 | 0.0577 | -0.3321 | 1.61E-11 |
| SNORD114-1 | MMP23B | Retained Intron | 3.4 | 0.2055 | -0.3447 | 2.00E-11 |
| SNORD114-1 | DCN | Alternate Terminator | 12 | 0.0814 | 0.3285 | 2.75E-11 |
| SNORD114-1 | VSTM4 | Alternate Terminator | 9 | 0.1472 | 0.3257 | 4.12E-11 |
| SNORD114-1 | VSTM4 | Alternate Terminator | 3 | 0.1472 | -0.3257 | 4.12E-11 |
| SNORD114-1 | ACTG1 | Alternate Donor Sites | 1.2 | 0.0042 | -0.322 | 6.96E-11 |
| SNORD114-1 | MXRA8 | Alternate Acceptor Sites | 10.1 | 0.1263 | -0.3277 | 7.73E-11 |
| SNORD114-1 | TPM2 | Exon Skip | 7 | 0.1728 | -0.3277 | 7.81E-11 |
| SNORD114-1 | EXOC7 | Exon Skip | 7:8.1:8.2 | 0.1464 | -0.3216 | 8.31E-11 |
| SNORD114-1 | TSPO | Alternate Promoter | 1 | 0.0628 | -0.3201 | 9.18E-11 |
| SNORD114-1 | TSPO | Alternate Promoter | 2 | 0.0628 | 0.32 | 9.30E-11 |
| SNORD114-1 | DCN | Alternate Terminator | 6 | 0.0813 | -0.3187 | 1.12E-10 |
| SNORD114-1 | ULK3 | Retained Intron | 6.2 | 0.1771 | -0.3197 | 1.21E-10 |
| SNORD114-1 | TMEM175 | Exon Skip | 2:3:4.1:4.2 | 0.1969 | -0.321 | 1.25E-10 |
| SNORD114-1 | P4HA3 | Alternate Terminator | 16 | 0.2276 | -0.318 | 1.30E-10 |
| SNORD114-1 | P4HA3 | Alternate Terminator | 14.2 | 0.2276 | 0.318 | 1.31E-10 |
| SNORD114-1 | NRBP2 | Retained Intron | 5.2 | 0.2275 | -0.3346 | 1.51E-10 |
| SNORD114-1 | TPM1 | Alternate Promoter | 4 | 0.213 | -0.3149 | 1.91E-10 |
| SNORD114-1 | ZNF771 | Alternate Terminator | 4 | 0.129 | -0.3139 | 2.18E-10 |
| SNORD114-1 | CERCAM | Retained Intron | 3.2 | 0.2229 | -0.3135 | 2.31E-10 |
| SNORD114-1 | LMO4 | Alternate Promoter | 1 | 0.1034 | 0.3134 | 2.47E-10 |
| SNORD114-1 | LMO4 | Alternate Promoter | 2 | 0.1034 | -0.3134 | 2.47E-10 |
| SNORD114-1 | ARL6IP4 | Exon Skip | 3.4:4.1 | 0.0033 | 0.312 | 2.84E-10 |
| SNORD114-1 | SEC14L1 | Alternate Promoter | 1 | 0.1351 | -0.312 | 2.84E-10 |
| SNORD114-1 | DUSP18 | Alternate Terminator | 3 | 0.1303 | -0.3119 | 2.85E-10 |
| SNORD114-1 | TCF12 | Alternate Promoter | 10 | 0.0994 | 0.3216 | 2.99E-10 |
| SNORD114-1 | MXRA8 | Alternate Promoter | 1 | 0.233 | -0.3132 | 3.12E-10 |
| SNORD114-1 | MXRA8 | Alternate Promoter | 2 | 0.233 | 0.3132 | 3.12E-10 |
| SNORD114-1 | SGSM3 | Retained Intron | 19.2 | 0.1512 | -0.3109 | 3.30E-10 |
| SNORD114-1 | LEPRE1 | Retained Intron | 14.3 | 0.1736 | -0.3107 | 3.37E-10 |
| SNORD114-1 | PPHLN1 | Alternate Terminator | 19 | 0.0715 | 0.3086 | 4.49E-10 |
| SNORD114-1 | FASTK | Retained Intron | 5.2 | 0.055 | -0.3081 | 4.85E-10 |
| SNORD114-1 | SRSF2 | Alternate Donor Sites | 1.2 | 0.0512 | -0.3076 | 5.40E-10 |
| SNORD114-1 | FAM120AOS | Alternate Promoter | 1 | 0.067 | 0.3064 | 6.09E-10 |
| SNORD114-1 | FAM120AOS | Alternate Promoter | 2 | 0.067 | -0.3064 | 6.09E-10 |
| SNORD114-1 | TMEM175 | Exon Skip | 4.1:4.2 | 0.1919 | -0.3083 | 6.35E-10 |
| SNORD114-1 | SBNO2 | Alternate Promoter | 1 | 0.2192 | -0.3249 | 6.37E-10 |
| SNORD114-1 | SBNO2 | Alternate Promoter | 5 | 0.2192 | 0.3249 | 6.37E-10 |
| SNORD114-1 | SYTL2 | Exon Skip | 13 | 0.1923 | -0.3212 | 6.89E-10 |
| SNORD114-1 | TMEM120B | Retained Intron | 12.2 | 0.0638 | 0.3041 | 8.21E-10 |
| SNORD114-1 | TMEM258 | Alternate Donor Sites | 3.3 | 0.0067 | -0.3037 | 8.67E-10 |
| SNORD114-1 | ME3 | Exon Skip | 10 | 0.2117 | -0.3085 | 1.04E-09 |
| SNORD114-1 | SERPINF1 | Alternate Acceptor Sites | 4.1 | 0.2027 | -0.3054 | 1.10E-09 |
| SNORD114-1 | MPC2 | Alternate Promoter | 2.1 | 0.0129 | -0.3018 | 1.12E-09 |
| SNORD114-1 | MPC2 | Alternate Promoter | 1 | 0.0129 | 0.3018 | 1.12E-09 |
| SNORD114-1 | SRSF7 | Alternate Acceptor Sites | 4.3:4.4:4.5 | 0.0931 | -0.3015 | 1.17E-09 |
| SNORD114-1 | RPL36 | Retained Intron | 4.4 | 0.0747 | 0.3018 | 1.18E-09 |
| SNORD114-1 | LTBP3 | Alternate Promoter | 1.1 | 0.2116 | 0.3013 | 1.20E-09 |
| SNORD114-1 | CSMD2 | Alternate Terminator | 50 | 0.2016 | -0.3011 | 1.23E-09 |
| SNORD114-1 | C7orf10 | Alternate Terminator | 16 | 0.1956 | 0.3011 | 1.24E-09 |
| SNORD114-1 | C7orf10 | Alternate Terminator | 7 | 0.1956 | -0.3011 | 1.24E-09 |
| SNORD114-1 | ACOXL | Alternate Terminator | 21 | 0.2058 | -0.3003 | 1.43E-09 |
| SNORD114-1 | PHLDB1 | Exon Skip | 18.3 | 0.1124 | 0.3076 | 1.95E-09 |
| SNORD114-1 | SGSM2 | Retained Intron | 5.2 | 0.1988 | -0.3065 | 2.12E-09 |
| SNORD114-1 | SPG20 | Alternate Promoter | 3 | 0.0434 | -0.3205 | 2.71E-09 |
| SNORD114-1 | SYTL2 | Alternate Promoter | 10.1 | 0.1833 | 0.309 | 2.73E-09 |
| SNORD114-1 | ZDHHC4 | Retained Intron | 1.2 | 0.2147 | 0.3059 | 1.15E-08 |
| SNORD114-1 | PLA2G6 | Exon Skip | 11:12 | 0.1821 | -0.3092 | 1.39E-08 |
| SNORD114-1 | COL1A1 | Exon Skip | 17:18:19:20:21:22:23:24:28:29:30:31 | 0.1082 | -0.3017 | 1.95E-08 |
| SNORD114-1 | ZNF664 | Retained Intron | 1.3 | 0.1019 | -0.3076 | 4.61E-08 |
| SNORD114-1 | FOXP1 | Alternate Promoter | 2 | 0.267 | -0.3066 | 5.96E-08 |
| U3 | CIRBP | Exon Skip | 9.5:9.6 | 0.1621 | 0.3157 | 1.70E-10 |
| U3 | CIRBP | Alternate Acceptor Sites | 9.5:9.6:9.7 | 0.1417 | 0.3065 | 6.01E-10 |
| U3 | ANKRD44 | Alternate Terminator | 27 | 0.2149 | 0.3126 | 8.27E-10 |
| U3 | HNRNPC | Retained Intron | 8.3 | 0.019 | -0.3037 | 8.75E-10 |
| U3 | MEIS3 | Alternate Acceptor Sites | 7.1 | 0.226 | -0.3034 | 7.64E-09 |
| U49A | DACT3 | Alternate Terminator | 4.2 | 0.2496 | 0.452 | 4.39E-21 |
| U49A | DACT3 | Alternate Terminator | 5 | 0.2496 | -0.452 | 4.39E-21 |
| U49A | SVIL | Exon Skip | 21 | 0.2558 | -0.4806 | 7.62E-20 |
| U49A | SMTN | Exon Skip | 21.1:21.2 | 0.0841 | 0.4212 | 3.02E-18 |
| U49A | CALD1 | Exon Skip | 8.3:9 | 0.284 | -0.4176 | 9.26E-18 |
| U49A | SMTN | Alternate Promoter | 12 | 0.0749 | -0.4162 | 1.00E-17 |
| U49A | SMTN | Alternate Promoter | 1 | 0.0749 | 0.4162 | 1.00E-17 |
| U49A | NCAM1 | Alternate Terminator | 21 | 0.1022 | 0.4187 | 2.40E-17 |
| U49A | NCAM1 | Alternate Terminator | 18 | 0.1022 | -0.4187 | 2.40E-17 |
| U49A | ATP2B4 | Exon Skip | 21 | 0.2498 | -0.4236 | 6.88E-17 |
| U49A | LRRFIP1 | Exon Skip | 15 | 0.1529 | -0.4133 | 1.42E-16 |
| U49A | BBS5 | Alternate Terminator | 12 | 0.0629 | 0.3986 | 2.42E-16 |
| U49A | BBS5 | Alternate Terminator | 17 | 0.0629 | -0.3986 | 2.43E-16 |
| U49A | ACTA2 | Alternate Promoter | 2 | 0.0814 | -0.3955 | 4.30E-16 |
| U49A | ACTA2 | Alternate Promoter | 1 | 0.0814 | 0.3955 | 4.31E-16 |
| U49A | MYH11 | Exon Skip | 42 | 0.2198 | -0.4147 | 8.18E-16 |
| U49A | CLEC3A | Alternate Terminator | 3 | 0.2746 | -0.3893 | 2.22E-15 |
| U49A | CLEC3A | Alternate Terminator | 5 | 0.2746 | 0.3893 | 2.22E-15 |
| U49A | DCN | Alternate Terminator | 6 | 0.0813 | 0.3845 | 3.19E-15 |
| U49A | DTNA | Exon Skip | 31:32.1 | 0.3764 | 0.4355 | 3.95E-15 |
| U49A | CXCL12 | Alternate Terminator | 3.3 | 0.2292 | -0.3783 | 9.39E-15 |
| U49A | CAPZB | Exon Skip | 11 | 0.071 | -0.3744 | 1.87E-14 |
| U49A | ABCB5 | Alternate Terminator | 16 | 0.0606 | -0.3716 | 3.01E-14 |
| U49A | DCN | Alternate Terminator | 12 | 0.0814 | -0.3659 | 7.81E-14 |
| U49A | COX17 | Alternate Terminator | 7 | 0.0835 | -0.3645 | 9.89E-14 |
| U49A | COX17 | Alternate Terminator | 5 | 0.0831 | 0.3603 | 1.99E-13 |
| U49A | ATP2A2 | Retained Intron | 21.2:21.3 | 0.0729 | 0.3601 | 2.04E-13 |
| U49A | DBN1 | Exon Skip | 14 | 0.0798 | -0.3618 | 2.20E-13 |
| U49A | CXCL12 | Alternate Terminator | 5.2 | 0.1851 | 0.3586 | 2.61E-13 |
| U49A | LRRFIP1 | Alternate Terminator | 21 | 0.1319 | 0.3562 | 3.84E-13 |
| U49A | LRRFIP1 | Alternate Terminator | 26 | 0.1319 | -0.3562 | 3.85E-13 |
| U49A | ACTG1 | Exon Skip | 3 | 0.0195 | -0.355 | 4.65E-13 |
| U49A | PTGER3 | Alternate Terminator | 12 | 0.1315 | -0.3511 | 8.68E-13 |
| U49A | FLNA | Exon Skip | 30 | 0.1752 | 0.3524 | 8.73E-13 |
| U49A | GPNMB | Alternate Terminator | 11 | 0.0756 | -0.3485 | 1.33E-12 |
| U49A | GPNMB | Alternate Terminator | 4.2 | 0.0756 | 0.3485 | 1.33E-12 |
| U49A | CSMD2 | Alternate Terminator | 78 | 0.112 | -0.3471 | 1.64E-12 |
| U49A | TAGLN | Alternate Donor Sites | 1.2:1.3 | 0.0477 | 0.3467 | 1.75E-12 |
| U49A | PDLIM7 | Exon Skip | 6 | 0.0862 | -0.3458 | 2.14E-12 |
| U49A | CYP46A1 | Alternate Terminator | 17 | 0.259 | -0.3445 | 2.62E-12 |
| U49A | CYP46A1 | Alternate Terminator | 9.2 | 0.259 | 0.3445 | 2.62E-12 |
| U49A | KIAA0930 | Alternate Promoter | 5.1 | 0.1193 | -0.3462 | 3.37E-12 |
| U49A | SPAG9 | Exon Skip | 30 | 0.1851 | -0.3438 | 4.04E-12 |
| U49A | KLC1 | Alternate Terminator | 19 | 0.1194 | -0.3361 | 8.91E-12 |
| U49A | TMEM108 | Alternate Terminator | 8 | 0.2977 | -0.3349 | 1.06E-11 |
| U49A | TMEM108 | Alternate Terminator | 6 | 0.2977 | 0.3349 | 1.06E-11 |
| U49A | CLDND1 | Alternate Terminator | 8 | 0.0234 | -0.334 | 1.21E-11 |
| U49A | CLDND1 | Alternate Terminator | 7 | 0.025 | 0.3323 | 1.55E-11 |
| U49A | TBC1D8B | Alternate Terminator | 7.2 | 0.1035 | 0.3311 | 1.86E-11 |
| U49A | CIRBP | Alternate Acceptor Sites | 9.5 | 0.0102 | 0.3293 | 2.44E-11 |
| U49A | JAM2 | Alternate Terminator | 12 | 0.1602 | -0.3293 | 2.44E-11 |
| U49A | JAM2 | Alternate Terminator | 11 | 0.1602 | 0.3293 | 2.44E-11 |
| U49A | PALLD | Alternate Terminator | 10 | 0.0443 | 0.3284 | 2.77E-11 |
| U49A | PALLD | Alternate Terminator | 26 | 0.0443 | -0.3284 | 2.77E-11 |
| U49A | VSTM4 | Alternate Terminator | 9 | 0.1472 | -0.3271 | 3.33E-11 |
| U49A | VSTM4 | Alternate Terminator | 3 | 0.1472 | 0.3271 | 3.33E-11 |
| U49A | TBC1D8B | Alternate Terminator | 22 | 0.0816 | -0.327 | 3.38E-11 |
| U49A | TAGLN | Retained Intron | 2.2 | 0.0608 | 0.3271 | 3.75E-11 |
| U49A | CLCN6 | Alternate Terminator | 6 | 0.0803 | 0.3248 | 4.65E-11 |
| U49A | 2-Sep | Alternate Promoter | 1 | 0.0592 | 0.3243 | 5.64E-11 |
| U49A | 2-Sep | Alternate Promoter | 2 | 0.0592 | -0.3243 | 5.64E-11 |
| U49A | CSMD2 | Alternate Terminator | 50 | 0.2016 | 0.3225 | 6.48E-11 |
| U49A | SVIL | Exon Skip | 10:11:12 | 0.2657 | -0.3449 | 6.62E-11 |
| U49A | KIAA0930 | Alternate Promoter | 1 | 0.1234 | 0.3258 | 6.73E-11 |
| U49A | FLNB | Exon Skip | 41 | 0.0767 | -0.323 | 7.14E-11 |
| U49A | TTC7B | Exon Skip | 18 | 0.1506 | -0.3274 | 8.58E-11 |
| U49A | TES | Alternate Promoter | 1 | 0.0559 | 0.3207 | 9.92E-11 |
| U49A | TES | Alternate Promoter | 2 | 0.0559 | -0.3207 | 9.92E-11 |
| U49A | SMIM11 | Alternate Terminator | 5 | 0.0676 | 0.3194 | 1.02E-10 |
| U49A | SMIM11 | Alternate Terminator | 7 | 0.0676 | -0.3194 | 1.02E-10 |
| U49A | TAF6 | Alternate Promoter | 1 | 0.1305 | -0.3181 | 1.21E-10 |
| U49A | PRKAR1B | Alternate Promoter | 1 | 0.1843 | 0.3302 | 1.24E-10 |
| U49A | CNTLN | Alternate Terminator | 7.3 | 0.1471 | 0.3156 | 1.72E-10 |
| U49A | BLOC1S6 | Alternate Terminator | 9 | 0.1426 | -0.3156 | 1.73E-10 |
| U49A | BLOC1S6 | Alternate Terminator | 4.2 | 0.1426 | 0.3156 | 1.73E-10 |
| U49A | SCD5 | Alternate Terminator | 6 | 0.1107 | 0.3151 | 1.84E-10 |
| U49A | CLCN6 | Alternate Terminator | 24 | 0.0832 | -0.3149 | 1.89E-10 |
| U49A | CD63 | Alternate Promoter | 3.1 | 0.0196 | 0.3144 | 2.02E-10 |
| U49A | UCK1 | Exon Skip | 6 | 0.0531 | -0.3153 | 2.23E-10 |
| U49A | CAMK2G | Exon Skip | 13 | 0.0669 | -0.3158 | 3.17E-10 |
| U49A | THOC5 | Exon Skip | 11 | 0.1813 | -0.3116 | 3.50E-10 |
| U49A | PTGER3 | Alternate Terminator | 8 | 0.2932 | 0.3096 | 3.95E-10 |
| U49A | CNTLN | Alternate Terminator | 27 | 0.1421 | -0.309 | 4.28E-10 |
| U49A | ATP5C1 | Exon Skip | 9 | 0.0667 | 0.3087 | 4.44E-10 |
| U49A | DMPK | Alternate Promoter | 2.1 | 0.1582 | 0.3078 | 5.04E-10 |
| U49A | DMPK | Alternate Promoter | 1 | 0.1582 | -0.3078 | 5.04E-10 |
| U49A | PTGIR | Alternate Terminator | 4.2 | 0.2147 | -0.3071 | 5.51E-10 |
| U49A | PTGIR | Alternate Terminator | 3 | 0.2147 | 0.3071 | 5.51E-10 |
| U49A | TPM1 | Alternate Promoter | 5.1 | 0.1086 | 0.3059 | 6.49E-10 |
| U49A | UBTF | Alternate Promoter | 5.1 | 0.0941 | 0.3062 | 6.85E-10 |
| U49A | ARHGEF11 | Exon Skip | 39 | 0.2057 | -0.3105 | 6.87E-10 |
| U49A | VDAC3 | Exon Skip | 5.2 | 0.0766 | -0.3044 | 8.31E-10 |
| U49A | FBLN1 | Alternate Terminator | 16 | 0.0382 | 0.3037 | 8.72E-10 |
| U49A | CIRBP | Exon Skip | 9.6 | 0.0231 | -0.3042 | 8.94E-10 |
| U49A | GSDMD | Alternate Promoter | 4 | 0.1435 | 0.3025 | 1.02E-09 |
| U49A | MBP | Alternate Terminator | 14.2 | 0.1034 | -0.3023 | 1.05E-09 |
| U49A | ATP8B2 | Alternate Terminator | 16 | 0.1182 | 0.3022 | 1.06E-09 |
| U49A | KRT222 | Alternate Terminator | 12 | 0.0188 | 0.3022 | 1.06E-09 |
| U49A | MBP | Alternate Terminator | 5.5 | 0.1042 | 0.3017 | 1.14E-09 |
| U49A | PPP1R14A | Alternate Promoter | 2.1 | 0.1586 | -0.3021 | 1.19E-09 |
| U49A | PPP1R14A | Alternate Promoter | 1 | 0.1586 | 0.3021 | 1.19E-09 |
| U49A | BEND6 | Alternate Terminator | 10 | 0.1221 | -0.301 | 1.44E-09 |
| U49A | BEND6 | Alternate Terminator | 4 | 0.1221 | 0.301 | 1.44E-09 |
| U49A | PODNL1 | Alternate Promoter | 2 | 0.2622 | -0.3109 | 2.27E-09 |
| U49A | CSNK1G3 | Exon Skip | 13 | 0.1879 | -0.3073 | 3.02E-09 |
| U49A | SPATA9 | Alternate Terminator | 10 | 0.26 | 0.3003 | 3.78E-09 |
| U49A | NNMT | Alternate Promoter | 1 | 0.0985 | -0.3241 | 3.87E-09 |
| U49A | CRAT | Exon Skip | 2:03 | 0.1605 | -0.3136 | 5.19E-09 |
| U49A | B4GALT2 | Alternate Promoter | 2.1 | 0.2221 | 0.3076 | 5.40E-09 |
| U49A | B4GALT2 | Alternate Promoter | 1 | 0.2221 | -0.3076 | 5.40E-09 |
| U49A | CDIP1 | Alternate Promoter | 2 | 0.115 | -0.3033 | 5.49E-09 |
| U49A | CABIN1 | Alternate Promoter | 31 | 0.2375 | -0.302 | 5.81E-09 |
| U49A | ARHGEF25 | Alternate Promoter | 1 | 0.1588 | -0.3025 | 7.66E-09 |
| U49A | ARHGEF25 | Alternate Promoter | 3 | 0.1588 | 0.3025 | 7.66E-09 |
| U49A | FBLN5 | Alternate Promoter | 1 | 0.0727 | 0.3036 | 9.58E-09 |
| U49A | FBLN5 | Alternate Promoter | 2 | 0.0727 | -0.3036 | 9.58E-09 |
| U49A | POSTN | Exon Skip | 17:18 | 0.1084 | -0.3105 | 4.43E-08 |
| U49A | CD44 | Exon Skip | 10:11:12.1:13:14 | 0.2495 | 0.3013 | 1.25E-07 |
| U49A | CAMK2D | Exon Skip | 17 | 0.1588 | -0.3015 | 1.50E-07 |
