# Supplementary material for: Small nucleolar RNAs signature (SNORS) identified clinical outcome and prognosis of bladder cancer (BLCA)
Source: Cancer Cell Int. 2020 Jul 10;20:299. doi: 10.1186/s12935-020-01393-7 (PMC7350589; doi:10.1186/s12935-020-01393-7)
Supplement: Supplementary file 19 — Additional file 19: Table S17. Spearman correlation analyses for candidate snoRNAs and relevant signature with Hallmark gene-sets in TCGA-BLCA cohort. [file 12935_2020_1393_MOESM19_ESM.docx]

**Additional file 19: Table S17 Spearman correlation analyses for candidate snoRNAs and relevant signature with Hallmark gene-sets in TCGA-BLCA cohort (n = 392)**

| id | HALLMARK gene-sets | cor | p.value |
| --- | --- | --- | --- |
| SNORD113-9 | HALLMARK_ADIPOGENESIS | 0.138409452 | 0.006527 |
| SNORD113-9 | HALLMARK_ALLOGRAFT_REJECTION | 0.193756326 | 0.00013 |
| SNORD113-9 | HALLMARK_ANDROGEN_RESPONSE | 0.173875312 | 0.000611 |
| SNORD113-9 | HALLMARK_ANGIOGENESIS | 0.139490091 | 0.006116 |
| SNORD113-9 | HALLMARK_APICAL_JUNCTION | 0.265055829 | 1.30E-07 |
| SNORD113-9 | HALLMARK_APICAL_SURFACE | 0.135901102 | 0.007578 |
| SNORD113-9 | HALLMARK_APOPTOSIS | 0.150129523 | 0.003148 |
| SNORD113-9 | HALLMARK_BILE_ACID_METABOLISM | 0.064053603 | 0.209833 |
| SNORD113-9 | HALLMARK_CHOLESTEROL_HOMEOSTASIS | 0.030044262 | 0.556716 |
| SNORD113-9 | HALLMARK_COAGULATION | 0.046702914 | 0.360771 |
| SNORD113-9 | HALLMARK_COMPLEMENT | 0.292695621 | 4.82E-09 |
| SNORD113-9 | HALLMARK_DNA_REPAIR | -0.148936278 | 0.003398 |
| SNORD113-9 | HALLMARK_E2F_TARGETS | 0.005954978 | 0.907283 |
| SNORD113-9 | HALLMARK_EPITHELIAL_MESENCHYMAL_TRANSITION | 0.37428307 | 3.01E-14 |
| SNORD113-9 | HALLMARK_ESTROGEN_RESPONSE_EARLY | 0.059917881 | 0.240834 |
| SNORD113-9 | HALLMARK_ESTROGEN_RESPONSE_LATE | -0.012750874 | 0.803062 |
| SNORD113-9 | HALLMARK_FATTY_ACID_METABOLISM | -0.052816445 | 0.301283 |
| SNORD113-9 | HALLMARK_G2M_CHECKPOINT | 0.069929095 | 0.170898 |
| SNORD113-9 | HALLMARK_GLYCOLYSIS | 0.114177491 | 0.025067 |
| SNORD113-9 | HALLMARK_HEDGEHOG_SIGNALING | 0.274325694 | 4.49E-08 |
| SNORD113-9 | HALLMARK_HEME_METABOLISM | 0.124239699 | 0.014716 |
| SNORD113-9 | HALLMARK_HYPOXIA | 0.128016155 | 0.011936 |
| SNORD113-9 | HALLMARK_IL2_STAT5_SIGNALING | 0.305948018 | 8.72E-10 |
| SNORD113-9 | HALLMARK_IL6_JAK_STAT3_SIGNALING | 0.18353567 | 0.000294 |
| SNORD113-9 | HALLMARK_INFLAMMATORY_RESPONSE | 0.307080597 | 7.50E-10 |
| SNORD113-9 | HALLMARK_INTERFERON_ALPHA_RESPONSE | 0.047671578 | 0.350886 |
| SNORD113-9 | HALLMARK_INTERFERON_GAMMA_RESPONSE | 0.155973893 | 0.002146 |
| SNORD113-9 | HALLMARK_KRAS_SIGNALING_DN | 0.107626048 | 0.034769 |
| SNORD113-9 | HALLMARK_KRAS_SIGNALING_UP | 0.331018373 | 2.69E-11 |
| SNORD113-9 | HALLMARK_MITOTIC_SPINDLE | 0.102887026 | 0.043635 |
| SNORD113-9 | HALLMARK_MTORC1_SIGNALING | 0.129712395 | 0.010846 |
| SNORD113-9 | HALLMARK_MYC_TARGETS_V1 | -0.032944473 | 0.519258 |
| SNORD113-9 | HALLMARK_MYC_TARGETS_V2 | 0.010819013 | 0.832419 |
| SNORD113-9 | HALLMARK_MYOGENESIS | 0.144235842 | 0.004572 |
| SNORD113-9 | HALLMARK_NOTCH_SIGNALING | 0.254989205 | 3.96E-07 |
| SNORD113-9 | HALLMARK_OXIDATIVE_PHOSPHORYLATION | -0.17373253 | 0.000617 |
| SNORD113-9 | HALLMARK_P53_PATHWAY | -0.071070822 | 0.164002 |
| SNORD113-9 | HALLMARK_PANCREAS_BETA_CELLS | 0.150389958 | 0.003095 |
| SNORD113-9 | HALLMARK_PEROXISOME | -0.058823993 | 0.249545 |
| SNORD113-9 | HALLMARK_PI3K_AKT_MTOR_SIGNALING | 0.075828454 | 0.137501 |
| SNORD113-9 | HALLMARK_PROTEIN_SECRETION | 0.136500721 | 0.007314 |
| SNORD113-9 | HALLMARK_REACTIVE_OXIGEN_SPECIES_PATHWAY | -0.039500437 | 0.439619 |
| SNORD113-9 | HALLMARK_SPERMATOGENESIS | 0.087498268 | 0.086428 |
| SNORD113-9 | HALLMARK_TGF_BETA_SIGNALING | 0.197262779 | 9.77E-05 |
| SNORD113-9 | HALLMARK_TNFA_SIGNALING_VIA_NFKB | 0.211502976 | 2.87E-05 |
| SNORD113-9 | HALLMARK_UNFOLDED_PROTEIN_RESPONSE | 0.117152352 | 0.021497 |
| SNORD113-9 | HALLMARK_UV_RESPONSE_DN | 0.253581682 | 4.60E-07 |
| SNORD113-9 | HALLMARK_UV_RESPONSE_UP | 0.049752635 | 0.330232 |
| SNORD113-9 | HALLMARK_WNT_BETA_CATENIN_SIGNALING | -0.016608815 | 0.745294 |
| SNORD113-9 | HALLMARK_XENOBIOTIC_METABOLISM | -0.108664736 | 0.033045 |
| SNORD114-1 | HALLMARK_ADIPOGENESIS | -0.036770873 | 0.471898 |
| SNORD114-1 | HALLMARK_ALLOGRAFT_REJECTION | -0.20288055 | 6.08E-05 |
| SNORD114-1 | HALLMARK_ANDROGEN_RESPONSE | 0.038435825 | 0.452056 |
| SNORD114-1 | HALLMARK_ANGIOGENESIS | -0.196101301 | 0.000108 |
| SNORD114-1 | HALLMARK_APICAL_JUNCTION | -0.269732946 | 7.65E-08 |
| SNORD114-1 | HALLMARK_APICAL_SURFACE | -0.08517338 | 0.095153 |
| SNORD114-1 | HALLMARK_APOPTOSIS | -0.091748544 | 0.072149 |
| SNORD114-1 | HALLMARK_BILE_ACID_METABOLISM | -0.078674655 | 0.123298 |
| SNORD114-1 | HALLMARK_CHOLESTEROL_HOMEOSTASIS | -0.09611201 | 0.059553 |
| SNORD114-1 | HALLMARK_COAGULATION | -0.281680851 | 1.87E-08 |
| SNORD114-1 | HALLMARK_COMPLEMENT | -0.15833703 | 0.001831 |
| SNORD114-1 | HALLMARK_DNA_REPAIR | 0.100331324 | 0.049159 |
| SNORD114-1 | HALLMARK_E2F_TARGETS | 0.10330563 | 0.042782 |
| SNORD114-1 | HALLMARK_EPITHELIAL_MESENCHYMAL_TRANSITION | -0.255060556 | 3.93E-07 |
| SNORD114-1 | HALLMARK_ESTROGEN_RESPONSE_EARLY | 0.041193325 | 0.420251 |
| SNORD114-1 | HALLMARK_ESTROGEN_RESPONSE_LATE | -0.049394327 | 0.333731 |
| SNORD114-1 | HALLMARK_FATTY_ACID_METABOLISM | 0.006956206 | 0.891783 |
| SNORD114-1 | HALLMARK_G2M_CHECKPOINT | 0.161272328 | 0.001499 |
| SNORD114-1 | HALLMARK_GLYCOLYSIS | -0.023725583 | 0.64259 |
| SNORD114-1 | HALLMARK_HEDGEHOG_SIGNALING | -0.10718554 | 0.035522 |
| SNORD114-1 | HALLMARK_HEME_METABOLISM | -0.020865883 | 0.683177 |
| SNORD114-1 | HALLMARK_HYPOXIA | -0.098340427 | 0.053858 |
| SNORD114-1 | HALLMARK_IL2_STAT5_SIGNALING | -0.146610978 | 0.00394 |
| SNORD114-1 | HALLMARK_IL6_JAK_STAT3_SIGNALING | -0.051310677 | 0.315294 |
| SNORD114-1 | HALLMARK_INFLAMMATORY_RESPONSE | -0.209349309 | 3.47E-05 |
| SNORD114-1 | HALLMARK_INTERFERON_ALPHA_RESPONSE | -0.024522467 | 0.63146 |
| SNORD114-1 | HALLMARK_INTERFERON_GAMMA_RESPONSE | -0.113965419 | 0.02534 |
| SNORD114-1 | HALLMARK_KRAS_SIGNALING_DN | -0.139411364 | 0.006145 |
| SNORD114-1 | HALLMARK_KRAS_SIGNALING_UP | -0.22321735 | 9.80E-06 |
| SNORD114-1 | HALLMARK_MITOTIC_SPINDLE | 0.075996959 | 0.136626 |
| SNORD114-1 | HALLMARK_MTORC1_SIGNALING | 0.093101639 | 0.06803 |
| SNORD114-1 | HALLMARK_MYC_TARGETS_V1 | 0.075710442 | 0.138116 |
| SNORD114-1 | HALLMARK_MYC_TARGETS_V2 | 0.114556958 | 0.024585 |
| SNORD114-1 | HALLMARK_MYOGENESIS | -0.296327271 | 3.05E-09 |
| SNORD114-1 | HALLMARK_NOTCH_SIGNALING | -0.117771308 | 0.020812 |
| SNORD114-1 | HALLMARK_OXIDATIVE_PHOSPHORYLATION | 0.010073928 | 0.843806 |
| SNORD114-1 | HALLMARK_P53_PATHWAY | 0.018626531 | 0.715617 |
| SNORD114-1 | HALLMARK_PANCREAS_BETA_CELLS | -0.079167358 | 0.120959 |
| SNORD114-1 | HALLMARK_PEROXISOME | 0.023267787 | 0.64902 |
| SNORD114-1 | HALLMARK_PI3K_AKT_MTOR_SIGNALING | 0.101072167 | 0.047501 |
| SNORD114-1 | HALLMARK_PROTEIN_SECRETION | 0.073834677 | 0.148176 |
| SNORD114-1 | HALLMARK_REACTIVE_OXIGEN_SPECIES_PATHWAY | -0.113756394 | 0.025611 |
| SNORD114-1 | HALLMARK_SPERMATOGENESIS | 0.16204892 | 0.001421 |
| SNORD114-1 | HALLMARK_TGF_BETA_SIGNALING | -0.002158076 | 0.966334 |
| SNORD114-1 | HALLMARK_TNFA_SIGNALING_VIA_NFKB | -0.123689429 | 0.015166 |
| SNORD114-1 | HALLMARK_UNFOLDED_PROTEIN_RESPONSE | 0.119498504 | 0.019001 |
| SNORD114-1 | HALLMARK_UV_RESPONSE_DN | -0.048467275 | 0.342894 |
| SNORD114-1 | HALLMARK_UV_RESPONSE_UP | -0.06561538 | 0.198909 |
| SNORD114-1 | HALLMARK_WNT_BETA_CATENIN_SIGNALING | 0.122021849 | 0.016602 |
| SNORD114-1 | HALLMARK_XENOBIOTIC_METABOLISM | -0.004485327 | 0.930097 |
| U3 | HALLMARK_ADIPOGENESIS | 0.050535809 | 0.322667 |
| U3 | HALLMARK_ALLOGRAFT_REJECTION | -0.067535846 | 0.186052 |
| U3 | HALLMARK_ANDROGEN_RESPONSE | 0.022067728 | 0.666 |
| U3 | HALLMARK_ANGIOGENESIS | -0.276790083 | 3.36E-08 |
| U3 | HALLMARK_APICAL_JUNCTION | -0.264930284 | 1.32E-07 |
| U3 | HALLMARK_APICAL_SURFACE | -0.117514198 | 0.021094 |
| U3 | HALLMARK_APOPTOSIS | -0.121397475 | 0.017169 |
| U3 | HALLMARK_BILE_ACID_METABOLISM | -0.091812166 | 0.071951 |
| U3 | HALLMARK_CHOLESTEROL_HOMEOSTASIS | -0.066828563 | 0.190714 |
| U3 | HALLMARK_COAGULATION | -0.188813356 | 0.000194 |
| U3 | HALLMARK_COMPLEMENT | -0.096719096 | 0.057954 |
| U3 | HALLMARK_DNA_REPAIR | 0.059915753 | 0.240851 |
| U3 | HALLMARK_E2F_TARGETS | 0.164523893 | 0.001196 |
| U3 | HALLMARK_EPITHELIAL_MESENCHYMAL_TRANSITION | -0.227835069 | 6.32E-06 |
| U3 | HALLMARK_ESTROGEN_RESPONSE_EARLY | 0.036031398 | 0.480862 |
| U3 | HALLMARK_ESTROGEN_RESPONSE_LATE | 0.011038122 | 0.829077 |
| U3 | HALLMARK_FATTY_ACID_METABOLISM | 0.047584291 | 0.351769 |
| U3 | HALLMARK_G2M_CHECKPOINT | 0.205055957 | 5.04E-05 |
| U3 | HALLMARK_GLYCOLYSIS | 0.042979431 | 0.400368 |
| U3 | HALLMARK_HEDGEHOG_SIGNALING | -0.090011206 | 0.077734 |
| U3 | HALLMARK_HEME_METABOLISM | -0.061761293 | 0.226641 |
| U3 | HALLMARK_HYPOXIA | -0.097991407 | 0.054719 |
| U3 | HALLMARK_IL2_STAT5_SIGNALING | -0.10143509 | 0.046705 |
| U3 | HALLMARK_IL6_JAK_STAT3_SIGNALING | -0.1554988 | 0.002215 |
| U3 | HALLMARK_INFLAMMATORY_RESPONSE | -0.131415414 | 0.009841 |
| U3 | HALLMARK_INTERFERON_ALPHA_RESPONSE | 0.014670744 | 0.774158 |
| U3 | HALLMARK_INTERFERON_GAMMA_RESPONSE | -0.104632428 | 0.04017 |
| U3 | HALLMARK_KRAS_SIGNALING_DN | -0.098625303 | 0.053163 |
| U3 | HALLMARK_KRAS_SIGNALING_UP | -0.120949469 | 0.017587 |
| U3 | HALLMARK_MITOTIC_SPINDLE | 0.028840806 | 0.572635 |
| U3 | HALLMARK_MTORC1_SIGNALING | 0.189770559 | 0.00018 |
| U3 | HALLMARK_MYC_TARGETS_V1 | 0.238885072 | 2.13E-06 |
| U3 | HALLMARK_MYC_TARGETS_V2 | 0.22753773 | 6.50E-06 |
| U3 | HALLMARK_MYOGENESIS | -0.402775281 | 1.90E-16 |
| U3 | HALLMARK_NOTCH_SIGNALING | -0.20057979 | 7.39E-05 |
| U3 | HALLMARK_OXIDATIVE_PHOSPHORYLATION | 0.097055127 | 0.057085 |
| U3 | HALLMARK_P53_PATHWAY | 0.071597022 | 0.160895 |
| U3 | HALLMARK_PANCREAS_BETA_CELLS | 0.047976362 | 0.347811 |
| U3 | HALLMARK_PEROXISOME | 0.066308746 | 0.194195 |
| U3 | HALLMARK_PI3K_AKT_MTOR_SIGNALING | 0.086418118 | 0.090398 |
| U3 | HALLMARK_PROTEIN_SECRETION | 0.085396883 | 0.094285 |
| U3 | HALLMARK_REACTIVE_OXIGEN_SPECIES_PATHWAY | -0.102367901 | 0.044713 |
| U3 | HALLMARK_SPERMATOGENESIS | 0.078484073 | 0.124212 |
| U3 | HALLMARK_TGF_BETA_SIGNALING | 0.096488732 | 0.058557 |
| U3 | HALLMARK_TNFA_SIGNALING_VIA_NFKB | -0.10944326 | 0.031801 |
| U3 | HALLMARK_UNFOLDED_PROTEIN_RESPONSE | 0.177829276 | 0.000455 |
| U3 | HALLMARK_UV_RESPONSE_DN | -0.15493146 | 0.0023 |
| U3 | HALLMARK_UV_RESPONSE_UP | 0.026262854 | 0.607443 |
| U3 | HALLMARK_WNT_BETA_CATENIN_SIGNALING | 0.118868203 | 0.019645 |
| U3 | HALLMARK_XENOBIOTIC_METABOLISM | -0.066083744 | 0.195715 |
| SNORD19B | HALLMARK_ADIPOGENESIS | -0.148207603 | 0.00356 |
| SNORD19B | HALLMARK_ALLOGRAFT_REJECTION | -0.296899171 | 2.83E-09 |
| SNORD19B | HALLMARK_ANDROGEN_RESPONSE | -0.122123802 | 0.016511 |
| SNORD19B | HALLMARK_ANGIOGENESIS | -0.262476847 | 1.74E-07 |
| SNORD19B | HALLMARK_APICAL_JUNCTION | -0.452100018 | 8.59E-21 |
| SNORD19B | HALLMARK_APICAL_SURFACE | -0.040726369 | 0.425543 |
| SNORD19B | HALLMARK_APOPTOSIS | -0.321684376 | 1.02E-10 |
| SNORD19B | HALLMARK_BILE_ACID_METABOLISM | 0.050408699 | 0.323887 |
| SNORD19B | HALLMARK_CHOLESTEROL_HOMEOSTASIS | -0.047932628 | 0.348251 |
| SNORD19B | HALLMARK_COAGULATION | -0.25997796 | 2.29E-07 |
| SNORD19B | HALLMARK_COMPLEMENT | -0.392306748 | 1.29E-15 |
| SNORD19B | HALLMARK_DNA_REPAIR | 0.008386993 | 0.869706 |
| SNORD19B | HALLMARK_E2F_TARGETS | -0.074290006 | 0.145684 |
| SNORD19B | HALLMARK_EPITHELIAL_MESENCHYMAL_TRANSITION | -0.406946477 | 8.66E-17 |
| SNORD19B | HALLMARK_ESTROGEN_RESPONSE_EARLY | 0.15340977 | 0.002543 |
| SNORD19B | HALLMARK_ESTROGEN_RESPONSE_LATE | -0.048486511 | 0.342703 |
| SNORD19B | HALLMARK_FATTY_ACID_METABOLISM | 0.159406803 | 0.001703 |
| SNORD19B | HALLMARK_G2M_CHECKPOINT | -0.112663109 | 0.027073 |
| SNORD19B | HALLMARK_GLYCOLYSIS | -0.038180274 | 0.455071 |
| SNORD19B | HALLMARK_HEDGEHOG_SIGNALING | -0.226652677 | 7.08E-06 |
| SNORD19B | HALLMARK_HEME_METABOLISM | -0.234191708 | 3.40E-06 |
| SNORD19B | HALLMARK_HYPOXIA | -0.174652354 | 0.000577 |
| SNORD19B | HALLMARK_IL2_STAT5_SIGNALING | -0.34516394 | 3.27E-12 |
| SNORD19B | HALLMARK_IL6_JAK_STAT3_SIGNALING | -0.242277051 | 1.51E-06 |
| SNORD19B | HALLMARK_INFLAMMATORY_RESPONSE | -0.334074823 | 1.72E-11 |
| SNORD19B | HALLMARK_INTERFERON_ALPHA_RESPONSE | -0.178525013 | 0.000432 |
| SNORD19B | HALLMARK_INTERFERON_GAMMA_RESPONSE | -0.265057427 | 1.30E-07 |
| SNORD19B | HALLMARK_KRAS_SIGNALING_DN | -0.005992512 | 0.906701 |
| SNORD19B | HALLMARK_KRAS_SIGNALING_UP | -0.345583662 | 3.06E-12 |
| SNORD19B | HALLMARK_MITOTIC_SPINDLE | -0.233860093 | 3.51E-06 |
| SNORD19B | HALLMARK_MTORC1_SIGNALING | -0.148318001 | 0.003535 |
| SNORD19B | HALLMARK_MYC_TARGETS_V1 | -0.031181037 | 0.541878 |
| SNORD19B | HALLMARK_MYC_TARGETS_V2 | -0.063571512 | 0.213291 |
| SNORD19B | HALLMARK_MYOGENESIS | -0.281389926 | 1.94E-08 |
| SNORD19B | HALLMARK_NOTCH_SIGNALING | -0.151482746 | 0.002884 |
| SNORD19B | HALLMARK_OXIDATIVE_PHOSPHORYLATION | 0.024766595 | 0.628067 |
| SNORD19B | HALLMARK_P53_PATHWAY | -0.036138226 | 0.479561 |
| SNORD19B | HALLMARK_PANCREAS_BETA_CELLS | -0.110921238 | 0.02955 |
| SNORD19B | HALLMARK_PEROXISOME | 0.121114238 | 0.017432 |
| SNORD19B | HALLMARK_PI3K_AKT_MTOR_SIGNALING | -0.155239119 | 0.002253 |
| SNORD19B | HALLMARK_PROTEIN_SECRETION | -0.122099409 | 0.016532 |
| SNORD19B | HALLMARK_REACTIVE_OXIGEN_SPECIES_PATHWAY | -0.162318368 | 0.001395 |
| SNORD19B | HALLMARK_SPERMATOGENESIS | 0.048747471 | 0.340108 |
| SNORD19B | HALLMARK_TGF_BETA_SIGNALING | -0.295203036 | 3.51E-09 |
| SNORD19B | HALLMARK_TNFA_SIGNALING_VIA_NFKB | -0.302149597 | 1.44E-09 |
| SNORD19B | HALLMARK_UNFOLDED_PROTEIN_RESPONSE | -0.092949591 | 0.068483 |
| SNORD19B | HALLMARK_UV_RESPONSE_DN | -0.274510866 | 4.39E-08 |
| SNORD19B | HALLMARK_UV_RESPONSE_UP | -0.138022174 | 0.00668 |
| SNORD19B | HALLMARK_WNT_BETA_CATENIN_SIGNALING | -0.016069646 | 0.75329 |
| SNORD19B | HALLMARK_XENOBIOTIC_METABOLISM | 0.221046664 | 1.20E-05 |
| U49A | HALLMARK_ADIPOGENESIS | 0.175474186 | 0.000553 |
| U49A | HALLMARK_ALLOGRAFT_REJECTION | 0.116395683 | 0.022408 |
| U49A | HALLMARK_ANDROGEN_RESPONSE | 0.204690549 | 5.40E-05 |
| U49A | HALLMARK_ANGIOGENESIS | 0.045955016 | 0.368359 |
| U49A | HALLMARK_APICAL_JUNCTION | 0.182214984 | 0.000333 |
| U49A | HALLMARK_APICAL_SURFACE | -0.075744188 | 0.137892 |
| U49A | HALLMARK_APOPTOSIS | 0.218985305 | 1.53E-05 |
| U49A | HALLMARK_BILE_ACID_METABOLISM | -0.087629954 | 0.085953 |
| U49A | HALLMARK_CHOLESTEROL_HOMEOSTASIS | -0.027628945 | 0.588723 |
| U49A | HALLMARK_COAGULATION | 0.015450887 | 0.762396 |
| U49A | HALLMARK_COMPLEMENT | 0.266417553 | 1.25E-07 |
| U49A | HALLMARK_DNA_REPAIR | 0.061160925 | 0.231083 |
| U49A | HALLMARK_E2F_TARGETS | 0.200711174 | 7.56E-05 |
| U49A | HALLMARK_EPITHELIAL_MESENCHYMAL_TRANSITION | 0.213089631 | 2.60E-05 |
| U49A | HALLMARK_ESTROGEN_RESPONSE_EARLY | -0.038912884 | 0.446288 |
| U49A | HALLMARK_ESTROGEN_RESPONSE_LATE | -0.000888441 | 0.986132 |
| U49A | HALLMARK_FATTY_ACID_METABOLISM | -0.09055035 | 0.075973 |
| U49A | HALLMARK_G2M_CHECKPOINT | 0.28421056 | 1.60E-08 |
| U49A | HALLMARK_GLYCOLYSIS | 0.114779246 | 0.024353 |
| U49A | HALLMARK_HEDGEHOG_SIGNALING | 0.154326341 | 0.002417 |
| U49A | HALLMARK_HEME_METABOLISM | 0.23448687 | 3.52E-06 |
| U49A | HALLMARK_HYPOXIA | 0.121936192 | 0.016726 |
| U49A | HALLMARK_IL2_STAT5_SIGNALING | 0.232857185 | 4.13E-06 |
| U49A | HALLMARK_IL6_JAK_STAT3_SIGNALING | 0.172818535 | 0.000671 |
| U49A | HALLMARK_INFLAMMATORY_RESPONSE | 0.168717406 | 0.000902 |
| U49A | HALLMARK_INTERFERON_ALPHA_RESPONSE | 0.126272837 | 0.013201 |
| U49A | HALLMARK_INTERFERON_GAMMA_RESPONSE | 0.104760994 | 0.039963 |
| U49A | HALLMARK_KRAS_SIGNALING_DN | -0.070936512 | 0.164733 |
| U49A | HALLMARK_KRAS_SIGNALING_UP | 0.213968609 | 2.40E-05 |
| U49A | HALLMARK_MITOTIC_SPINDLE | 0.286118868 | 1.27E-08 |
| U49A | HALLMARK_MTORC1_SIGNALING | 0.284826896 | 1.49E-08 |
| U49A | HALLMARK_MYC_TARGETS_V1 | 0.165613014 | 0.001123 |
| U49A | HALLMARK_MYC_TARGETS_V2 | 0.194789844 | 0.000123 |
| U49A | HALLMARK_MYOGENESIS | -0.052532846 | 0.303744 |
| U49A | HALLMARK_NOTCH_SIGNALING | 0.090724253 | 0.075409 |
| U49A | HALLMARK_OXIDATIVE_PHOSPHORYLATION | -0.001937328 | 0.969763 |
| U49A | HALLMARK_P53_PATHWAY | 0.039936957 | 0.434406 |
| U49A | HALLMARK_PANCREAS_BETA_CELLS | 0.083200996 | 0.103076 |
| U49A | HALLMARK_PEROXISOME | -0.015026748 | 0.76873 |
| U49A | HALLMARK_PI3K_AKT_MTOR_SIGNALING | 0.242069427 | 1.66E-06 |
| U49A | HALLMARK_PROTEIN_SECRETION | 0.244902976 | 1.24E-06 |
| U49A | HALLMARK_REACTIVE_OXIGEN_SPECIES_PATHWAY | 0.028859935 | 0.57222 |
| U49A | HALLMARK_SPERMATOGENESIS | 0.136270481 | 0.007454 |
| U49A | HALLMARK_TGF_BETA_SIGNALING | 0.330894413 | 3.59E-11 |
| U49A | HALLMARK_TNFA_SIGNALING_VIA_NFKB | 0.173342137 | 0.000646 |
| U49A | HALLMARK_UNFOLDED_PROTEIN_RESPONSE | 0.259184274 | 2.77E-07 |
| U49A | HALLMARK_UV_RESPONSE_DN | 0.247439607 | 9.59E-07 |
| U49A | HALLMARK_UV_RESPONSE_UP | 0.1458831 | 0.004155 |
| U49A | HALLMARK_WNT_BETA_CATENIN_SIGNALING | 0.123639266 | 0.015254 |
| U49A | HALLMARK_XENOBIOTIC_METABOLISM | -0.210701669 | 3.20E-05 |
| riskscore | HALLMARK_ADIPOGENESIS | 0.053389664 | 0.29606 |
| riskscore | HALLMARK_ALLOGRAFT_REJECTION | 0.128499849 | 0.011615 |
| riskscore | HALLMARK_ANDROGEN_RESPONSE | -0.006477294 | 0.899192 |
| riskscore | HALLMARK_ANGIOGENESIS | 0.250421469 | 6.45E-07 |
| riskscore | HALLMARK_APICAL_JUNCTION | 0.290073373 | 6.70E-09 |
| riskscore | HALLMARK_APICAL_SURFACE | 0.056498152 | 0.26879 |
| riskscore | HALLMARK_APOPTOSIS | 0.07072167 | 0.166089 |
| riskscore | HALLMARK_BILE_ACID_METABOLISM | 0.101330727 | 0.046933 |
| riskscore | HALLMARK_CHOLESTEROL_HOMEOSTASIS | -0.032260908 | 0.527968 |
| riskscore | HALLMARK_COAGULATION | 0.196072128 | 0.000108 |
| riskscore | HALLMARK_COMPLEMENT | 0.20663249 | 4.40E-05 |
| riskscore | HALLMARK_DNA_REPAIR | -0.019362865 | 0.704891 |
| riskscore | HALLMARK_E2F_TARGETS | 0.001164719 | 0.981826 |
| riskscore | HALLMARK_EPITHELIAL_MESENCHYMAL_TRANSITION | 0.351356277 | 1.25E-12 |
| riskscore | HALLMARK_ESTROGEN_RESPONSE_EARLY | 0.00279691 | 0.956377 |
| riskscore | HALLMARK_ESTROGEN_RESPONSE_LATE | -0.013818931 | 0.786946 |
| riskscore | HALLMARK_FATTY_ACID_METABOLISM | -0.015315627 | 0.764517 |
| riskscore | HALLMARK_G2M_CHECKPOINT | -0.053238736 | 0.297429 |
| riskscore | HALLMARK_GLYCOLYSIS | 0.135761478 | 0.007641 |
| riskscore | HALLMARK_HEDGEHOG_SIGNALING | 0.200375774 | 7.52E-05 |
| riskscore | HALLMARK_HEME_METABOLISM | 0.100796035 | 0.048113 |
| riskscore | HALLMARK_HYPOXIA | 0.168798689 | 0.000884 |
| riskscore | HALLMARK_IL2_STAT5_SIGNALING | 0.226498657 | 7.18E-06 |
| riskscore | HALLMARK_IL6_JAK_STAT3_SIGNALING | 0.195215923 | 0.000116 |
| riskscore | HALLMARK_INFLAMMATORY_RESPONSE | 0.227276228 | 6.67E-06 |
| riskscore | HALLMARK_INTERFERON_ALPHA_RESPONSE | 0.04352771 | 0.39438 |
| riskscore | HALLMARK_INTERFERON_GAMMA_RESPONSE | 0.085553335 | 0.093681 |
| riskscore | HALLMARK_KRAS_SIGNALING_DN | 0.068341818 | 0.180842 |
| riskscore | HALLMARK_KRAS_SIGNALING_UP | 0.260529895 | 2.16E-07 |
| riskscore | HALLMARK_MITOTIC_SPINDLE | 0.048069373 | 0.346876 |
| riskscore | HALLMARK_MTORC1_SIGNALING | 0.06848746 | 0.179912 |
| riskscore | HALLMARK_MYC_TARGETS_V1 | -0.019887728 | 0.69728 |
| riskscore | HALLMARK_MYC_TARGETS_V2 | 0.000690799 | 0.989221 |
| riskscore | HALLMARK_MYOGENESIS | 0.249800533 | 6.89E-07 |
| riskscore | HALLMARK_NOTCH_SIGNALING | 0.256550616 | 3.34E-07 |
| riskscore | HALLMARK_OXIDATIVE_PHOSPHORYLATION | -0.070083505 | 0.169953 |
| riskscore | HALLMARK_P53_PATHWAY | -0.028277718 | 0.580157 |
| riskscore | HALLMARK_PANCREAS_BETA_CELLS | 0.062392346 | 0.221921 |
| riskscore | HALLMARK_PEROXISOME | 0.016200369 | 0.751349 |
| riskscore | HALLMARK_PI3K_AKT_MTOR_SIGNALING | -0.01357098 | 0.79068 |
| riskscore | HALLMARK_PROTEIN_SECRETION | -0.00987749 | 0.846814 |
| riskscore | HALLMARK_REACTIVE_OXIGEN_SPECIES_PATHWAY | 0.085399132 | 0.094276 |
| riskscore | HALLMARK_SPERMATOGENESIS | -0.037284946 | 0.465721 |
| riskscore | HALLMARK_TGF_BETA_SIGNALING | 0.033747668 | 0.509118 |
| riskscore | HALLMARK_TNFA_SIGNALING_VIA_NFKB | 0.210900412 | 3.02E-05 |
| riskscore | HALLMARK_UNFOLDED_PROTEIN_RESPONSE | 0.032762942 | 0.521564 |
| riskscore | HALLMARK_UV_RESPONSE_DN | 0.130157724 | 0.010574 |
| riskscore | HALLMARK_UV_RESPONSE_UP | 0.097777279 | 0.055252 |
| riskscore | HALLMARK_WNT_BETA_CATENIN_SIGNALING | -0.030920847 | 0.545257 |
| riskscore | HALLMARK_XENOBIOTIC_METABOLISM | 0.009718002 | 0.849258 |
