# Supplementary material for: Small nucleolar RNAs signature (SNORS) identified clinical outcome and prognosis of bladder cancer (BLCA)
Source: Cancer Cell Int. 2020 Jul 10;20:299. doi: 10.1186/s12935-020-01393-7 (PMC7350589; doi:10.1186/s12935-020-01393-7)
Supplement: Supplementary file 23 — Additional file 23: Table S18. Comparison of AUC values in Fig. 7. [file 12935_2020_1393_MOESM23_ESM.docx]

**Additional file 23: Table S18 Comparison of AUC values in Figure 7 (n = 336)**

Comparison of AUC values in Figure 7k

| Variable 1 | ALL Combine |
| --- | --- |
| Variable 2 | Clinical Combine |
| Variable 3 | SNORS |
| Variable 4 | Pathologic N stage |
| Variable 5 | Pathologic T stage |
| Variable 6 | Age |
| Classification variable | OS status |

| Sample size | 336 |
| --- | --- |
| Positive group ^a^ | 146 (43.45%) |
| Negative group ^b^ | 190 (56.55%) |

^a^ fustat = 1
^b^ fustat = 0

| Variable | AUC | SE ^a^ | 95% CI ^b^ |
| --- | --- | --- | --- |
| ALL Combine | 0.785 | 0.0253 | 0.737 to 0.829 |
| Clinical Combine | 0.698 | 0.0278 | 0.646 to 0.756 |
| SNORS | 0.692 | 0.0297 | 0.639 to 0.741 |
| Age | 0.645 | 0.0261 | 0.590 to 0.698 |
| Pathologic N stage | 0.607 | 0.0259 | 0.554 to 0.658 |
| Pathologic T stage | 0.581 | 0.0242 | 0.526 to 0.634 |

^a^ DeLong et al., 1988

^b^ Binomial exact

## Pairwise comparison of AUC values

| ALL Combine ~ Clinical Combine | |
| --- | --- |
| Difference between areas | 0.0873 |
| Standard Error ^a^ | 0.0194 |
| 95% Confidence Interval | 0.0494 to 0.125 |
| z statistic | 3.625 |
| Significance level | P < 0.0001 |
| ALL Combine ~ SNORS | |
| Difference between areas | 0.0935 |
| Standard Error ^a^ | 0.0284 |
| 95% Confidence Interval | 0.0381 to 0.150 |
| z statistic | 3.479 |
| Significance level | P < 0.0001 |
| ALL Combine ~ Age | |
| Difference between areas | 0.140 |
| Standard Error ^a^ | 0.0309 |
| 95% Confidence Interval | 0.0795 to 0.201 |
| z statistic | 6.127 |
| Significance level | P < 0.0001 |
| ALL Combine ~ Pathologic N stage | |
| Difference between areas | 0.178 |
| Standard Error ^a^ | 0.0378 |
| 95% Confidence Interval | 0.1312 to 0.225 |
| z statistic | 6.424 |
| Significance level | P < 0.0001 |
| ALL Combine ~ Pathologic T stage | |
| Difference between areas | 0.204 |
| Standard Error ^a^ | 0.0450 |
| 95% Confidence Interval | 0.155 to 0.253 |
| z statistic | 8.265 |
| Significance level | P < 0.0001 |
| Clinical Combine ~ SNORS | |
| Difference between areas | 0.0064 |
| Standard Error ^a^ | 0.0225 |
| 95% Confidence Interval | -0.1113 to 0.112 |
| z statistic | 0.668 |
| Significance level | P = 0.8941 |
| Clinical Combine ~ Age | |
| Difference between areas | 0.0536 |
| Standard Error ^a^ | 0.0288 |
| 95% Confidence Interval | -0.0036 to 0.113 |
| z statistic | 2.132 |
| Significance level | P = 0.028 |
| Clinical Combine ~ Pathologic N stage | |
| Difference between areas | 0.0912 |
| Standard Error ^a^ | 0.0169 |
| 95% Confidence Interval | 0.0579 to 0.1241 |
| z statistic | 3.473 |
| Significance level | P < 0.0001 |
| Clinical Combine ~ Pathologic T stage | |
| Difference between areas | 0.117 |
| Standard Error ^a^ | 0.0293 |
| 95% Confidence Interval | 0.0603 to 0.159 |
| z statistic | 5.281 |
| Significance level | P < 0.0001 |
| SNORS ~ Age | |
| Difference between areas | 0.0475 |
| Standard Error ^a^ | 0.0397 |
| 95% Confidence Interval | 0.026 to 0.068 |
| z statistic | 2.278 |
| Significance level | P = 0.0428 |
| SNORS ~ Pathologic N stage | |
| Difference between areas | 0.0852 |
| Standard Error ^a^ | 0.0418 |
| 95% Confidence Interval | 0.0286 to 0.145 |
| z statistic | 2.725 |
| Significance level | P = 0.0002 |
| SNORS ~ Pathologic T stage | |
| Difference between areas | 0.1115 |
| Standard Error ^a^ | 0.0263 |
| 95% Confidence Interval | 0.0597 to 0.163 |
| z statistic | 4.231 |
| Significance level | P < 0.0001 |
| Age ~ Pathologic N stage | |
| Difference between areas | 0.0385 |
| Standard Error ^a^ | 0.0377 |
| 95% Confidence Interval | -0.027 to 0.104 |
| z statistic | 1.595 |
| Significance level | P = 0.1108 |
| Age ~ Pathologic T stage | |
| Difference between areas | 0.0645 |
| Standard Error ^a^ | 0.0354 |
| 95% Confidence Interval | -0.0503 to 0.179 |
| z statistic | 0.217 |
| Significance level | P = 0.0009 |
| Pathologic N stage ~ Pathologic T stage | |
| Difference between areas | 0.0525 |
| Standard Error ^a^ | 0.0305 |
| 95% Confidence Interval | -0.007 to 0.112 |
| z statistic | 1.720 |
| Significance level | P = 0.8854 |

^a^ DeLong et al., 1988

Comparison of AUC values in Figure 8l

| Variable 1 | ALL Combine |
| --- | --- |
| Variable 2 | Clinical Combine |
| Variable 3 | SNORS |
| Variable 4 | Pathologic N stage |
| Variable 5 | Pathologic T stage |
| Variable 6 | Age |
| Classification variable | OS status |

| Sample size | 336 |
| --- | --- |
| Positive group ^a^ | 146 (43.45%) |
| Negative group ^b^ | 190 (56.55%) |

^a^ fustat = 1
^b^ fustat = 0

| Variable | AUC | SE ^a^ | 95% CI ^b^ |
| --- | --- | --- | --- |
| ALL Combine | 0.760 | 0.0253 | 0.714 to 0.806 |
| Clinical Combine | 0.684 | 0.0278 | 0.633 to 0.735 |
| SNORS | 0.682 | 0.0297 | 0.629 to 0.733 |
| Pathologic N stage | 0.622 | 0.0259 | 0.615 to 0.719 |
| Pathologic T stage | 0.595 | 0.0242 | 0.540 to 0.649 |
| Age | 0.580 | 0.0261 | 0.525 to 0.634 |

^a^ DeLong et al., 1988

^b^ Binomial exact

## Pairwise comparison of AUC values

| ALL Combine ~ Clinical Combine | |
| --- | --- |
| Difference between areas | 0.0761 |
| Standard Error ^a^ | 0.0214 |
| 95% Confidence Interval | 0.0276 to 0.124 |
| z statistic | 3.625 |
| Significance level | P = 0.0002 |
| ALL Combine ~ SNORS | |
| Difference between areas | 0.0782 |
| Standard Error ^a^ | 0.0264 |
| 95% Confidence Interval | 0.0224 to 0.133 |
| z statistic | 3.479 |
| Significance level | P = 0.0009 |
| ALL Combine ~ Pathologic N stage | |
| Difference between areas | 0.138 |
| Standard Error ^a^ | 0.0248 |
| 95% Confidence Interval | 0.0952 to 0.181 |
| z statistic | 5.424 |
| Significance level | P < 0.0001 |
| ALL Combine ~ Pathologic T stage | |
| Difference between areas | 0.165 |
| Standard Error ^a^ | 0.0256 |
| 95% Confidence Interval | 0.116 to 0.214 |
| z statistic | 7.265 |
| Significance level | P < 0.0001 |
| ALL Combine ~ Age | |
| Difference between areas | 0.180 |
| Standard Error ^a^ | 0.0319 |
| 95% Confidence Interval | 0.120 to 0.239 |
| z statistic | 6.127 |
| Significance level | P < 0.0001 |
| Clinical Combine ~ SNORS | |
| Difference between areas | 0.0002 |
| Standard Error ^a^ | 0.0325 |
| 95% Confidence Interval | -0.0514 to 0.052 |
| z statistic | 0.668 |
| Significance level | P = 0.9541 |
| Clinical Combine ~ Pathologic N stage | |
| Difference between areas | 0.0622 |
| Standard Error ^a^ | 0.0239 |
| 95% Confidence Interval | 0.0290 to 0.0943 |
| z statistic | 3.173 |
| Significance level | P = 0.0002 |
| Clinical Combine ~ Pathologic T stage | |
| Difference between areas | 0.0893 |
| Standard Error ^a^ | 0.0163 |
| 95% Confidence Interval | 0.0575 to 0.121 |
| z statistic | 4.231 |
| Significance level | P < 0.0001 |
| Clinical Combine ~ Age | |
| Difference between areas | 0.104 |
| Standard Error ^a^ | 0.0298 |
| 95% Confidence Interval | 0.0510 to 0.150 |
| z statistic | 5.032 |
| Significance level | P < 0.0001 |
| SNORS ~ Pathologic N stage | |
| Difference between areas | 0.0601 |
| Standard Error ^a^ | 0.0219 |
| 95% Confidence Interval | 0.0346 to 0.0854 |
| z statistic | 3.873 |
| Significance level | P = 0.0004 |
| SNORS ~ Pathologic T stage | |
| Difference between areas | 0.0872 |
| Standard Error ^a^ | 0.0295 |
| 95% Confidence Interval | 0.068 to 0.108 |
| z statistic | 4.853 |
| Significance level | P < 0.0001 |
| SNORS ~ Age | |
| Difference between areas | 0.102 |
| Standard Error ^a^ | 0.0387 |
| 95% Confidence Interval | 0.0560 to 0.152 |
| z statistic | 5.278 |
| Significance level | P < 0.0001 |
| Pathologic N stage ~ Pathologic T stage | |
| Difference between areas | 0.0275 |
| Standard Error ^a^ | 0.0415 |
| 95% Confidence Interval | -0.0483 to 0.102 |
| z statistic | 1.720 |
| Significance level | P = 0.1089 |
| Pathologic N stage ~ Age | |
| Difference between areas | 0.0426 |
| Standard Error ^a^ | 0.0347 |
| 95% Confidence Interval | -0.0138 to 0.134 |
| z statistic | 1.595 |
| Significance level | P = 0.1108 |
| Pathologic T stage ~ Age | |
| Difference between areas | 0.0151 |
| Standard Error ^a^ | 0.0344 |
| 95% Confidence Interval | -0.0585 to 0.063 |
| z statistic | 0.217 |
| Significance level | P = 0.4012 |

^a^ DeLong et al., 1988

Comparison of AUC values in Figure 8m

| Variable 1 | ALL Combine |
| --- | --- |
| Variable 2 | Clinical Combine |
| Variable 3 | SNORS |
| Variable 4 | Pathologic N stage |
| Variable 5 | Pathologic T stage |
| Variable 6 | Age |
| Classification variable | OS status |

| Sample size | 336 |
| --- | --- |
| Positive group ^a^ | 146 (43.45%) |
| Negative group ^b^ | 190 (56.55%) |

^a^ fustat = 1
^b^ fustat = 0

| Variable | AUC | SE ^a^ | 95% CI ^b^ |
| --- | --- | --- | --- |
| ALL Combine | 0.791 | 0.0253 | 0.743 to 0.839 |
| SNORS | 0.717 | 0.0297 | 0.664 to 0.768 |
| Clinical Combine | 0.703 | 0.0278 | 0.652 to 0.754 |
| Pathologic N stage | 0.644 | 0.0259 | 0.592 to 0.696 |
| Pathologic T stage | 0.608 | 0.0242 | 0.553 to 0.664 |
| Age | 0.575 | 0.0261 | 0.520 to 0.629 |

^a^ DeLong et al., 1988

^b^ Binomial exact

## Pairwise comparison of AUC values

| ALL Combine ~ SNORS | |
| --- | --- |
| Difference between areas | 0.0745 |
| Standard Error ^a^ | 0.0225 |
| 95% Confidence Interval | 0.0367 to 0.111 |
| z statistic | 3.765 |
| Significance level | P < 0.0001 |
| ALL Combine ~ Clinical Combine | |
| Difference between areas | 0.0882 |
| Standard Error ^a^ | 0.0295 |
| 95% Confidence Interval | 0.0321 to 0.143 |
| z statistic | 4.147 |
| Significance level | P = 0.0002 |
| ALL Combine ~ Pathologic N stage | |
| Difference between areas | 0.147 |
| Standard Error ^a^ | 0.0238 |
| 95% Confidence Interval | 0.1055 to 0.189 |
| z statistic | 5.479 |
| Significance level | P < 0.0001 |
| ALL Combine ~ Pathologic T stage | |
| Difference between areas | 0.183 |
| Standard Error ^a^ | 0.0269 |
| 95% Confidence Interval | 0.121 to 0.249 |
| z statistic | 7.124 |
| Significance level | P < 0.0001 |
| ALL Combine ~ Age | |
| Difference between areas | 0.216 |
| Standard Error ^a^ | 0.0309 |
| 95% Confidence Interval | 0.156 to 0.276 |
| z statistic | 6.627 |
| Significance level | P < 0.0001 |
| SNORS ~ Clinical Combine | |
| Difference between areas | 0.0143 |
| Standard Error ^a^ | 0.0408 |
| 95% Confidence Interval | -0.0345 to 0.373 |
| z statistic | 0.488 |
| Significance level | P = 0.7856 |
| SNORS ~ Pathologic N stage | |
| Difference between areas | 0.0734 |
| Standard Error ^a^ | 0.0215 |
| 95% Confidence Interval | 0.0248 to 0.123 |
| z statistic | 3.758 |
| Significance level | P < 0.0001 |
| SNORS ~ Pathologic T stage | |
| Difference between areas | 0.109 |
| Standard Error ^a^ | 0.0257 |
| 95% Confidence Interval | 0.0618 to 0.156 |
| z statistic | 4.759 |
| Significance level | P < 0.0001 |
| SNORS ~ Age | |
| Difference between areas | 0.142 |
| Standard Error ^a^ | 0.0387 |
| 95% Confidence Interval | 0.0728 to 0.211 |
| z statistic | 6.089 |
| Significance level | P < 0.0001 |
| Clinical Combine ~ Pathologic N stage | |
| Difference between areas | 0.0593 |
| Standard Error ^a^ | 0.0386 |
| 95% Confidence Interval | -0.0385 to 0.156 |
| z statistic | 3.447 |
| Significance level | P = 0.0246 |
| Clinical Combine ~ Pathologic T stage | |
| Difference between areas | 0.0951 |
| Standard Error ^a^ | 0.0278 |
| 95% Confidence Interval | 0.0476 to 0.143 |
| z statistic | 3.985 |
| Significance level | P < 0.0001 |
| Clinical Combine ~ Age | |
| Difference between areas | 0.128 |
| Standard Error ^a^ | 0.0295 |
| 95% Confidence Interval | 0.0725 to 0.1850 |
| z statistic | 4.896 |
| Significance level | P < 0.0001 |
| Pathologic N stage ~ Pathologic T stage | |
| Difference between areas | 0.0365 |
| Standard Error ^a^ | 0.0351 |
| 95% Confidence Interval | -0.0159 to 0.090 |
| z statistic | 1.259 |
| Significance level | P = 0.1879 |
| Pathologic N stage ~ Age | |
| Difference between areas | 0.0697 |
| Standard Error ^a^ | 0.0347 |
| 95% Confidence Interval | -0.0215 to 0.161 |
| z statistic | 1.689 |
| Significance level | P = 0.0648 |
| Pathologic T stage ~ Age | |
| Difference between areas | 0.0335 |
| Standard Error ^a^ | 0.0364 |
| 95% Confidence Interval | -0.0525 to 0.120 |
| z statistic | 0.845 |
| Significance level | P = 0.3985 |

^a^ DeLong et al., 1988

Comparison of AUC values with our study published in JCMM

| Variable 1 | EMTscore |
| --- | --- |
| Variable 2 | SNORSscore |
| Classification variable | fustat |

| Sample size | 329 |
| --- | --- |
| Positive group ^a^ | 142 (43.16%) |
| Negative group ^b^ | 187 (56.84%) |

^a^ fustat = 1
^b^ fustat = 0

| Variable | AUC | SE ^a^ | 95% CI ^b^ |
| --- | --- | --- | --- |
| EMTscore | 0.723 | 0.0296 | 0.672 to 0.776 |
| SNORSscore | 0.717 | 0.0302 | 0.656 to 0.778 |

^a^ DeLong et al., 1988

^b^ Binomial exact

## Pairwise comparison of AUC values

| EMTscore ~ SNORSscore | |
| --- | --- |
| Difference between areas | 0.00572 |
| Standard Error ^a^ | 0.0352 |
| 95% Confidence Interval | -0.0632 to 0.0746 |
| z statistic | 0.163 |
| Significance level | P = 0.8707 |

^a^ DeLong et al., 1988
